# Mechanistic Insight into *Para*‐Substituent Control of Thermal Half‐Lives in Arylazopyrazole Photoswitches

**DOI:** 10.1002/anie.202514433

**Published:** 2025-09-04

**Authors:** Katharina Schlögl, Nadja K. Singer, Dominik Dreier, Hubert Kalaus, Rafaela C. O. Conceição, Marko D. Mihovilovic, Leticia González

**Affiliations:** ^1^ Institute of Applied Synthetic Chemistry TU Wien Getreidemarkt 9 Vienna 1060 Austria; ^2^ Institute of Theoretical Chemistry Faculty of Chemistry University of Vienna Währinger Str. 17 Vienna 1090 Austria; ^3^ Vienna Doctoral School in Chemistry (DoSChem) University of Vienna Währinger Str. 42 Vienna 1090 Austria; ^4^ Vienna Research Platform on Accelerating Photoreaction Discovery University of Vienna Währinger Str. 17 Vienna 1090 Austria

**Keywords:** Azo compounds, Density functional calculations, Isomerization, Photoswitches

## Abstract

Arylazopyrazoles are versatile photoswitches with excellent photochromic properties and tunable thermal half‐lives, yet the mechanistic role of substituents in controlling thermal stability remains poorly understood. Here, we synthesized an extensive library of arylazo‐1,3,5‐trimethylpyrazole photoswitches and rationalized the influence of *para*‐substituents on the thermal half‐lives, finding excellent agreement between calculated and measured trends. Calculations show that the electron‐donating and electron‐withdrawing nature of the substituents modulates the back‐isomerization process through at least two distinct mechanisms. Strong electron‐donating groups enhance delocalization at the azo moiety and thus favor a nonadiabatic out‐of‐plane rotational pathway via the lowest triplet state. In contrast, strong electron‐withdrawing groups reduce delocalization and promote a conventional ground state in‐plane inversion mechanism. Intermediate substituents exhibit a gradual shift that combines both major mechanisms. These findings provide prospects for rational design of responsive photoswitches with controllable thermal stability, essential for in vivo applications.

## Graphical abstract

Herein we provide an overview of photochromic and photophysical properties of a broad variety of arylazo‐1,3,5‐trimethylpyrazole photoswitches, including the influence of 23 different para‐substituents. Measured half‐lives correlated in a Hammet plot are rationalized by density functional theory calculations, revealing the underlying thermal isomerization mechanisms. The nature of the substituent controls the *Z/E* thermal isomerization pathway.

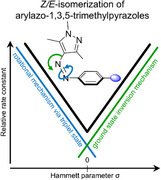

## Introduction

Photoswitchable molecules are of high value for a wide range of applications, including photopharmacology,^[^ ^1^, ^2^, ^3^ ^]^ data storage^[^ ^4^ ^]^ smart materials,^[^ ^5^, ^6^ ^]^ or energy storage.^[^ ^7^ ^]^ Azobenzenes,^[^ ^8^, ^9^, ^10^ ^]^ stilbenes,^[^ ^11^ ^]^ hemithioindigos,^[^ ^12^, ^13^ ^]^ related hemiindigos,^[^ ^13^, ^14^ ^]^ and several azoheteroaryls^[^ ^15^, ^16^, ^17^, ^18^, ^19^, ^20^ ^]^ are known photoswitches undergoing *E*/*Z* photoisomerism. Azobenzenes, with their high extinction coefficients, robust photostability across switching cycles, and ease of synthesis^[^ ^21^, ^22^ ^]^ are attractive for biology.^[^ ^23^, ^24^, ^25^ ^]^ However, their utility for in vivo applications is limited for two main reasons. They do not undergo complete *E/Z* isomerization and they back isomerize within few days at room temperature.^[^ ^26^, ^27^, ^28^ ^]^ These limitations have spurred the search of derivatives capable of achieving complete photoswitching and extended half‐life times.^[^ ^28^, ^29^ ^]^ Fluoroazobenzenes scaffolds stand out as exemplary photoswitches that exhibit complete bidirectional photoswitching along with exceptionably long thermal half‐lives of several years. ^[^ ^30^, ^31^, ^32^ ^]^

Most azoheteroaryl‐photoswitches described in the literature are either *N*‐heterocyclic or *S*‐heterocyclic derivatives of azobenzene.^[^ ^19^ ^]^ Introducing π‐donating heterocycles leads to blue‐shifted absorption spectra, while π‐acceptors lead to red‐shifted absorption spectra.^[^ ^33^ ^]^ Five‐membered rings give rise to transition states (TSs) that are sterically hindered in six‐membered azoheteroaryl‐photoswitches, which can facilitate long‐lived *Z*‐isomers.^[^ ^20^ ^]^ Substituting both aromatic rings with heterocycles can lead to unique combinations, forming push‐pull systems between an electron‐poor and an electron‐rich aryl, facilitating rapid rotational isomerization around the N─N bond.^[^ ^19^ ^]^

Arylazopyrazole‐photoswitches are emerging as attractive alternatives to azobenzenes because they possess well‐separated absorption bands that enable (near) quantitative photoswitching in both directions.^[^ ^15^, ^16^ ^]^ Moreover, their high versatility enables functionalizations that achieve half‐lives ranging from minutes^[^ ^20^, ^34^ ^]^ to nearly 1000 days.^[^ ^15^, ^16^ ^]^ For example, ether‐based arylazopyrazoles outperform earlier arylazopyrazole scaffolds in both quantitative switchability and extended thermal half‐lives.^[^ ^35^ ^]^ However, establishing clear correlations between thermal half‐lives and the underlying isomerization mechanisms remains a major challenge–hindering the rational design of photoswitches with tailored thermal stabilities. Depending on the substitution pattern of the (hetero)aromatic rings, different TSs have been reported. Long half‐lives found in unsubstituted arylazopyrazoles were attributed to a T‐shaped TS due to H–π interactions.^[^ ^20^ ^]^ In contrast, less stable out‐of‐plane TSs were found in systems containing di‐methyl substituted heteroaromatic rings.^[^ ^20^ ^]^ A comparison between *N*‐methylated pyrazole rings and *N*‐H pyrazoles showed that the free *N*‐H favors hydrogen‐bonding, thereby facilitating the tautomerization back‐switch mechanism leading to shorter half‐lives.^[^ ^34^ ^]^

Notably, even the thermal isomerization mechanism of the parent azobenzene has been the subject of new scrutiny,^[^ ^36^, ^37^ ^]^ underscoring the complexity of this process. Studies using Eyring transition state theory (TST) to calculate reaction rates systematically reported discrepancies between theoretical predictions and experimental observations.^[^ ^36^ ^]^ Mismatches have been often attributed to the level of theory–as reaction rate calculations are very sensitive to the accuracy of the activation barriers associated with the TSs. However, recent studies have shown that experimental half‐lives can be accurate reproduced when an unconventional rotational mechanism mediated by intersystem crossing (ISC) to triplet states is considered.^[^ ^36^, ^37^ ^]^ This rotation‐based ISC reaction mechanism–originally proposed over two decades ago^[^ ^38^ ^]^ yet largely overlooked–has been adopted as a reference framework to predict machine learned isomerization barriers across thousands of azobenzene derivatives.^[^ ^37^ ^]^ In contrast to the parent azobenzene,^[^ ^36^, ^37^ ^]^ we have demonstrated that in the arylazo‐1,3,5‐trimethylpyrazole (**3a**) photoswitch, two distinct thermal isomerization mechanisms are operative at room temperature.^[^ ^39^ ^]^ One mechanism is the rotation‐based ISC pathway, which accounts for ca. 25% of the isomerization, while a TS in the electronic ground state displaying inversion of a N atom is responsible for 75% of the observed half‐life.^[^ ^39^ ^]^

**Scheme 1:**
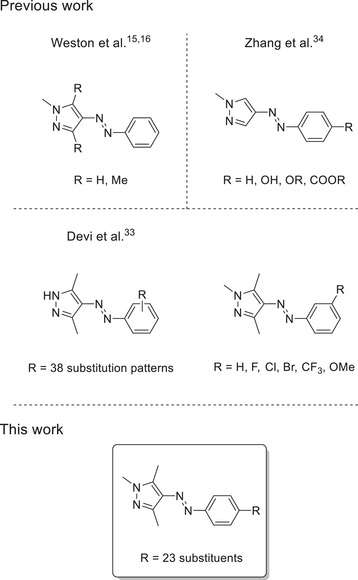
Arylazopyrazoles synthesized and investigated in the literature so far and their substitution patterns compared to this work.

Given that establishing correlations between molecular structure and thermal half‐lives is essential for rational photoswitch design, in this paper we investigate the influence of *para*‐substituents on the thermal half‐lives of arylazopyrazoles and elucidate their switching mechanisms. Although various substitution patterns for arylazopyrazole photoswitches have already been explored (Scheme 1), the isolated influence of different *para*‐substituents on the phenyl‐ring of this scaffold is not reported so far. The arylazo‐1,3,5‐trimethylpyrazole is chosen as scaffold to exclude stabilizing interactions through hydrogen bonding. Calculated half‐lives show excellent agreement with experimental values, supporting the validity of our mechanistic study. By identifying generalizable trends across *para*‐substituted compounds, our results contribute to the design of photoswitches with tailored and predictable thermal behavior, expanding the toolbox of heteroaryl systems for diverse applications.

## Results and Discussion

### Synthesis of Arylazopyrazoles

**Scheme 2:**
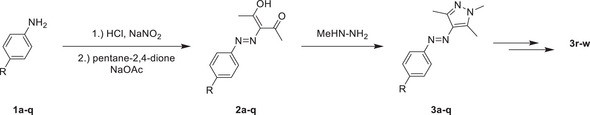
Synthesis of substituted arylazo‐1,3,5‐trimethylpyrazoles **3a–w**. The list of substituents can be found in Table S1. Conditions: (**3r**) **3h**, Na_2_S, THF/H_2_O 3/1, reflux, (**3s**) **3i**, K_2_CO_3_, Cs_2_CO_3_ then MeI, DMF, rt, (**3t**) **3k**, conc. H_2_SO_4_, 50 °C, (**3u**) **3j**, conc. H_2_SO_4_, EtOH, 60 °C, (**3v**), **3j**, EDCI•HCl, HOBt, DIPEA, then aniline, DMF, rt, (**3w**) **3j**, EDCI•HCl, HOBt, DIPEA, then benzylamine, DMF, rt.

We adapted a straightforward synthetic route formerly introduced by Weston et al. toward unsubstituted arylazopyrazoles.^[^ ^15^, ^16^, ^20^ ^]^ Based on an arylazopyrazole photoswitch that possesses a reasonably long thermal half‐life of 10 days^[^ ^15^, ^16^ ^]^ a compound library bearing different substituents in *para*‐position on the phenyl‐ring was prepared in typically two steps (Scheme 2 and Table S1). Starting from the respective anilines, we synthesized diazonium intermediates which, upon condensation with methylhydrazine, led to arylazotrimethylpyrazoles. The azo coupling in the first step worked in good to excellent yield (66%–92%) in most cases. Only synthesis of **2p** showed a lower yield of 44% as acidic catalyzed hydration led to degradation of the product species **2p** toward acetyl species **2q**. The obtained arylazopyrazole intermediates **2a–q** were then reacted with methylhydrazine to give the arylazopyrazoles **3a–q** in 71% to quantitative yield. Further arylazopyrazoles were synthesized by functional group interconversions from already prepared arylazopyrazole derivatives **3h**, **3i**, **3k**, and **3j**. Aniline derivative **3r** was synthesized upon reduction of the nitro group of **3h** by addition of Na_2_S. Methoxy compound **3s** was obtained by standard methylation conditions with methyl iodide starting from compound **3i**. Benzamide **3t** was prepared by hydrolysis of **3k** with conc. H_2_SO_4_ whereas benzamide derivatives **3v** and **3w** were prepared by standard EDCI amide coupling conditions starting from carboxylic acid derivative **3j**. Esterification of the carboxylic acid **3j** with ethanol led to ethyl ester derivative **3u**. All of these functional group interconversions were conducted with very good yields of at least 68%.

A total of 23 substituted arylazo‐1,3,5‐trimethylpyrazoles was then synthetized, for which the absorption spectra, half‐lives, and thermal switching behavior was investigated. Synthetic procedures and ^1^H and ^13^C spectra can be found in Sections S1 and S2.

### Absorption Spectra

The UV/Vis absorption spectra, photostationary states (PSSs) of the *Z*‐ and *E*‐isomers, and their thermal half‐lives were experimentally determined by irradiation with four different LEDs (365, 385, 400, and 460 nm), see Experimental Section below and Sections S3,S4 and S5. DMSO was used as solvent for all measurements because it is commonly used in photoswitchable studies and is a water‐miscible solvent, allowing compatibility with potential pharmacological uses.

**Figure 1:**
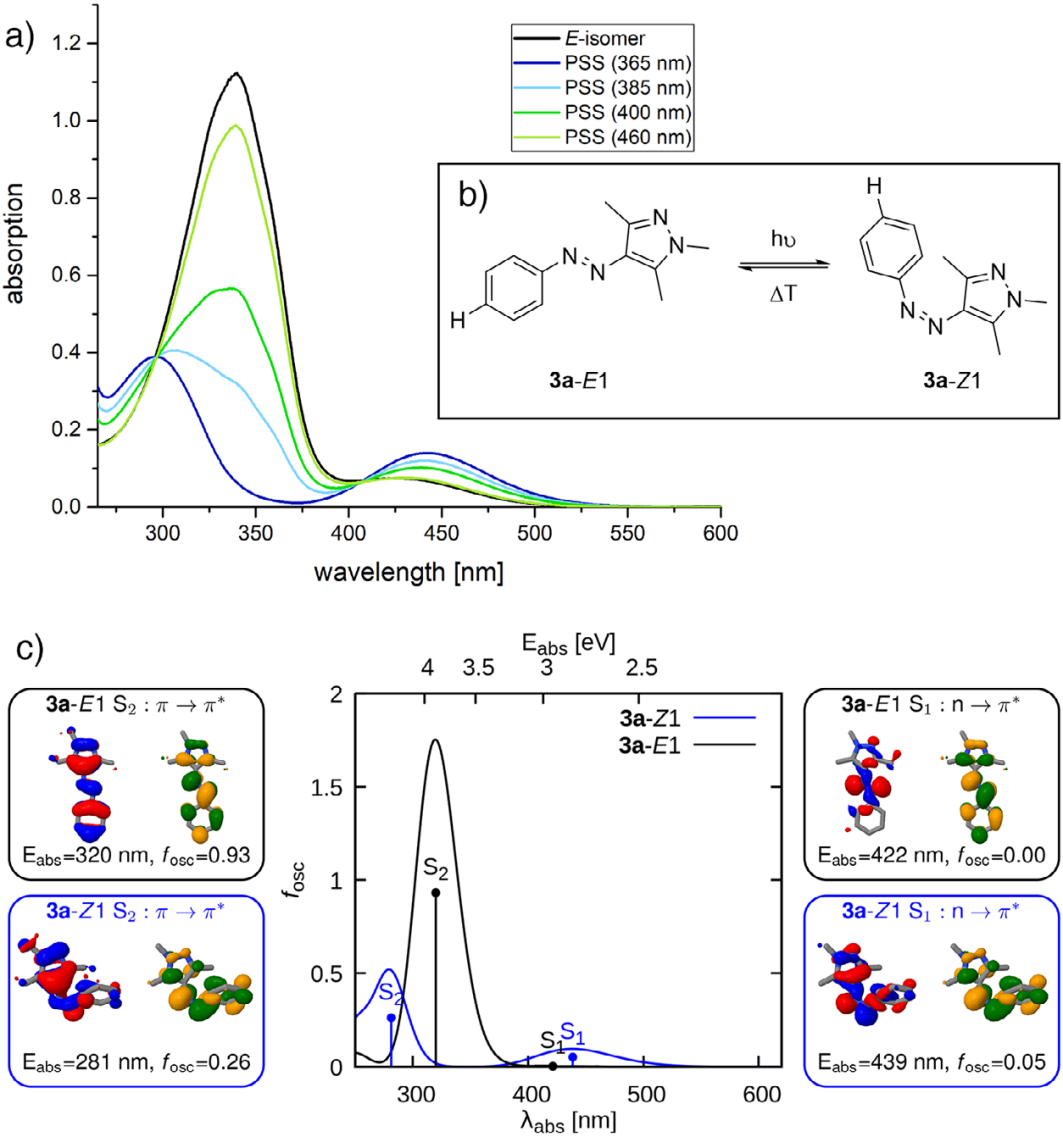
a) Typical absorption spectra, shown on the example of **3a**, of the unirradiated arylazopyrazole (*E* isomer in black) and the compound after irradiation with wavelengths as indicated (from 365 nm in blue to 460 nm in green). b) Isomerization scheme of compound **3a**. c) Calculated vertical excitations of **3a**‐*E*1 (black) and **3a**‐*Z*1 (blue) and natural transition orbitals involved in the S_0_→S_1_ and S_0_→S_2_ transitions. Absorption spectra are obtained convoluting with a Gaussian function of full width half maximum of 0.5 eV.

Most arylazopyrazoles achieve near‐full photoswitching (Table 1), underscoring the exceptional performance of this scaffold and its clear advantage over conventional azobenzenes.^[^ ^15^, ^16^ ^]^ The superiority of arylazopyrazoles in photoswitching toward the *Z*‐isomers is also reflected in their PSS UV/Vis spectra, exemplified in Figure 1a for compound **3a**. The absorption signature of the parent **3a** compound in its most stable *E*‐isomer (black) shows a high‐intensity peak at 340 nm, equaling the π–π· transition band, and a low‐intensity one at ∼425 nm, representing the n–π· transition band (see theoretical assignment below). Upon irradiation with 365 nm (our closest wavelength to the π–π· transition band at 340 nm), **3a**‐*E* isomerizes to the **3a**‐*Z* form (dark blue), shifting peaks to ∼300 nm (π–π·) and 442 nm (n–π·). Irradiation with the wavelengths 385, 400, and 460 nm leads to PSSS, which are mixtures of *E*‐ and *Z*‐isomer in ratios characteristic of the employed wavelength. These ratios reflect the contribution of photochemical *E*/*Z*‐isomerization and simultaneous photochemical *Z*/*E*‐isomerization by the respective wavelength.

**Table 1:** Overview of the observed photophysical properties of compounds **3a‐3 w**. Hammett parameters for the respective substituents were taken from the literature.

Compound	Substituent	*λ*_max_ (*E‐*π–π·)/nm	*λ*_max_ (*E‐*n–π·) / nm^a)^	*λ*_max_ (*Z‐*π–π·) / nm	*λ*_max_ (*Z‐*n–π·) / nm	k/s^−1^	*τ*_1/2_	Substituent Constant *σ* ^[^ ^40^ ^]^	Z% PSS (365 nm)
**3a**	H	340	425	296	442	7.6 · 10^−7^	10.5 d	0.00	99%
**3b**	F	340	∼425	297	441	7.7 · 10^−7^	10.5 d	0.06	98%
**3c**	Cl	346	∼425	300	445	1.5 · 10^−6^	5.5 d	0.23	97%
**3d**	Br	347	∼425	300	446	1.4 · 10^−6^	6 d	0.23	97%
**3e**	I	350	∼425	302	447	1.4 · 10^−6^	6 d	0.18	99%
**3f**	Me	343	∼425	300	444	1.3 · 10^−6^	6 d	−0.17	97%
**3g**	CF_3_	346	∼425	∼300^b)^	445	2.2 · 10^−5^	9 h	0.54	94%^c)^
**3h**	NO_2_	375	∼475	–^d)^	–^d)^	–^d)^	<1 s	0.78	–^d)^
**3i**	OH	352	∼430	–^d)^	–^d)^	–^d)^	<1 s	−0.37	–^d)^
**3j**	COOH	352	∼425	299	448	2.9 · 10^−5^	6.5 h	0.45	98%
**3k**	C≡N	356	∼430	305	451	5.0 · 10^−4^	23 min	0.66	97%
**3l**	NEt_2_	400	∼435	370	459	4.3 · 10^−5^	4.5 h	−0.83^e)^	87%^f)^
**3m**	Ph	357	∼435	∼305^b)^	450	3.5 · 10^−6^	2 d	−0.01	97%^c)^
**3n**	1‐naphthyl	375	∼430	314	455	2.4 · 10^−5^	8 h	–^g)^	94%^c)^
**3o**	2‐naphthyl	350	∼430	∼300^b)^	449	3.7 · 10^−6^	2 d	–^g)^	95%^c)^
**3p**	C≡CH	357	∼430	305	449	2.7 · 10^−6^	3 d	0.23	96%
**3q**	COCH_3_	357	∼430	305	451	2.1 · 10^−4^	55 min	0.50	97%
**3r**	NH_2_	385	∼425	350	461	7.2 · 10^−5^	2.5 h	−0.66	94%^h)^
**3s**	OCH_3_	349	∼425	308	448	2.2 · 10^−6^	3.5 d	−0.27	99%
**3t**	CONH_2_	351	∼425	299	448	9.4 · 10^−6^	1 d	0.36	99%
**3u**	COOEt	354	∼430	∼300^b)^	450	9.7 · 10^−5^	2 h	0.45	96%^c)^
**3v**	CONHPh	355	∼430	303	449	1.9 · 10^−5^	10 h	0.41	97%
**3w**	CONHBn	351	∼425	297	448	9.4 · 10^−6^	1 d	0.36^i)^	99%

^a)^ Value estimated, as *E*‐n–π· maxima occur as shoulders on the *E*‐π–π· absorbance.

^b)^ Value estimated, kink in spectrum, see Section S3.

^c)^ Minimum amount; see Section S4.

^d)^ Compounds **3h** and **3i** exhibited shorter half‐lives than the resolution of our instruments.

^e)^ Value for NMe_2_.

^f)^ *Z*‐content for PSS (400 nm).

^g)^ No value available.

^h)^ *Z*‐content for PSS (385 nm).

^i)^ Value for CONHMe.

After irradiation with 385 nm (light blue), the absorption spectrum is similarly shaped to the **3a**‐*Z* curve, reflecting a relatively high content of **3a**‐*Z* (75% *Z*‐isomer, see details in Section S4), while the absorption spectra after irradiation with 400 and 460 nm (green and light green) resemble more that of **3a**‐*E* (400 nm: 48% *E*‐isomer, 460 nm: 87% *E*‐isomer, see details in Section S4). This photoisomerization observed for the arylazopyrazole scaffold, where π–π· excitation drives *E*/*Z*‐isomerization and n–π· excitation drives *Z*/*E*‐isomerization, matches photochemical behavior known from azobenzenes.^[^ ^8^ ^]^

The decrease of the π–π·‐band upon irradiation with several wavelengths reflects the shrinking content of *E*‐isomer in the respective PSSs. The lowest *E*‐isomer content is reached after irradiation with 365 nm, where absorption at 340 nm gets as low as 0.06. We were able to observe PSSs with ≥94% *Z*‐content for nearly all our compounds. For most of them, 365 nm was the most suitable wavelength to reach the maximum *Z*‐content, whereas irradiation with 460 nm switched the compound back to majorly *E*‐isomer.

All investigated substituents lead to red‐shifts of the UV/Vis spectra compared to the parent compound **3a**. These shifts are generally more pronounced for the π–π· transition bands than for the n–π· transition bands (see Table 1 and Figure S1 A–D). The red‐shift of the π–π· bands is especially prominent in compounds bearing strong electron‐donating groups like **3f**, **3l**, **3q**, **3r,** and **3s**. The most substantial shift is observed for compound **3l**, where the *E*‐π–π· maximum is shifted by 60 nm (from 340 nm in **3a** to 400 nm in **3l**) and the *Z*‐π–π· maximum shifted by 74 nm (from 296 nm in **3a** to 470 nm in **3l)**. This can be attributed to the energy increase of the conjugated system's π orbital through the electron‐donating effect of the substituents (see more detailed discussion below). Electron‐withdrawing groups as present in **3g**, **3h**, **3p,** and **3u**, also induce red‐shifts in the absorption spectra by stabilizing and thereby lowering the π· orbital, though the effect is less pronounced than for electron‐donating groups (see Table 1 and Figure S1 A–D and discussion below).

While red‐shifted absorption spectra enable *E*/*Z*‐isomerization at lower‐energy light (longer wavelengths), preserving well‐separated *E*‐π–π· and *Z*‐n–π· absorption bands is essential to assure (near) quantitative bidirectional photoswitching. In most investigated *para*‐substituted derivatives, the corresponding energy gap between the *E*‐π–π· and *Z*‐n–π· transitions is reduced relative to the parent compound **3a**, slightly impairing their quantitative *E*/*Z*‐isomerization. This is reflected in lower *Z*‐isomer contents relative to compound **3a** as reported in Table 1. The effect is most pronounced in compound **3l**, where the strong red‐shift significantly narrows the gap between the relevant transition bands, resulting in a maximum observable *Z*‐content of only 87%.

A theoretical characterization of the absorption spectra of the **3a**‐*E* and **3a**‐*Z* isomers is done using time‐dependent density functional theory (TDDFT, see details in Section S6). Since the pyrazole moiety is not symmetric, **3a** possesses two conformers, labeled 1 and 2. Here, we discuss the results related to conformer 1 (**3a**‐*E1* and **3a**‐*Z1* in Figure 1b) while excited states of conformer 2 are reported in Section S6. Figure 1c depicts the calculated absorption spectra obtained from the S_0_→S_1_ and S_0_→S_2_ excitations (vertical sticks). The absorption bands at ∼400–500 nm correspond to the S_0_→S_1_ excitation for both **3a**‐*E*1 and **3a**‐*Z*1. According to the natural transition orbitals (NTOs), which identify the most significant hole–particle orbital pairs involved in a transition, this transition is assigned to the n–π·‐band known for other azobenzene derivatives.^[^ ^34^, ^41^ ^]^ In the equilibrium geometry of **3a**‐*E*1, the n–π· transition becomes symmetry forbidden, as a mirror plane through the azo‐benzene moiety exists resulting in zero intensity (*f*_osc_ = 0). However, enhanced intensity is expected, if vibrational and rotational conformers are included in the spectra. The NTOs also show that the second and more intense absorption band at ∼250–400 nm is due to a π–π· transition. In all the arylazopyrazoles, the intensity of the n–π·‐band increases upon E/Z‐isomerization, while the π–π·‐band decreases (Figure S1). These results nicely agree with the experimental bands shown in Figure 1a, for **3a**‐*E* and for **3a** at the PSS at 365 nm, respectively.

### Thermal Half‐Lives

All substituents lead to shorter half‐lives than the unsubstituted **3a** parent system (Table 1). This behavior is similar to that reported in azobenzenes,^[^ ^27^ ^]^ where regardless of the electronic effects imposed by *para*‐substituents, the thermal back‐switch was always accelerated. Such behavior has been attributed to stabilization of the ground state TS involved in the back‐isomerization mechanism.^[^ ^26^ ^]^ However, our previous study on the unsubstituted **3a** compound revealed that the half‐live at room temperature is governed not only by a ground state isomerization pathway but also by a mechanism including both singlet and triplet states.^[^ ^39^ ^]^ Therefore, the prevailing rationale for shorter half‐lives, based solely on ground state stabilization, is questionable.

**Scheme 3:**
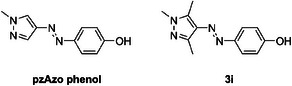
Structures of **pzAzo phenol** and compound **3i**.

The measured half‐lives for the substituted arylazopyrazoles range from a couple of days and hardly any loss in *Z*‐isomer stability (e.g., F, Cl, Br, I, and Me) to significantly accelerated back‐switches with half‐lives less than an hour (e.g., CN and COCH_3_). For compounds **3h** and **3i,** bearing NO_2_ and OH groups, no changes could be observed in the UV/Vis spectra upon irradiation, indicating that thermal relaxation occurs faster than the temporal resolution of our UV/Vis spectrophotometer. The ultrafast relaxation processes of these compounds can be attributed to an azo‐hydrazone tautomerism accompanied by a facilitation of the rotation around the N─N bond.^[^ ^42^, ^43^, ^44^ ^]^ The nitro‐compound **3h** shows a very strong electron withdrawing effect, speeding up the relaxation process by reducing electron density at the N─N bond. Phenol **3i** undergoes azo‐hydrazone tautomerism that enables facile rotation around the N─N bond. This observation goes along the reports of Crespi and Zhang^[^ ^19^, ^35^ ^]^ where a half‐life of 5 min in MeCN for compound **pzAzo phenol** (Scheme 3) was reported. As our compound **3i** shows double methylation at positions 3 and 5, a faster thermal back‐isomerization in comparison to **pzAzo phenol** is reasonable, as **3i** has no proton available to interact with the π‐system of the phenyl‐rings to stabilize the *Z*‐isomer.^[^ ^20^ ^]^

**Figure 2:**
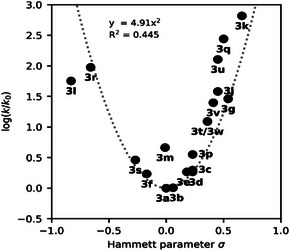
Hammett plot of the investigated arylazopyrazole compounds. The V‐shape of the plot is indicated with a parabolic fit (4.91 · x^2^) with compound **3a** set to be the minimum of the curve. The coefficient of determination (*R* ^2^) is given as 0.445.

Next, we sought to correlate the differences in thermal half‐lives with specific electronic and structural properties of the respective substituents. Since both electron withdrawing and electron donating substituents resulted in shorter thermal half‐lives, we investigated whether this effect can be linked to the resonance properties of the substituents. To this end, the Hammett equation (Equation 1) was applied and the relative logarithmic rate constant log(*k*/*k*_0_), with *k*_0_ being the rate constant of the unsubstituted compound **3a**, was plotted against the tabulated Hammett substituent constant *σ*,^[^ ^40^ ^]^ giving the plot shown in Figure 2.

(1) $$\log\left(\frac{k}{k_{0}}\right)=\rho \cdot \sigma =\rho \cdot \log\left(\frac{K}{K_{0}}\right)$$

The convex or V‐shape of this plot indicates that any *para*‐substituent–through its mesomeric or resonance interaction—accelerates the relaxation from *Z*‐ to *E*‐isomer, as observed in azobenzenes.^[^ ^27^ ^]^ Moreover, a correlation between strength of the resonance and the amount of acceleration is apparent: The greater the deviation of the substituent constant *σ* from zero, the faster the thermal back‐switch appears to proceed. Although the Hammett equation is unable to describe the experimental data for some compounds (eg. biphenyl **3m**), the general change of slope indicates a change in the reaction mechanism depending on the nature of the substituent. In order to rationalize the behavior of the V‐shaped Hammett plot, we turn to computationally investigate the thermal isomerization mechanisms and associated half‐lives of selected compounds.

### Thermal Isomerization Mechanisms

**Figure 3:**
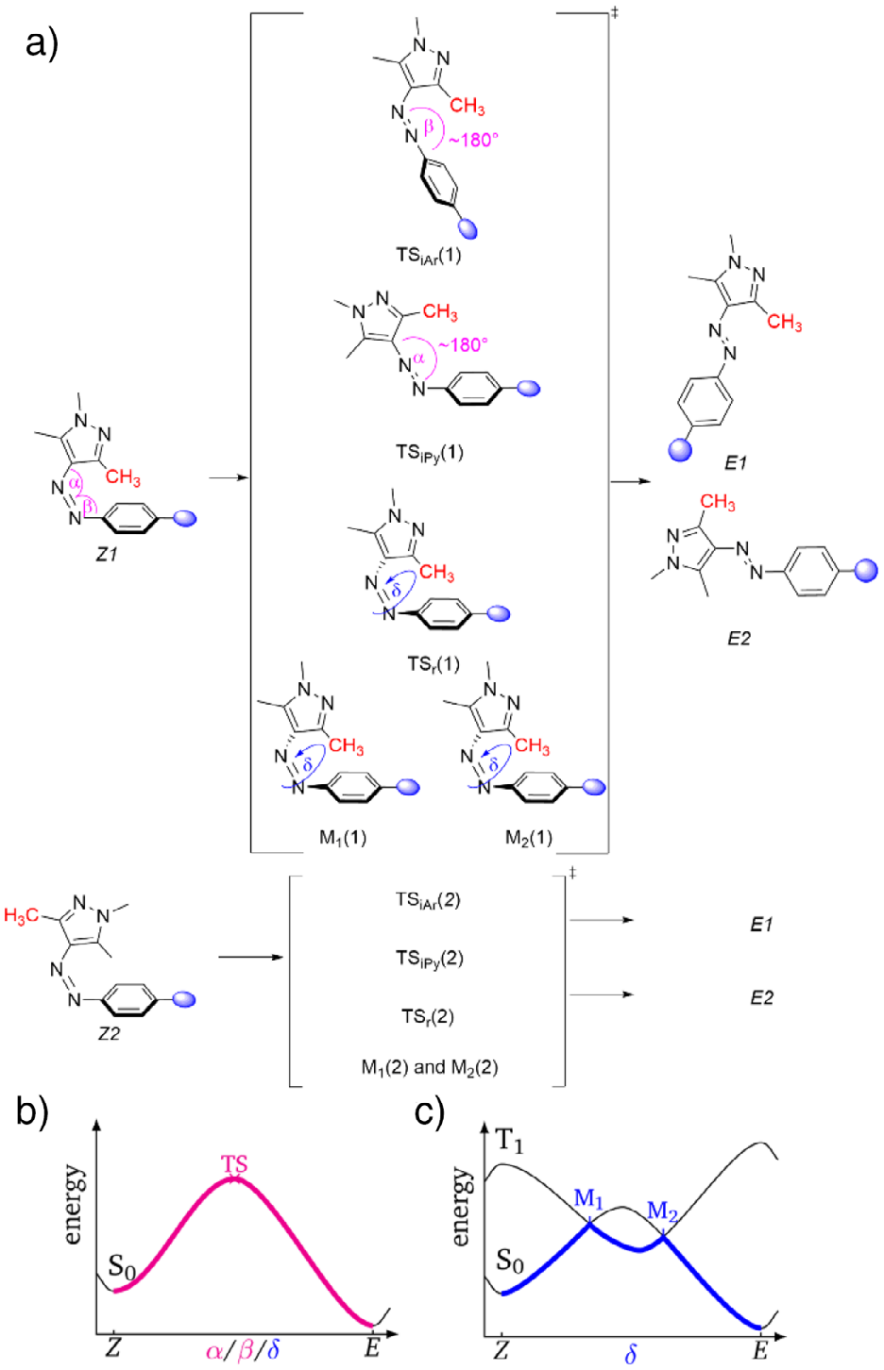
a) Lowest‐energy *Z/E* thermal isomerization pathways for both conformers (*Z1* and *Z2*) of the arylazopyrazoles. Chemical structures for *Z2* are omitted for clarity. The in‐plane inversion of the aryl and pyrazole moiety transition state (TS) is denoted as TS_iAr_ and TS_iPy_, respectively. The out‐of‐plane rotational TS is labelled TS_r_. The out‐of‐plane rotational isomerization involving the triplet state involves two minimum energy crossing points (MECPs) M_1_ and M_2_. b) Schematic *Z/E* isomerization along a TS in the electronic singlet ground state. c) Schematic *Z/E* isomerization involving a singlet and triplet state, and characterized by the two MECPs M_1_ and M_2_.

**Figure 4:**
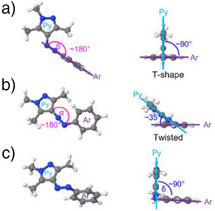
Side and front views of the ground‐state transition state geometries involved in the thermal isomerization of the unsubstituted arylazopyrazole (**3a**): a) TS_iAr_(1), b) TS_iPy_(1), and c) TS_r_(1).

In arylazopyrazoles, four distinct thermal isomerization mechanisms, summarized in Figure 3a, are possible.^[^ ^39^ ^]^ The first three are conventional pathways occurring in the electronic singlet ground state via TSs, as sketched in Figure 3b. One involves the in‐plane inversion of the aryl moiety around the neighboring azo nitrogen (Path_iAr_). The corresponding TS is labelled TS_iAr_ and is characterized by a T‐shaped structure with an 90° dihedral angle between the aryl and the pyrazole rings and an 180° β‐angle (Figure 4a). Likewise, there is a mechanism related to the in‐plane inversion of the pyrazole moiety around its neighboring azo nitrogen (Path_iPy_). In this case, the geometry of the TS_iPy_ is twisted with a dihedral angle of around 35° between the rings and an 180° α‐angle (Figure 4b). The third pathway in the singlet ground state is an out‐of‐plane rotational pathway around the azo‐bond (Path_r_). The corresponding TS_r_ is characterized by a *δ* dihedral angle around the azo‐bond of approximately 90° (Figure 4c). The fourth pathway is a more complicated, nonconventional rotational mechanism that involves ISC to the lowest triplet state (Path_rT1_). In this case, the rotational isomerization proceeds from the *Z*‐isomer along a nonadiabatic crossing from the singlet ground state to the lowest‐lying triplet state at a minimum energy crossing point (MECP, M_1_), as sketched in Figure 3c. From the triplet state, the molecule then crosses back to the singlet surface through a second MECP (M_2_), before finally reaching the *E*‐isomer. Since the pyrazole moiety is not symmetric, each of the four pathways can proceed through two conformers that we labeled (1) and (2) (see Figure 3a).

Motivated by the obtained experimental correlations between the Hammett parameter *σ* and the relative rate constant, we selected nine arylazopyrazoles (**3r**, **3s, 3f, 3m, 3a**, **3e, 3c**, **3p, 3g**) spanning the range ‐0.66 ≤ *σ* ≤ 0.54 to investigate the four isomerization pathways. The half‐lives related to the ground‐state isomerization mechanisms Path_iAr_, Path_iPy,_ and Path_r_ are calculated with conventional TST,^[^ ^45^, ^46^ ^]^ where the rate constant of the thermal back isomerization–and thus the half‐life—depends on the Gibbs free energy difference (ΔΔ*G* ^‡^) between the *Z*‐isomer and the TS of the isomerization pathway *en route* to the *E*‐isomer. Since Path_rT1_ involves singlet and triplet states and the associated MECPs, we resort to nonadiabatic TST (NA‐TST).^[^ ^36^, ^37^, ^39^, ^47^ ^]^ In NA‐TST the rate constant depends not only on the ΔΔ*G* ^‡^ between the *Z*‐isomer and the MECPs but is also modulated by the spin‐orbit couplings (SOCs) at the crossing points. The value of the SOCs can be seen as a measure of how easy ISC at the MECPs can occur. We thus set to investigate the four *Z/E*‐isomerization mechanisms to obtain the associated rate constants by conventional TST and NA‐TST. Accordingly, for each derivative, we optimized two *Z*‐conformers, two *E*‐conformers, six TSs (2 TS_iAr_, 2 TS_iPy_, and 2 TS_r_), and four MECPs (2 M_1_ and 2 M_2_) with DFT in implicit DMSO (geometries can be found in repository^[^ ^48^ ^]^). The Gibbs free energies are then calculated including thermal corrections to the total partition function at 296.15 K. The relative Gibbs free energies are given in Table S4.

**Figure 5:**
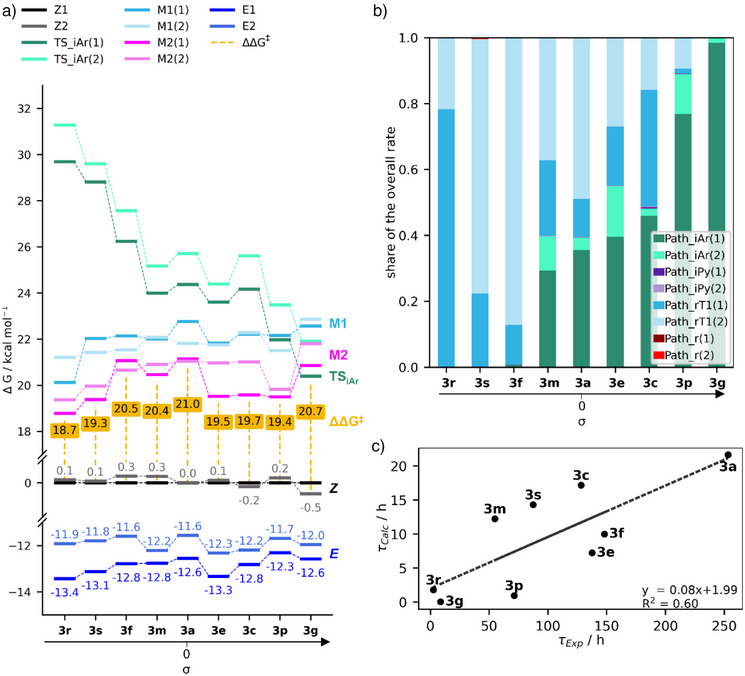
a) Gibbs free energy (Δ*G*) plot of all *Z* (black/gray), *E* (dark blue), TS_iAr_ conformers (green and turquoise), and minimum energy crossing points (M1 and M2, light blue shades, pink shades) at the ωB97X‐D/def2‐TZVP@SMD(DMSO) level of theory. Energies of additional ground‐state inversion and rotational TSs are in Figure S4. All energies are plotted relative to the respective *Z1* conformer. The Gibbs free energy difference (ΔΔ*G* ^‡^, yellow) is calculated as the difference between the Boltzmann weighted average of the *Z* conformers and the lowest TS/MECP energy. The molecules are sorted by Hammett parameter σ values. Dotted lines between energy levels have no physical meaning but are shown as visual guide. b) Composition of the calculated overall rate constant in terms of pathways. The shares of the rate constants for all four pathways for both conformers are shown in green/turquoise (Path_iAr_), violet shades (Path_iPy_, only visible in **3c**), light blue shades (Path_rT1_), and red shades (Path_r_, negligible). c) Experimental against computational half‐lives in hours with a linear fit.

The calculated Δ*G* energies of all the structures for each of the 9 arylazopyrazoles are displayed in Figure S4. Figure 5a shows the energies of the most relevant structures. The *Z1‐* and *Z2*‐conformers (black/gray) are predicted almost degenerated in energy and likewise, the energies of the *E1* and *E2* structures (dark blue) differ at most by 2 kcal mol^−1^. For clarity, the Gibbs free energy of the lowest *Z/E*‐isomerization barrier (ΔΔ*G* ^‡^) is highlighted in yellow.

The Δ*G* of all ground state pathways (Path_iAr_, Path_iPy_, and Path_r_) overall decreases with increasing Hammett parameter. However, while for the Path_iPy_ there is a shallow descent, the slope in Path_iAr_ and Path_r_ is much steeper (see Figure S4). By contrast, the nonadiabatic Path_rT1_ (represented by M_1_ and M_2_) increases slightly with increasing Hammett parameter. This fact must lead to a switch in the main operating mechanisms with changing Hammett parameters. However, it is not easy to identify which pathway controls the thermal isomerization by looking only at the Δ*G* of all pathways. While for conventional TST the energy of the corresponding TS is the rate limiting step that dictates the rate constant and associated half‐lives, for NA‐TST the SOCs also have to be taken into account.^[^ ^39^, ^40^, ^45^, ^46^ ^]^ The overall rate constant is thus determined by summing over all four (Path_iAr_, Path_iPy_, Path_r_, and Path_rT1_) independent parallel paths for each conformer separately and then summing the Boltzmann weighted (according to the *Z*‐conformer energies) results of both conformers. Figure 5b shows the shares of the calculated Path_iAr_, Path_iPy_, Path_r_, and Path_rT1_ rate constants to the overall rate constant for each compound. Clearly, the two pathways (1) and (2) for each of the conformers of Path_iAr_ (green shades) and Path_rT1_ (blue shades) contribute the most to the overall rate constants, while Path_iPy_ (violet shades) and Path_r_ (red shades) are negligible.

However, there are striking differences along the Hammett parameter scale for the two dominant isomerization reactions. In the large negative σ scale (strong electron donating groups), i.e., in **3r**, **3s**, and **3f**, the operating mechanism is Path_rT1_, while for large positive *σ* (strong electron withdrawing groups), as in **3g** and **3p**, the leading contribution is Path_iAr_. In molecules with intermediate values σ, like **3m**, **3a**, **3e**, and **3c,** there seems to be a mixture of Path_iAr_ and Path_rT1_.

**Scheme 4:**
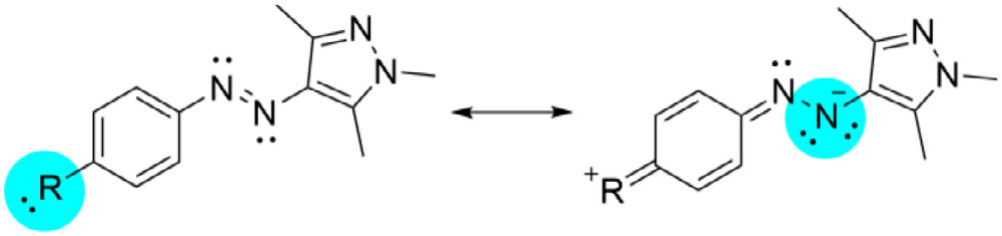
Resonance structures of *para*‐substituted AAPs.

The observed preferences for distinct isomerization mechanisms can be rationalized by examining the influence of substituent electronic effects. Electron‐donating groups inject electron density into the aromatic ring, which subsequently conjugates with the azo group. This shift in electron density can be visualized through the resonance structures depicted in Scheme 4. To quantify this characteristic shift of electrons, particularly into the pyrazole‐adjacent nitrogen atom of the azo bond for electron‐donating substituents, we employed Mulliken population analysis (Figure S5). This electron density redistribution is especially prevalent in geometries relevant to the rotational isomerization pathways Path_r_ and Path_rT1_ (TS_r_, M_1_, and M_2_). Furthermore, we investigated the Wiberg bond index (WI) as a quantitative measure of electron delocalization (Figure S6). Our analysis revealed a significant increase in azo moieties WI, indicating enhanced electron delocalization, particularly for the TS_r_ geometries. This increasing electronic delocalization enhances π conjugation, which favors a rotational isomerization mechanism. While electron‐donating groups primarily raise the energy of the π orbital of the conjugated system, the extended conjugation and delocalization over the azo–aryl system inherently stabilizes the π· orbital and the triplet state overall. The nonbonding n orbital on the nitrogen is less affected by electron‐donating groups, or may be slightly lowered in energy due to secondary inductive effects. Consequently, the delocalization leads to a smaller n → π· energy gap, resulting in a lower‐energy triplet state. This provides a clear explanation for why derivatives bearing strong electron‐donating groups (characterized by more negative *σ* values) exhibit a preferred nonadiabatic rotational isomerization pathway via the triplet state.

In contrast, electron‐withdrawing groups pull electron density away from the aromatic ring. This reduces conjugation and increases localization on the azo moiety, a trend also evident in the Mulliken population analysis (Figure S5) and the WI (Figure S6). This diminished delocalization has a distinct effect: it stabilizes the π· orbital more significantly than the non‐bonding n orbital. This ultimately leads to an increased n → π· energy gap and thus a higher‐energy triplet state. The localization of the excitation and the reduced overlap between the aryl ring and the azo unit both contribute to a diminished stabilization of the triplet state. As a direct result, the nonadiabatic rotational mechanism via triplet intermediates is expected to be less favored in arylazopyrazoles bearing electron‐withdrawing substituents. Accordingly, the stronger the electron‐withdrawing effect (indicated by a more positive Hammett *σ* value), the more likely isomerization is to proceed via the singlet‐state inversion pathway.

**Figure 6:**
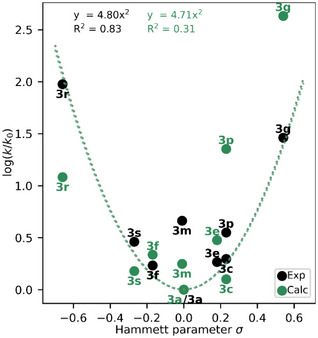
Hammett plot of the investigated nine arylazopyrazole compounds with experiment (black) and calculations (green). The convex shape of the plots is indicated with a parabolic fit (experiment: 4.80 · x^2^, calculated: 4.71 · x^2^) with compound **3a** set to be the minimum of the curve. The coefficient of determination (*R* ^2^) is given.

Both extremes in the Hammett plot lead to shorter half‐lives, whereas intermediate compounds, influenced by a mixture of the Path_rT1_ and the Path_iAr_ display longer half‐lives, resulting in the convex Hammet plot of Figure 2. Our calculated half‐lives are compared with the experimental values in Figure 5c. In general, the calculated absolute rates are predicted to be faster than the experimental ones. This is expected as TST (and NA‐TS) uses several approximations, therefore the calculated rates should be seen as an upper limit. Compared to a perfect fit (1x + 0), we obtain a fit for the calculated absolute half‐lives of 0.08x + 1.99 with a coefficient of determination *R* ^2 ^= 0.60. This means that the calculated half‐lives are ∼12.5 times shorter than the experimental ones. One should keep in mind that because the half‐lives are inversely proportional to the rate constants, which exponentially depend on the Gibbs free energy barrier, a small variation <1 kcal mol^−1^ in the energy barrier leads to a change of few folds in the half‐life. To remove this systematic error from the calculations, we use a similar plot as Figure 2, where the calculated overall rate constants for the nine investigated compounds are plotted against σ relative to **3a**, together with the corresponding experimental values, see Figure 6. The fact that we obtain the same convex shape and nearly identical parabolic fit function (4.80x^2^ versus 4.71x^2^) when compound **3a** is set at the minimum of the curve confirms that our predicted mechanisms are responsible for the thermal half‐lives of arylazopyrazoles. In general, we are able to reproduce the experimental parabolic plot, even if the calculated rates tend to underestimate the rate for negative *σ* and therefore the rate of Path_rT1,_ and overestimate the rate for positive σ and thus the rate of Path_iAr_. The combination of errors leads to an overall error cancelation in the fit function, even if the *R* ^2^ of 0.31 is worse. However, it is gratifying to see that the experimental convex Hammett plot can be replicated with our calculations. In contrast, omitting the nonadiabatic Path_rT1_ results in a linear plot with drastic deviations for *σ* ≤ 0 (Figure S7), highlighting the importance of including the triplet state mechanism, as well as considering not only energetics but also SOCs. Our conclusive predictions of the half‐lives will be used in the future to expand the library of arylazopyrazole compounds with desired half‐lives by directing the synthesis.

For the sake of completeness, we discuss in Section S7 that hydrogen bonding, concentration dependence, and solvent pH do not influence half‐lives–contrary to what it has been suggested in other studies.

## Conclusions

We have presented an extensive library of *para*‐substituted arylazopyrazoles photoswitches, with their associated thermal half‐lives. Irrespective of whether the substituents are electron ‐withdrawing or ‐donating, a faster thermal isomerization compared to the unsubstituted compound is observed. However, correlation of the thermal isomerization rates with Hammett parameters revealed a characteristic convex V‐shaped plot, similar to that reported in azobenzenes,^[^ ^27^ ^]^ meaning that different mechanisms are operative at the two extremes of the substitution scale.

We employed quantum chemical calculations to elucidate the mechanisms underlying the thermal isomerization process. Intriguingly, and different from what has been recently proposed in azobenzene derivatives,^[^ ^36^, ^37^ ^]^ we find that different pathways are operative. Compounds with large positive Hammett parameters (and thus electron‐withdrawing substituents) back isomerize mainly via an in‐plane inversion of the aryl moiety in the electronic ground state. By contrast, those with large negative Hammett parameters (electron‐donating substituents), isomerize mainly via an out‐of‐plane rotational nonadiabatic pathway involving intersystem crossing to the lowest triplet state. Compounds with Hammett parameters around ∼0 are driven by a combination of both mechanisms. These trends can be rationalized by the influence of the substituents on the electronic delocalization at the azo group, which modulates the relative favorability of the inversion and rotational pathways: electron‐donating groups enhance delocalization and favor triplet‐assisted rotation, while electron‐withdrawing groups reduce delocalization and favor singlet‐state inversion. This mechanistic shift explains the convex correlation between Hammett parameters and thermal isomerization rates, and paves the way for rational property‐driven compound design, concomitantly lowering synthetic efforts.

## Experimental Section

Unless otherwise noted, chemicals were purchased from commercial suppliers and used without further purification. The purity of the reported compounds is >95% according to NMR. NMR spectra were recorded on a Bruker *AC 200* (^1^H: 200 MHz, ^13^C: 50 MHz), Bruker *Avance Ultrashield 400* (^1^H: 400 MHz, ^13^C: 101 MHz) and Bruker *Avance IIIHD 600* spectrometer equipped with a Prodigy BBO cryo probe (^1^H: 600 MHz, ^13^C: 151 MHz). Chemical shifts are given in parts per million (ppm) and were calibrated with internal standards of deuterium labeled solvents CDCl_3_ (^1^H 7.26 ppm, ^13^C 77.16 ppm) and DMSO‐*d_6_* (^1^H 2.50 ppm, ^13^C 39.52 ppm). NMR assignments of unknown compounds were confirmed by ^1^H–^1^H COSY, ^1^H–^13^C, HSQC and ^1^H–^13^C, HMBC and by comparison to predicted spectra. TLC was performed using silica gel 60 aluminum plates containing fluorescent indicator from Merck and detected with UV light at 254 nm. HPLC chromatography was carried out with an Auto purification system of Waters using an ACQUITY QDa Detector in combination with a 2998 Photodiode Array Detector. Analytical separation was conducted using XSELECT CSH Fluoro‐Phenyl 5 µm 4.6 x 150 mm and XSELECT CSH C18 5 µm 4.6 x 150 mm columns. Preparative separation was performed using XSELECT CSH Prep Fluoro‐Phenyl 5 µm 30 x 150 mm and XSELECT CSH Prep C18 5 µm OBD 30 x 150 mm columns. As solvents HPLC grade methanol and HPLC grade H_2_O were used containing 0.1% formic acid. Flash column chromatography (FC) was carried out with a Büchi Sepacore MPLC system using silica gel 60 M (particle size 40–63 µm, 230–400 mesh ASTM, Macherey Nagel, Düren). GC/MS spectra were measured on a Thermo Trace 1300 / ISQ LT (single quadrupole MS (EI)) using a standard capillary column BGB 5 (30 m x 0.25 mm ID). Melting points were determined by a Leica Galen III Kofler and a Büchi Melting Point B‐545. An Agilent 6230 LC TOFMS mass spectrometer equipped with an Agilent Dual AJS ESI‐Source was used for HR‐MS analysis. The mass spectrometer was connected to a liquid chromatography system of the 1100/1200 series from Agilent Technologies, Palo Alto, CA, USA. The system consisted of a 1200SL binary gradient pump, a degasser, column thermostat, and an HTC PAL autosampler (CTC Analytics AG, Zwingen, Switzerland). A silica‐based Phenomenex C‐18 Security Guard Cartridge was used as stationary phase. Data evaluation was performed using Agilent Mass Hunter Qualitative Analysis B.07.00. Identification was based on peaks obtained from extracted ion chromatograms (extraction width ± 20 ppm). Photophysical properties of the compounds were measured on a UV‐1800 UV/Vis spectrophotometer from Shimadzu at 23 °C. Spectra were recorded in a range from 265 to 600 nm. For irradiation of the samples, OmniCure LED heads of 365, 385, 400, and 460 nm were used set to 100% power (OmniCure LX400, max. power 320 mW). Samples of 50 µM in DMSO were typically irradiated for 5 s from the top to reach the PSS. Detailed synthetic protocols and (photophysical) compound characterization can be found in the Supporting Information.

## Supporting Information

The authors have cited additional references within the Supporting Information.^[^ ^49^, ^50^, ^51^, ^52^, ^53^, ^54^, ^55^, ^56^, ^57^, ^58^, ^59^, ^60^, ^61^, ^62^, ^63^, ^64^, ^65^, ^66^ ^]^

## Conflict of Interests

The authors declare no conflict of interest.

## Supporting information



Supporting Information

## Data Availability

The data that support the findings of this study are openly available in Phaidra at 10.25365/phaidra.691.
