# Supplementary material for: Mechanistic Insight into Para‐Substituent Control of Thermal Half‐Lives in Arylazopyrazole Photoswitches
Source: Angew Chem Int Ed Engl. 2025 Sep 4;64(43):e202514433. doi: 10.1002/anie.202514433 (PMC12535399; doi:10.1002/anie.202514433)
Supplement: Supplementary file 1 — Supporting Information [file ANIE-64-e202514433-s001.pdf]

SUPPORTING INFORMATION

Mechanistic Insight into *para*-Substituent Control of Thermal Half-Lives in Arylazopyrazole Photoswitches

Katharina Schlögl,[a]† Nadja K. Singer,[b,c]† Dominik Dreier,[a] Hubert Kalaus,[a] Rafaela C. O. Conceição,[a] Marko D. Mihovilovic,*[a] and Leticia González*[b,d]

[a] Dr. K. Schlögl, Dr. D. Dreier, H. Kalaus, Dr. R. C. O. Conceição, Prof. Dr. M. D. Mihovilovic
Institute of Applied Synthetic Chemistry
TU Wien
Getreidemarkt 9, 1060 Vienna, Austria
E-mail: marko.mihovilovic@tuwien.ac.at

[b] N. K. Singer, Prof. Dr. L. González
Institute of Theoretical Chemistry, Faculty of Chemistry
University of Vienna
Währinger Str. 17, 1090 Vienna, Austria
E-mail: leticia.gonzalez@univie.ac.at

[c] N. K. Singer
Vienna Doctoral School in Chemistry (DoSChem)
University of Vienna
Währinger Str. 42, 1090 Vienna, Austria

[d] Prof. Dr. Leticia González
Vienna Research Platform on Accelerating Photoreaction Discovery
University of Vienna
Währinger Str. 17, 1090 Vienna, Austria

†K.S. and N.K.S: contributed equally.

Table of contents

SECTION S1: SYNTHETIC PROCEDURES..3

SECTION S2: ^{1}H AND ^{13}C SPECTRA..14

SECTION S3: EXPERIMENTAL UV/VIS SPECTRA..55

SECTION S4: CALCULATION OF PSS..59

SECTION S5: EXPERIMENTAL KINETIC DATA..62

SECTION S6: COMPUTATIONAL DETAILS..68

SECTION S7: OTHER POTENTIAL INFLUENCES..74

REFERENCES:..75

SECTION S1: SYNTHETIC PROCEDURES

General procedure A: Synthesis of arylazopyrazole intermediates (2)

Arylazopyrazole intermediates **2** were synthesized according to a modified literature procedure.[1] $NaNO_2$ (1.2 equiv.) in H_2O (2.5 mL) was added dropwise to a solution of the respective aniline **1** (1 equiv.) in acetic acid (10 mL) and conc. HCl (1.7 mL) at 0°C. The resulting solution was stirred at 0°C for 1 hour. Then, the solution was added to a suspension of NaOAc (3 equiv.) and acetylacetone (1.3 equiv.) in EtOH (7 mL) and H_2O (4 mL). The reaction mixture was stirred at room temperature for 1 hour. The precipitate was collected by filtration, washed with little cold H_2O followed by little cold H_2O/EtOH (1/1) and dried *in vacuo* to afford the azo intermediate **2**.

General procedure B: Synthesis of arylazopyrazoles (3)

Methylhydrazine (1 equiv.) was added to a solution of the corresponding azo intermediate **2** (1 equiv.) in EtOH and the resulting solution was stirred at reflux temperature for 3 hours. Evaporation of the solvent *in vacuo* afforded the desired product **3**.[1]

Arylazopyrazoles (3)

4-Hydroxy-3-(phenyldiazenyl)pent-3-en-2-one (2a):

General procedure A; using aniline **1a** (0.26 mL, 7.40 mmol) with the temperature during the formation of the diazonium salt being held at -5°C giving yellow crystals (73%, 1.10 g); mp: 86.0 – 86.5°C. ^{1}H-NMR (200 MHz, $CDCl_3$): δ = 2.49 (s, 3H), 2.61 (s, 3H), 7.13 – 7.25 (m, 1H), 7.36 – 7.45 (m, 4H), 14.73 (br s, 1H) ppm. ^{13}C-NMR (101 MHz, $CDCl_3$): δ = 26.7 (q, 1C), 31.7 (q, 1C), 116.3 (d, 2C), 126.0 (d, 1C), 129.7 (d, 2C), 133.3 & 141.6 (s, 2C), 197.1 & 198.0 (s, 2C) ppm.

1,3,5-Trimethyl-4-(phenyldiazenyl)-1*H*-pyrazole (3a):

General procedure B; using azo intermediate **2a** (1.00 g, 4.90 mmol) and EtOH (25 mL) giving yellow crystals (quant., 1.05 g); mp: 61.0 – 61.5°C. MS (EI, 70 eV): m/z = 215 (10), 214 (76, M^+), 137 (100), 109 (86). ^{1}H-NMR (400 MHz, $CDCl_3$): δ = 2.51 (s, 3H), 2.58 (s, 3H), 3.78 (s, 3H), 7.33 – 7.40 (m, 1H), 7.43 – 7.48 (m, 2H), 7.76 – 7.80 (m, 2H) ppm. ^{13}C-NMR (101 MHz, $CDCl_3$): δ = 10.1 (q, 1C), 14.0 (q, 1C), 36.1 (q, 1C), 121.9 & 129.0 (d, 4C), 129.4 (d, 1C), 135.3 & 138.8 & 142.6 & 153.7 (s, 4C) ppm.

3-((4-Fluorophenyl)diazenyl)-4-hydroxypent-3-en-2-one (2b):

General procedure A; using 4-fluoroaniline **1b** (822 mg, 7.40 mmol) giving yellow crystals (87%, 1.43 g); mp: 115.0 – 115.5°C. ^{1}H-NMR (400 MHz, $CDCl_3$): δ = 2.48 (s, 3H), 2.60 (s, 3H), 7.11 (dd, J = 9.0, 8.1 Hz, 2H), 7.38 (dd, J = 9.1, 4.6 Hz, 2H), 14.80 (br s, 1H) ppm. ^{13}C-NMR (101 MHz, $CDCl_3$): δ = 26.7 (q, 1C), 31.8 (q, 1C), 116.7 (d/d, $^2J_{CF}$ = 23.3 Hz, 2C), 117.8 (d/d, $^3J_{CF}$ = 8.2 Hz, 2C), 133.4 (s, 1C), 138.0 (s/d, $^4J_{CF}$ = 2.8 Hz, 1C), 160.8 (s/d, $^1J_{CF}$ = 245.9 Hz, 1C), 197.1 & 198.2 (s, 2C) ppm.

4-((4-Fluorophenyl)diazenyl)-1,3,5-trimethyl-1*H*-pyrazole (3b):

General procedure B; using azo intermediate **2b** (700 mg, 3.15 mmol) and EtOH (16 mL) giving yellow crystals (99%, 724 mg); mp: 76.0 – 76.5°C. HR-MS calcd. for $C_{12}H_{14}FN_4^+$ $[M+H]^+$: 233.1197 Da, found: 233.1210 Da, difference: 1.3 mDa. MS (EI, 70 eV): m/z = 232 (55, M^+), 137 (90), 109 (100). ^{1}H-NMR (400 MHz, $CDCl_3$): δ = 2.48 (s, 3H), 2.56 (s, 3H), 3.77 (s, 3H), 7.09 – 7.15 (m, 2H), 7.75 – 7.80 (m, 2H) ppm. ^{13}C-NMR (101 MHz, $CDCl_3$): δ = 10.1 (q, 1C), 14.0 (q, 1C), 36.1 (q, 1C), 115.8 (d/d, $^2J_{CF}$ = 22.8 Hz, 2C), 123.6 (d/d, $^3J_{CF}$ = 8.5 Hz, 2C), 135.1 & 138.9 & 142.5 (s, 1C), 150.2 (s/d, $^4J_{CF}$ = 3.1 Hz, 1C), 163.5 (s/d, $^1J_{CF}$ = 249.1 Hz, 1C) ppm.

3-((4-Chlorophenyl)diazenyl)-4-hydroxypent-3-en-2-one (2c):

General procedure A; using 4-chloroaniline **1c** (2.380 g, 18.66 mmol) and the 2.5-fold amount of solvent giving yellow crystals (95%, 4.252 g); mp: 135.0 – 135.5°C. ^{1}H-NMR (400 MHz, $CDCl_3$): δ = 2.49 (s, 3H), 2.61 (s, 3H), 7.33 – 7.40 (m, 4H), 14.70 (br s, 1H) ppm. ^{13}C-NMR (101 MHz, $CDCl_3$): δ = 26.8 (q, 1C), 31.8 (q, 1C), 117.5 (d, 2C), 129.9 (d, 2C), 131.2 & 133.6 & 140.3 (s, 3C), 197.1 & 198.3 (s, 2C) ppm.

4-((4-Chlorophenyl)diazenyl)-1,3,5-trimethyl-1*H*-pyrazole (3c):

General procedure B; using azo intermediate **2c** (1.64 g, 6.87 mmol) and EtOH (38 mL) giving yellow crystals (quant, 1.71 g); mp: 85.0 – 86.0°C. HR-MS calcd. for $C_{12}H_{14}ClN_4^+$ $[M+H]^+$: 249.0902 Da, found: 249.0912 Da, difference: 1.0 mDa. MS (EI, 70 eV): m/z = 250 (13, M^+), 248 (141, M^+), 137 (100), 109 (69). ^{1}H-NMR (400 MHz, $CDCl_3$): δ = 2.48 (s, 3H), 2.57 (s, 3H), 3.78 (s, 3H), 7.42 (d, *J* = 8.7 Hz, 2H), 7.72 (d, *J* = 8.7 Hz, 2H) ppm. ^{13}C-NMR (101 MHz, $CDCl_3$): δ = 10.1 (q, 1C), 14.0 (q, 1C), 36.2 (q, 1C), 123.1 & 129.2 (d, 4C), 135.0 & 135.2 & 139.2 & 142.7 & 152.2 (s, 5C) ppm.

3-((4-Bromophenyl)diazenyl)-4-hydroxypent-3-en-2-one (2d):

General procedure A; using 4-bromoaniline **1d** (3.210 g, 18.66 mmol) and the 2.5-fold amount of solvent giving yellow crystals (94%, 4.991 g); mp: 143.0 – 143.5°C. ^{1}H-NMR (400 MHz, $CDCl_3$): δ = 2.48 (s, 3H), 2.61 (s, 3H), 7.29 (d, *J* = 8.8 Hz, 2H), 7.52 (d, *J* = 8.8 Hz, 2H), 14.67 (br s, 1H) ppm. ^{13}C-NMR (101 MHz, $CDCl_3$): δ = 26.8 (q, 1C), 31.9 (q, 1C), 117.8 (d, 2C), 118.8 (s, 1C), 132.9 (d, 2C), 133.7 & 140.8 (s, 2C), 197.1 & 198.4 (s, 2C) ppm.

4-((4-Bromophenyl)diazenyl)-1,3,5-trimethyl-1*H*-pyrazole (3d):

General procedure B; using azo intermediate **2d** (1.945 g, 6.87 mmol) and EtOH (38 mL) giving yellow crystals (quant., 2.014 g); mp: 88.0 – 90.0°C. HR-MS calcd. for $C_{12}H_{14}BrN_4^+$ $[M+H]^+$: 293.0396 Da, found: 293.0408 Da, difference: 1.2 mDa. MS (EI, 70 eV): m/z = 294 (23, M^+), 292 (24, M^+), 137 (100), 109 (62). ^{1}H-NMR (400 MHz, $CDCl_3$): δ = 2.48 (s, 3H), 2.57 (s, 3H), 3.78 (s, 3H), 7.57 (d, *J* = 8.9 Hz, 2H), 7.66 (d, *J* = 8.8 Hz, 2H) ppm. ^{13}C-NMR (101 MHz, $CDCl_3$): δ = 10.1 (q, 1C), 14.0 (q, 1C), 36.2 (q, 1C), 123.3 (s, 1C), 123.4 & 132.2 (d, 4C), 135.3 & 139.3 & 142.7 & 152.5 (s, 4C) ppm.

4-Hydroxy-3-((4-iodophenyl)diazenyl)pent-3-en-2-one (2e):

General procedure A; using 4-iodoaniline **1e** (1.62 g, 7.40 mmol) giving yellow crystals (92%, 2.25 g); mp: 152.5 – 153.0°C. ^{1}H-NMR (400 MHz, $CDCl_3$): δ = 2.47 (s, 3H), 2.59 (s, 3H), 7.15 (d, J = 8.8 Hz, 2H), 7.69 (d, J = 8.8 Hz, 2H), 14.62 (br s, 1H) ppm. ^{13}C-NMR (101 MHz, $CDCl_3$): δ = 26.7 (q, 1C), 31.8 (q, 1C), 89.4 (s, 1C), 118.1 (d, 2C), 133.7 (s, 1C), 138.7 (d, 2C), 141.5 (s,1C), 197.0 & 198.3 (s, 2C) ppm.

4-((4-Iodophenyl)diazenyl)-1,3,5-trimethyl-1*H*-pyrazole (3e):

General procedure B; using azo intermediate **2e** (1.10 g, 3.33 mmol) and EtOH (17 mL) giving orange crystals (99%, 1.13 g); mp: 116.5 – 117.5°C. MS (EI, 70 eV): m/z = 340 (43, M^+), 137 (100), 109 (70). ^{1}H-NMR (400 MHz, $CDCl_3$): δ = 2.48 (s, 3H), 2.56 (s, 3H), 3.78 (s, 3H), 7.51 (d, J = 8.6 Hz, 2H), 7.78 (d, J = 8.7 Hz, 2H) ppm. ^{13}C-NMR (101 MHz, $CDCl_3$): δ = 10.1 (q, 1C), 14.0 (q, 1C), 36.2 (q, 1C), 95.2 (s, 1C), 123.6 (d, 2C), 135.3 (s,1C), 138.2 (d, 2C), 139.3 (s, 1C), 142.7 (s, 1C), 153.1 (s, 1C) ppm.

4-Hydroxy-3-(*p*-tolyldiazenyl)pent-3-en-2-one (2f):

General procedure A; using *p*-toluidine **1f** (2.00 g, 18.66 mmol) and the 2.5-fold amount of solvent giving yellow crystals (95%, 3.878 g); mp: 97.0 – 98.0°C. ^{1}H-NMR (400 MHz, $CDCl_3$): δ = 2.36 (s, 3H), 2.49 (s, 3H), 2.60 (s, 3H), 7.21 (d, J = 8.3 Hz, 2H), 7.31 (d, J = 8.5 Hz, 2H), 14.81 (br s, 1H) ppm. ^{13}C-NMR (101 MHz, $CDCl_3$): δ = 21.2 (q, 1C), 26.8 (q, 1C), 31.8 (q, 1C), 116.4 (d, 2C), 130.4 (d, 2C), 133.1 & 136.1 & 139.4 (s, 3C), 197.2 & 197.9 (s, 2C) ppm.

1,3,5-Trimethyl-4-(*p*-tolyldiazenyl)-1*H*-pyrazole (3f):

General procedure B; using azo intermediate **2f** (1.50 g, 6.87 mmol) and EtOH (38 mL) giving yellow crystals (quant., 1.57 g); mp: 81.0 – 83.0°C. HR-MS calcd. for $C_{13}H_{17}N_4^+$ $[M+H]^+$: 229.1448 Da, found: 229.1458 Da, difference: 1.0 mDa. MS (EI, 70 eV): m/z = 228 (67, M^+), 137 (100), 109 (81). ^{1}H-NMR (400 MHz, $CDCl_3$): δ = 2.41 (s, 3H), 2.49 (s, 3H), 2.57 (s, 3H), 3.78 (s, 3H) 7.25 (d, J = 8.0 Hz, 2H), 7.69 (d, J = 8.2 Hz, 2H) ppm. ^{13}C-NMR (101 MHz, $CDCl_3$): δ = 10.1 (q, 1C), 13.9 (q, 1C), 21.5 (q, 1C), 36.1 (q, 1C), 121.8 & 129.7 (d, 4C), 135.2 & 138.5 & 139.7 & 142.5 & 151.8 (s, 5C) ppm.

4-Hydroxy-3-((4-(trifluoromethyl)phenyl)diazenyl)pent-3-en-2-one (2g):

General procedure A; using 4-(trifluoromethyl)aniline **1g** (500 mg, 3.10 mmol) and the 0.5-fold amount of solvent giving yellow crystals (quant., 836 mg); mp: 179.0 – 180.0°C. ^{1}H-NMR (400 MHz, $CDCl_3$): δ = 2.50 (s, 3H), 2.61 (s, 3H), 7.47 (d, J = 8.4 Hz, 2H), 7.65 (d, J = 8.5 Hz, 2H), 14.58 (br s, 1H) ppm. ^{13}C-NMR (101 MHz, $CDCl_3$): δ = 26.8 (q, 1C), 31.9 (q, 1C), 116.2 (d, 2C), 124.1 (s/q, $^{1}J_{CF}$ = 271.7 Hz, 1C), 127.1 (d/q, $^{3}J_{CF}$ = 3.8 Hz, 2C), 127.5 (s/q, $^{2}J_{CF}$ = 32.9 Hz, 1C), 134.3 & 144.4 (s, 2C), 197.1 & 198.6 (s, 2C) ppm.

1,3,5-Trimethyl-4-((4-(trifluoromethyl)phenyl)diazenyl)-1*H*-pyrazole (3g):

General procedure B; using azo intermediate **2g** (425 mg, 1.56 mmol) and EtOH (8.5 mL). The crude material was purified by flash column chromatography (45 g silica gel, LP à LP/EtOAc = 1/1) giving yellow crystals (90%, 397 mg); mp: 80.0 – 81.0°C. HR-MS calcd. for $C_{13}H_{14}F_3N_4^+$ $[M+H]^+$: 283.1165 Da, found: 283.1187 Da, difference: 2.2 mDa. MS (EI, 70 eV): m/z = 282 (49, M^+), 144 (11), 137 (100), 109 (79). ^{1}H-NMR (600 MHz, $CDCl_3$): δ = 2.50 (s, 3H), 2.59 (s, 3H), 3.80 (s, 3H), 7.71 (d, J = 8.3 Hz, 2H), 7.85 (d, J = 8.1 Hz, 2H) ppm. ^{13}C-NMR (151 MHz, $CDCl_3$): δ = 10.2 (q, 1C), 14.1 (q, 1C), 36.2 (q, 1C), 122.1 (d, 2C), 124.3 (s/q, $^1J_{CF}$ = 272.2 Hz, 1C), 126.2 (d/q, $^3J_{CF}$ = 3.8 Hz, 2C), 130.7 (s/q, $^2J_{CF}$ = 32.2 Hz, 1C), 135.5 & 140.0 & 142.8 & 155.7 (s, 4C) ppm.

4-Hydroxy-3-((4-nitrophenyl)diazenyl)pent-3-en-2-one (2h):

General procedure A; using 4-nitroaniline **1h** (1.02 g, 7.40 mmol) with the temperature during the formation of the diazonium salt being held at -5°C giving yellow crystals (91% 1.67 g); mp: 224.0 – 225.5°C. ^{1}H-NMR (400 MHz, $CDCl_3$): δ = 2.53 (s, 3H), 2.64 (s, 3H), 7.49 (d, J = 9.1 Hz, 2H), 8.30 (d, J = 9.1 Hz, 2H), 14.53 (br s, 1H) ppm. ^{13}C-NMR (101 MHz, DMSO-d_6): δ = 26.1 (q, 1C), 31.2 (q, 1C), 116.0 & 125.5 (d, 4C), 137.1 & 143.0 & 148.0 (s, 3C), 196.4 & 198.1 (s, 2C) ppm.

1,3,5-Trimethyl-4-((4-nitrophenyl)diazenyl)-1*H*-pyrazole (3h):

General procedure B; using azo intermediate **2h** (900 mg, 3.61 mmol) and EtOH (19 mL) giving orange crystals (99%, 924 mg); mp: 149.0 – 151.0°C. MS (EI, 70 eV): m/z = 259 (22, M^+), 137 (100), 109 (71). ^{1}H-NMR (400 MHz, $CDCl_3$): δ = 2.50 (s, 3H), 2.61 (s, 3H), 3.81 (s, 3H), 7.87 (d, J = 9.0 Hz, 2H), 8.32 (d, J = 9.0 Hz, 2H) ppm. ^{13}C-NMR (101 MHz, $CDCl_3$): δ = 10.2 (q, CH_3), 14.2 (q, 1C), 36.3 (q,1C), 122.4 & 124.8 (d, 4C), 136.0 & 140.8 & 143.1 & 147.7 & 157.3 (s, 5C) ppm.

4-Hydroxy-3-((4-hydroxyphenyl)diazenyl)pent-3-en-2-one (2i):

General procedure A; using 4-aminophenol **1i** (808 mg, 7.40 mmol) with the temperature during the formation of the diazonium salt being held at -5°C giving brown crystals (66%, 1.07 g); mp: 240.5 – 241.0°C. ^{1}H-NMR (400 MHz, DMSO-d_6): δ = 2.37 (s, 3H), 2.45 (s, 3H), 6.83 (d, J = 8.9 Hz, 2H), 7.42 (d, J = 8.8 Hz, 2H), 9.65 (s, 1H), 14.50 (br s, 1H) ppm. ^{13}C-NMR (151 MHz, DMSO-d_6): δ = 26.4 (q, 1C), 31.1 (q, 1C), 116.1 & 118.1 (d, 4C), 132.1 & 133.7 & 155.9 (s, 3C), 195.9 & 196.0 (s, 2C) ppm.

4-((1,3,5-Trimethyl-1*H*-pyrazol-4-yl)diazenyl)phenol (3i):

General procedure B; using azo intermediate **2i** (500 mg, 2.27 mmol) and EtOH (12 mL) giving yellow crystals (quant., 523 mg); mp: 232.5 – 233.5°C. MS (EI, 70 eV): m/z = 230 (27, M^+), 137 (41), 113 (15), 109 (38). ^{1}H-NMR (400 MHz, DMSO-d_6): δ = 2.34 (s, 3H), 2.51 (s, 3H), 3.71 (s, 3H), 6.87 (d, J = 8.8 Hz, 2H), 7.61 (d, J = 8.8 Hz, 2H), 9.93 (br s, 1H) ppm. ^{13}C-NMR (101 MHz, DMSO-d_6): δ = 9.4 (q, 1C), 13.7 (q, 1C), 35.8 (q, 1C), 115.6 & 123.1 (d, 4C), 134.0 & 138.1 & 139.9 & 146.1 & 159.1 (s, 5C) ppm.

4-((2-Hydroxy-4-oxopent-2-en-3-yl)diazenyl)benzoic acid (2j):

General procedure A; using 4-aminobenzoic acid **1j** (1.02 g, 7.40 mmol) giving yellow crystals (87%, 1.60 g); mp: 268.5 – 269.0°C. ^{1}H-NMR (400 MHz, DMSO-d_6): δ = 2.43 (s, 3H), 2.47 (s, 3H), 7.63 (d, J = 8.4 Hz, 2H), 7.96 (d, J = 8.5 Hz, 2H), 13.68 (br s, 1H) ppm. ^{13}C-NMR (101 MHz, DMSO-d_6): δ = 26.3 (q, 1C), 31.2 (q, 1C), 115.7 (d, 2C), 126.9 (s, 1C), 130.9 (d, 2C), 134.8 (s, 1C), 145.4 (s, 1C), 166.8 (s, 1C), 196.4 & 197.3 (s, 2C) ppm.

4-((1,3,5-Trimethyl-1*H*-pyrazol-4-yl)diazenyl)benzoic acid (3j):

General procedure B; using azo intermediate **2j** (750 mg, 3.02 mmol) and EtOH (16 mL) giving yellow crystals (quant., 778 mg); mp: 278.0 – 279.0°C. ^{1}H-NMR (400 MHz, DMSO-d_6): δ = 2.38 (s, 3H), 2.56 (s, 3H), 3.74 (s, 3H), 7.78 (d, J = 8.5 Hz, 2H), 8.06 (d, J = 8.5 Hz, 2H), 13.06 (br s, 1H) ppm. ^{13}C-NMR (101 MHz, DMSO-d_6): δ = 9.5 (q, 1C), 13.8 (q, 1C), 36.0 (q, 1C), 121.3 & 130.4 (d, 4C), 131.0 & 134.8 & 104.5 & 140.7 & 155.5 (s, 5C), 166.9 (s, 1C) ppm.

4-((2-Hydroxy-4-oxopent-2-en-3-yl)diazenyl)benzonitrile (2k):

General procedure A; using 4-aminobenzonitril **1k** (874 mg, 7.40 mmol) giving yellow crystals (91%, 1.54 g); mp: 190.5 – 191.0°C. ^{1}H-NMR (400 MHz, $CDCl_3$): δ = 2.49 (s, 3H), 2.60 (s, 3H), 7.45 (d, J = 8.8 Hz, 2H), 7.68 (d, J = 8.8 Hz, 2H), 14.48 (br s, 1H) ppm. ^{13}C-NMR (101 MHz, $CDCl_3$): δ = 26.7 (q, 1C), 31.9 (q,1C), 108.4 (s,1C), 116.4 (d, 2C), 118.6 (s,1C), 134.0 (d, 2C), 134.7 (s,1C), 145.1 (s, 1C), 196.9 & 198.7 (s, 2C) ppm.

4-((1,3,5-Trimethyl-1*H*-pyrazol-4-yl)diazenyl)benzonitrile (3k):

General procedure B; using azo intermediate **2k** (750 mg, 3.27 mmol) and EtOH (17 mL) giving orange crystals (quant.,782 mg); mp: 141.0 – 141.5°C. HR-MS calcd. for $C_{13}H_{14}N_5^+$ [M+H]$^+$: 240.1244 Da, found: 240.1257 Da, difference: 1.3 mDa. MS (EI, 70 eV): m/z = 239 (42, M$^+$), 137 (100), 109 (77), 102 (10). ^{1}H-NMR (400 MHz, $CDCl_3$): δ = 2.48 (s, 3H), 2.58 (s, 3H), 3.79 (s, 3H), 7.73 (d, J = 8.8 Hz, 2H), 7.82 (d, J = 8.7 Hz, 2H) ppm. ^{13}C-NMR (101 MHz, $CDCl_3$): δ = 10.1 (q, 1C), 14.1 (q, 1C), 36.2 (q, 1C), 112.1 (s, 1C), 119.0 (s, 1C), 122.4 & 133.2 (d, 4C), 135.7 & 140.4 & 143.0 & 155.9 (s, 4C) ppm.

3-((4-(Diethylamino)phenyl)diazenyl)-4-hydroxypent-3-en-2-one (2l):

General procedure A; using *p*-(diethylamino)aniline **1l** (1.22 g, 7.40 mmol). The obtained crude product was purified by flash column chromatography giving red crystals (75%, 1.543 g); mp: 67.5 – 68.0°C. ^{1}H-NMR (400 MHz, $CDCl_3$): δ = 1.18 (t, J = 7.1 Hz, 6H), 2.45 (s, 3H), 2.58 (s, 3H), 3.38 (q, J = 7.1, 4H), 6.68 (d, J = 9.1 Hz, 2H), 7.31 (d, J = 9.1 Hz, 2H), 15.27 (br s, 1H) ppm. ^{13}C-NMR (101 MHz, $CDCl_3$): δ = 12.7 (q, 2C), 26.8 (q, 1C), 31.5 (q, 1C), 44.8 (t, 2C), 112.4 & 118.3 (d, 4C), 130.7 & 132.2 & 146.8 (s, 3C), 197.0 & 197.1 (s, 2C) ppm.

***N*,*N*-Diethyl-4-((1,3,5-trimethyl-1*H*-pyrazol-4-yl)diazenyl)aniline (3l):**

General procedure B; using azo intermediate **2l** (750 mg, 2.72 mmol) and EtOH (14 mL). Purification by flash column chromatography provided the pure product as orange crystals (86%, 668 mg); mp:

102.0 – 102.5°C. HR-MS calcd. for $C_{16}H_{24}N_5^+$ [M+H]$^+$: 286.2026 Da, found: 286.2040 Da, difference: 1.4 mDa. MS (EI, 70 eV): m/z = 286 (17), 285 (85, M$^+$), 271 (19), 270 (100). ^{1}H-NMR (400 MHz, $CDCl_3$): δ = 1.21 (t, J = 7.1 Hz, 6H), 2.48 (s, 3H), 2.54 (s, 3H), 3.43 (q, J = 7.1 Hz, 4H), 3.76 (s, 3H), 6.70 (d, J = 9.1 Hz, 2H), 7.72 (d, J = 9.2 Hz, 2H) ppm. ^{13}C-NMR (101 MHz, $CDCl_3$): δ = 10.1 (q, 1C), 12.8 (q, 2C), 13.8 (q, 1C), 36.0 (q, 1C), 44.8 (t, 2C), 111.3 & 123.7 (d, 4C), 135.1 & 136.7 & 142.1 & 144.1 & 149.1 (s, 5C) ppm.

3-([1,1'-Biphenyl]-4-yldiazenyl)-4-hydroxypent-3-en-2-one (2m):

General procedure A; using [1,1'-biphenyl]-4-amine **1m** (1.00 g, 5.91 mmol) and AcOH (25 mL). Ice was added to the filtrate and the precipitate was combined with the precipitate from the first step. The combined crude material was purified by flash column chromatography (90 g silica gel, LP/EtOAc = 9/1 à 1/1) giving yellow crystals (44%, 737 mg); mp: 137.5 – 140.0°C. ^{1}H-NMR (400 MHz, $CDCl_3$): δ = 2.52 (s, 3H), 2.63 (s, 3H), 7.34 – 7.39 (m, 1H), 7.43 – 7.52 (m, 4H), 7.58 – 7.62 (m, 2H), 7.67 – 7.73 (m, 2H), 14.83 (br s, 1H) ppm. ^{13}C-NMR (101 MHz, $CDCl_3$): δ = 26.8 (q, 1C), 31.8 (q, 1C), 116.8 (d, 2C), 127.0 (d, 2C), 127.7 (d, 1C), 128.5 (d, 2C), 129.1 (d, 2C), 133.5 & 139.0 & 140.2 & 140.9 (s, 4C), 197.2 & 198.1 (s, 2C) ppm.

4-([1,1'-Biphenyl]-4-yldiazenyl)-1,3,5-trimethyl-1*H*-pyrazole (3m):

General procedure B; using azo intermediate **2m** (359 mg, 1.28 mmol) and EtOH (7 mL) giving yellow crystals (97%, 361 mg); mp: 141.0 – 142.0°C. HR-MS calcd. for $C_{18}H_{19}N_4^+$ [M+H]$^+$: calculated: 291.1604 Da, found: 291.1631 Da, difference: 2.7 mDa. MS (EI, 70 eV): m/z = 290 (47, M$^+$), 152 (13), 137 (100), 109 (53). ^{1}H-NMR (400 MHz, $CDCl_3$): δ = 2.52 (s, 3H), 2.60 (s, 3H), 3.80 (s, 3H), 7.35 – 7.40 (m, 1H), 7.44 – 7.49 (m, 2H), 7.63 – 7.72 (m, 4H), 7.86 (d, J = 8.6 Hz, 2H), ppm. ^{13}C-NMR (101 MHz, $CDCl_3$): δ = 10.2 (q, 1C), 14.0 (q, 1C), 36.2 (q, 1C), 122.4 (d, 2C), 127.2 (d, 2C), 127.7 (d, 1C), 127.8 (d, 2C), 129.0 (d, 2C), 135.4 & 138.9 & 140.7 & 142.2 & 142.7 & 153.0 (s, 6C) ppm.

4-Hydroxy-3-(naphthalen-1-yldiazenyl)pent-3-en-2-one (2n):

General procedure A; using naphthalen-1-amine **1n** (2.672 g, 18.66 mmol) and the 2.5-fold amount of solvent giving a brown solid (86%, 4.076 g); mp: 144.0 – 145.0°C. ^{1}H-NMR (400 MHz, $CDCl_3$): δ = 2.57 (s, 3H), 2.69 (s, 3H), 7.53 – 7.65 (m, 3H), 7,74 (d, J = 8.2 Hz, 1H), 7.92 (d, J = 7.5 Hz, 2H), 8.04 (d, J = 8.8 Hz, 1H 15.74 (br s, 1H) ppm. ^{13}C-NMR (101 MHz, $CDCl_3$): δ = 26.9 (q, 1C), 31.9 (q, 1C), 112.2 (d, 1C), 119.7 (d, 1C), 123.6 (s, 1C), 126.2 (d, A1C), 126.2 (d, 1C), 126.6 (d, 1C), 127.2 (d, 1C), 129.0 (d, 1C), 134.2 & 134.5 & 136.7 (s, 3C), 197.4 & 198.4 (s, 2C) ppm.

1,3,5-Trimethyl-4-(naphthalen-1-yldiazenyl)-1*H*-pyrazole (3n):

General procedure B; using azo intermediate **2n** (1.747 g, 6.87 mmol) and EtOH (38 mL) giving yellow crystals (quant., 1.816 g); mp: 115.0 – 118.0°C. HR-MS calcd. for $C_{16}H_{17}N_4^+$ [M+H]$^+$: 265.1448 Da, found: 265.1457 Da, difference: 0.9 mDa. MS (EI, 70 eV): m/z = 264 (52, M$^+$), 137 (100), 127 (19), 109 (75). ^{1}H-NMR (400 MHz, $CDCl_3$): δ = 2.64 (s, 3H), 2.67 (s, 3H), 3.83 (s, 3H), 7.52 – 7.63 (m, 3H), 7.76 (dd, J = 7.6 & 1.1 Hz, 1H), 7.87 – 7.92 (m, 2H), 8.79 (d, J = 8.4 Hz, 1H) ppm. ^{13}C-NMR (101 MHz, $CDCl_3$): δ = 10.2 (q, 1C), 14.5 (q, 1C), 36.2 (q, 1C), 110.8 & 123.7 & 125.8 & 126.3 & 126.5 & 128.0 & 129.6 (d, 7C), 131.1 & 134.4 & 136.4 & 139.6 & 142.2 & 148.9 (s, 6C) ppm.

4-Hydroxy-3-(naphthalen-2-yldiazenyl)pent-3-en-2-one (2o):

General procedure A; using naphthalen-2-amine **1o** (2.672 g, 18.66 mmol) and the 2.5-fold amount of solvent giving a brown solid (90%, 4.270 g); mp: 131.0 – 133.0°C. ^{1}H-NMR (400 MHz, $CDCl_3$): *δ* = 2.56 (s, 3H), 2.64 (s, 3H), 7.46 (ddd, *J* = 8.0, 6.9, 1.3 Hz, 1H), 7.52 (ddd, *J* = 8.4, 6.8, 1.3 Hz, 1H), 7.67 – 7.73 (m, 2H), 7.83 (d, *J* = 8.9 Hz, 2H), 7.90 (d, *J* = 8.8 Hz, 2H), 14.98 (br s, 1H) ppm. ^{13}C-NMR (101 MHz, $CDCl_3$): *δ* = 26.9 (q, 1C), 31.8 (q, 1C), 113.7 (d, 1C), 115.7 (d, 1C), 125.9 (d, 1C), 127.4 (d, 1C), 127.8 (d, 1C), 128.2 (d, 1C), 130.2 (d, 1C), 131.9 & 133.6 & 133.8 & 139.3 (s, 4C), 197.2 & 198.1 (s, 2C) ppm.

1,3,5-Trimethyl-4-(naphthalen-2-yldiazenyl)-1*H*-pyrazole (3o):

General procedure B; using azo intermediate **2o** (1.747 g, 6.87 mmol) and EtOH (38 mL) giving yellow crystals (quant., 1.816 g); mp: 120.0 – 121.0°C. HR-MS calcd. for $C_{16}H_{17}N_4^+$ $[M+H]^+$: 265.1448 Da, found: 265.1457 Da, difference: 0.9 mDa. MS (EI, 70 eV): m/z = 264 (54, M^+), 236 (10), 235 (11), 137 (100), 127 (19), 109 (76) ^{1}H-NMR (400 MHz, $CDCl_3$): *δ* = 2.56 (s, 3H), 2.63 (s, 3H), 3.80 (s, 3H), 7.48 – 7.55 (m, 2H), 7.84 – 7.89 (m, 2H), 7.94 – 8.01 (m, 2H), 8.26 (d, *J* = 1.5 Hz, 1H) ppm. ^{13}C-NMR (101 MHz, $CDCl_3$): *δ* = 10.2 (q, 1C), 14.1 (q, 1C), 36.2 (q, 1C), 117.2 & 125.4 & 126.6 & 126.8 & 128.0 & 129.0 & 129.1 (d, 7C), 133.9 & 134.2 & 135.4 & 138.9 & 142.7 & 151.4 (s, 6C) ppm.

4-Ethynylaniline (1p):

4-Ethynylaniline **1p** was prepared *via* Sonogashira coupling.[2] An oven-dried round bottom flask equipped with a magnetic stirring bar was charged with 4-iodoaniline **1e** (9.35 g, 42.7 mmol, 1 equiv.), bis(triphenylphosphine)palladium(II) dichloride (599 mg, 0.85 mmol, 2 mol%), and copper(I) iodide (163 mg, 0.85 mmol, 2 mol%). The flask was closed with a septum, evacuated, and flushed with argon three times. Triethylamine (35 mL) and dry THF (71 mL) were added *via* syringe. The suspension was stirred for 5 minutes at room temperature and then trimethylsilylacetylene (5.59 g, 56.9 mmol, 1.33 equiv.) was added *via* syringe and the reaction mixture was stirred for 16 hours. TLC and GC-MS analysis showed full conversion. Volatiles were removed *in vacuo*. The residue was dissolved in DCM and the solution was filtered through a short pad of silica gel using DCM as eluent. The solvent was removed *in vacuo* providing the TMS-protected product. The material was dissolved in MeOH (71 mL). K_2CO_3 (5.89 g, 42.7 mmol, 1 equiv.) was added and the reaction was stirred at room temperature for 16 hours. K_2CO_3 was removed by filtration. MeOH was used for washing and the filtrate was concentrated *in vacuo*. The residue was dissolved in H_2O (100 mL) and DCM (100 mL) and phases were separated. The aqu. phase was extracted with DCM (3 x 50 mL). The combined organic phases were washed with brine (150 mL) and dried over $MgSO_4$. After evaporation of the solvent *in vacuo* a brown solid (78%, 3.91 g) was obtained; mp: 101.0 – 102.0°C. ^{1}H-NMR (400 MHz, DMSO-d_6): *δ* = 3.75 (s, 1H), 5.50 (br s, 2H), 6.52 (d, *J* = 8.6 Hz, 2H), 7.12 (d, *J* = 8.5 Hz, 2H,) ppm. ^{13}C-NMR (101 MHz, DMSO-d_6): *δ* = 77.1 & 85.1 (s & d, 2C), 107.7 (s, 1C), 113.5 (d, 2C), 132.8 (d, 2C), 149.5 (s, 1C) ppm.

3-((4-Ethynylphenyl)diazenyl)-4-hydroxypent-3-en-2-one (2p):

General procedure A; using 4-ethynylaniline **1p** (443 mg, 3.70 mmol) and the 0.5-fold amount of solvent. The product was obtained after purification by flash column chromatography as yellow crystals (34%, 285 mg), 52% based on combined yield with side product **2q**; mp: 190.0 – 191.0°C. ^{1}H-NMR (400 MHz, $CDCl_3$): *δ* = 2.49 (s, 3H), 2.60 (s, 3H), 3.12 (s, 1H), 7.35 (d, *J* = 8.7 Hz, 2H), 7.52

(d, J = 8.7 Hz, 2H), 14.65 (br s, 1H) ppm. ^{13}C-NMR (101 MHz, $CDCl_3$): δ = 26.8 (q, 1C), 31.8 (q, 1C), 78.1 & 83.2 (s & d, 2C), 116.1 (d, 2C), 119.4 (s, 1C), 133.7 (d, 2C), 133.7 (s, 1C), 141.8 (s, 1C), 197.1 & 198.3 (s, 2C) ppm.

4-((4-Ethynylphenyl)diazenyl)-1,3,5-trimethyl-1*H*-pyrazole (3p):

General procedure B; using azo intermediate **2p** (100 mg, 0.44 mmol) and EtOH (2.3 mL). Purification by flash column chromatography provided yellow crystals (71%, 74 mg); mp: 102.5 – 103.5°C. HR-MS calcd. for $C_{14}H_{15}N_4^+$ [M+H]$^+$: 239.1291 Da, found: 239.1309 Da, difference: 1.8 mDa. MS (EI, 70 eV): m/z = 238 (49, M$^+$), 137 (100), 109 (71), 101 (15). ^{1}H-NMR (400 MHz, $CDCl_3$): δ = 2.49 (s, 3H), 2.58 (s, 3H), 3.17 (s, 1H), 3.78 (s, 3H), 7.57 (d, J = 8.7 Hz, 2H), 7.74 (d, J = 8.6 Hz, 2H) ppm. ^{13}C-NMR (101 MHz, $CDCl_3$): δ = 10.1 (q, 1C), 14.0 (q, 1C), 36.2 (q, 1C), 78.7 & 83.8 (s & d, 2C), 121.9 (d, 2C), 122.9 (s, 1C), 133.0 (d, 2C), 135.5 (s,1C), 139.4 (s, 1C), 142.7 (s, 1C), 153.5 (s, 1C) ppm.

3-((4-Acetylphenyl)diazenyl)-4-hydroxypent-3-en-2-one (2q):

Compound **2q** was obtained as a side product in the preparation of alkyne **2p** due to partial hydration of the triple bond. Orange crystals (18%, 285 mg, 52% based on combined yield with main product **2p**) were isolated through separation *via* flash column chromatography; mp: 143.0 – 143.5°C. ^{1}H-NMR (400 MHz, $CDCl_3$): δ = 2.49 (s, 3H), 2.57 (s, 3H), 2.59 (s, 3H), 7.43 (d, J = 8.8 Hz, 2H), 7.99 (d, J = 8.7 Hz, 2H), 14.54 (br s, 1H) ppm. ^{13}C-NMR (101 MHz, $CDCl_3$): δ = 26.6 (q, 1C), 26.7 (q, 1C), 31.8 (q, 1C), 115.8 & 130.4 (d, 4C,), 134.2 & 134.4 & 145.3 (s, 3C), 196.6 & 197.0 & 198.4 (s, 3C) ppm.

1-(4-((1,3,5-Trimethyl-1*H*-pyrazol-4-yl)diazenyl)phenyl)ethan-1-one (3q):

General procedure B; using azo intermediate **2q** (50 mg, 0.20 mmol) and EtOH (1.0 mL). Purification by flash column chromatography provided orange crystals (77%, 39 mg); mp: 111.5 – 112.5°C. HR-MS calcd. for $C_{14}H_{17}N_4O^+$ [M+H]$^+$: 257.1397 Da, found: 257.1406 Da, difference: 0.9 mDa. MS (EI, 70 eV): m/z = 256 (46, M$^+$), 137 (100), 109 (76). ^{1}H-NMR (400 MHz, $CDCl_3$): δ = 2.50 (s, 3H), 2.60 (s, 3H), 2.64 (s, 3H), 3.79 (s, 3H), 7.82 (d, J = 8.6 Hz, 2H), 8.05 (d, J = 8.7 Hz, 2H) ppm. ^{13}C-NMR (101 MHz, $CDCl_3$): δ = 10.2 (q, 1C), 14.1 (q, 1C), 26.9 (q, 1C), 36.2 (q, 1C), 121.9 & 129.5 (d, 4C), 135.8 & 137.2 & 139.9 & 142.9 & 156.5 (s, 5C), 197.7 (s, 1C) ppm.

4-((1,3,5-Trimethyl-1*H*-pyrazol-4-yl)diazenyl)aniline (3r):

Compound **3r** was prepared by applying literature conditions[3] for similar substrates. Na_2S (1.25 g, 5.21 mmol, 3 equiv.) was added to a solution of compound **3h** (451 mg, 1.74 mmol, 1 equiv.) in THF/H_2O (3/1, 22.8 mL). The suspension was stirred at reflux temperature for 3 hours. THF was removed *in vacuo* and the residue was partitioned between EtOAc (28.5 mL) and 1 N NaOH (9.5 mL). The organic phase was washed with 1 N NaOH, satd. aqu. $NaHCO_3$ (10 mL) and brine (10 mL) and was dried over $MgSO_4$ and concentrated *in vacuo*, affording the crude product. After purification by flash column chromatography yellow crystals (87%, 347 mg) were obtained; mp: 198.0 – 201.0°C. HR-MS calcd. for $C_{12}H_{16}N_5^+$ [M+H]$^+$: 230.1400 Da, found: 230.1425 Da, difference: 2.5 mDa. MS (EI, 70 eV): m/z = 230 (15), 229 (100, M$^+$), 137 (79), 109 (78). ^{1}H-NMR (400 MHz, $CDCl_3$): δ = 2.48 (s, 3H), 2.54 (s, 3H), 3.76 (s, 3H), 3.90 (br s, 2H), 6.72 (d, J = 8.7 Hz, 2H), 7.66

(d, J = 8.7 Hz, 2H) ppm. ^{13}C-NMR (101 MHz, $CDCl_3$): δ = 10.1 (q, 1C), 13.8 (q, 1C), 36.0 (q, 1C), 114.9 & 123.6 (d, 4C), 135.0 & 137.4 & 142.2 & 146.7 & 148.1 (s, 5C) ppm.

4-((4-Methoxyphenyl)diazenyl)-1,3,5-trimethyl-1*H*-pyrazole (3s):

Compound **3i** (100 mg, 0.43 mmol, 1 equiv.) was added to a stirred suspension of K_2CO_3 (120 mg, 0.87 mmol, 2 equiv.) and Cs_2CO_3 (71 mg, 0.21 mmol, 0.5 equiv.) in DMF (2.2 mL). After stirring at room temperature for 10 minutes methyl iodide (123 mg, 0.87 mmol, 2 equiv.) was added to the reaction. After stirring at room temperature for 16 hours, complete conversion was observed by GC-MS and TLC analysis. H_2O (10 mL) was added and the mixture was extracted with EtOAc (3 x 10 mL). The combined organic phases were washed with H_2O (2 x 10 mL) and dried over $MgSO_4$. The solvent was removed *in vacuo* and the crude product was purified by flash column chromatography yielding yellow crystals (78%, 83 mg); mp: 104.0 – 104.5°C. HR-MS calcd. for $C_{13}H_{17}N_4O$ $[M+H]^+$: 245.1397 Da, found: 245.1426 Da, difference: 2.9 mDa. MS (EI, 70 eV): m/z = 245 (15), 244 (100, M^+), 137 (100), 122 (11), 109 (65). ^{1}H-NMR (400 MHz, $CDCl_3$): δ = 2.49 (s, 3H), 2.56 (s, 3H), 3.77 (s, 3H), 3.87 (s, 3H), 6.97 (d, J = 9.0 Hz, 2H), 7.77 (d, J = 9.0 Hz, 2H) ppm. ^{13}C-NMR (101 MHz, $CDCl_3$): δ = 10.1 (q, 1C), 13.9 (q, 1C), 36.1 (q, 1C), 55.7 (q, 1C), 114.2 & 123.4 (d, 4C), 135.0 & 138.1 & 142.3 & 148.1 & 160.9 (s, 5C) ppm.

4-((1,3,5-Trimethyl-1*H*-pyrazol-4-yl)diazenyl)benzamide (3t):

Compound **3k** (46.5 mg, 0.19 mmol, 1 equiv.) was dissolved in conc. H_2SO_4 (0.5 mL) and stirred at 50°C for 4 hours after which time it was slowly poured into ice/H_2O and basified using 6 N NaOH followed by satd. aqu. $NaHCO_3$. The aqu. mixture was extracted with EtOAc (3 x 5 mL), dried over $MgSO_4$ and concentrated *in vacuo* to provide orange crystals (68%, 34 mg); mp: 198.0 – 201.0°C. HR-MS calcd. for $C_{13}H_{16}N_5O^+$ $[M+H]^+$: 258.1350 Da, found: 258.1357 Da, difference: 0.7 mDa. MS (EI, 70 eV): m/z = 257 (46, M^+), 208 (10), 207 (56), 137 (100), 109 (76). ^{1}H-NMR (400 MHz, $CDCl_3$): δ = 2.50 (s, 3H), 2.60 (s, 3H), 3.80 (s, 3H), 5.63 (br s, 1H) 6.09 (br s, 1H) 7.83 (d, J = 8.7 Hz, 2H), 7.91 (d, J = 8.7 Hz, 2H) ppm. ^{13}C-NMR (101 MHz, $CDCl_3$): δ = 10.2 (q, 1C), 14.1 (q, 1C), 36.2 (q, 1C), 122.0 & 128.4 (d, 4C), 133.4 & 135.6 & 139.8 & 142.9 & 156.0 (s, 5C), 168.8 (s, 1C) ppm.

Ethyl 4-((1,3,5-trimethyl-1*H*-pyrazol-4-yl)diazenyl)benzoate (3u):

Acid **3j** (300 mg, 1.16 mmol) was placed in an 8 mL vial. EtOH (5 mL) and conc. H_2SO_4 (3 drops) was added and the suspension was stirred for 16 hours at 60°C in a thermo block (thermo sensor was set to 66°C). The reaction was extracted with diethyl ether (2 x 20 mL). The combined organic phases were washed with 1 N NaOH (20 mL), satd. aqu. $NaHCO_3$ (2 x 15 mL) and brine (2 x 15 mL). After evaporation of the solvent *in vacuo* orange crystals (92%, 305 mg) were obtained; mp: 132.0 – 133.0°C. HR-MS calcd. for $C_{15}H_{19}N_4O_2^+$ $[M+H]^+$: 287.1503 Da, found: 287.1509 Da, difference: 0.6 mDa. MS (EI, 70 eV): m/z = 286 (29, M^+), 137 (100), 109 (58). ^{1}H-NMR (400 MHz, $CDCl_3$): δ = 1.42 (t, J = 7.1 Hz, 3H), 2.51 (s, 3H), 2.60 (s, 3H), 3.80 (s, 3H), 4.40 (q, J = 7.1 Hz, 2H), 7.80 (d, J = 8.8 Hz, 2H), 8.13 (d, J = 8.8 Hz, 2H) ppm. ^{13}C-NMR (101 MHz, $CDCl_3$): δ = 10.2 (q, 1C), 14.1 (q,1C), 14.5 (q, 1C), 36.2 (q,1C), 61.2 (t,1C), 121.7 & 130.6 (d, 4C), 130.7 & 135.7 & 139.8 & 142.9 & 156.5 (s, 5C), 166.5 (s, 1C) ppm.

***N*-Phenyl-4-((1,3,5-trimethyl-1*H*-pyrazol-4-yl)diazenyl)benzamide (3v):**

Acid **3j** (38.7 mg, 0.150 mmol, 1 equiv.), EDCI•HCl (31.4 mg, 0.165 mmol, 1.1 equiv.), HOBt (25.2 mg, 0.165 mmol, 1.1 equiv.) and DIPEA (40.7 mg, 0.315 mmol, 2.1 equiv.) were dissolved in dry DMF (1.5 mL) and were stirred under argon at room temperature for 30 minutes. Then a solution of aniline **1a** (14.0 mg, 0.150 mmol, 1 equiv.) in DMF (1.5 mL) was added *via* syringe and the reaction was stirred at room temperature for 16 hours. Volatiles were removed *in vacuo*. Purification by flash column chromatography.afforded yellow crystals (87%, 43.5 mg); mp: 168.0 – 170.0°C. HR-MS calcd. for $C_{19}H_{20}N_5O^+$ $[M+H]^+$: 334.1663 Da, found: 334.1687 Da, difference: 2.4 mDa. ^{1}H-NMR (400 MHz, $CDCl_3$): δ = 2.51 (s, 3H), 2.61 (s, 3H), 3.80 (s, 3H), 7.17 (t, J = 7.4 Hz, 1H), 7.39 (t, J = 7.9 Hz, 1H), 7.67 (d, J = 7.7 Hz, 2H), 7.84 – 7.89 (m, 3H), 7.97 (d, J = 8.5 Hz, 2H) ppm. ^{13}C-NMR (101 MHz, $CDCl_3$): δ = 10.2 (q, 1C), 14.1 (q, 1C), 36.2 (q, 1C), 120.3 & 122.2 & 128.0 & 129.3 (d, 8C), 124.8 (d, 1C), 135.1 & 135.6 & 138.1 & 139.8 & 142.9 & 155.9 (s, 6C), 165.3 (s, 1C) ppm.

***N*-Benzyl-4-((1,3,5-trimethyl-1*H*-pyrazol-4-yl)diazenyl)benzamide (3w):**

Acid **3j** (37.2 mg, 0.144 mmol, 1 equiv.), EDCI•HCl (30.3 mg, 0.158 mmol, 1.1 equiv.), HOBt (24.2 mg, 0.158 mmol, 1.1 equiv.) and DIPEA (39.1 mg, 0.302 mmol, 2.1 equiv.) were dissolved in dry DMF (1.5 mL) and were stirred under argon at room temperature for 30 minutes. Then a solution of benzylamine (15.4 mg, 0.144 mmol, 1 equiv.) in DMF (1.5 mL) was added *via* syringe and the reaction was stirred at room temperature for 16 hours. Volatiles were removed *in vacuo*. Purification by flash column chromatography afforded yellow crystals (79%, 39.5 mg); mp: 177.0 – 178.0°C. HR-MS calcd. for $C_{20}H_{22}N_5O^+$ $[M+H]^+$: 348.1819 Da, found: 348.1843 Da, difference: 2.4 mDa. ^{1}H-NMR (400 MHz, $CDCl_3$): δ = 2.50 (s, 3H), 2.59 (s, 3H), 3.79 (s, 3H), 4.68 (d, J = 5.6 Hz, 2H), 6.43 (br t, J = 4.9 Hz, 1H), 7.28 – 7.40 (m, 5H), 7.81 (d, J = 8.7 Hz, 2H), 7.89 (d, J = 8.6 Hz, 2H) ppm. ^{13}C-NMR (101 MHz, $CDCl_3$): δ = 10.2 (q, 1C), 14.1 (q, 1C), 36.2 (q, 1C), 44.4 (t, 1C), 122.0 & 128.0 & 128.1 & 129.0 (d, 8C), 127.8 (d,1C), 134.5 & 135.6 & 138.3 & 139.7 & 142.8 & 155.7 (s, 6C), 167.0 (s, 1C) ppm.

Table S1. Yields of synthetic steps towards substituted arylazo-1,3,5-trimethylpyrazoles **3a-w**. quant. = quantitative yield

compound	substituent	yield / %	compound	yield / %
**2a**	H	73	**3a**	quant.
**2b**	F	87	**3b**	99
**2c**	Cl	95	**3c**	quant.
**2d**	Br	94	**3d**	quant.
**2e**	I	92	**3e**	quant.
**2f**	Me	95	**3f**	quant.
**2g**	CF_3	quant.	**3g**	90
**2h**	NO_2	91	**3h**	99
**2i**	OH	66	**3i**	quant.
**2j**	COOH	87	**3j**	quant.
**2k**	C≡N	91	**3k**	quant.
**2l**	NEt_2	75	**3l**	86
**2m**	Ph	44	**3m**	97
**2n**	1-naphthyl	86	**3n**	quant.
**2o**	2-naphthyl	90	**3o**	quant.
**2p**	C≡CH	34	**3p**	71
**2q**	$COCH_3$	18	**3q**	77
	NH_2		**3r**	87
	OCH_3		**3s**	78
	$CONH_2$		**3t**	68
	COOEt		**3u**	92
	CONHPh		**3v**	87
	CONHBn		**3w**	79

SECTION S2: ^{1}H AND ^{13}C SPECTRA

4-Hydroxy-3-(phenyldiazenyl)pent-3-en-2-one (2a)

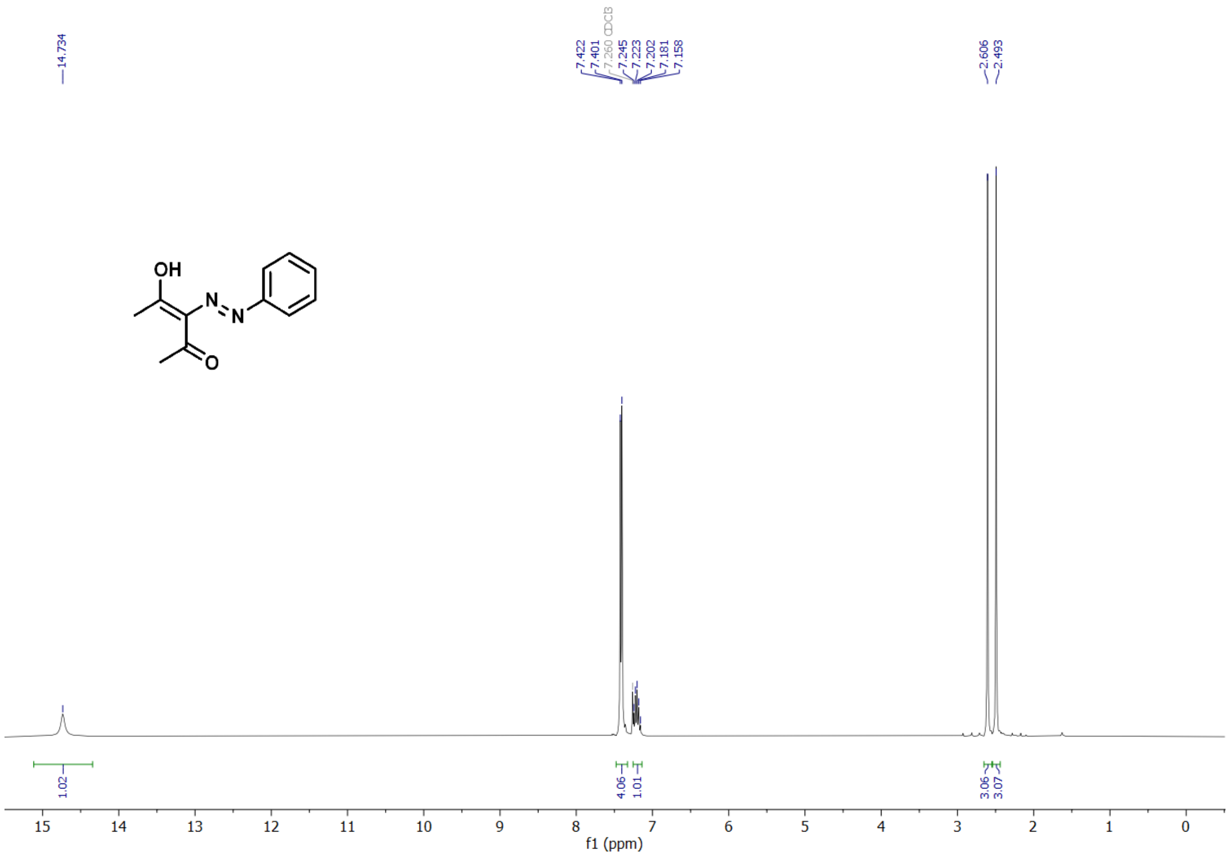

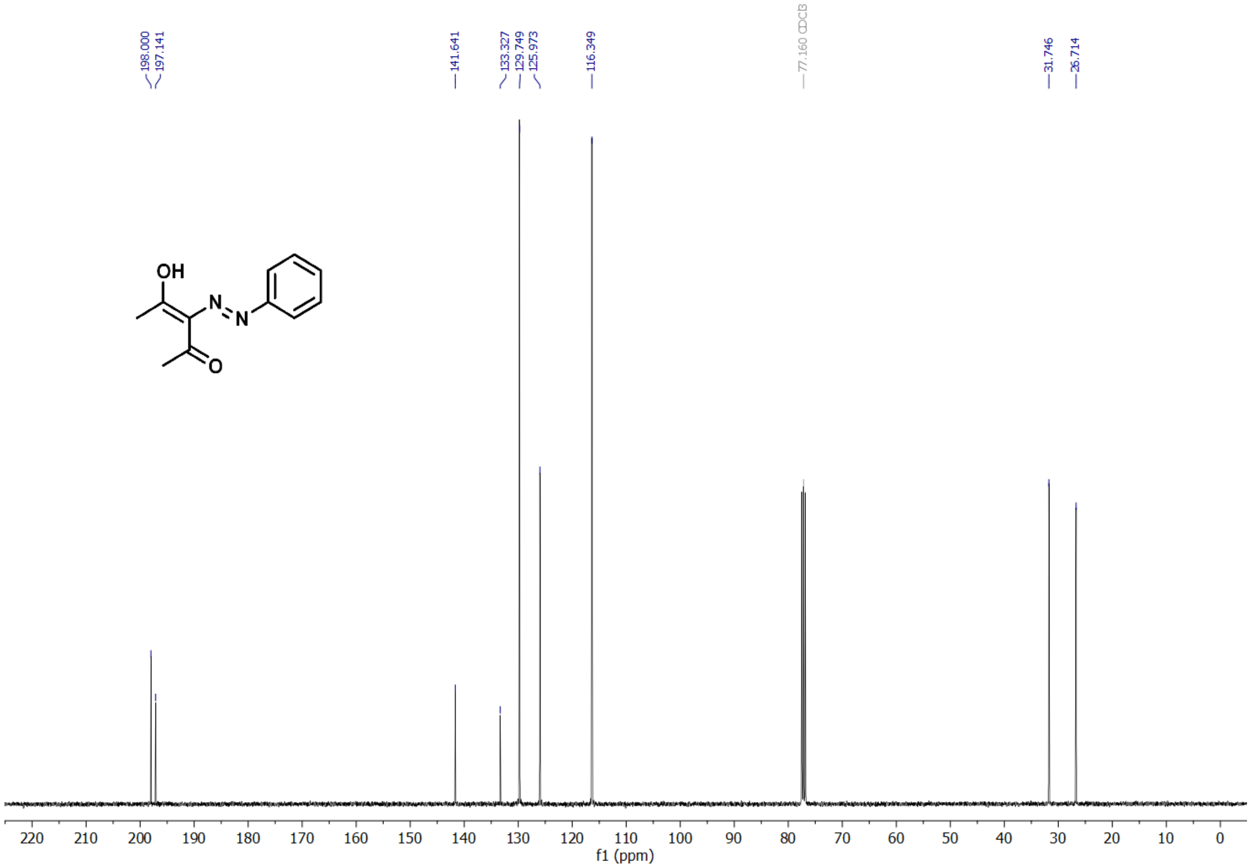

1,3,5-Trimethyl-4-(phenyldiazenyl)-1H-pyrazole (3a)

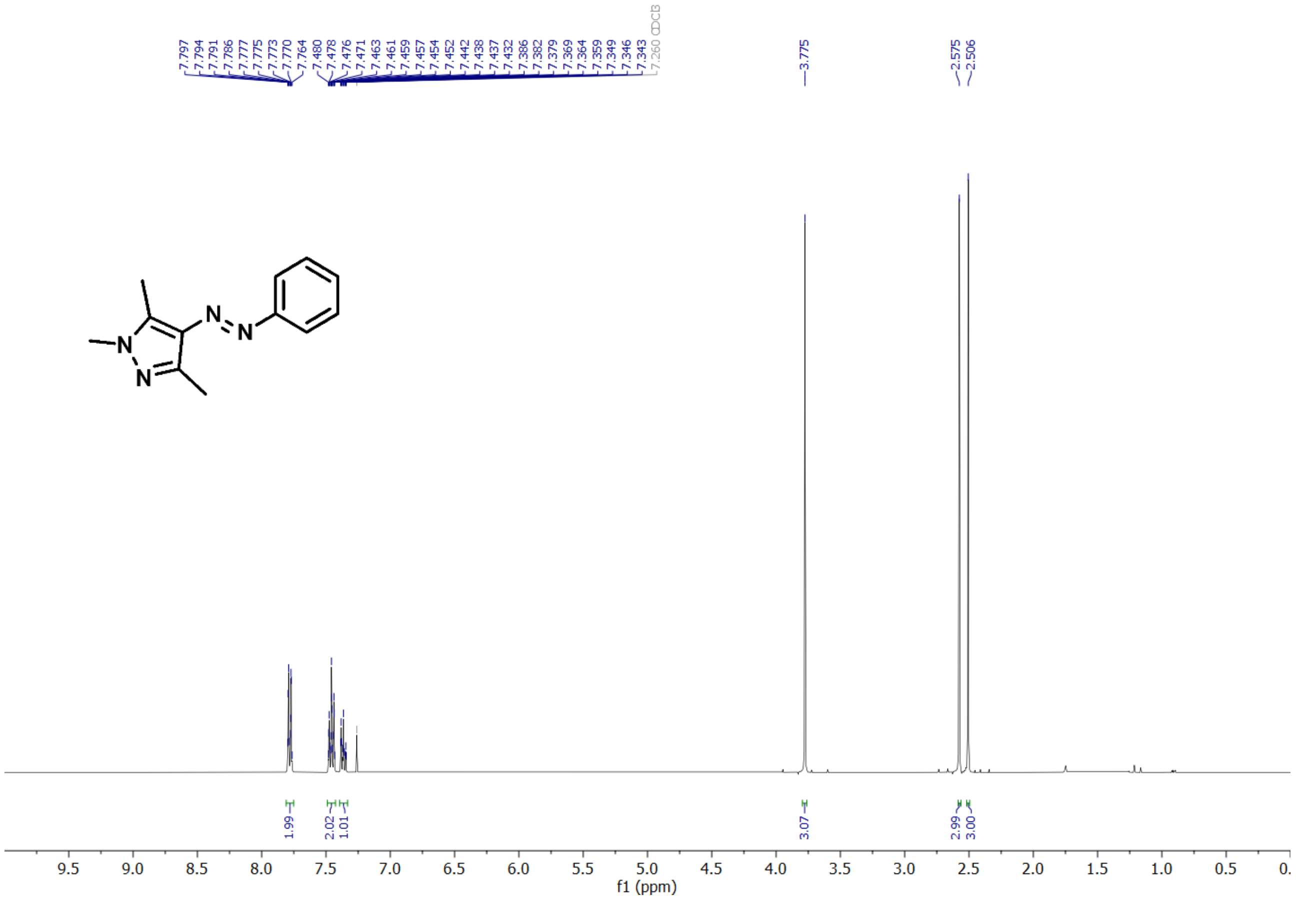

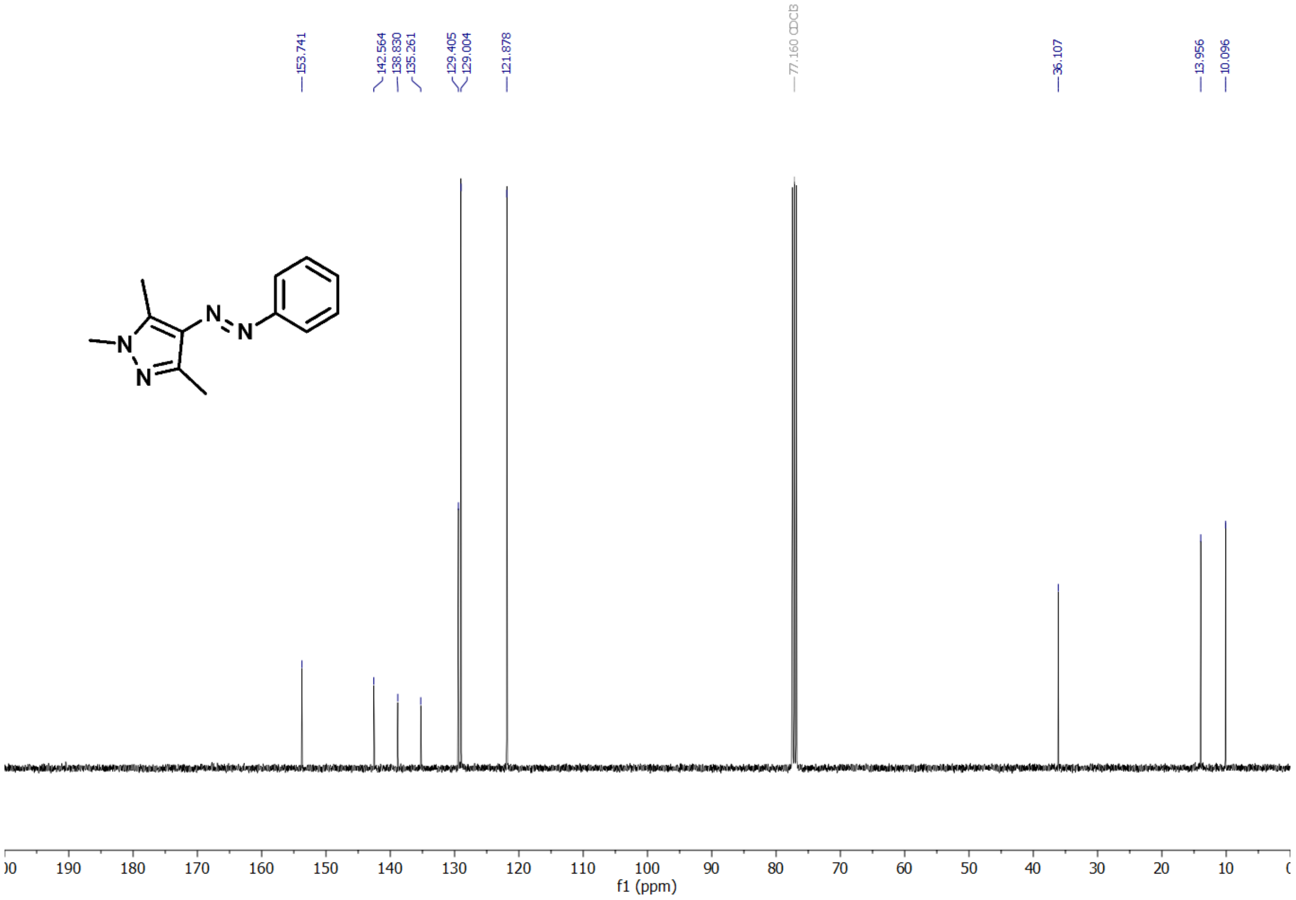

3-((4-Fluorophenyl)diazenyl)-4-hydroxypent-3-en-2-one (2b)

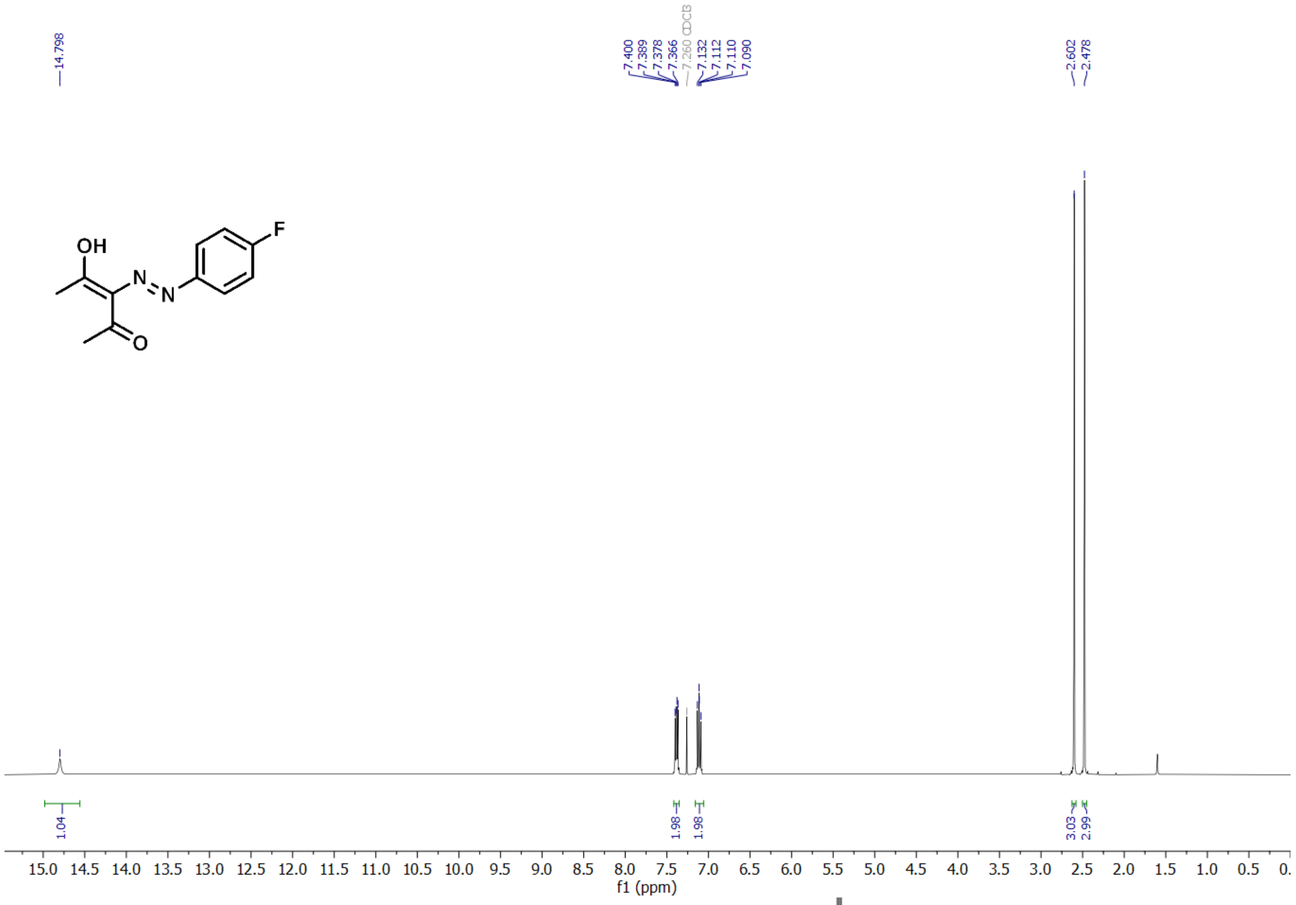

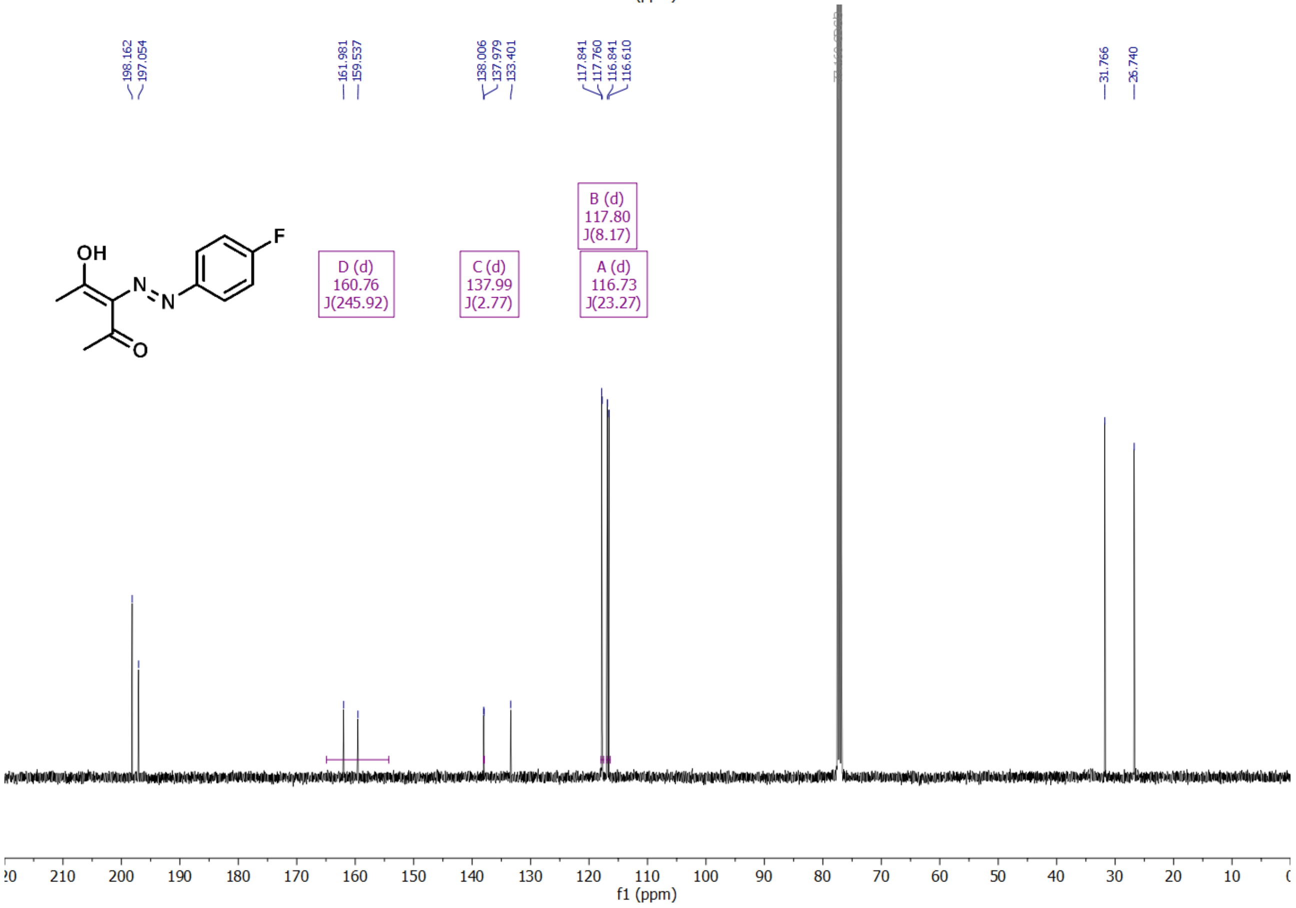

4-((4-Fluorophenyl)diazenyl)-1,3,5-trimethyl-1H-pyrazole (3b)

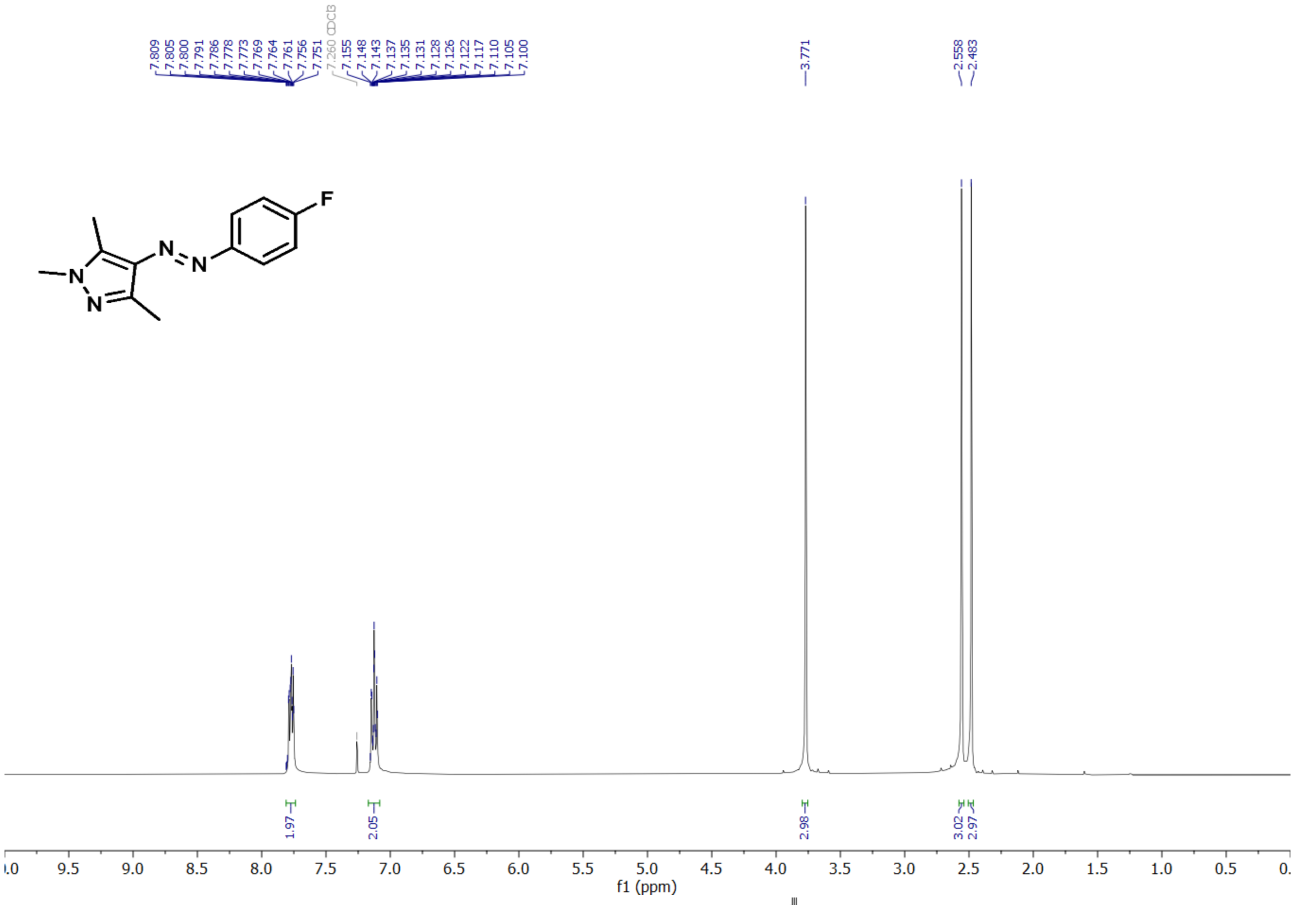

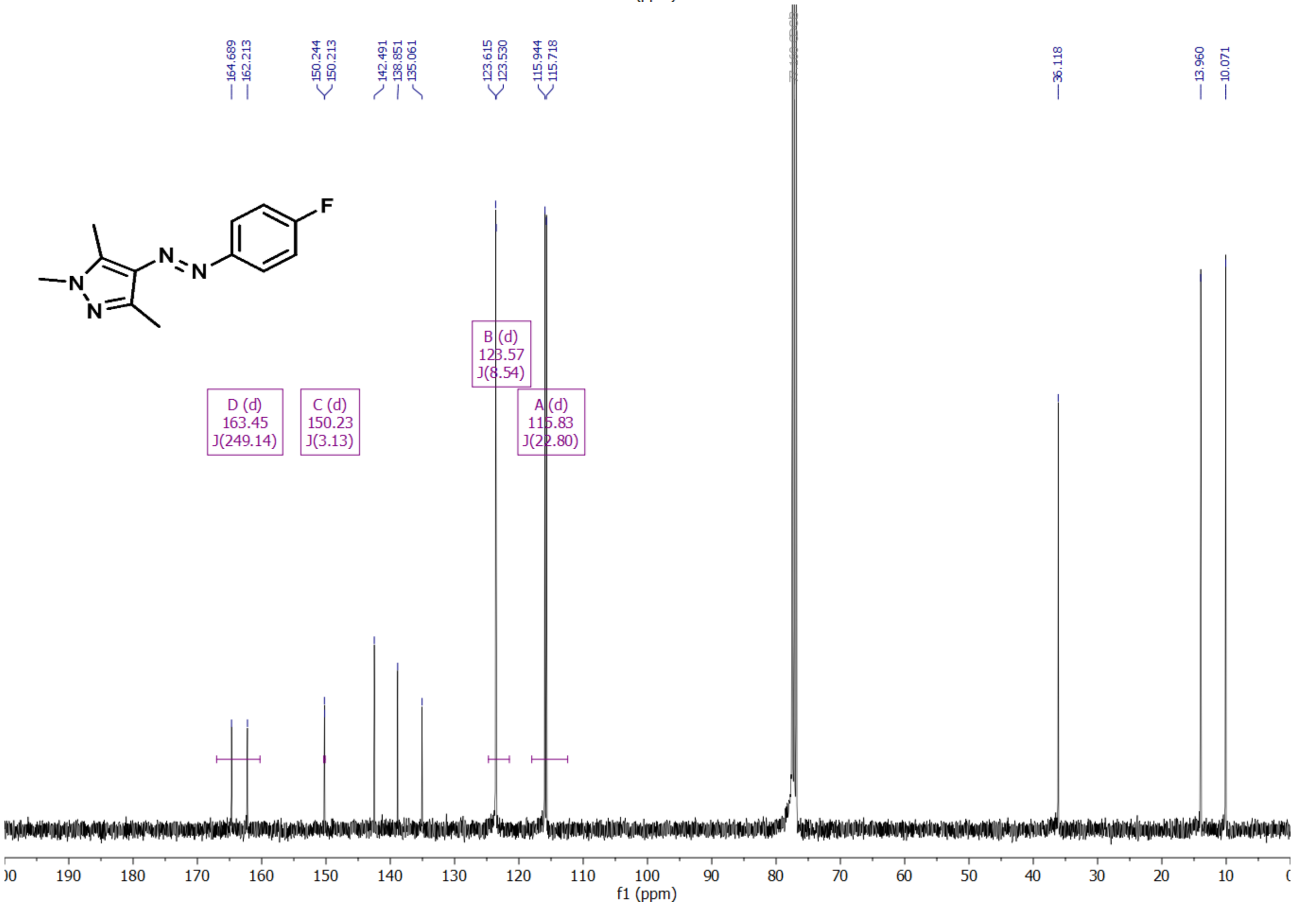

3-((4-Chlorophenyl)diazenyl)-4-hydroxypent-3-en-2-one (2c)

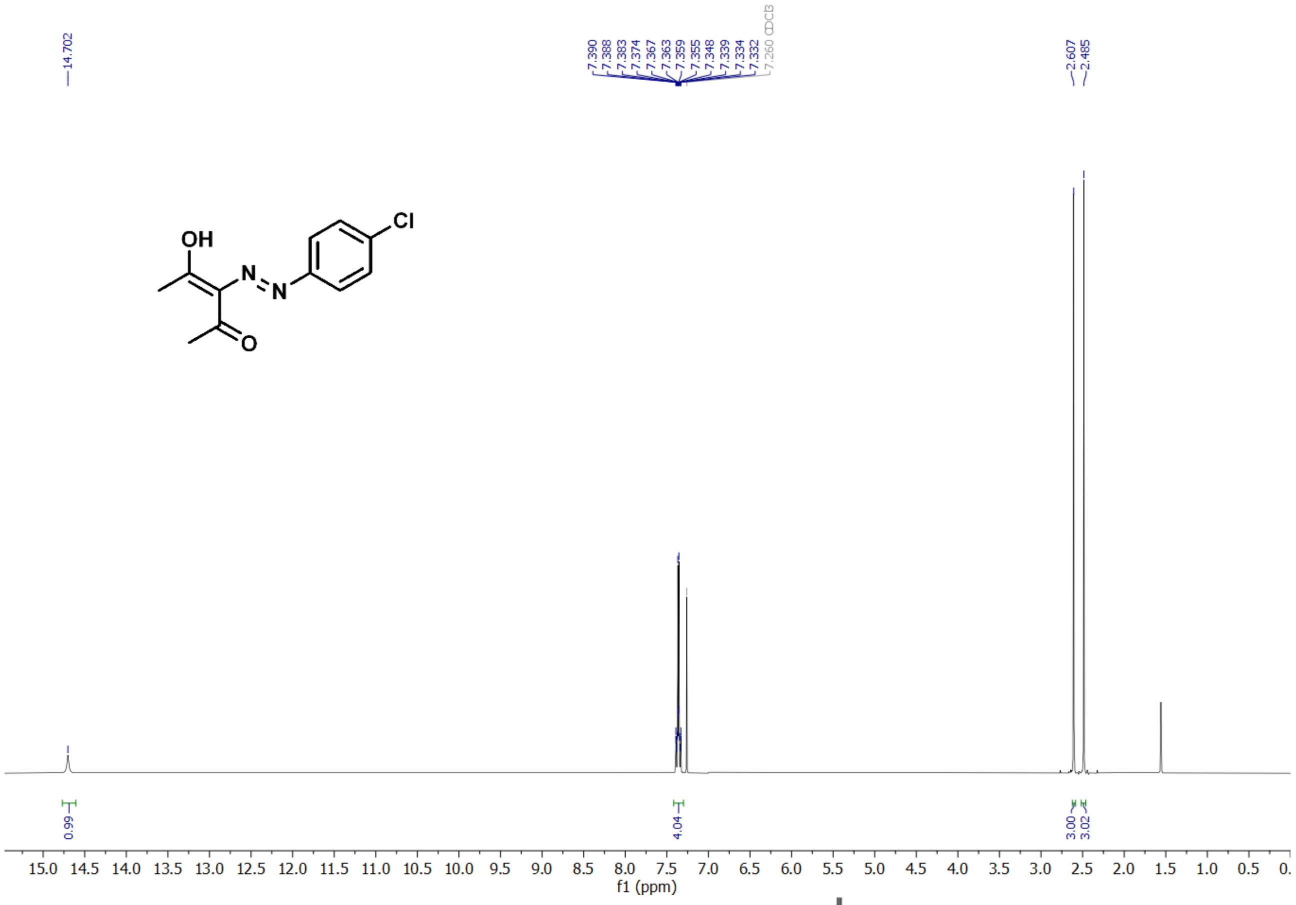

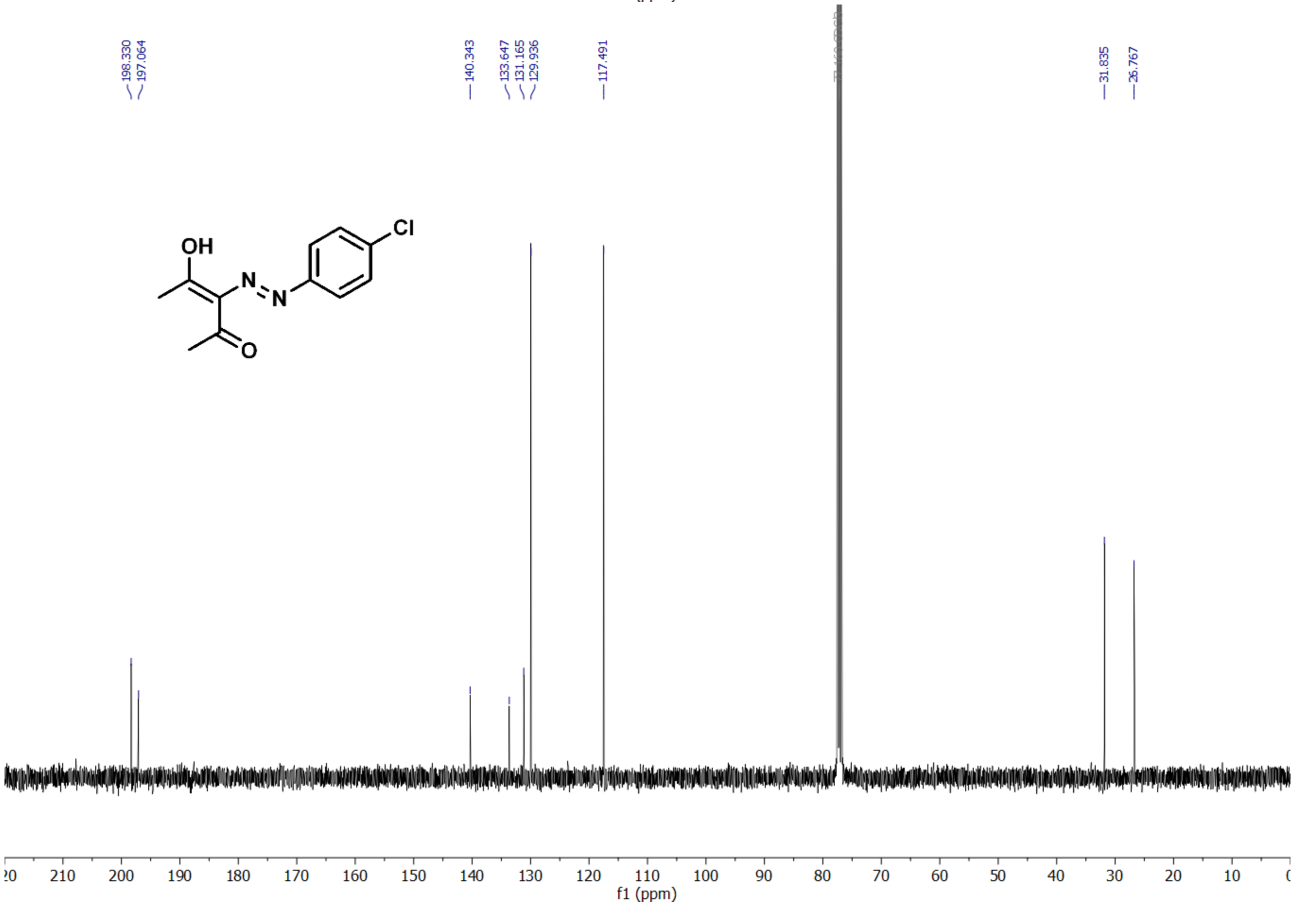

4-((4-Chlorophenyl)diazenyl)-1,3,5-trimethyl-1H-pyrazole (3c)

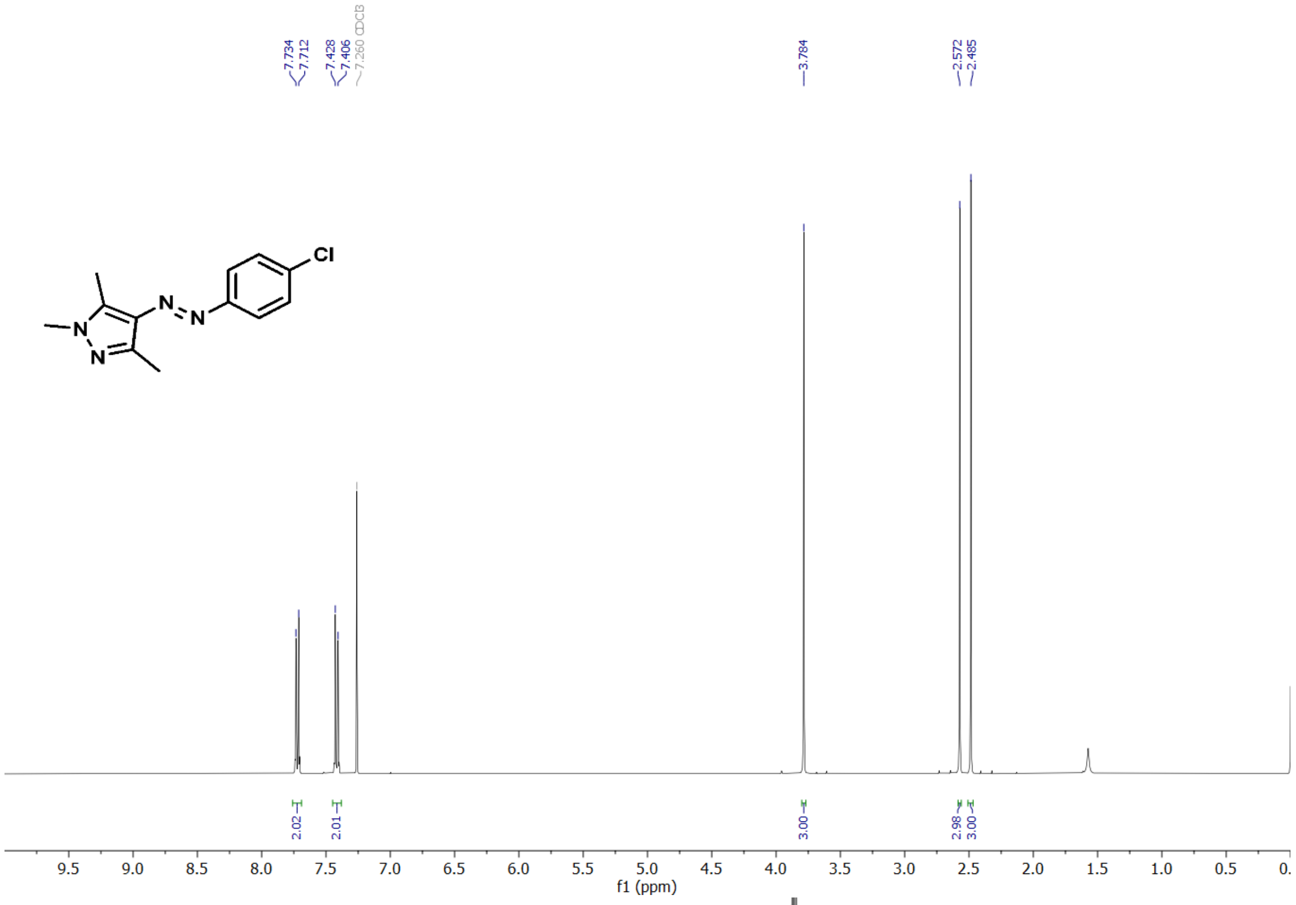

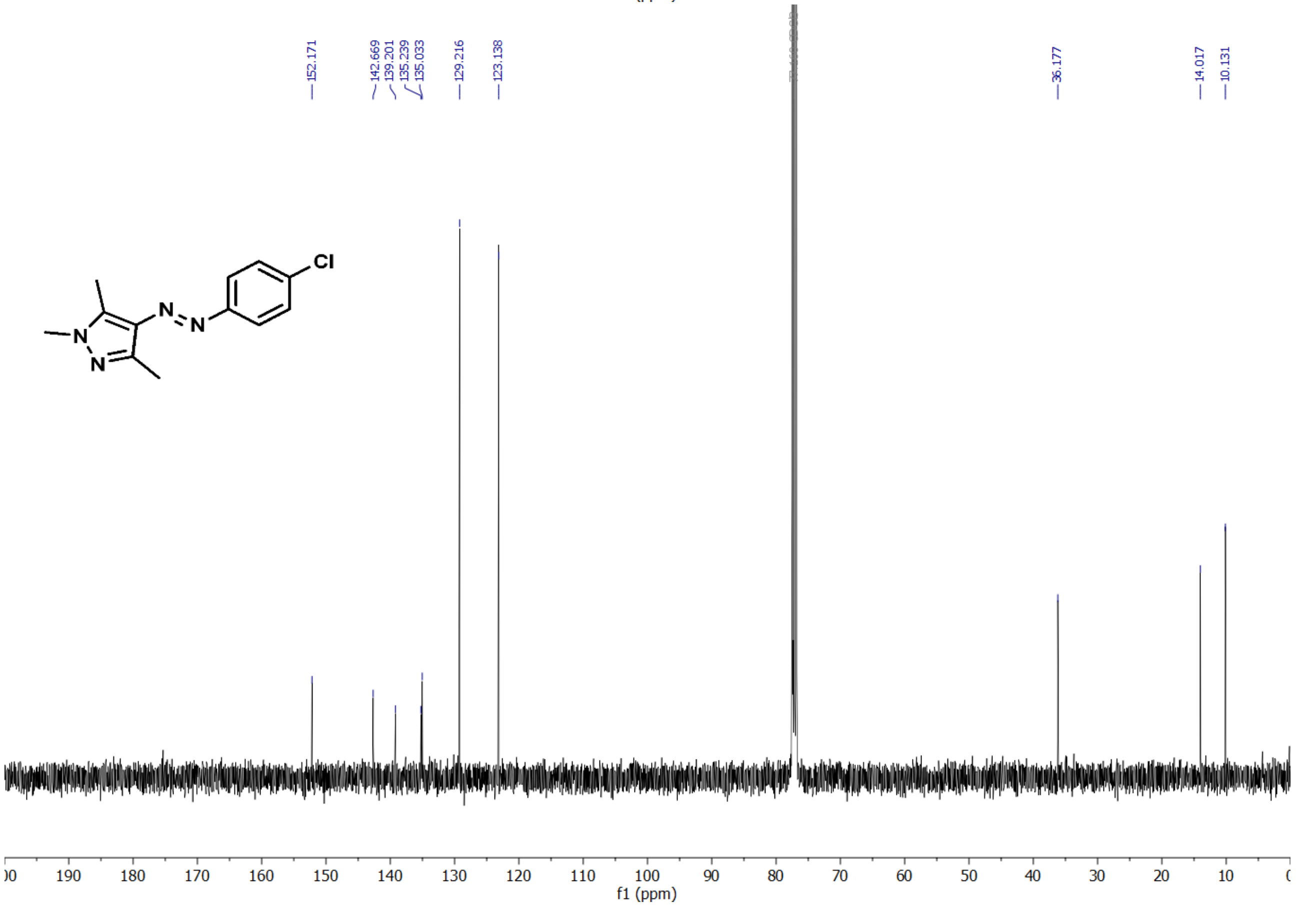

3-((4-Bromophenyl)diazenyl)-4-hydroxypent-3-en-2-one (2d)

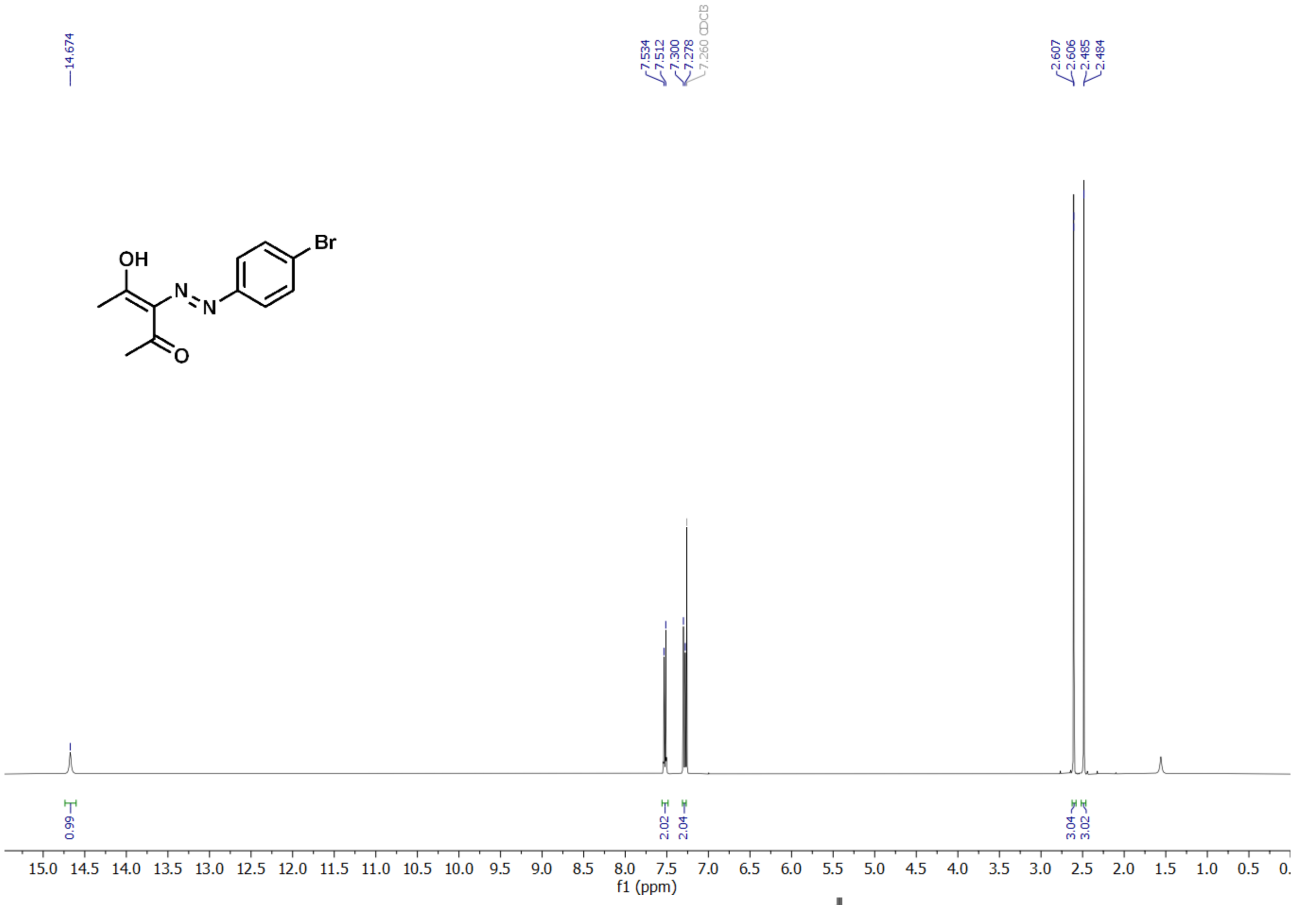

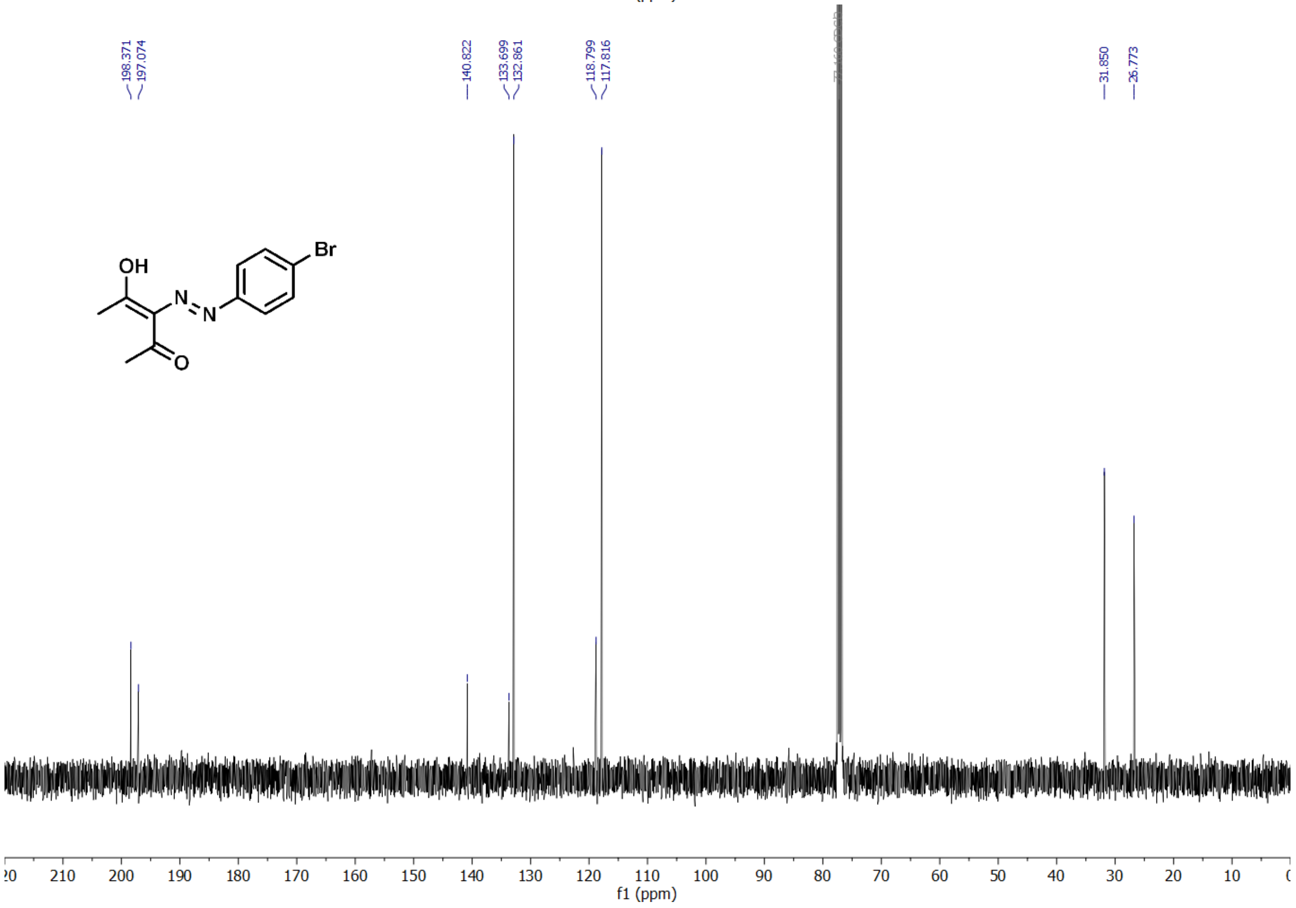

4-((4-Bromophenyl)diazenyl)-1,3,5-trimethyl-1H-pyrazole (3d)

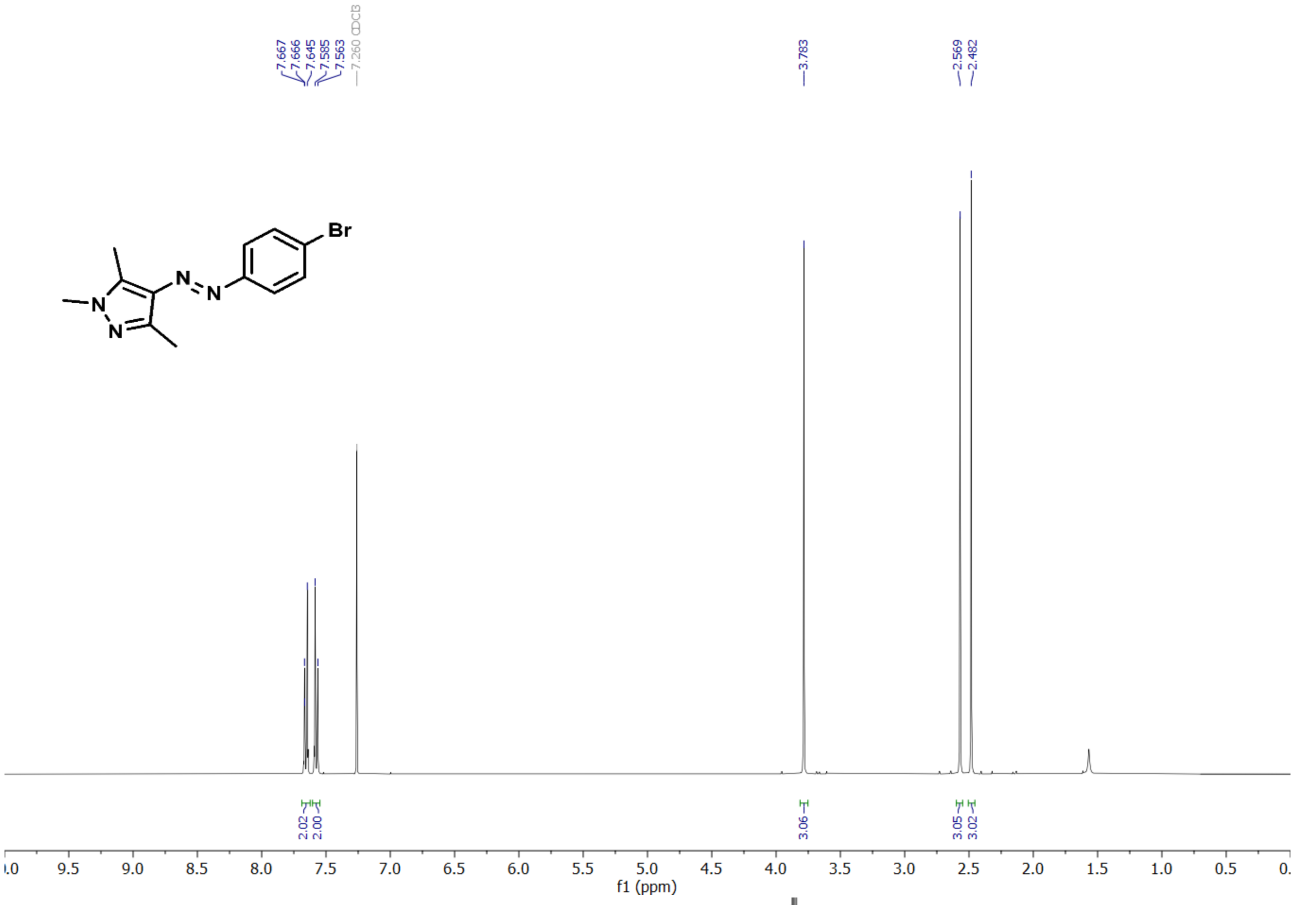

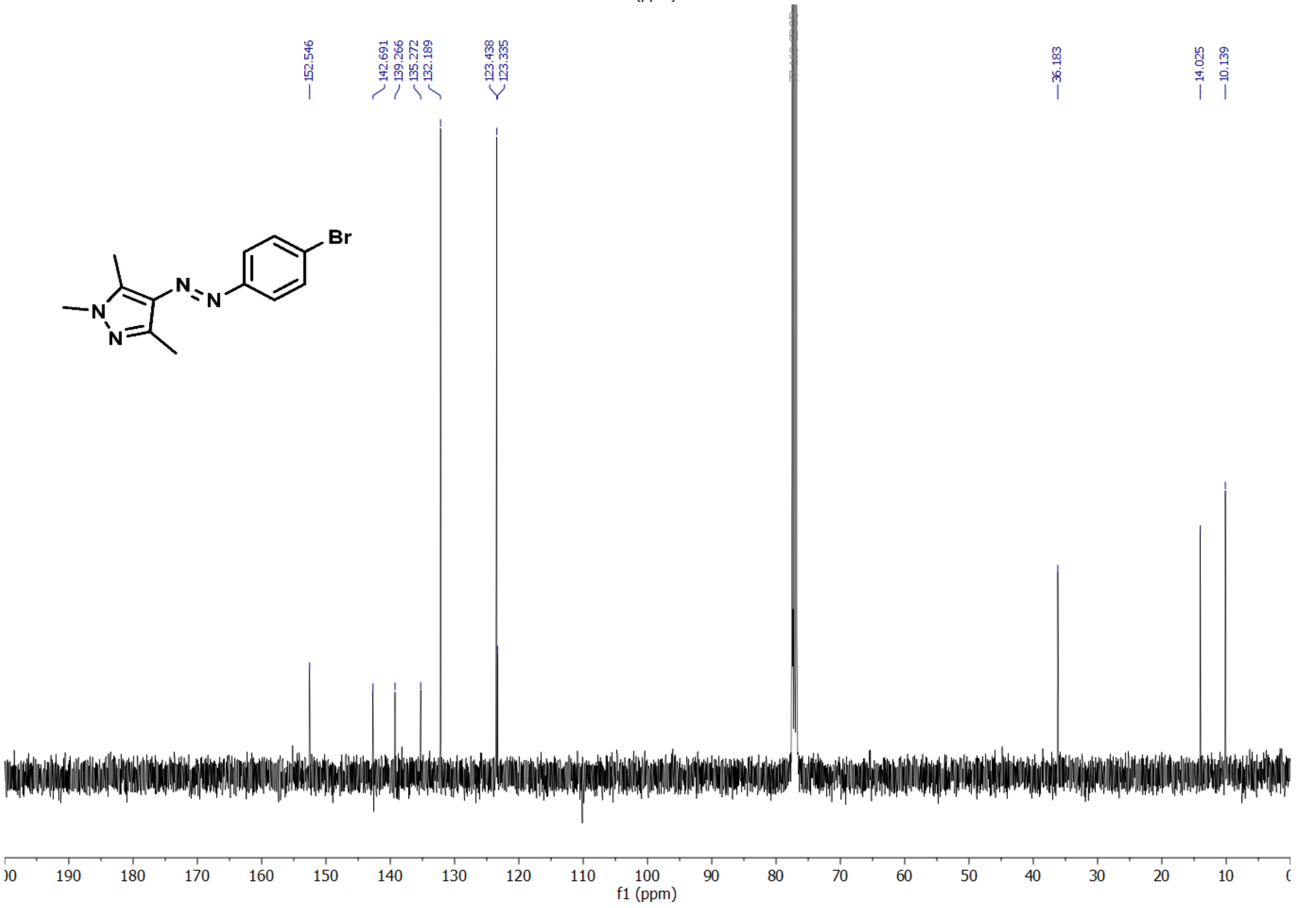

4-Hydroxy-3-((4-iodophenyl)diazenyl)pent-3-en-2-one (2e)

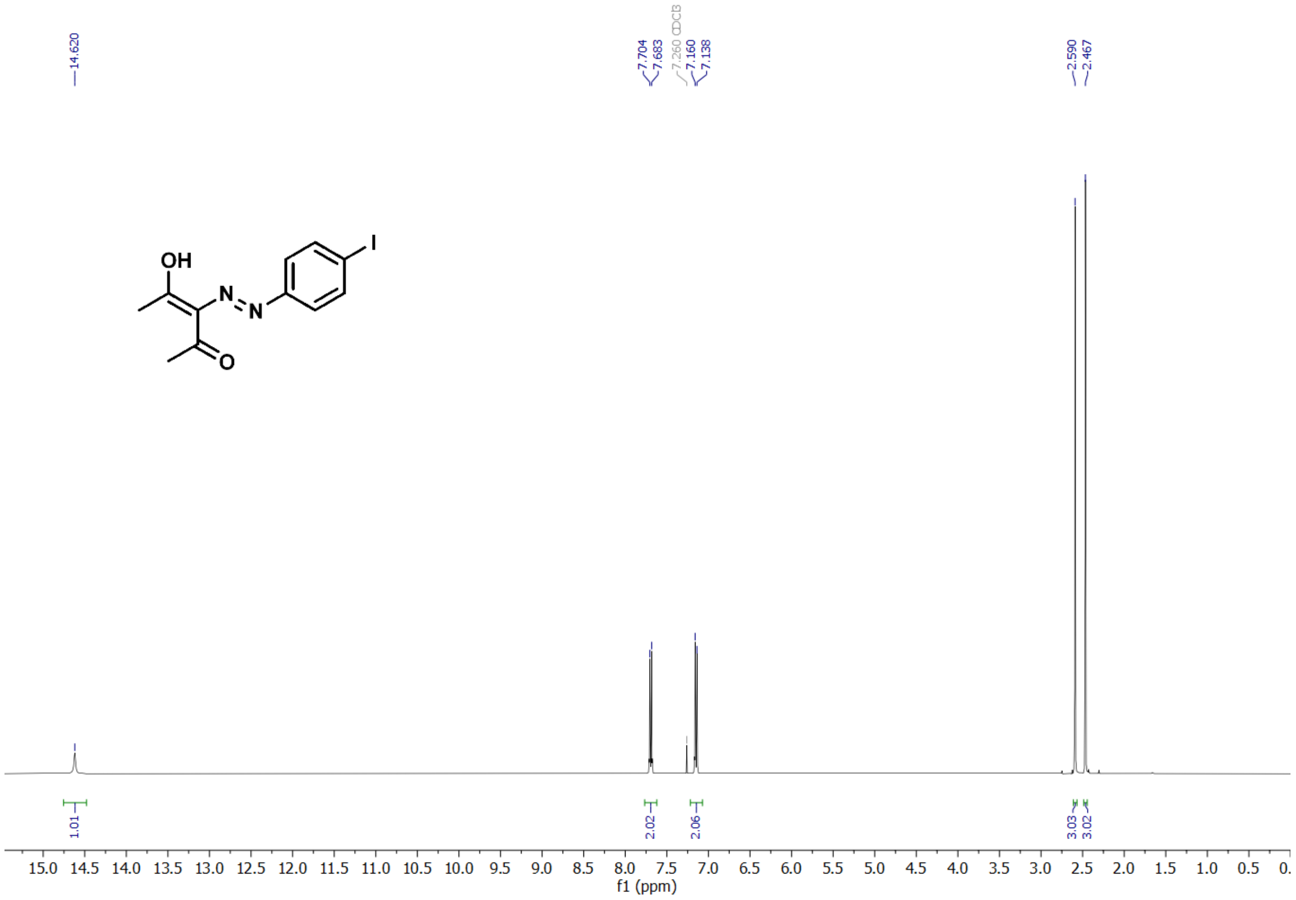

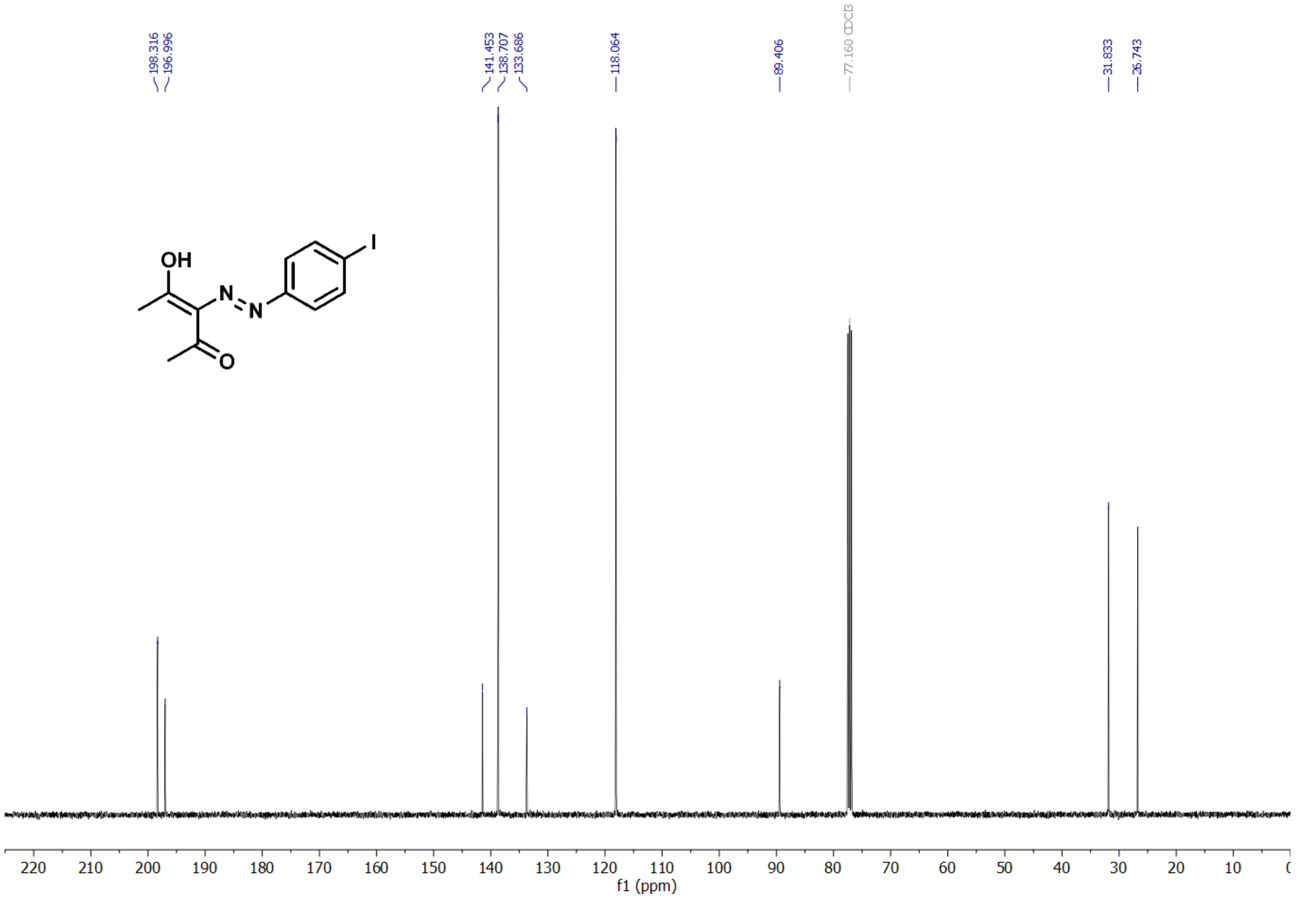

4-((4-Iodophenyl)diazenyl)-1,3,5-trimethyl-1H-pyrazole (3e)

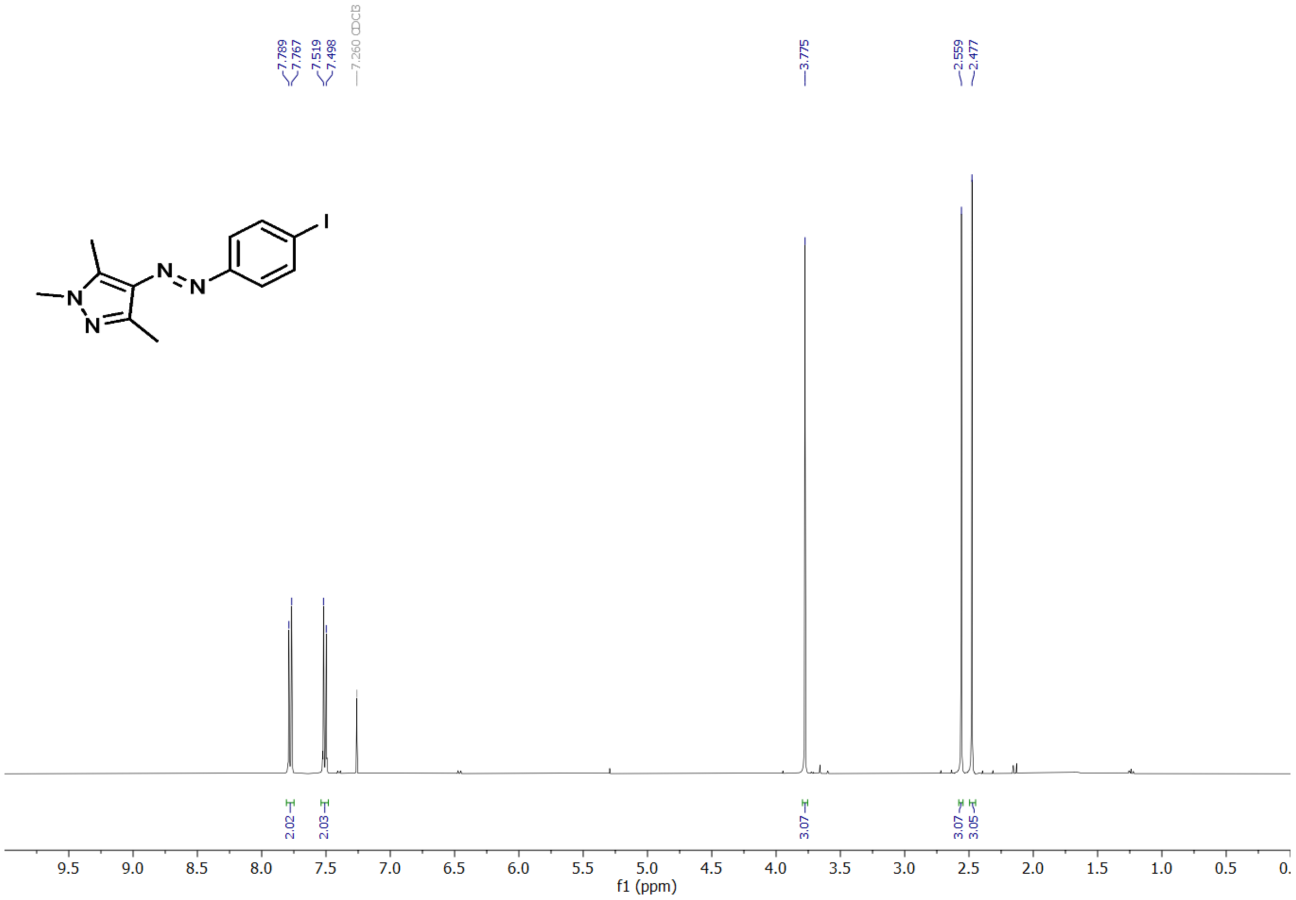

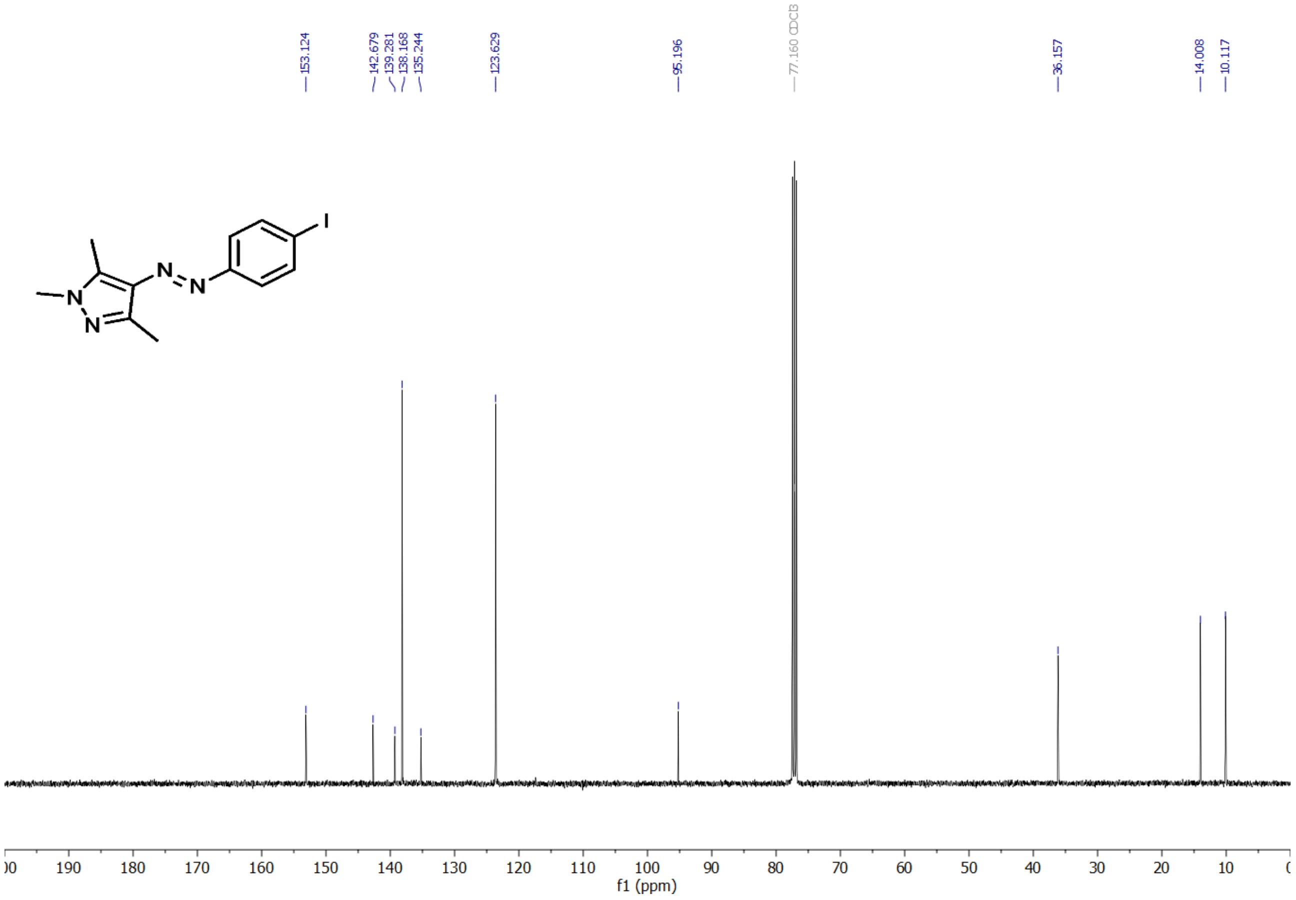

4-Hydroxy-3-(p-tolyldiazenyl)pent-3-en-2-one (2f)

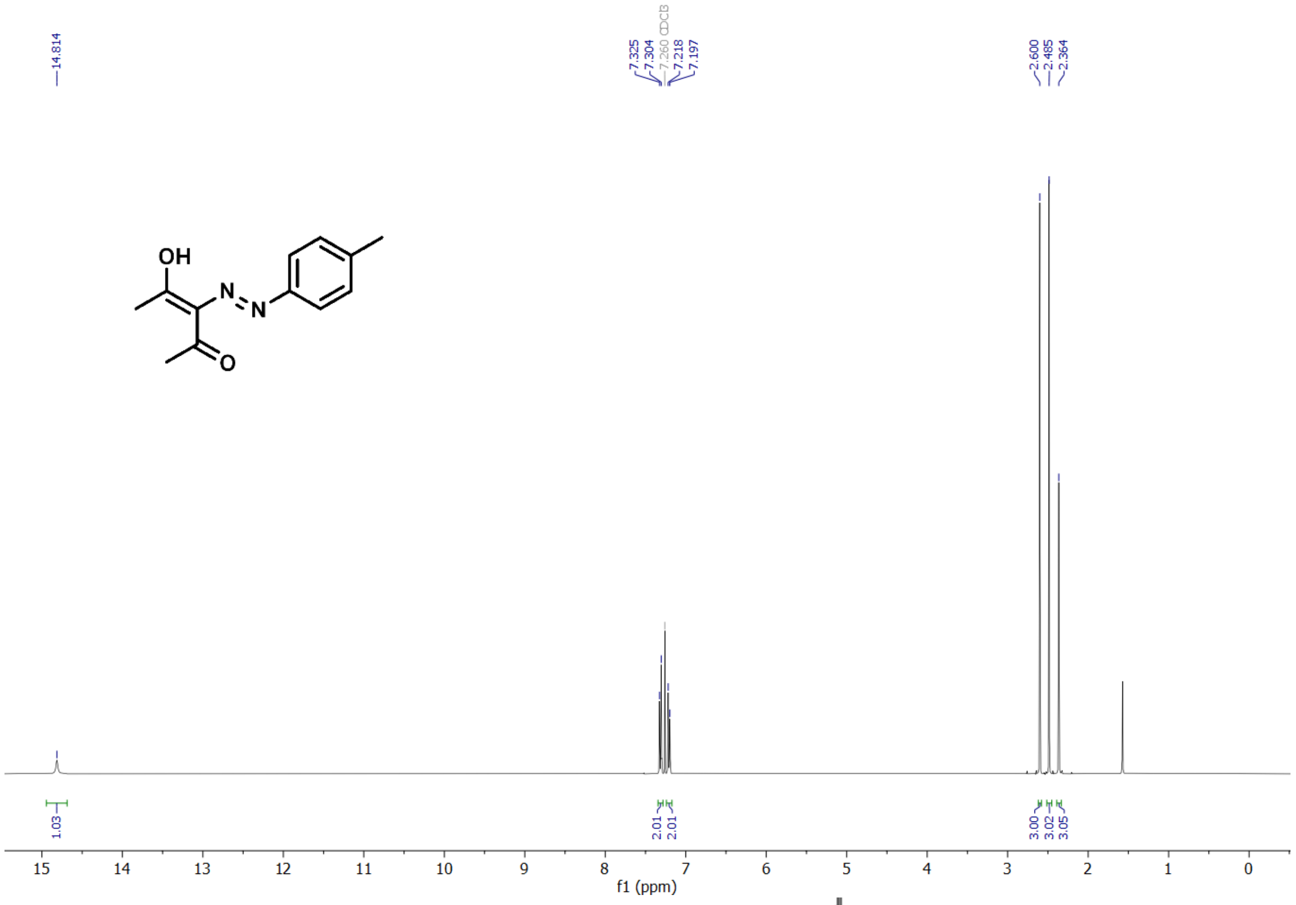

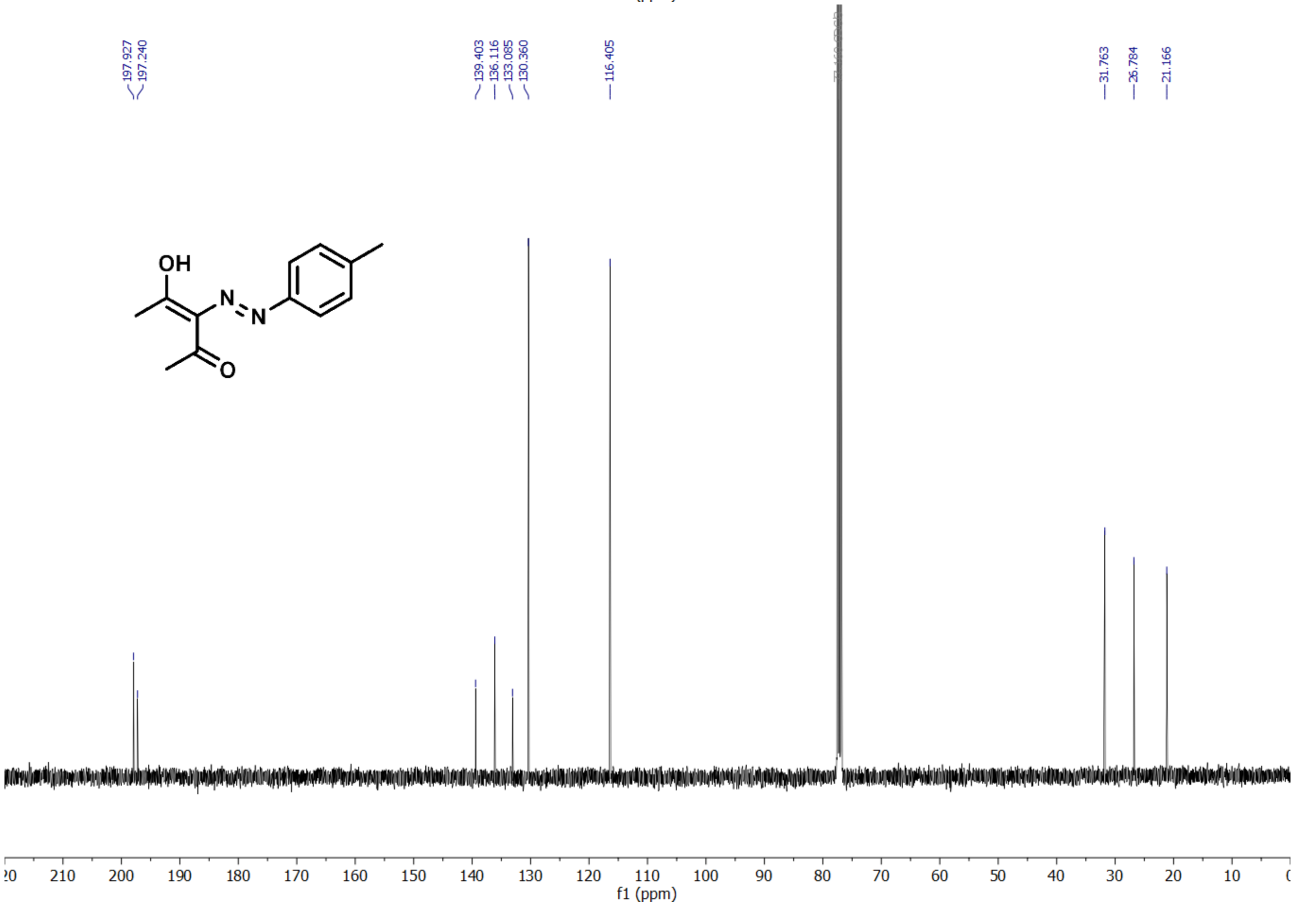

1,3,5-Trimethyl-4-(p-tolyldiazenyl)-1H-pyrazole (3f)

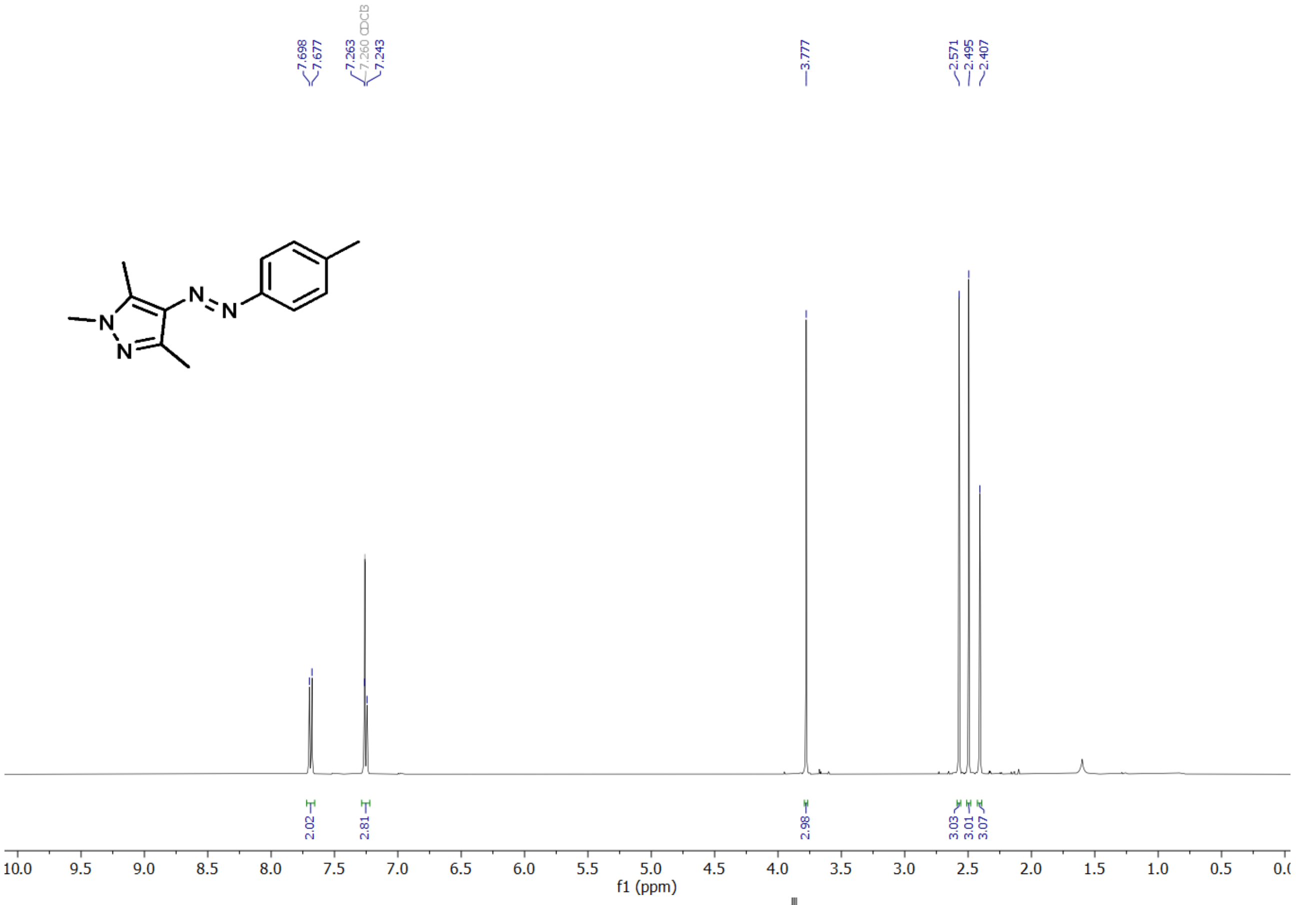

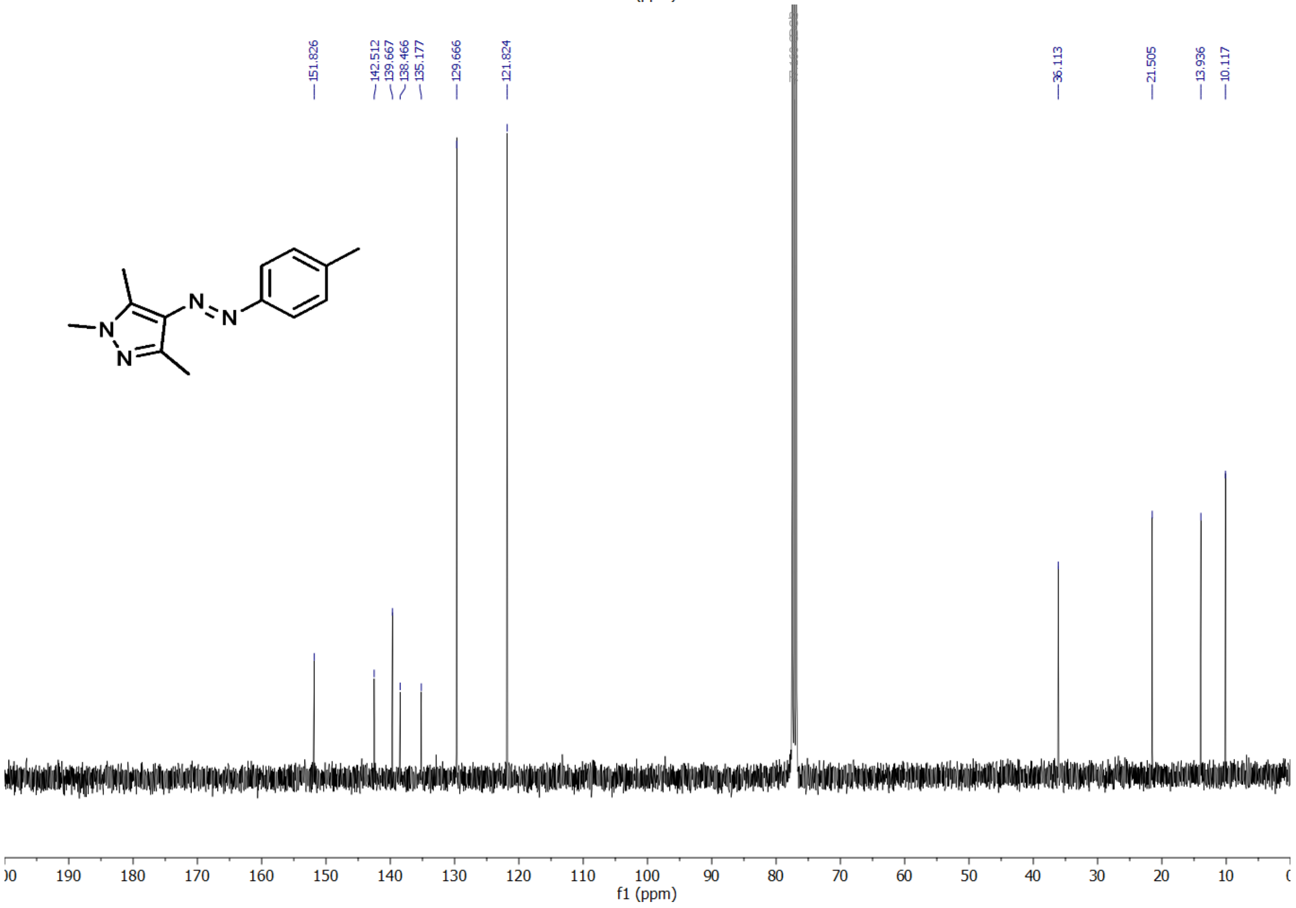

4-Hydroxy-3-((4-(trifluoromethyl)phenyl)diazenyl)pent-3-en-2-one (2g)

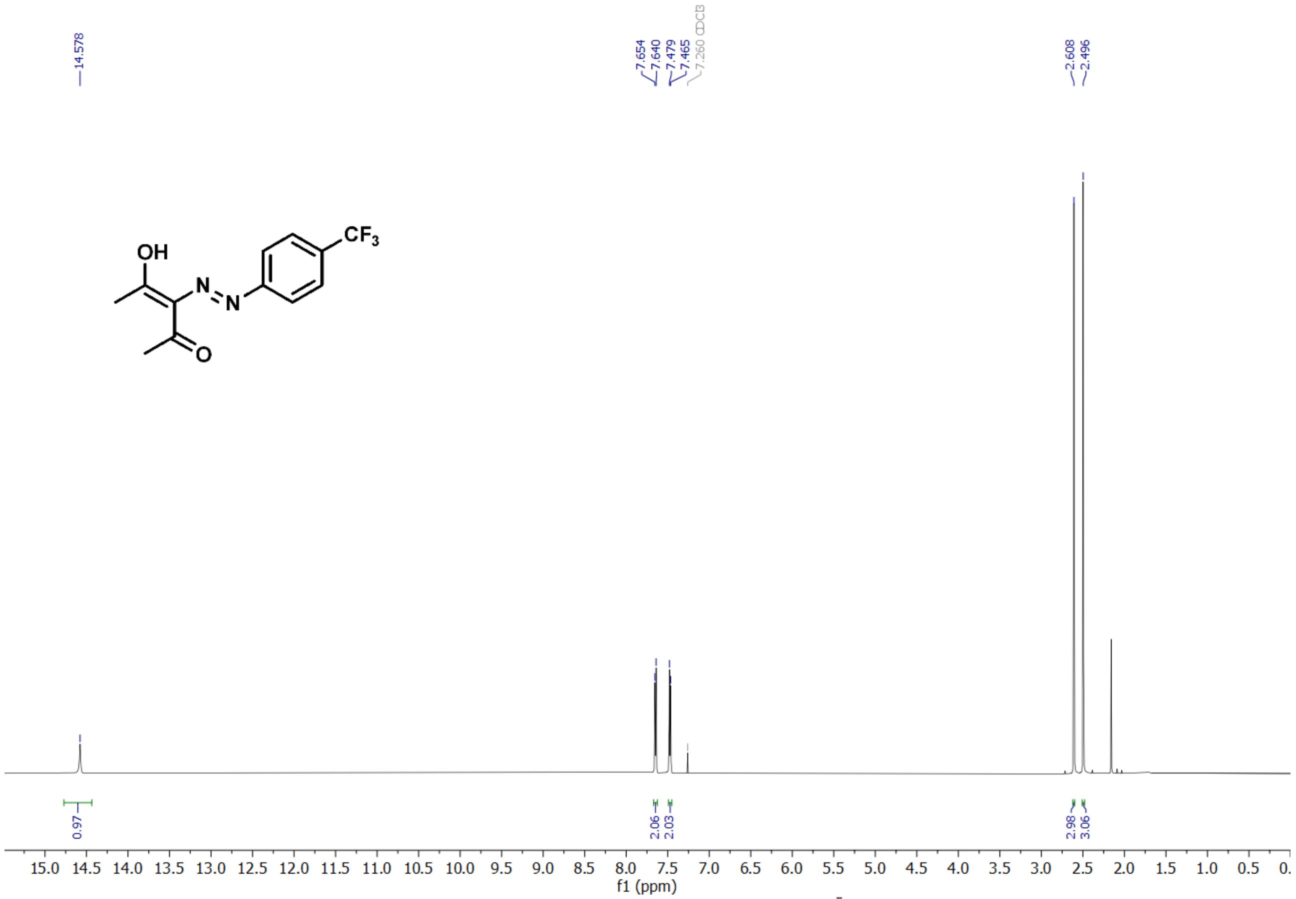

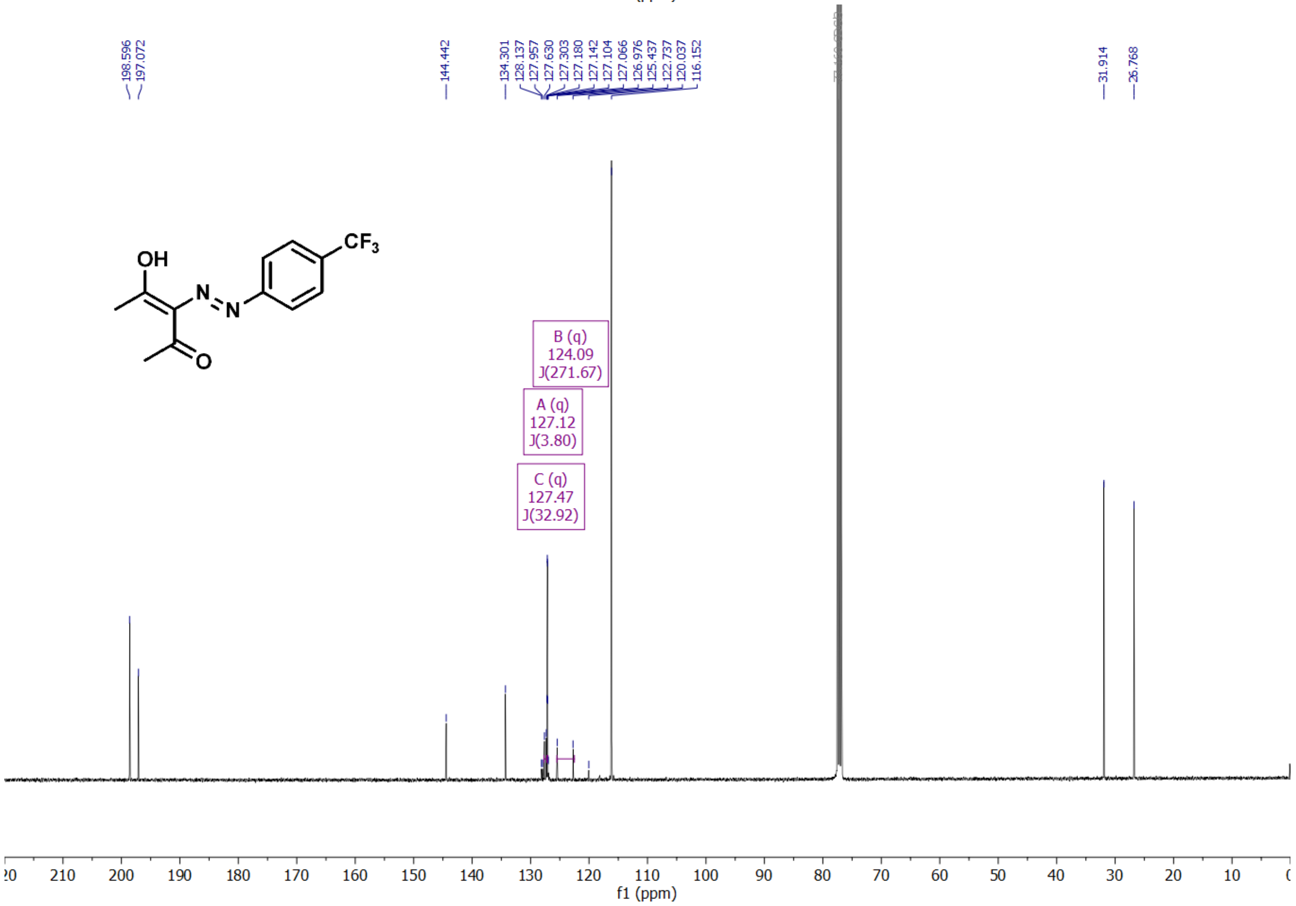

1,3,5-Trimethyl-4-((4-(trifluoromethyl)phenyl)diazenyl)-1H-pyrazole (3g)

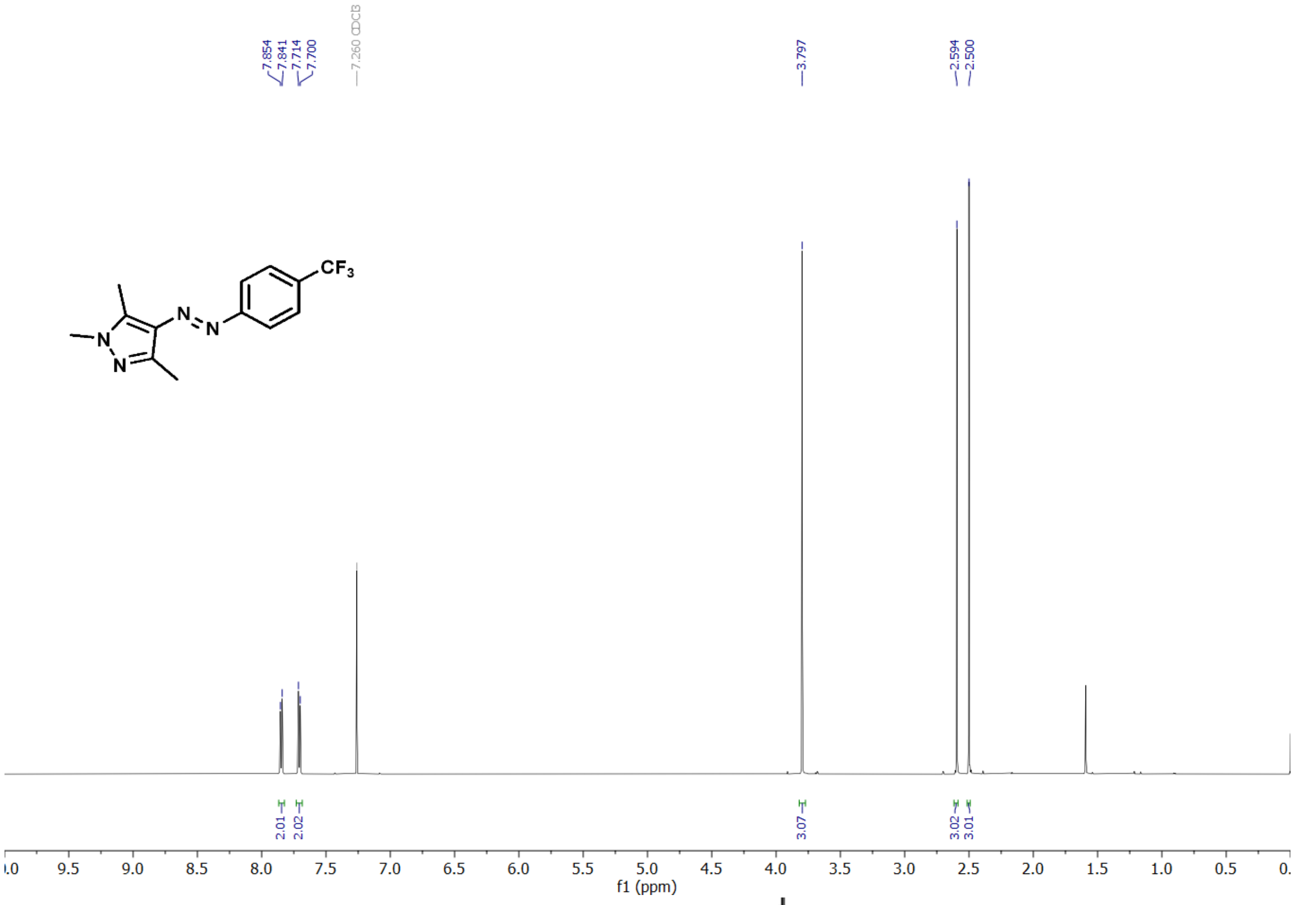

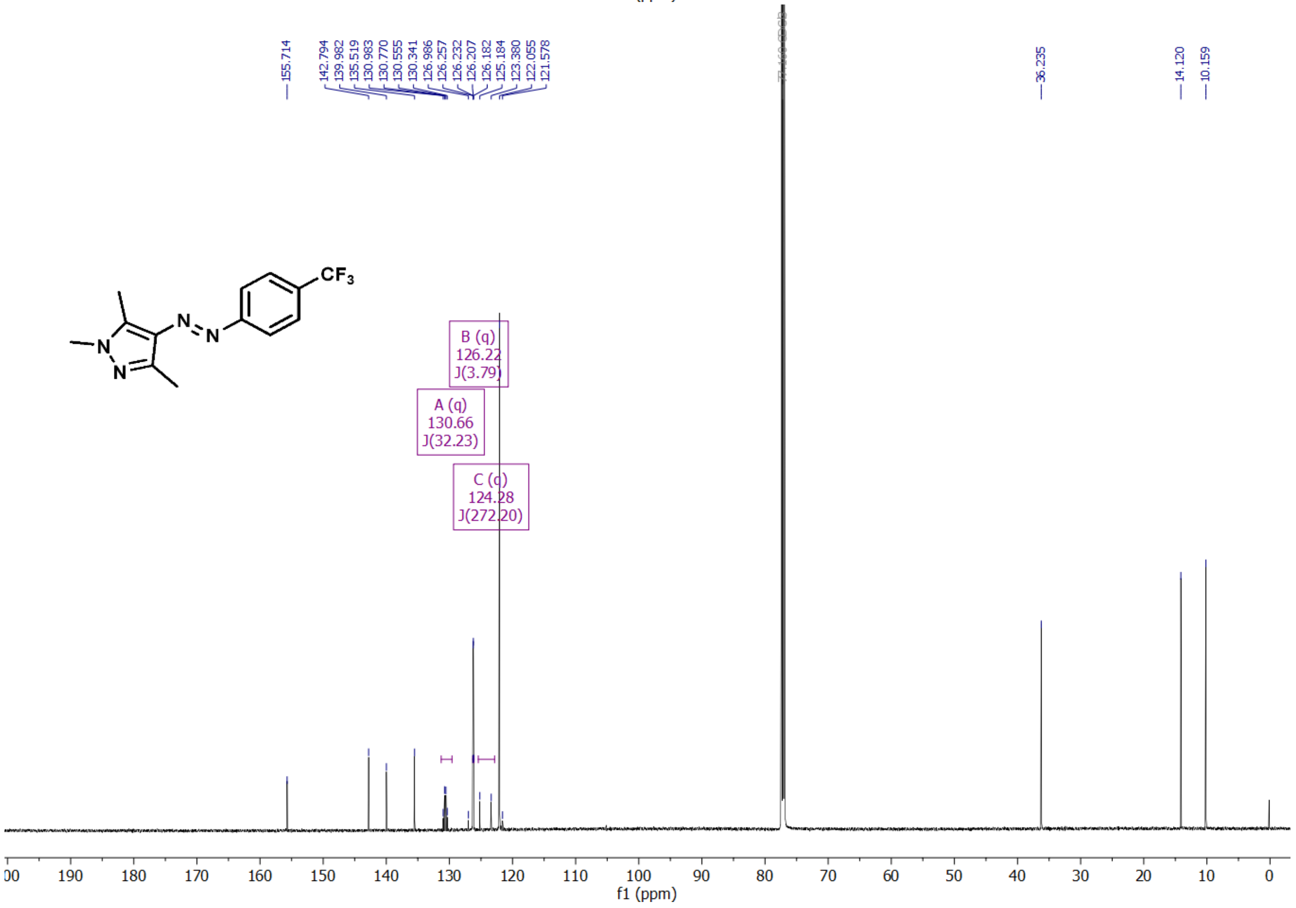

4-Hydroxy-3-((4-nitrophenyl)diazenyl)pent-3-en-2-one (2h)

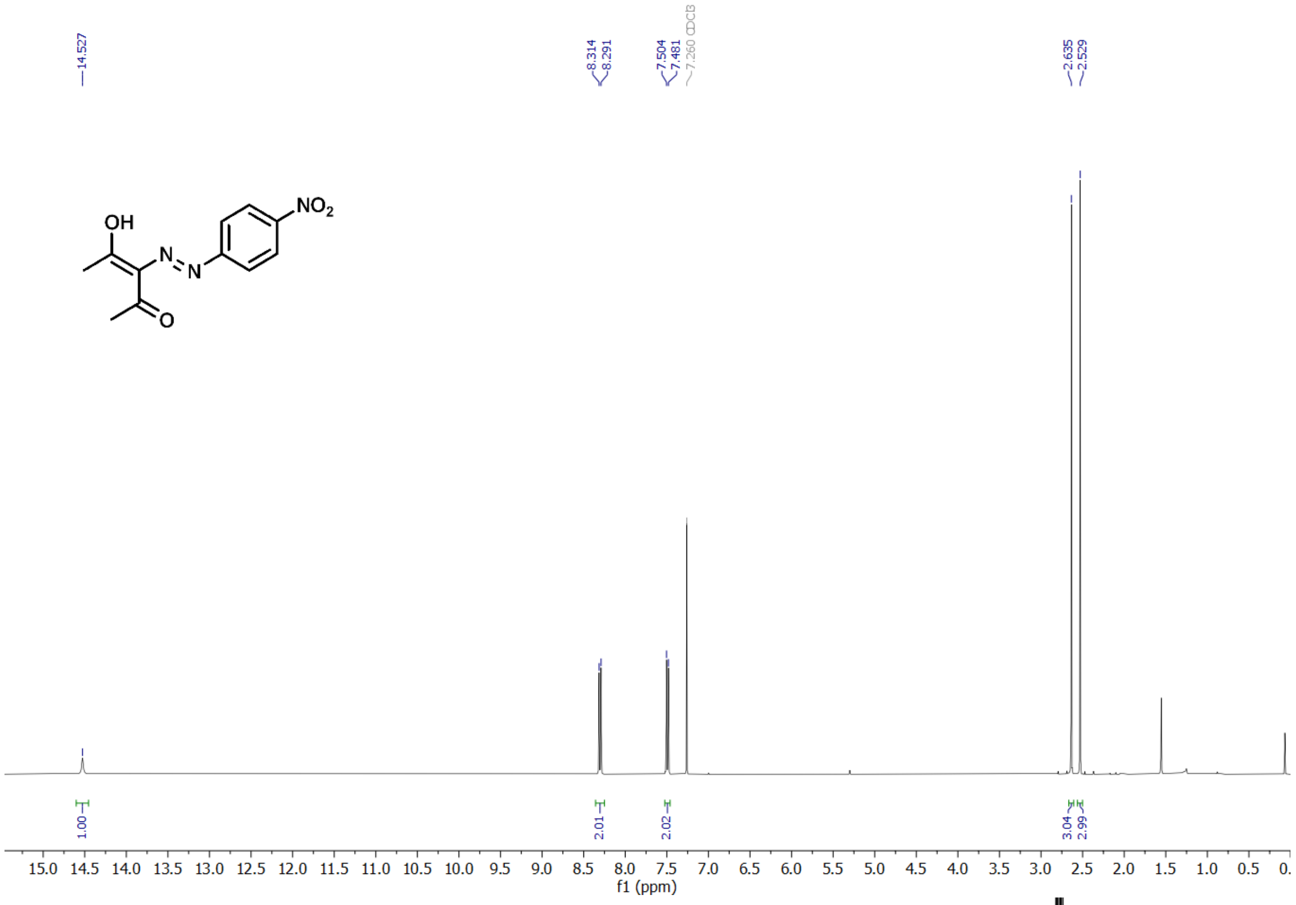

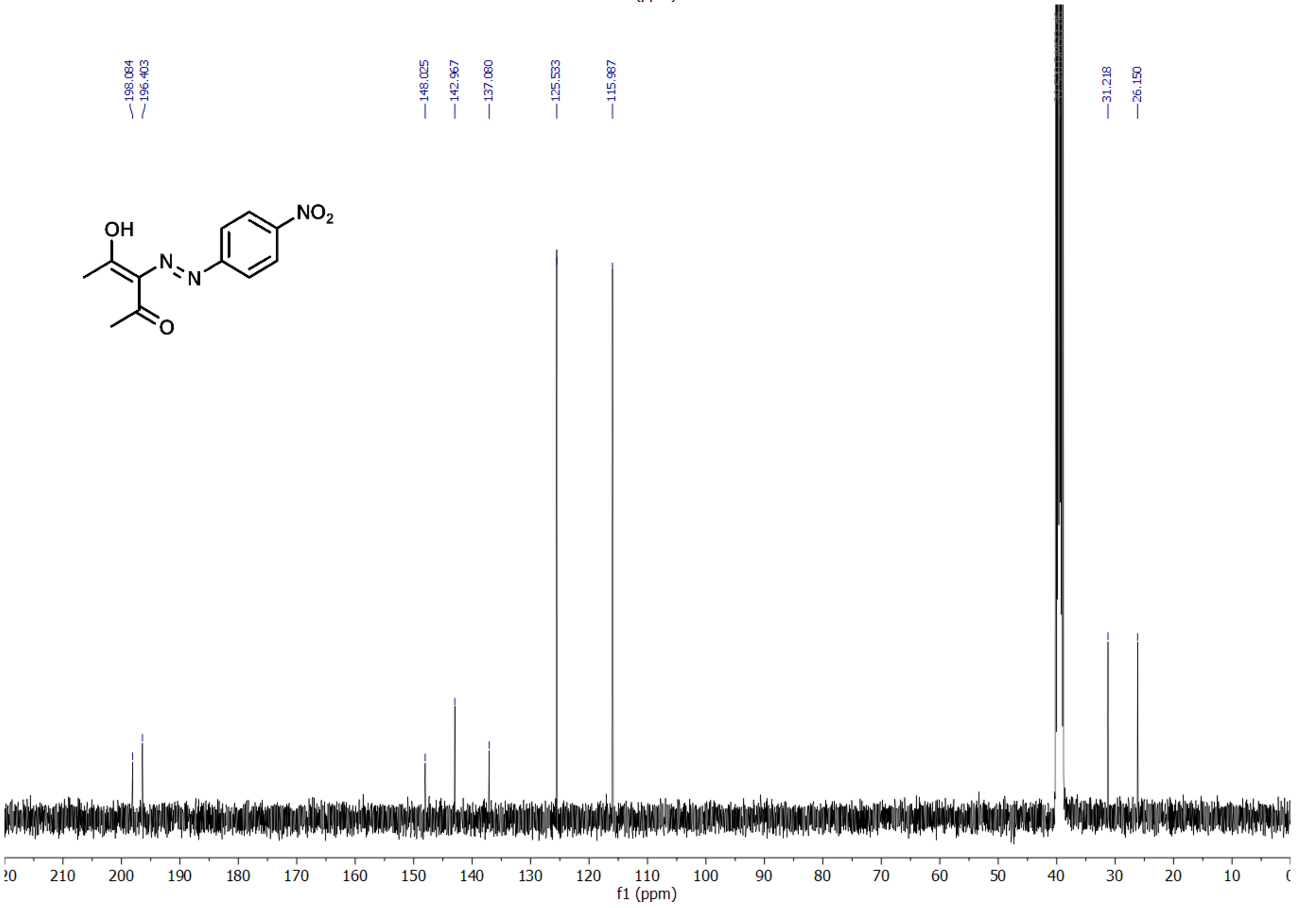

1,3,5-Trimethyl-4-((4-nitrophenyl)diazenyl)-1H-pyrazole (3h)

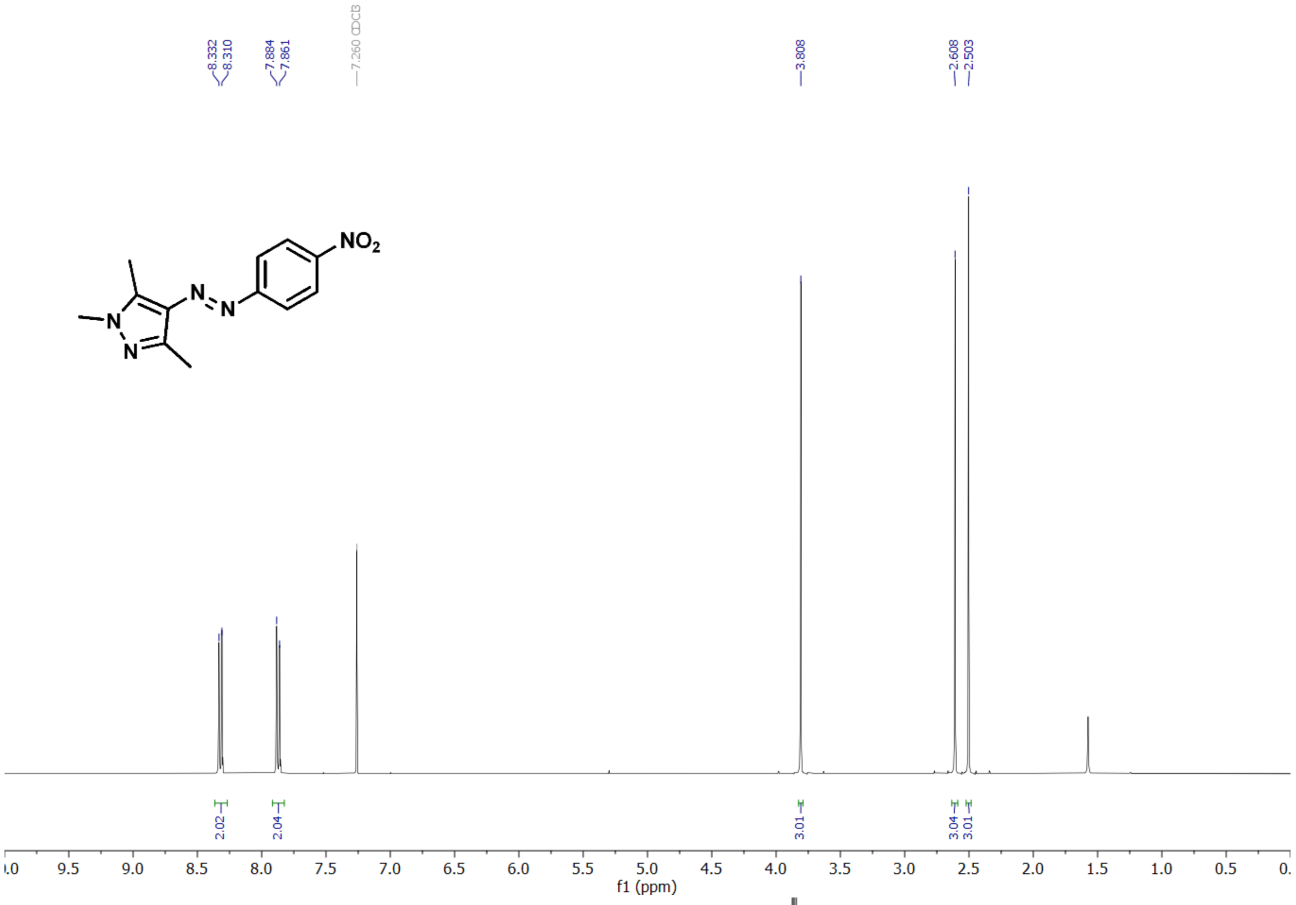

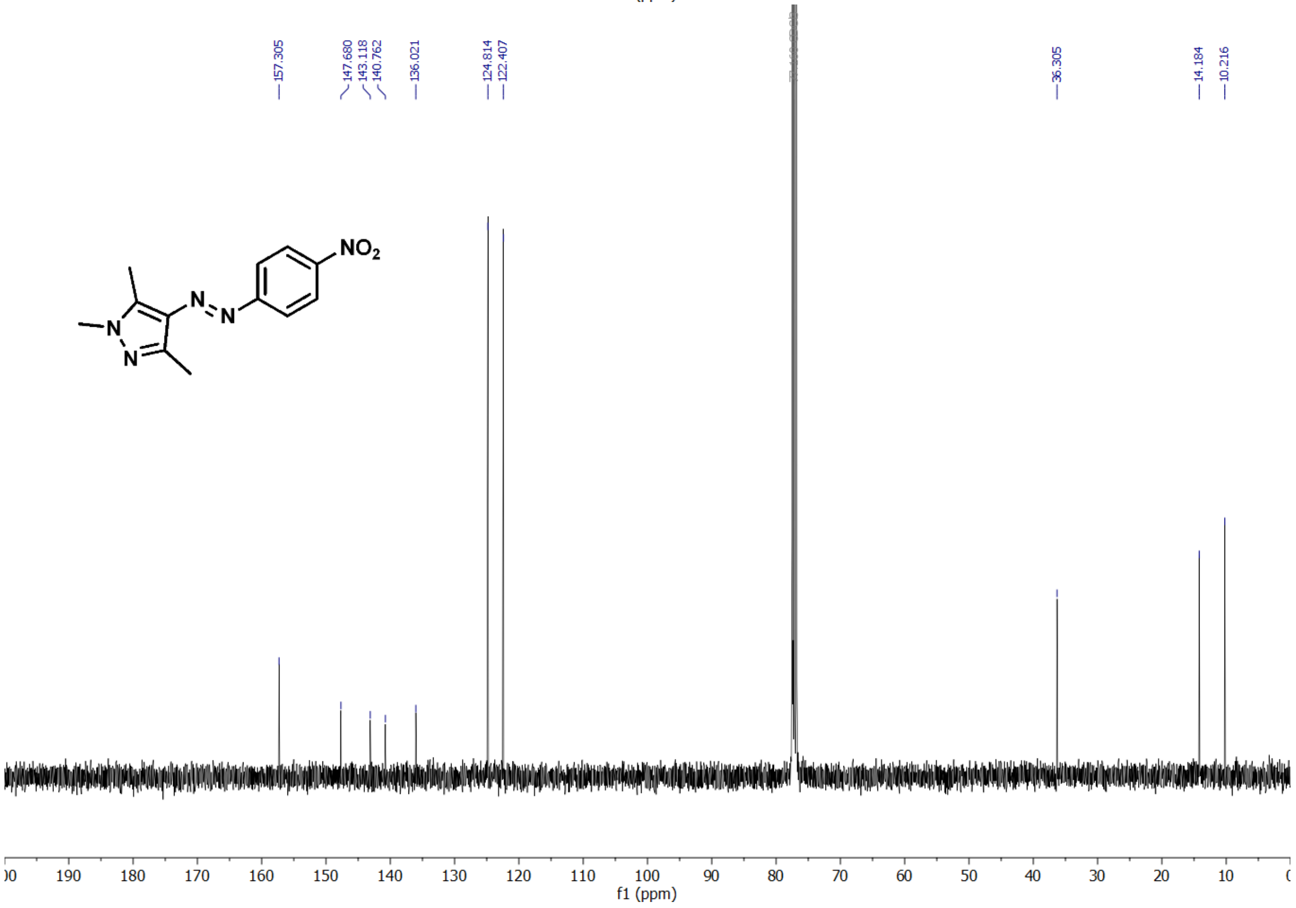

4-Hydroxy-3-((4-hydroxyphenyl)diazenyl)pent-3-en-2-one (2i)

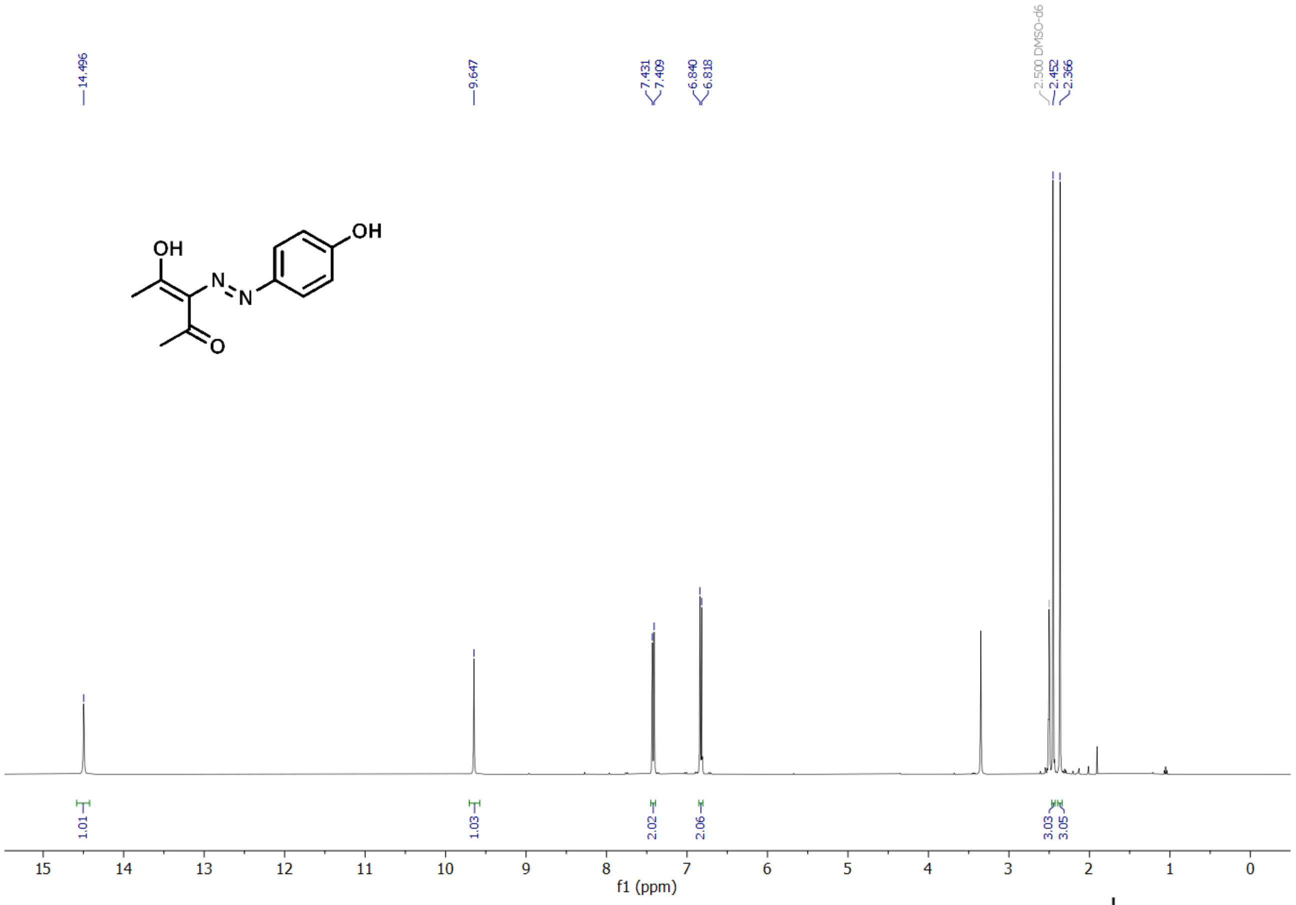

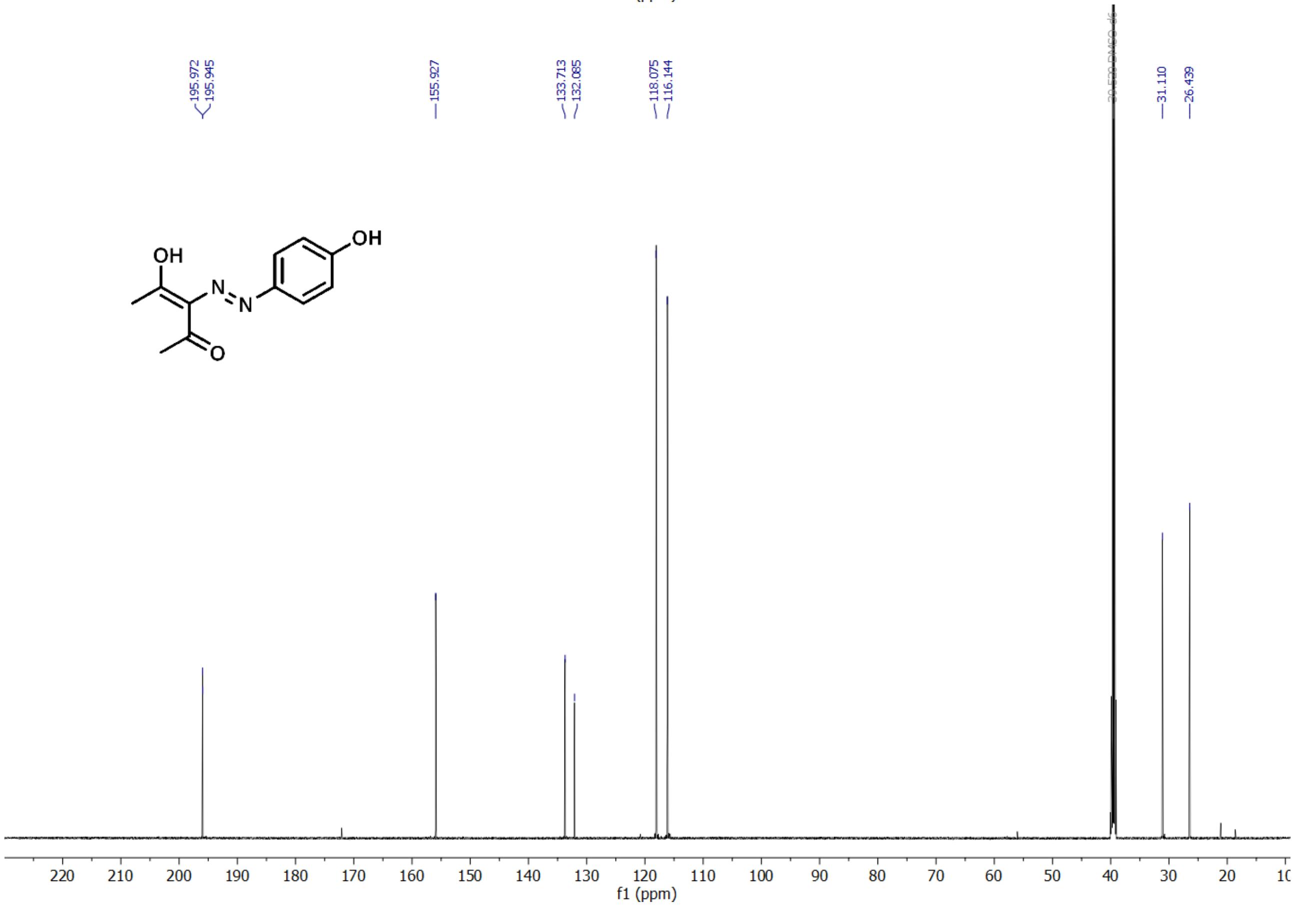

4-((1,3,5-Trimethyl-1H-pyrazol-4-yl)diazenyl)phenol (3i)

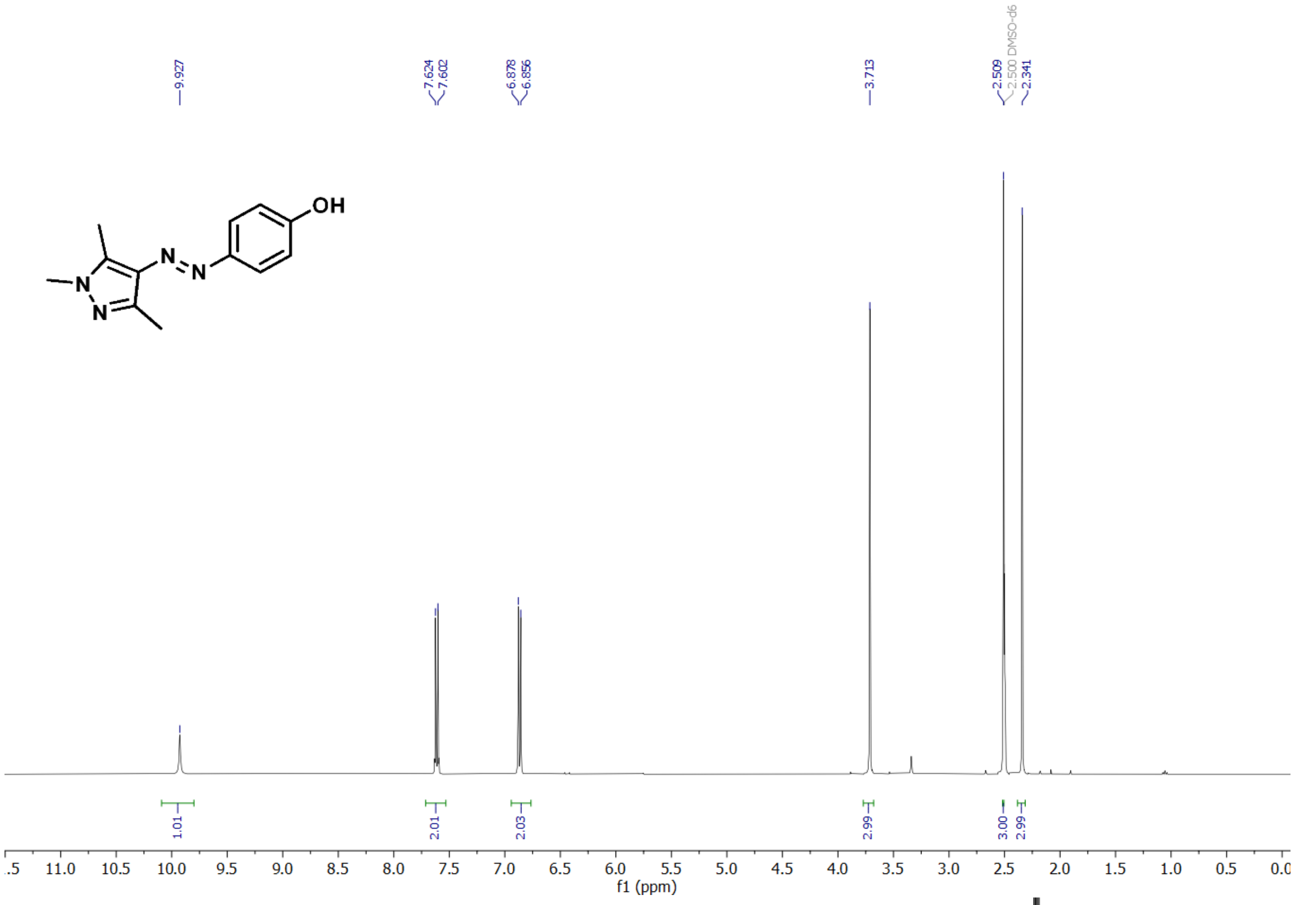

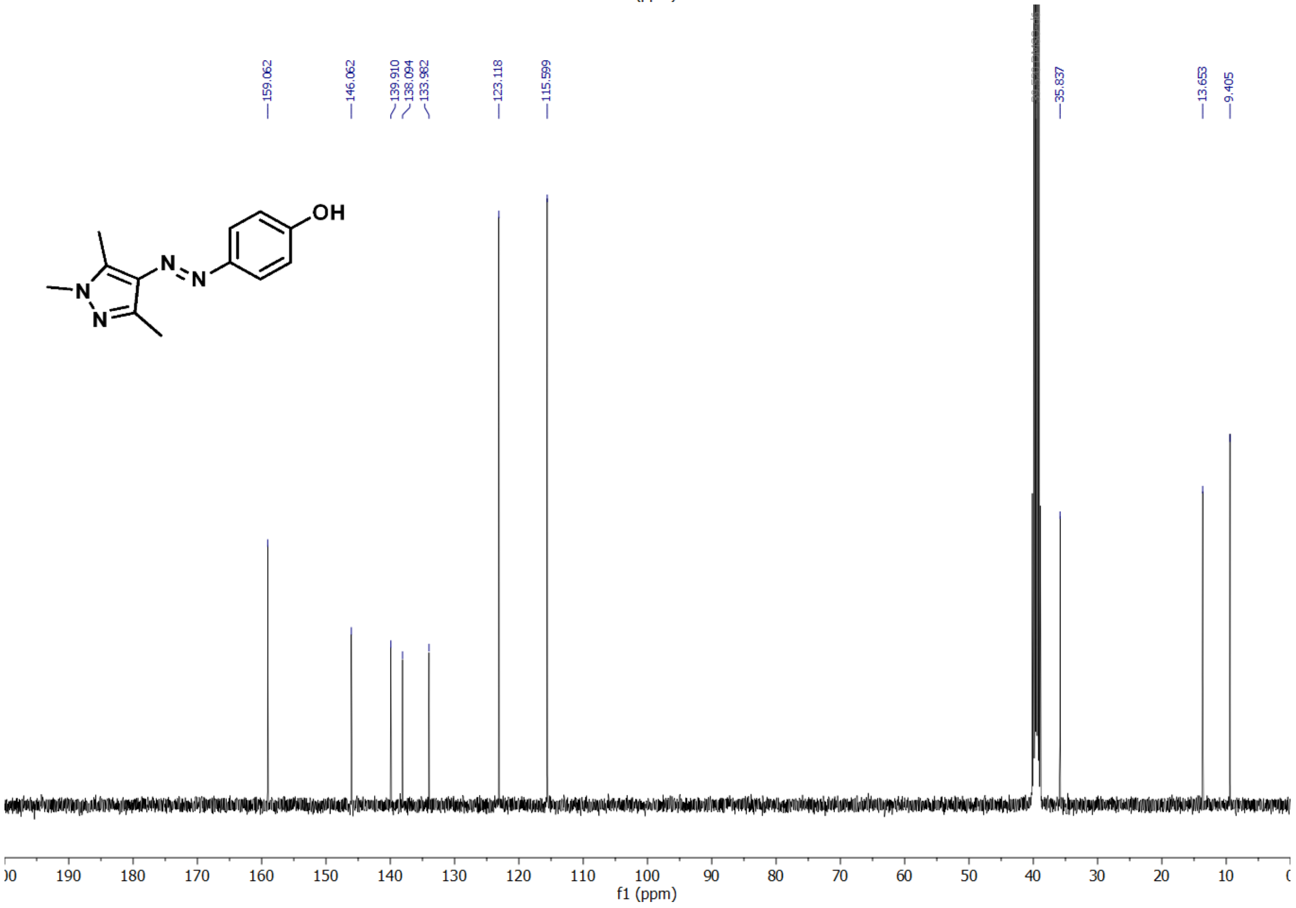

4-((2-Hydroxy-4-oxopent-2-en-3-yl)diazenyl)benzoic acid (2j)

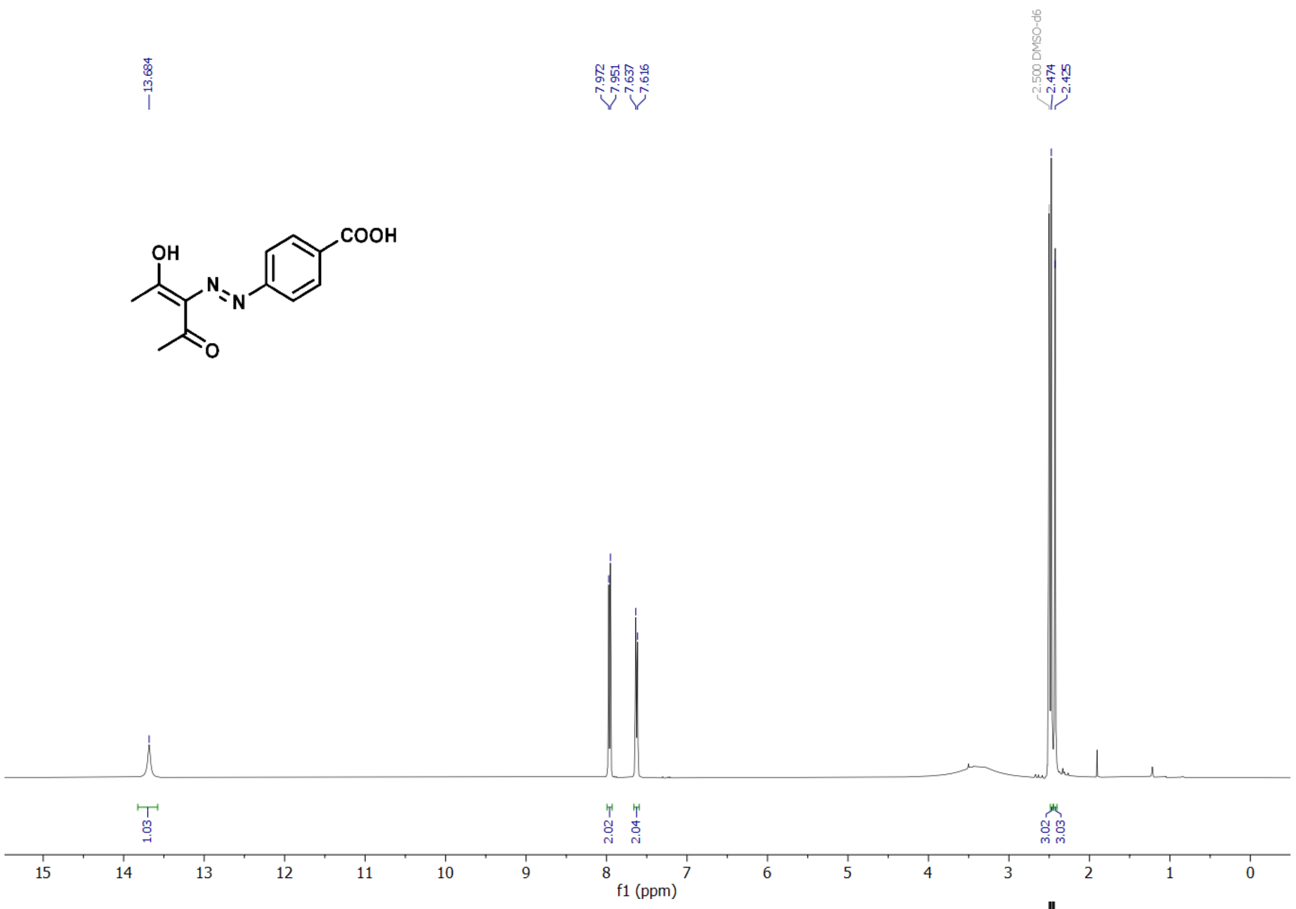

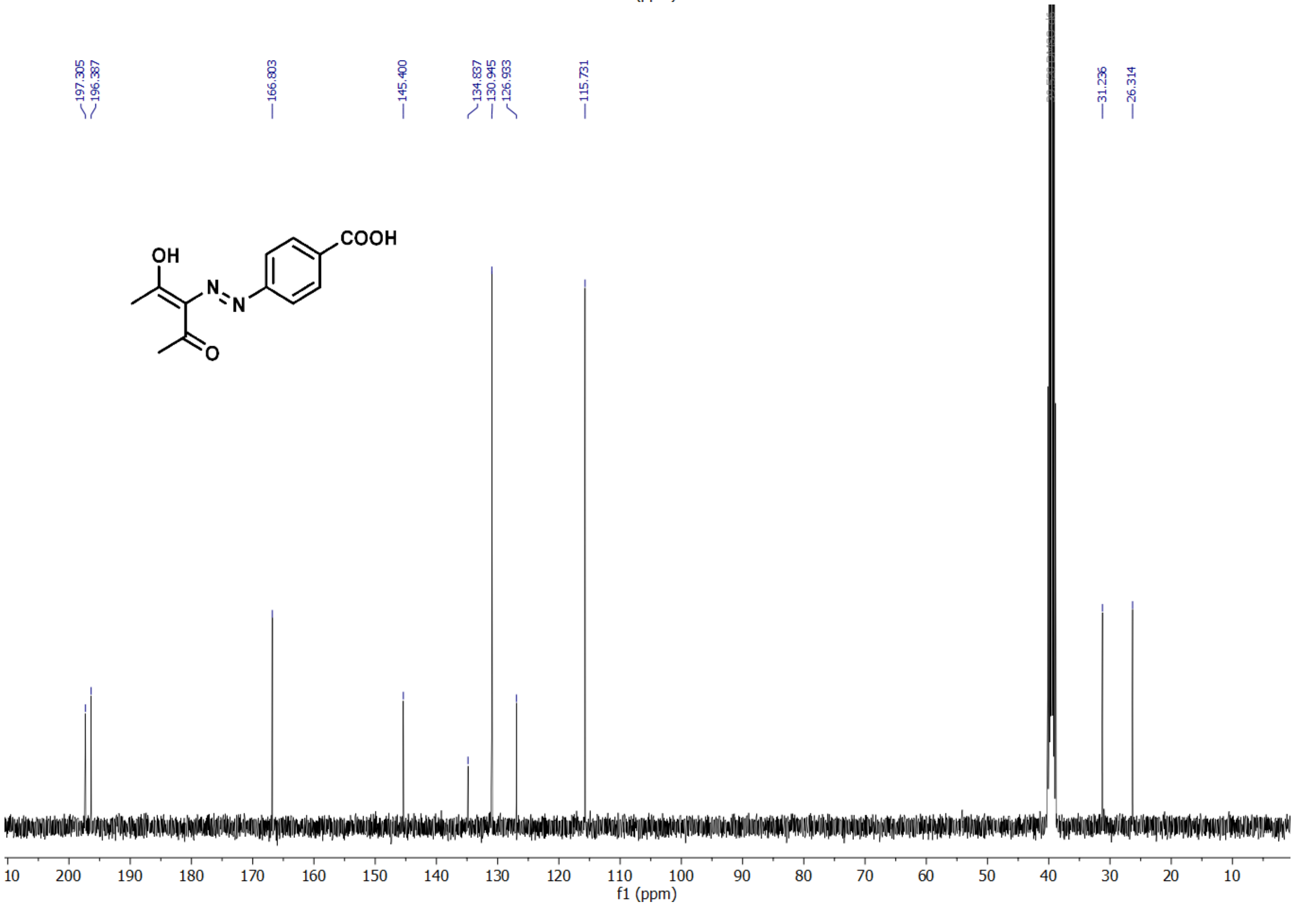

4-((1,3,5-Trimethyl-1H-pyrazol-4-yl)diazenyl)benzoic acid (3j)

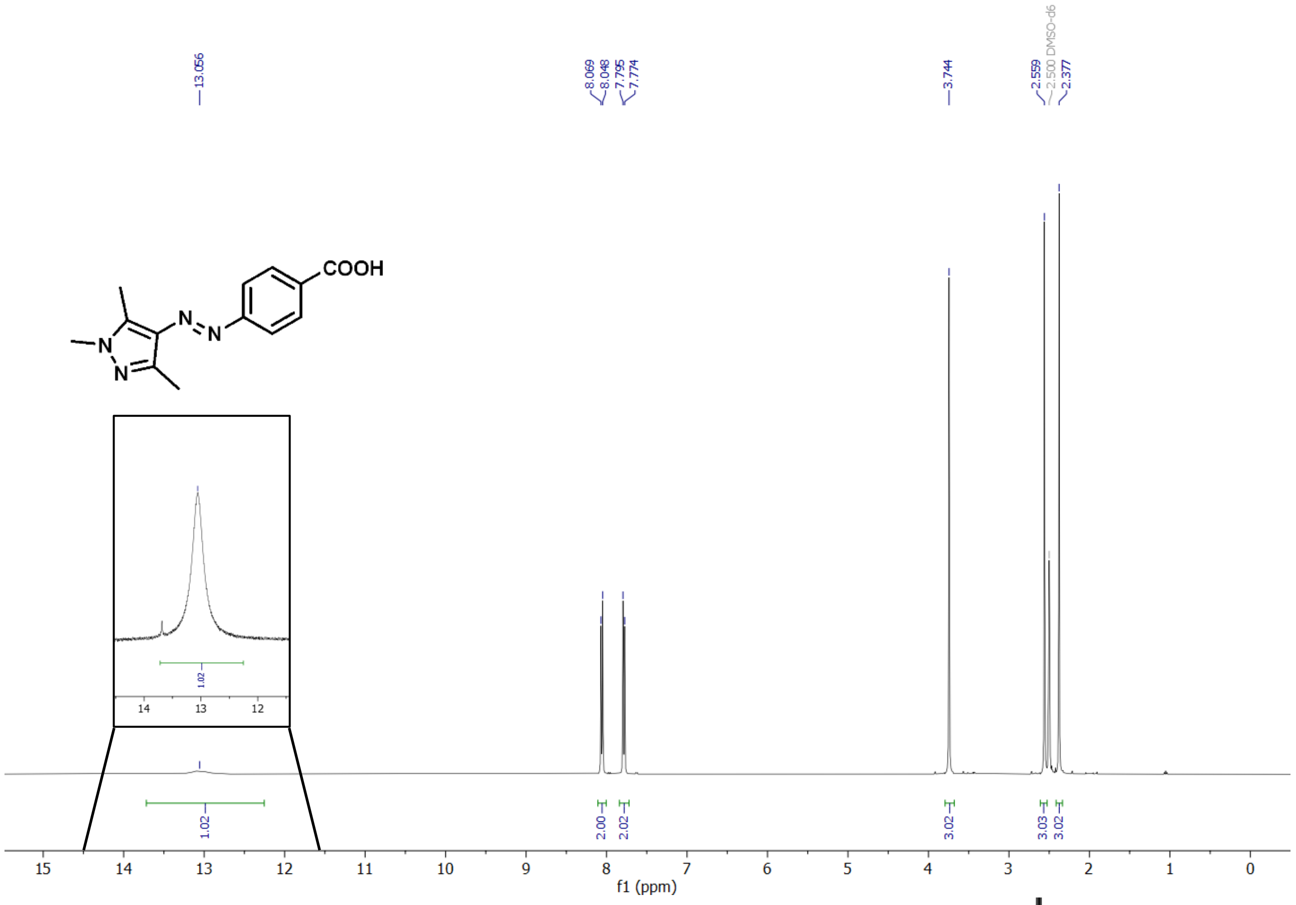

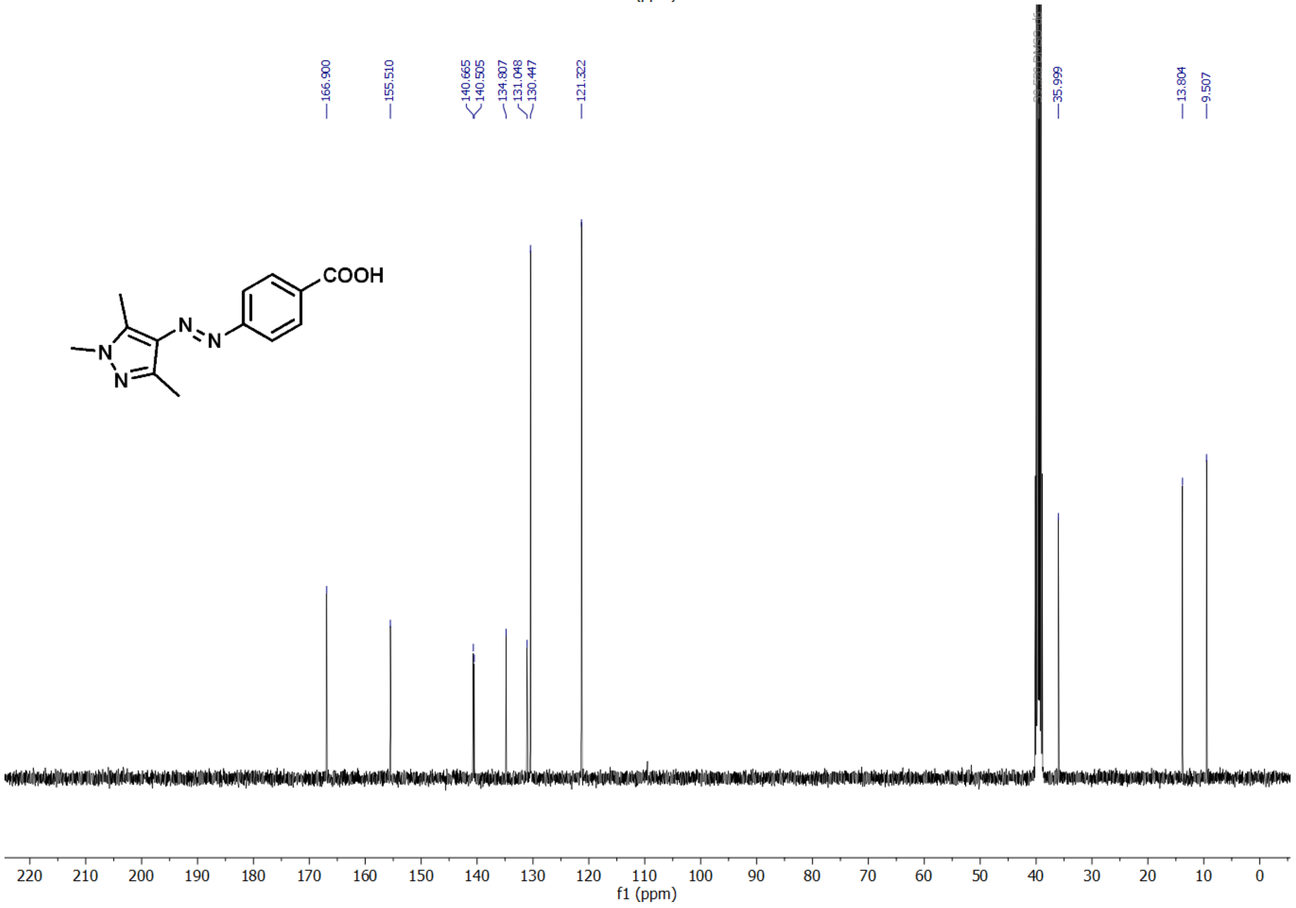

4-((2-Hydroxy-4-oxopent-2-en-3-yl)diazenyl)benzonitrile (2k)

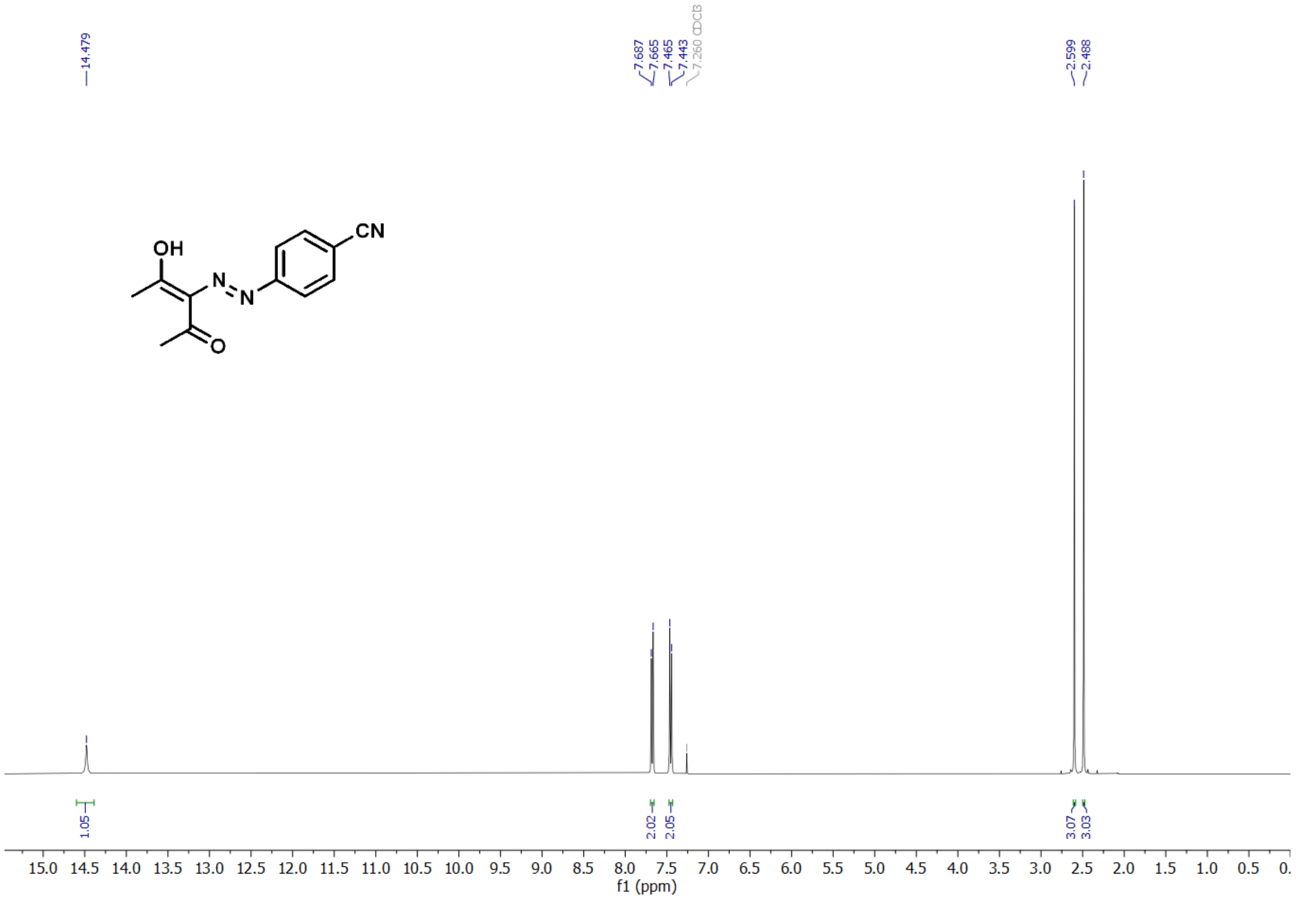

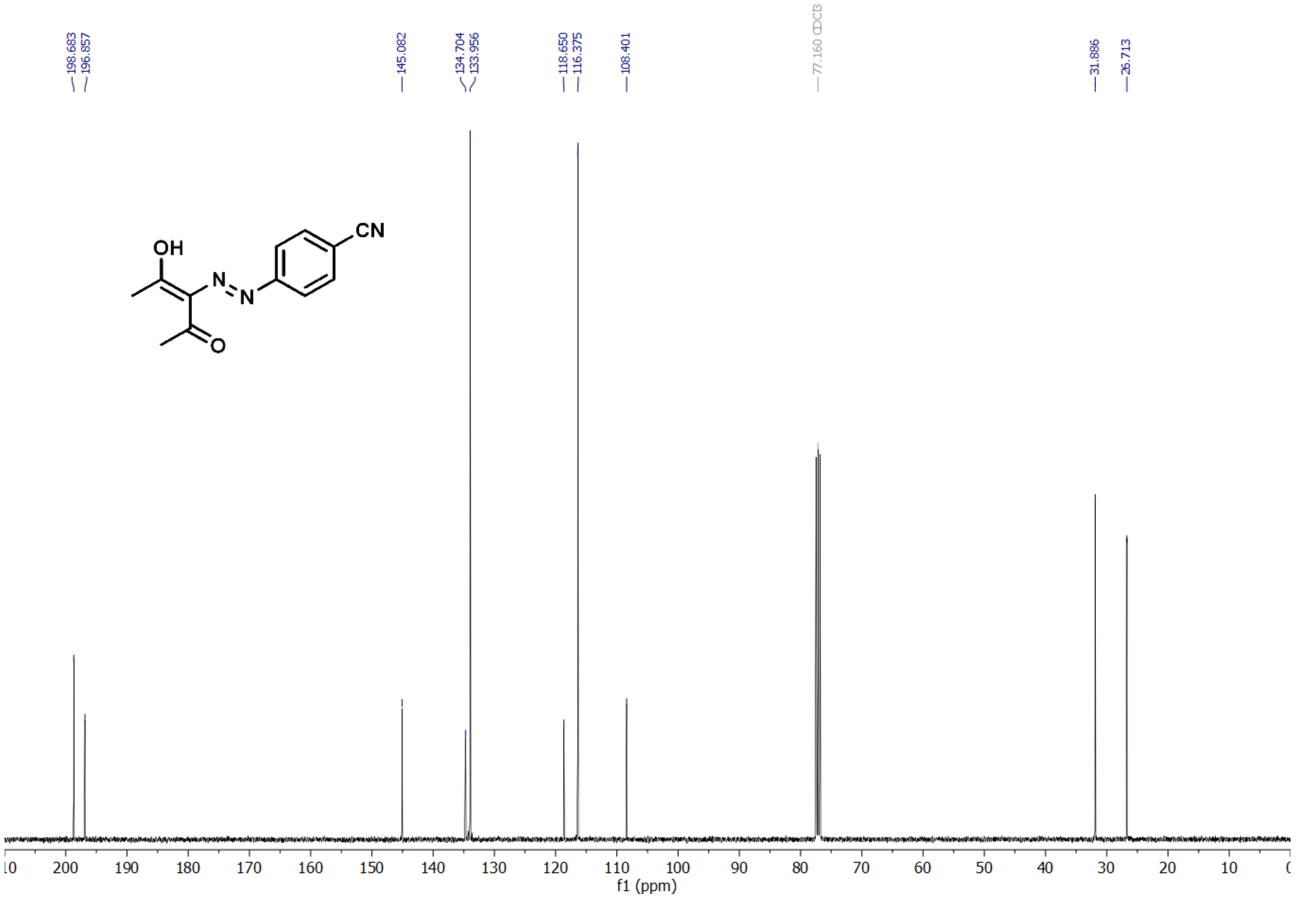

4-((1,3,5-Trimethyl-1H-pyrazol-4-yl)diazenyl)benzonitrile (3k)

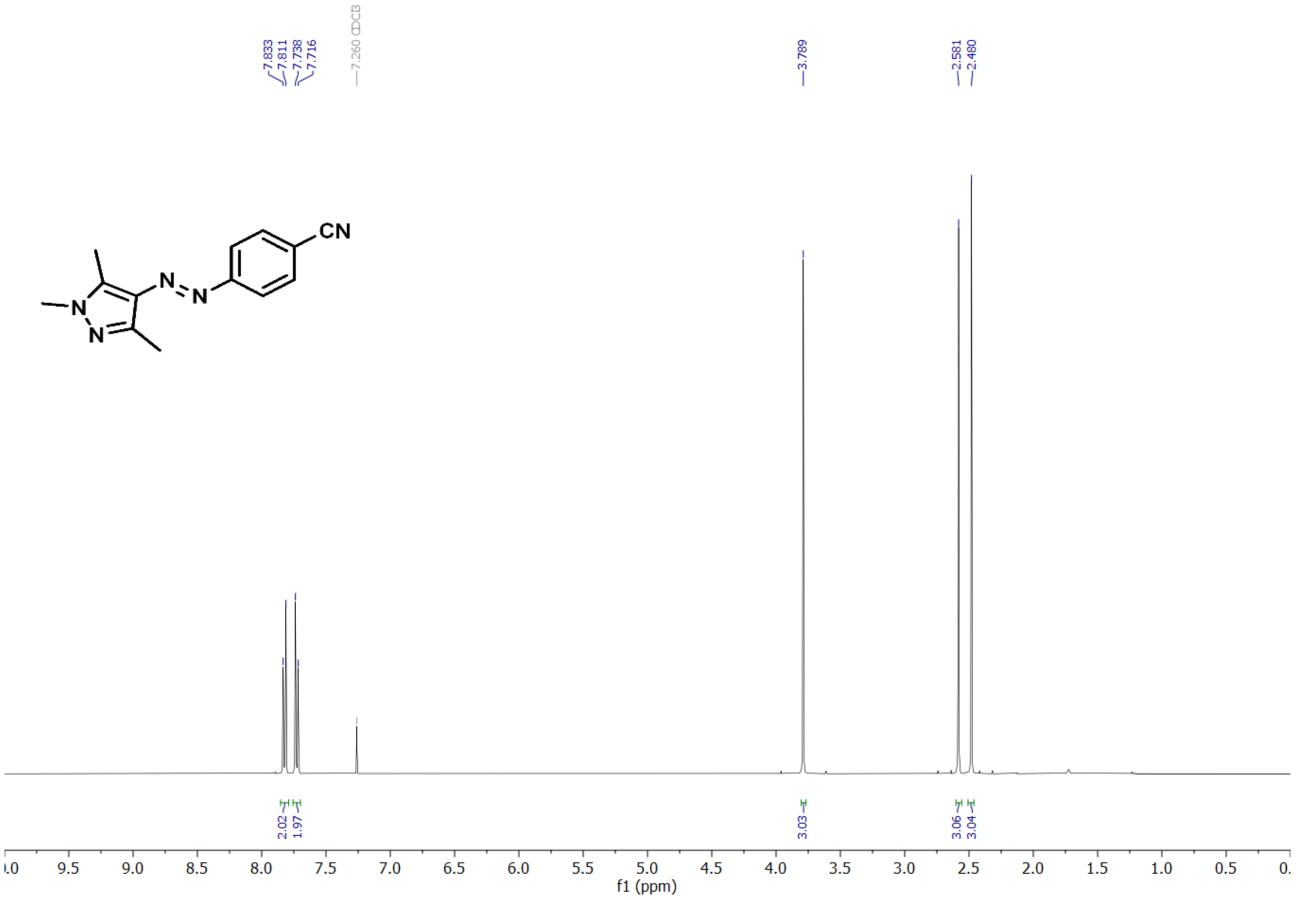

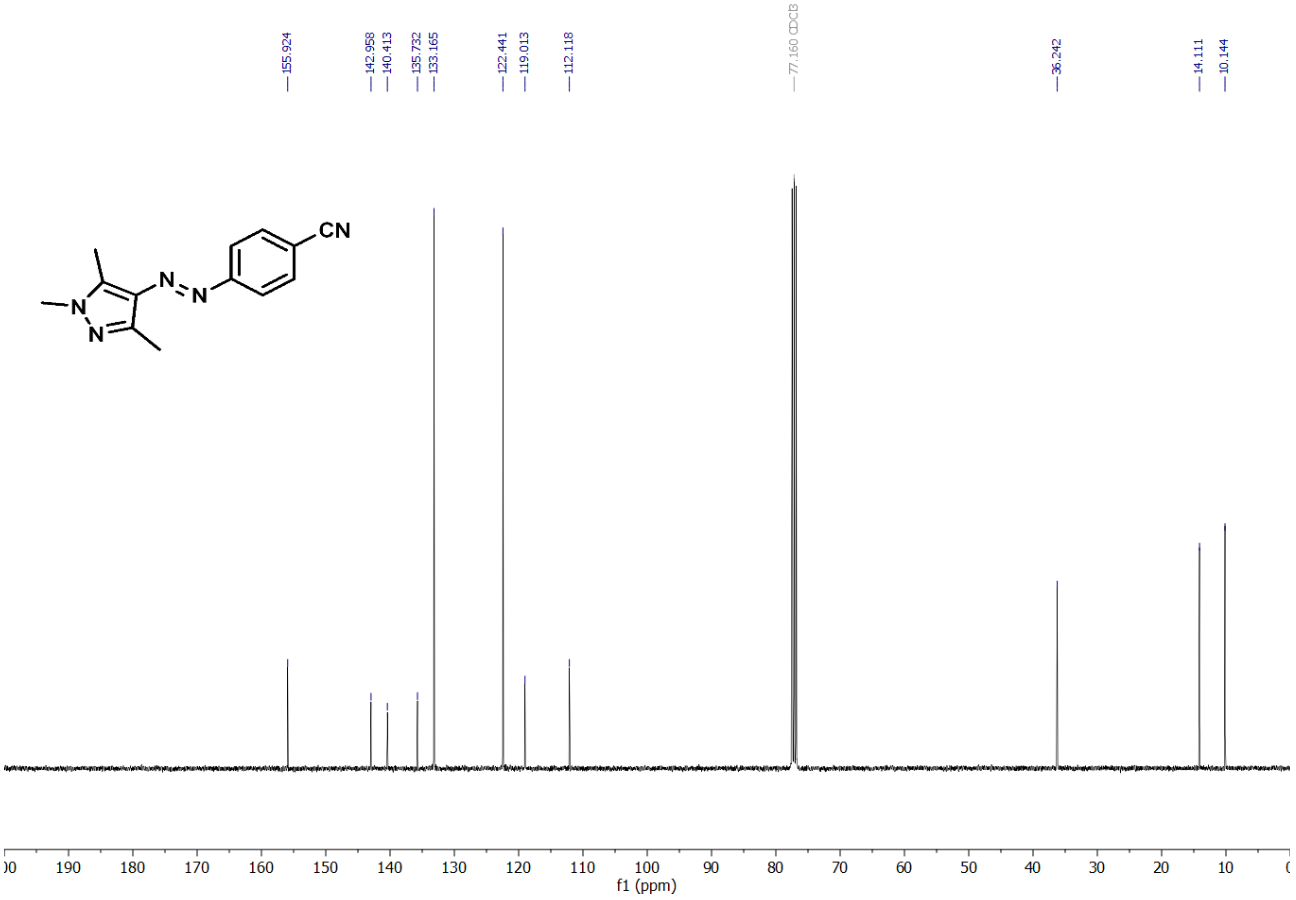

3-((4-(Diethylamino)phenyl)diazenyl)-4-hydroxypent-3-en-2-one (2l)

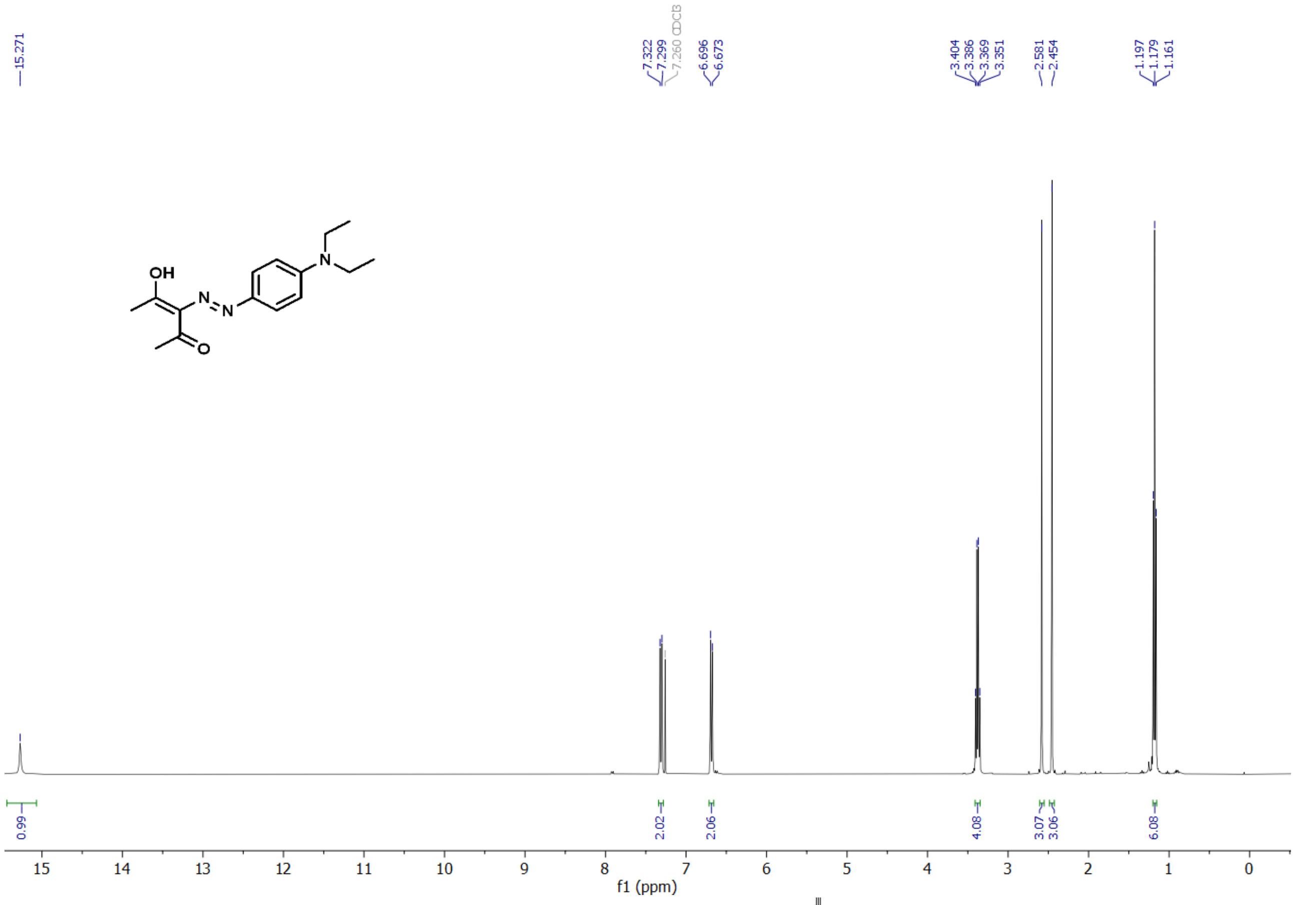

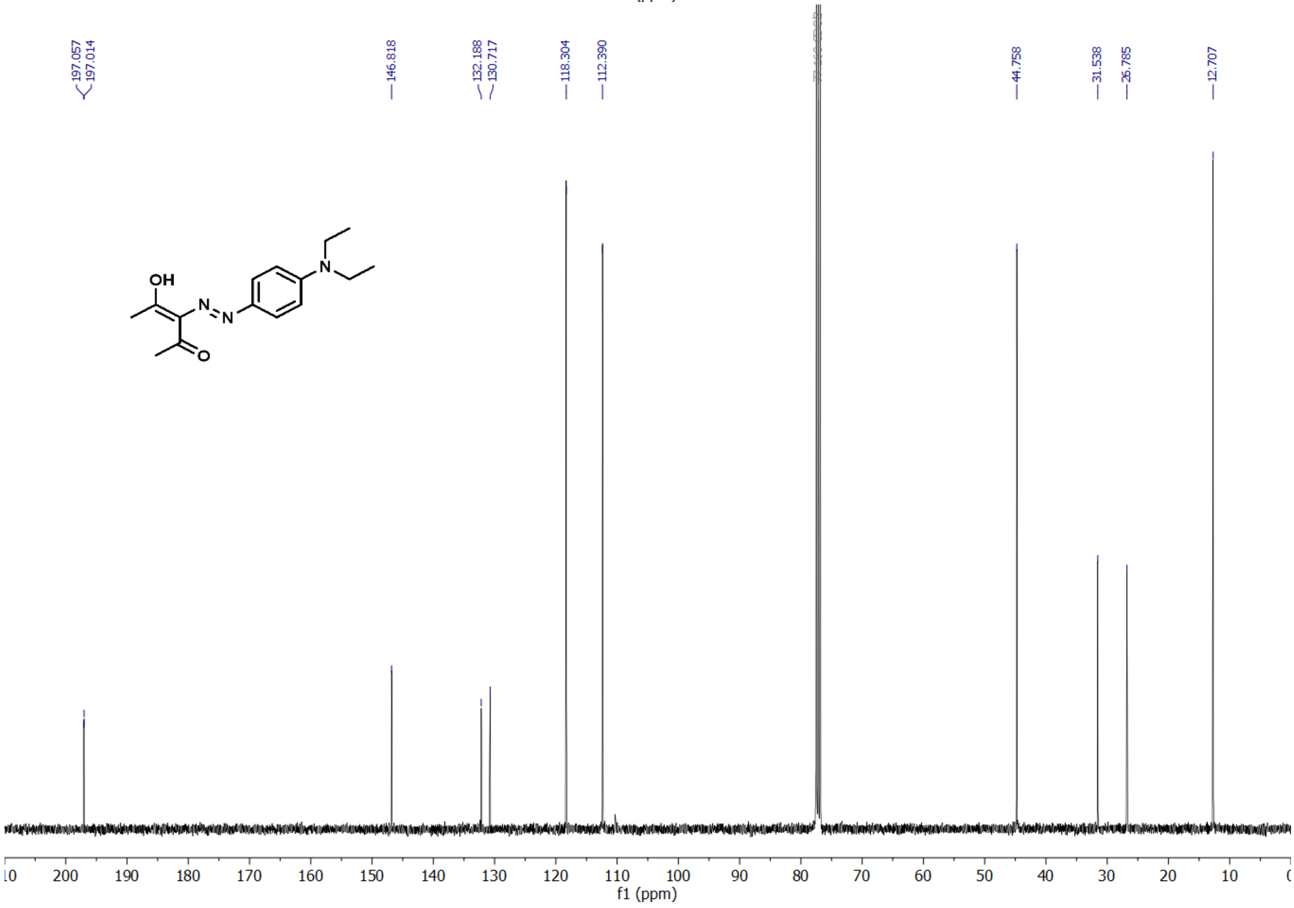

***N,N*-Diethyl-4-((1,3,5-trimethyl-1H-pyrazol-4-yl)diazenyl)aniline (3l)**

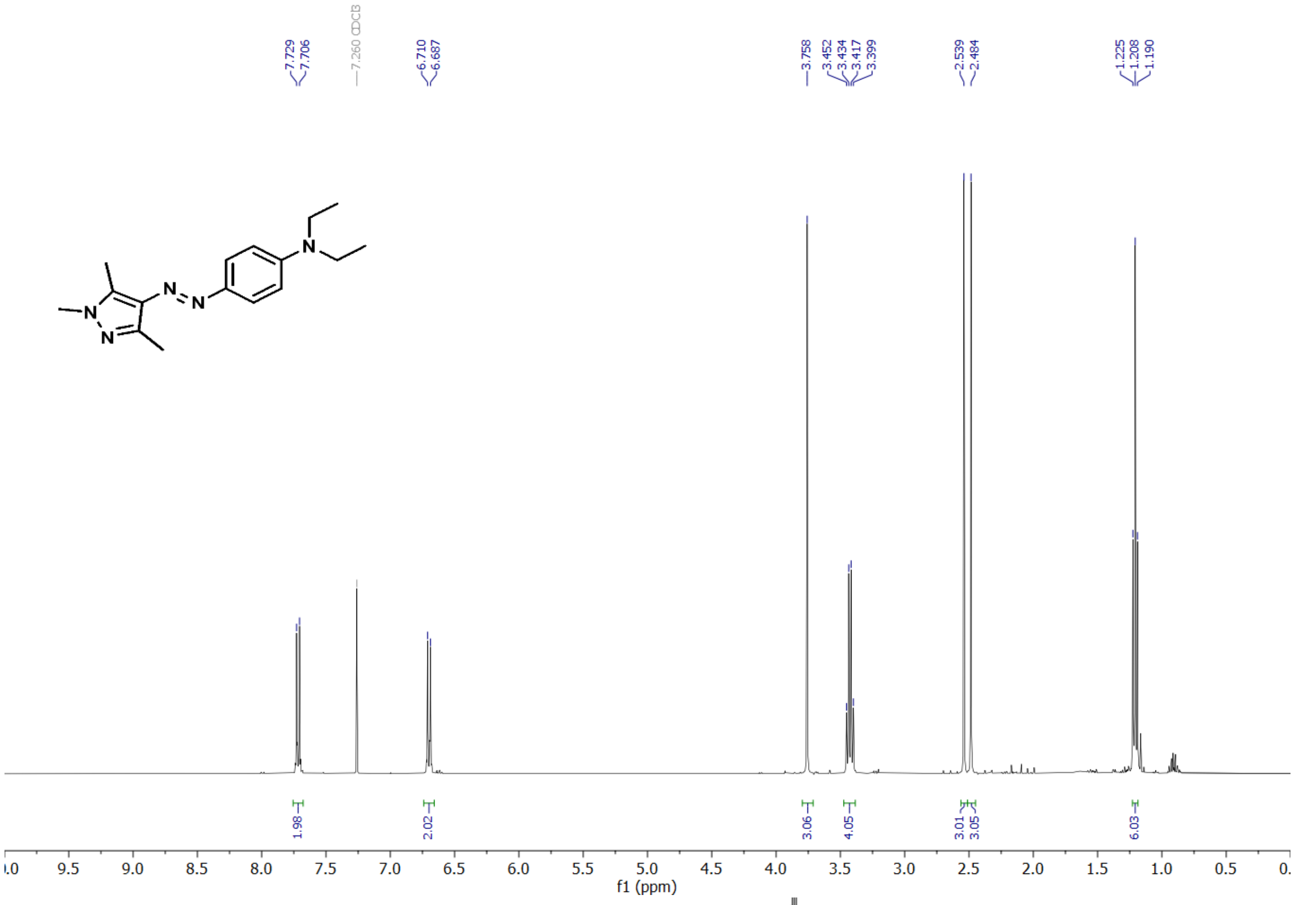

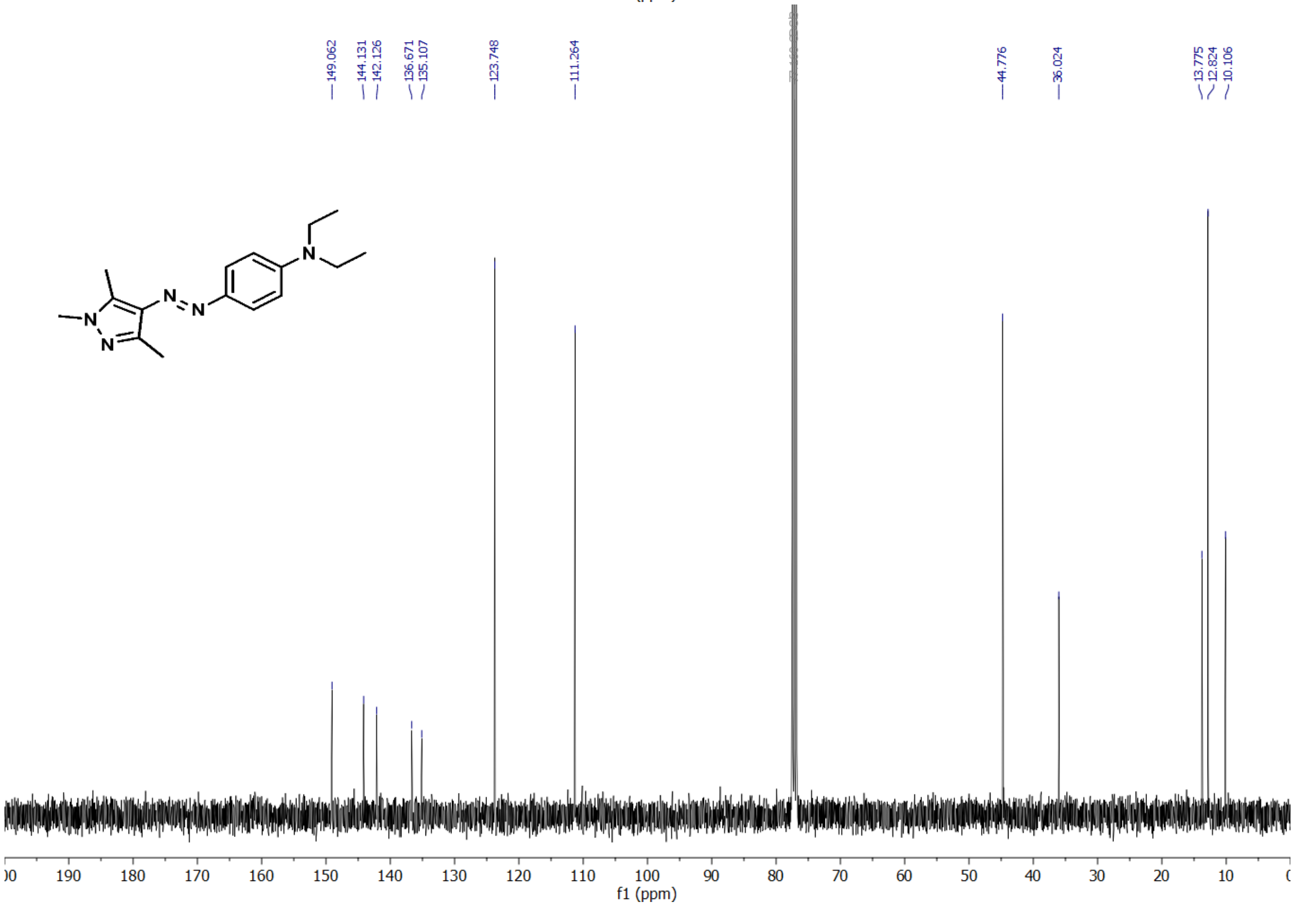

3-([1,1'-Biphenyl]-4-yldiazenyl)-4-hydroxypent-3-en-2-one (2m)

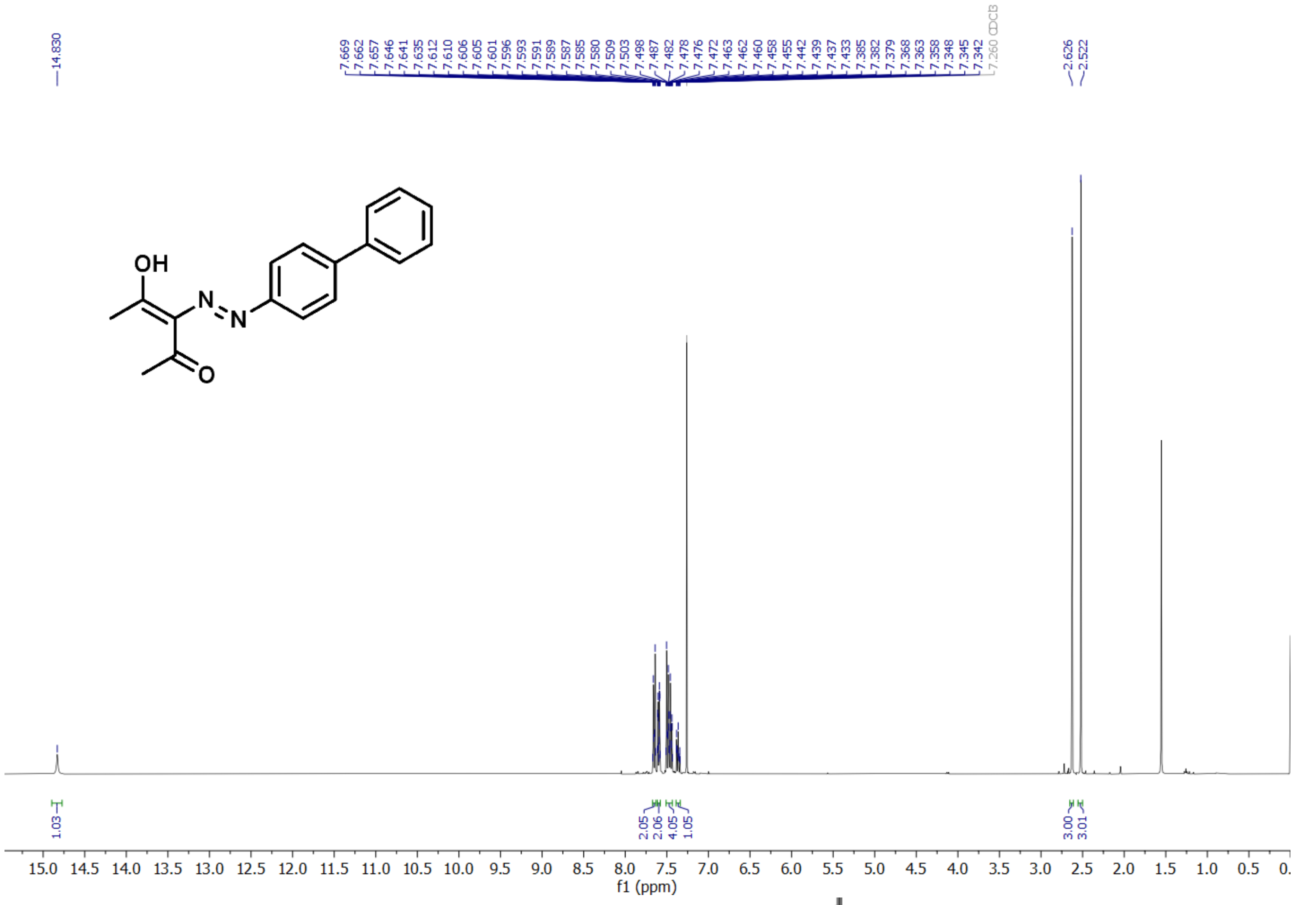

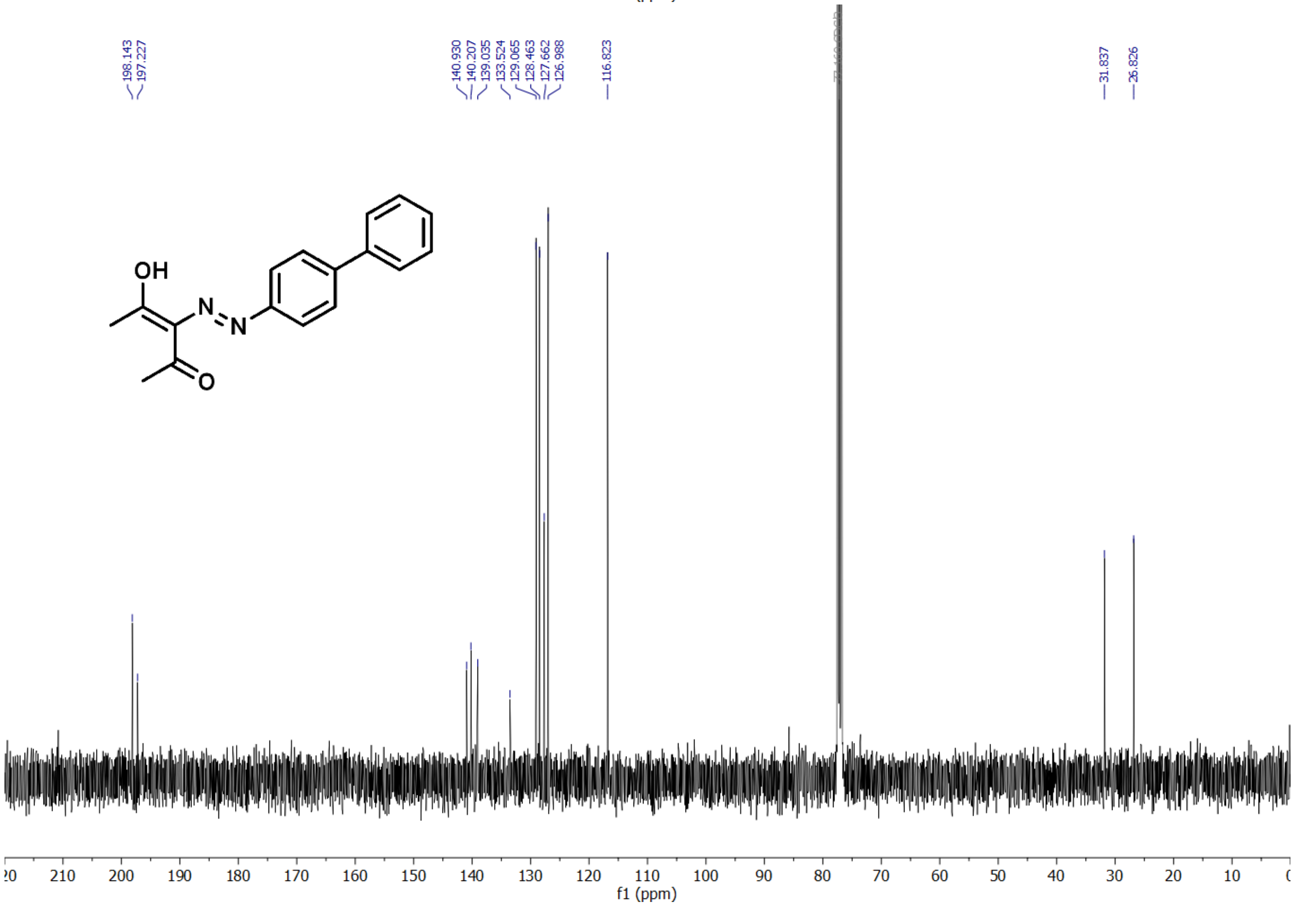

4-([1,1'-Biphenyl]-4-yldiazenyl)-1,3,5-trimethyl-1H-pyrazole (3m)

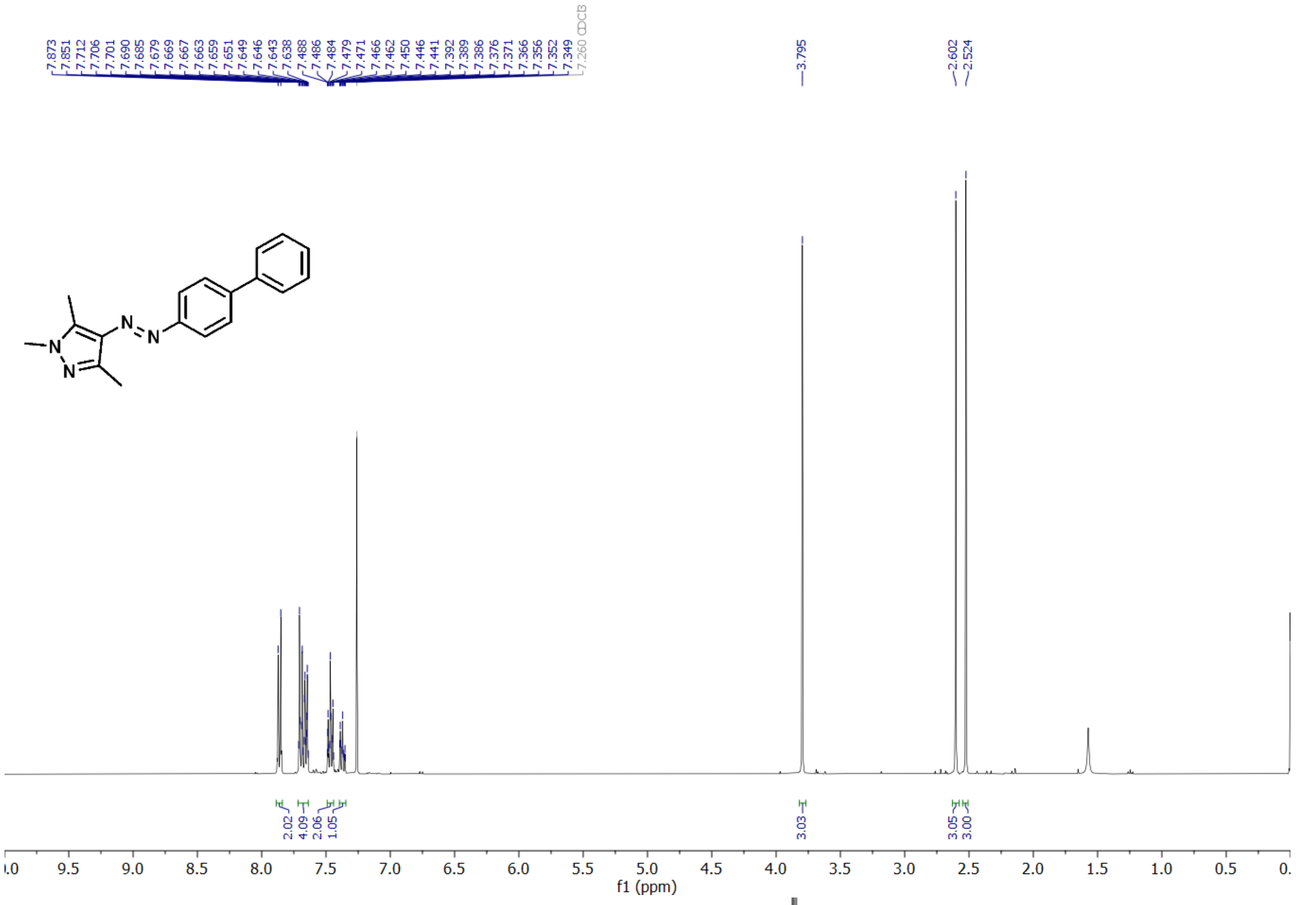

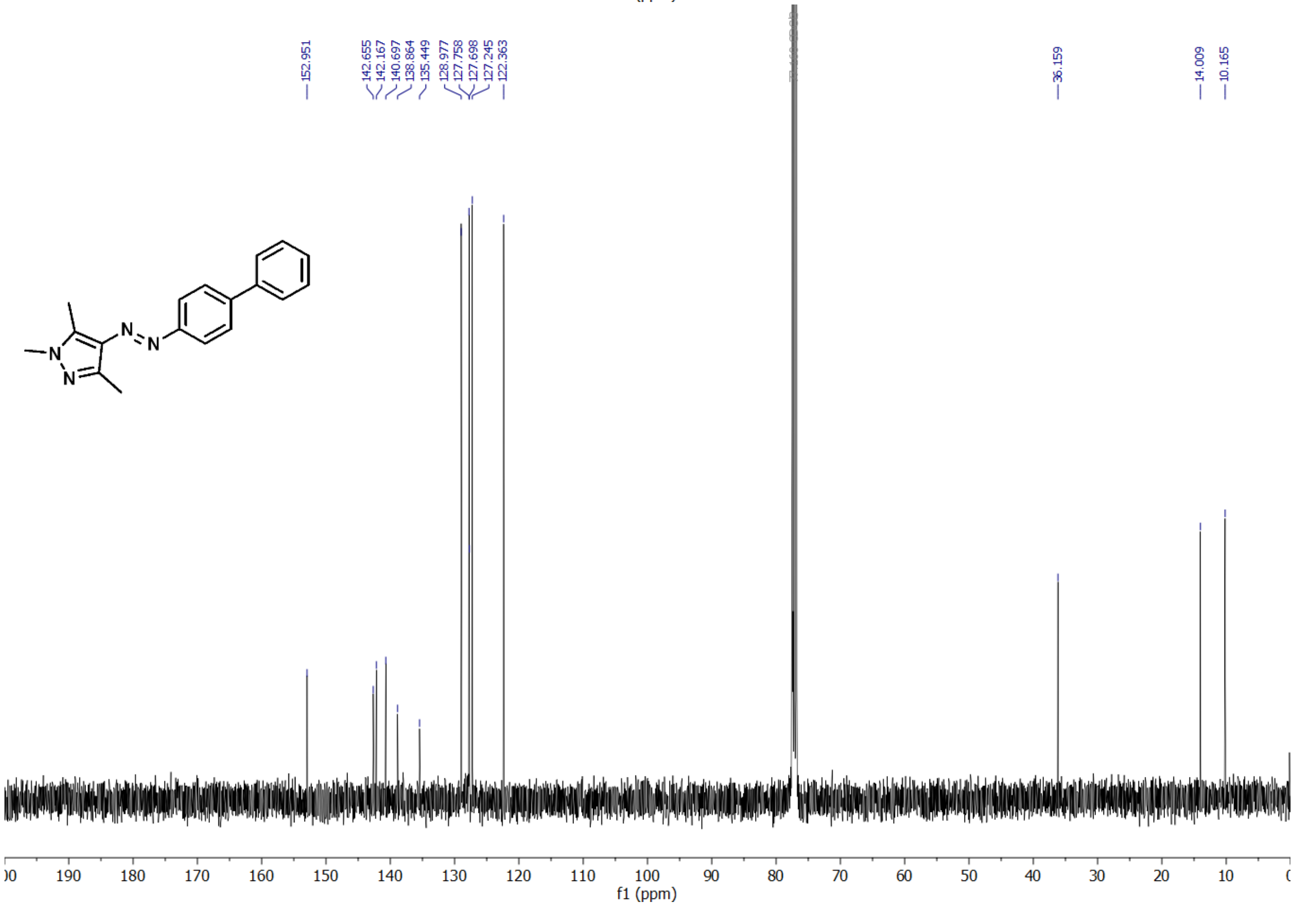

4-Hydroxy-3-(naphthalen-1-yldiazenyl)pent-3-en-2-one (2n)

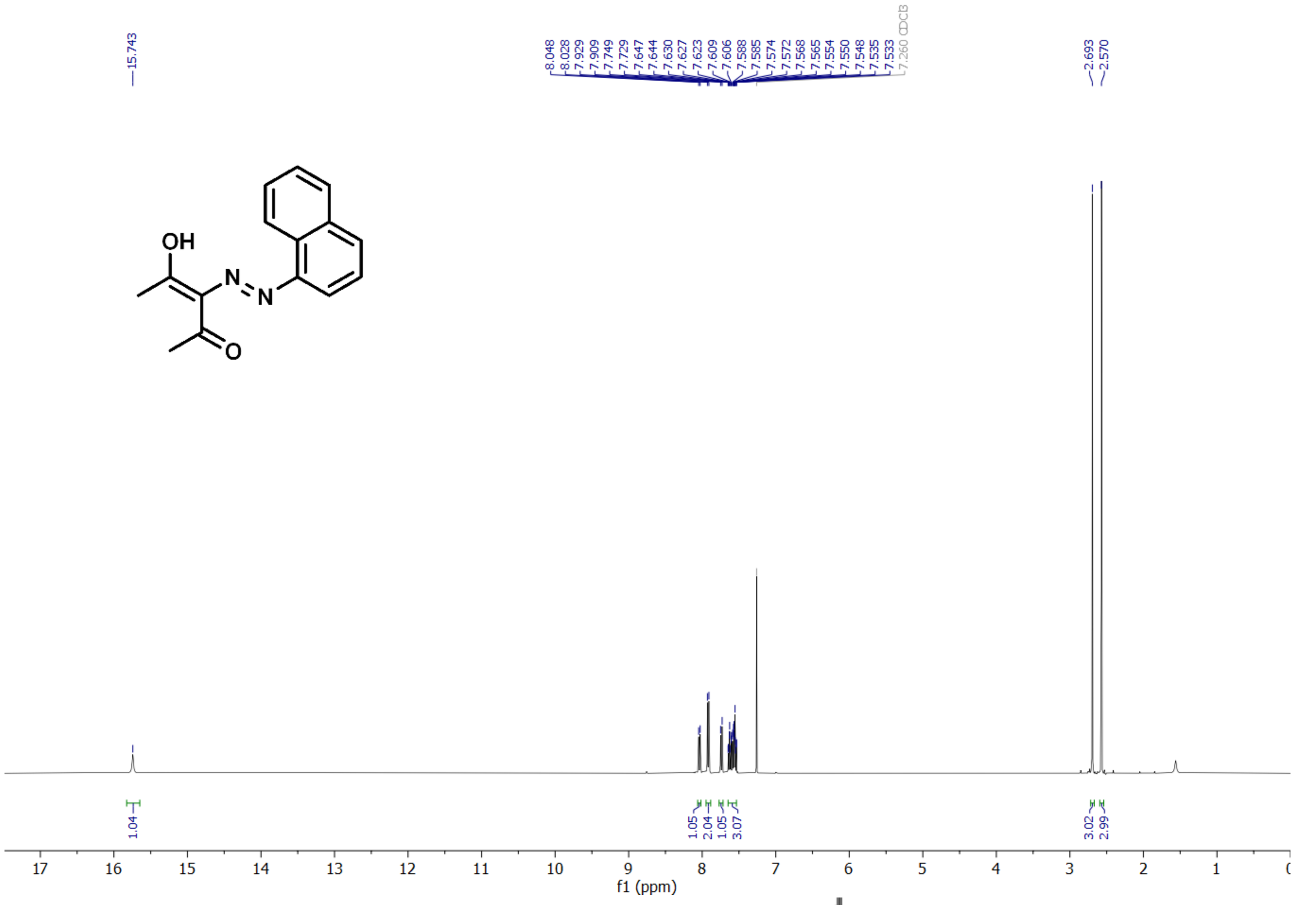

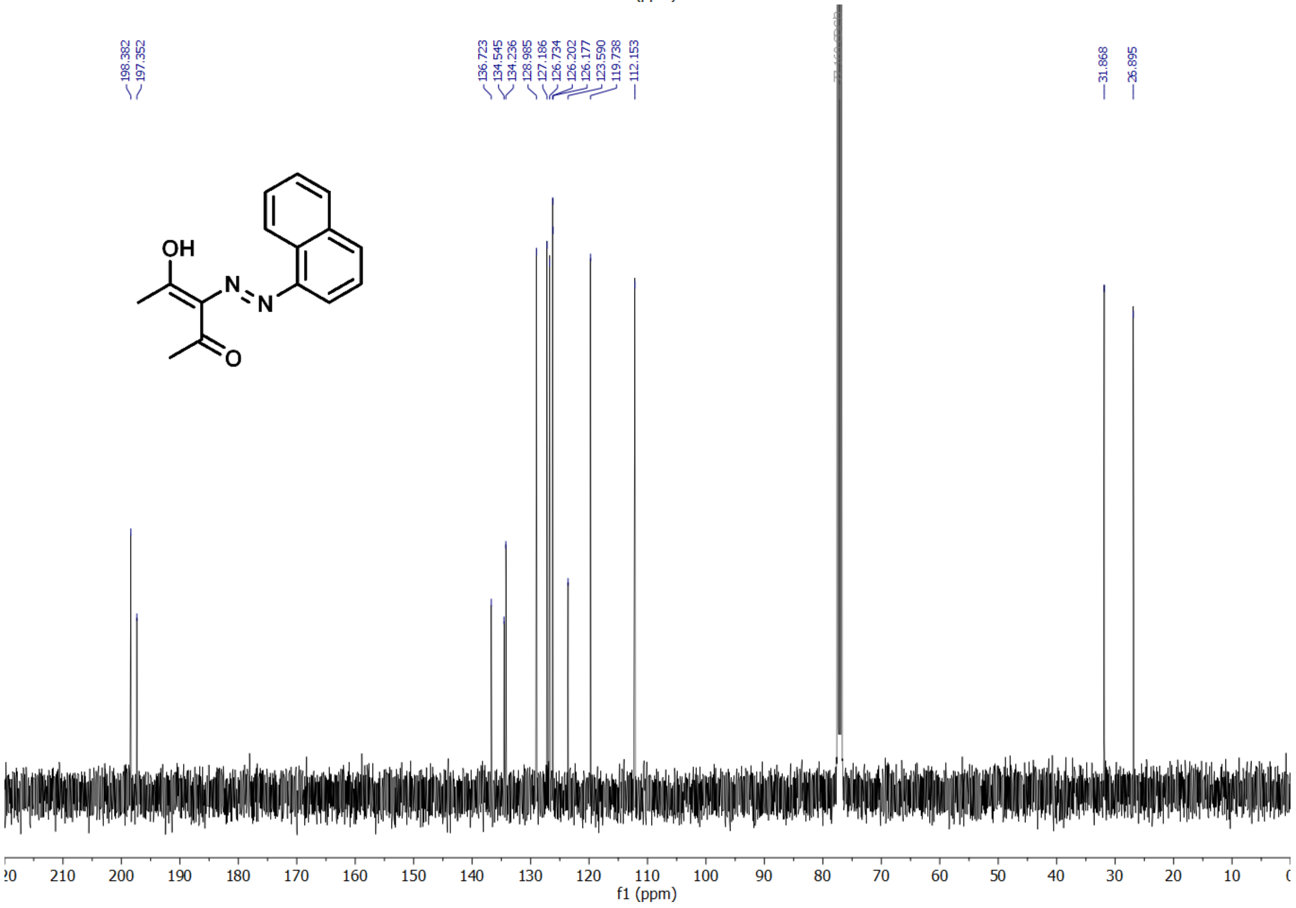

1,3,5-Trimethyl-4-(naphthalen-1-yldiazenyl)-1H-pyrazole (3n)

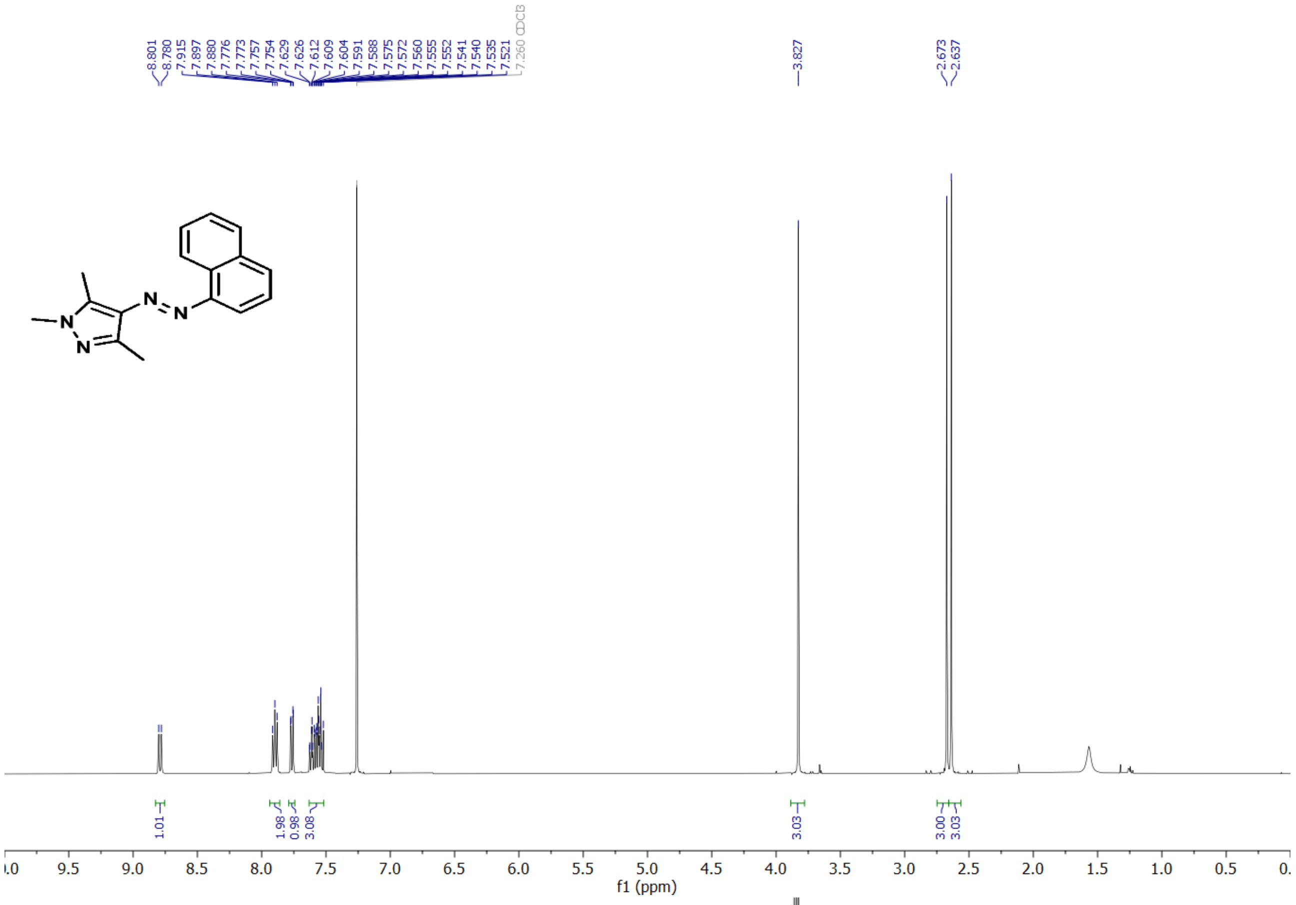

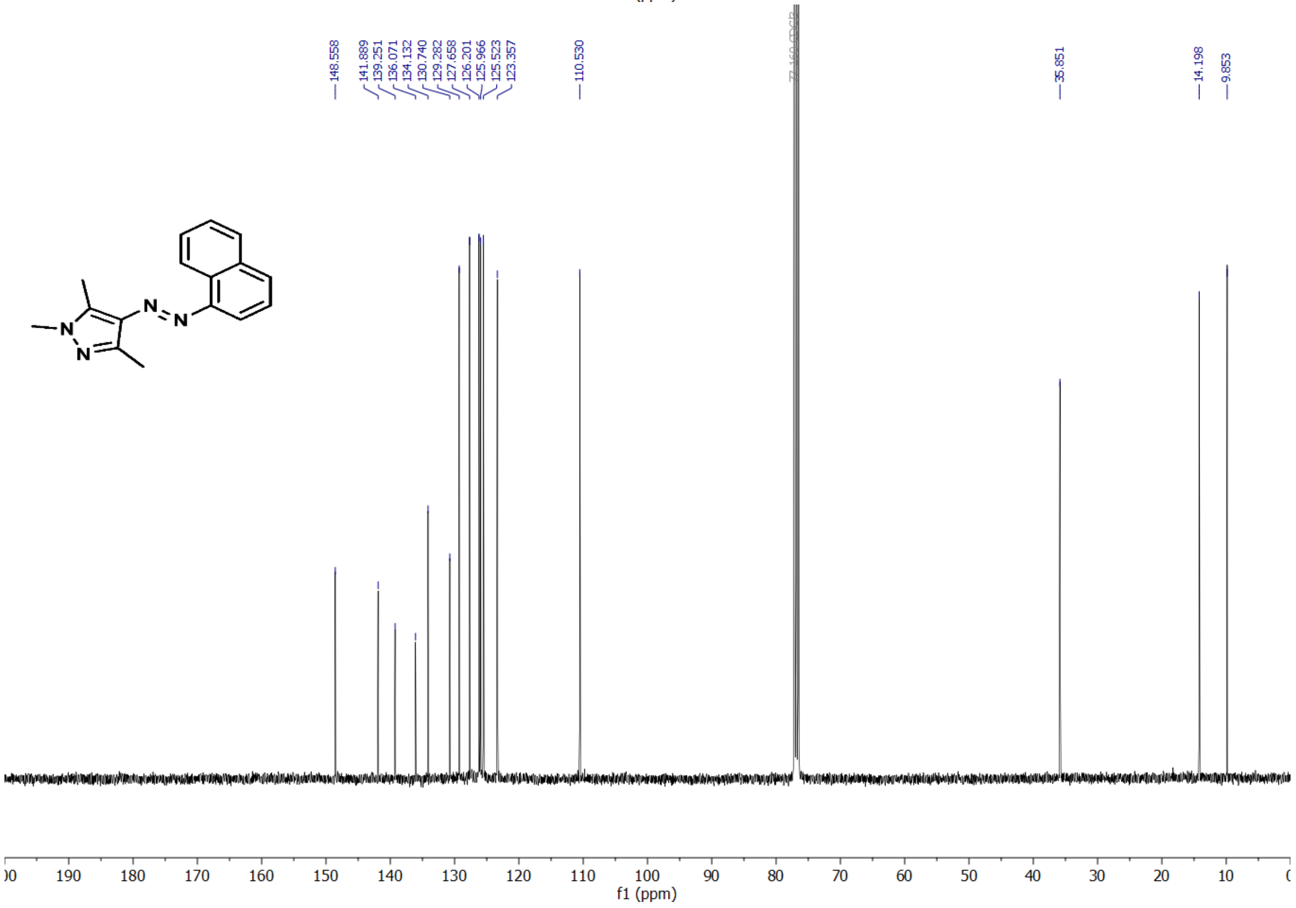

4-Hydroxy-3-(naphthalen-2-yldiazenyl)pent-3-en-2-one (2o)

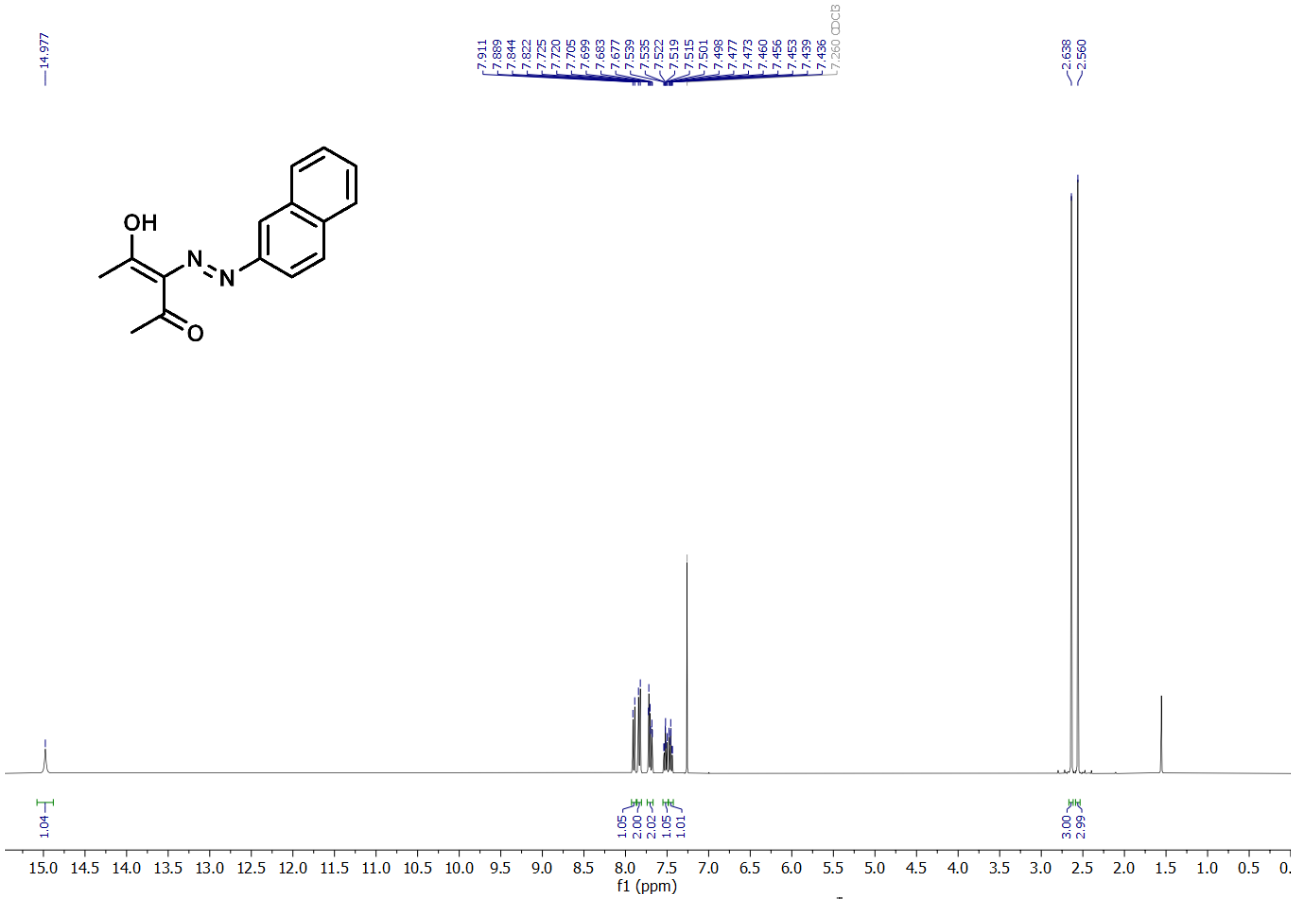

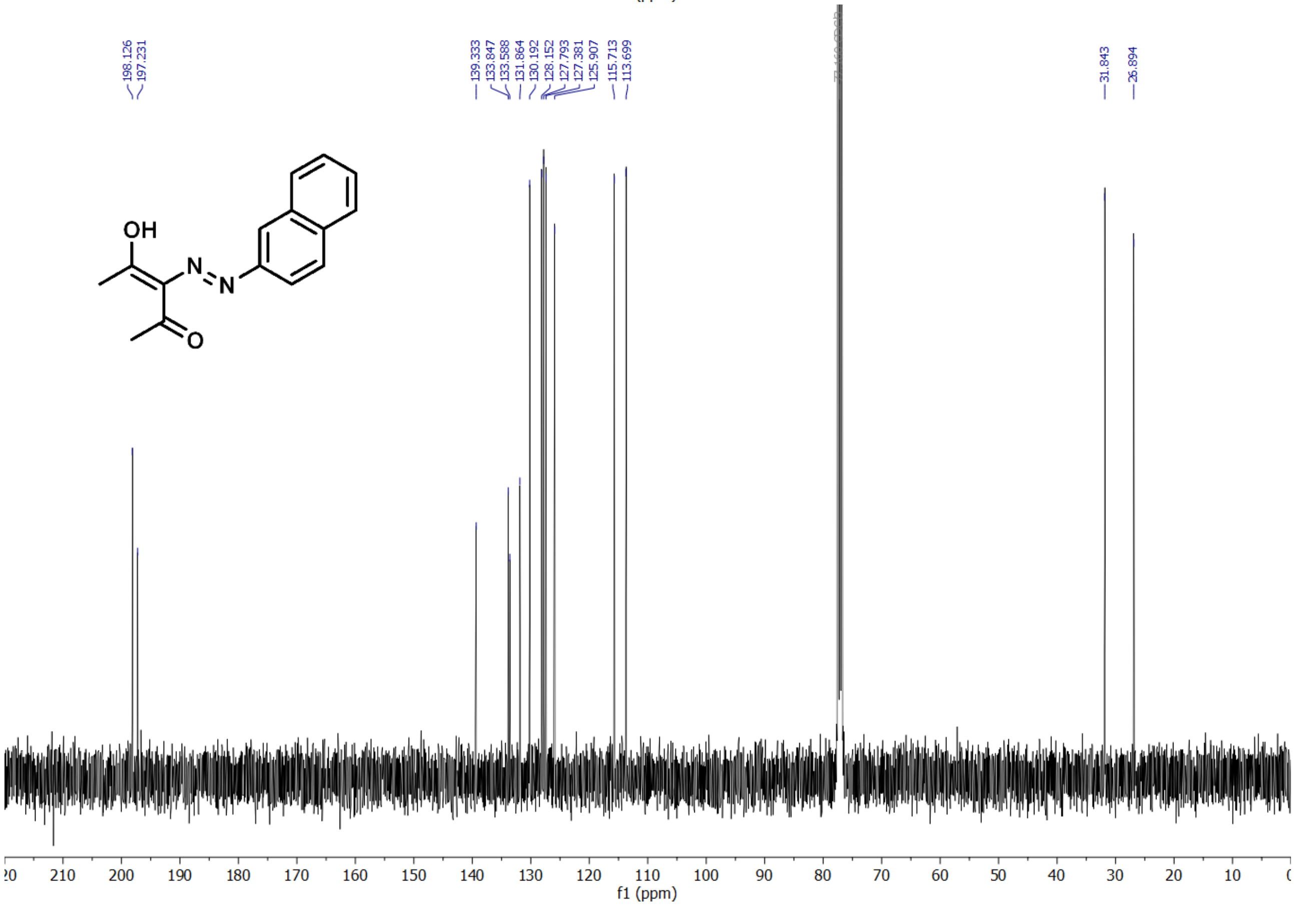

1,3,5-Trimethyl-4-(naphthalen-2-yldiazenyl)-1H-pyrazole (3o)

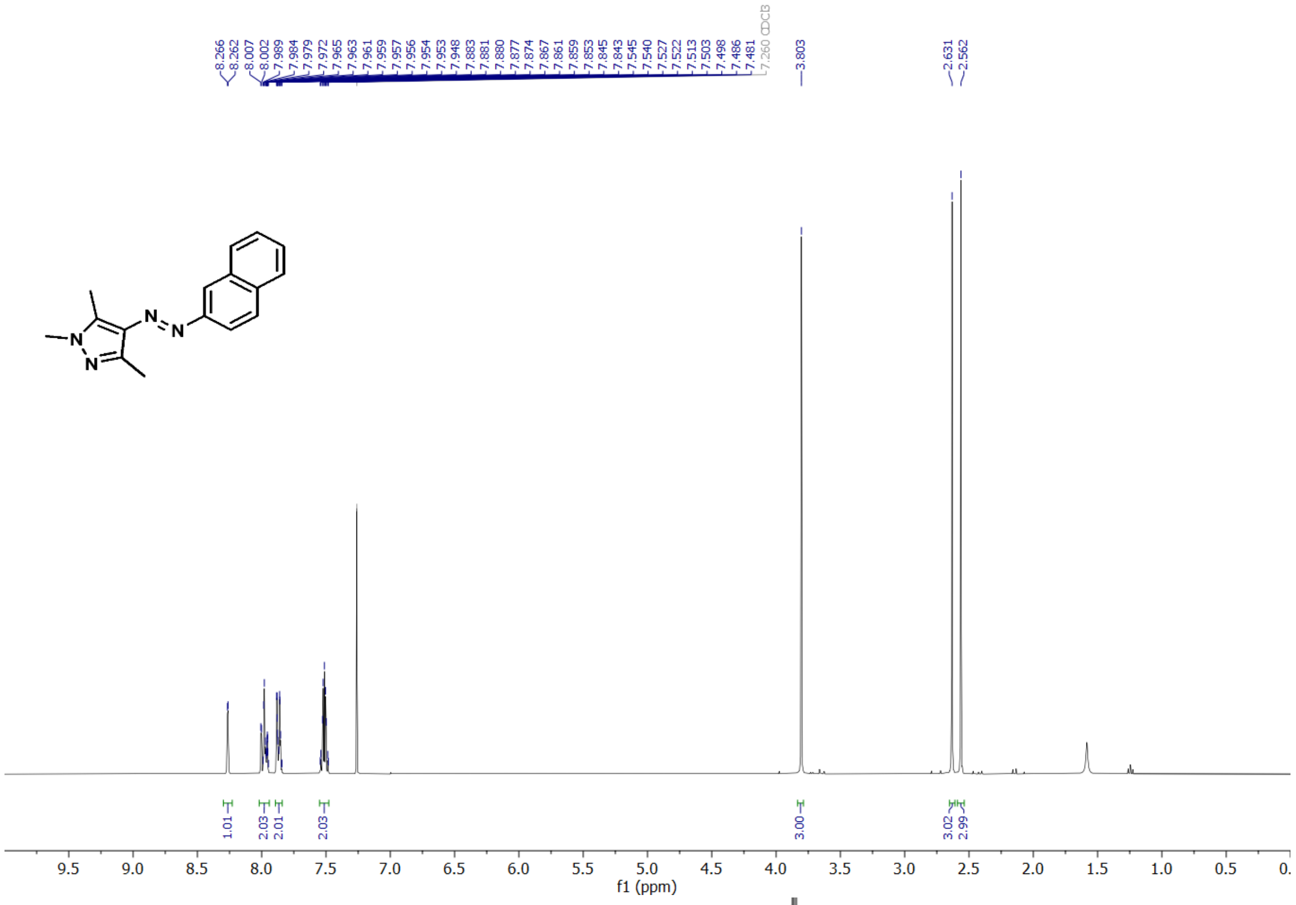

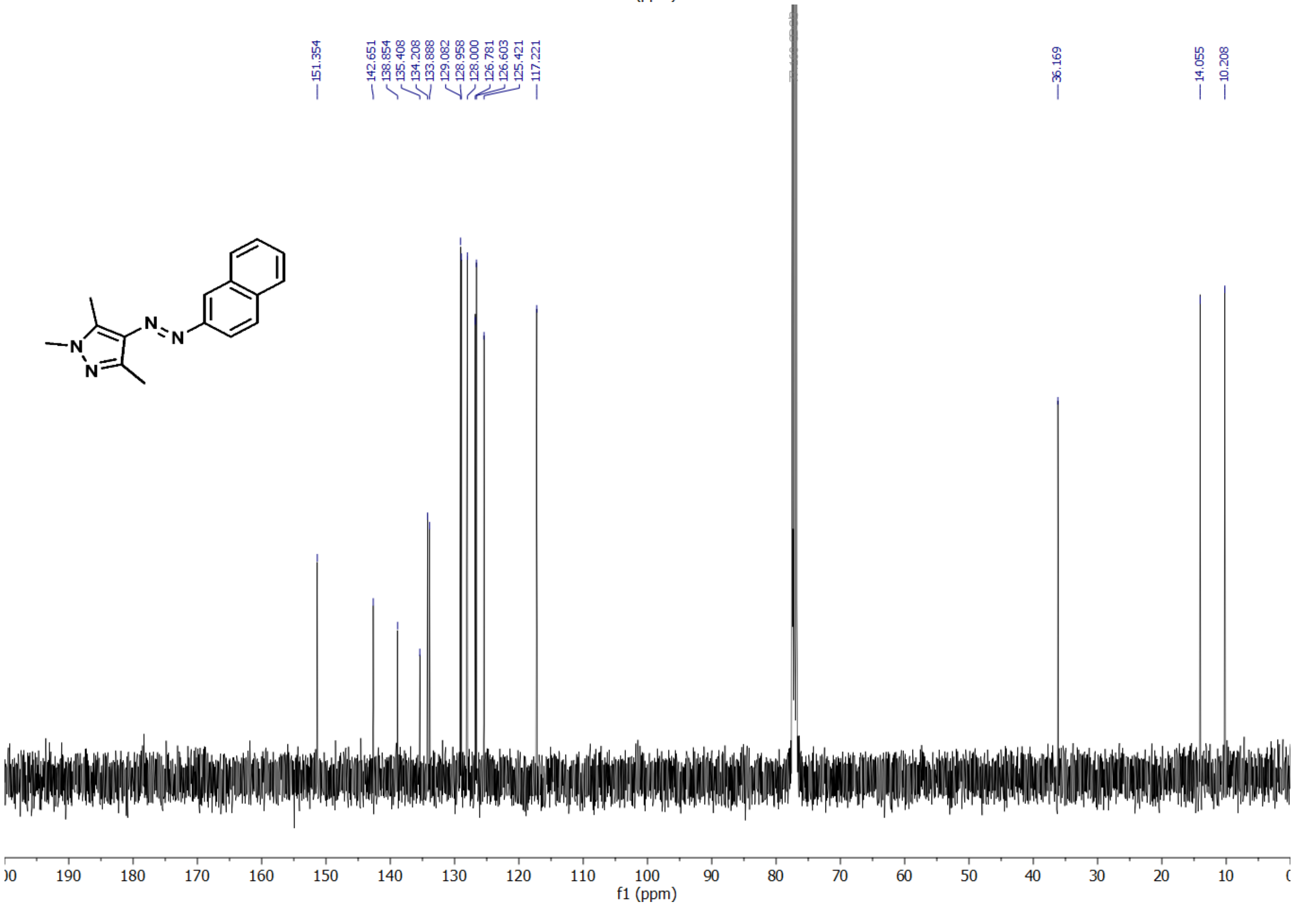

4-Ethynylaniline (1p)

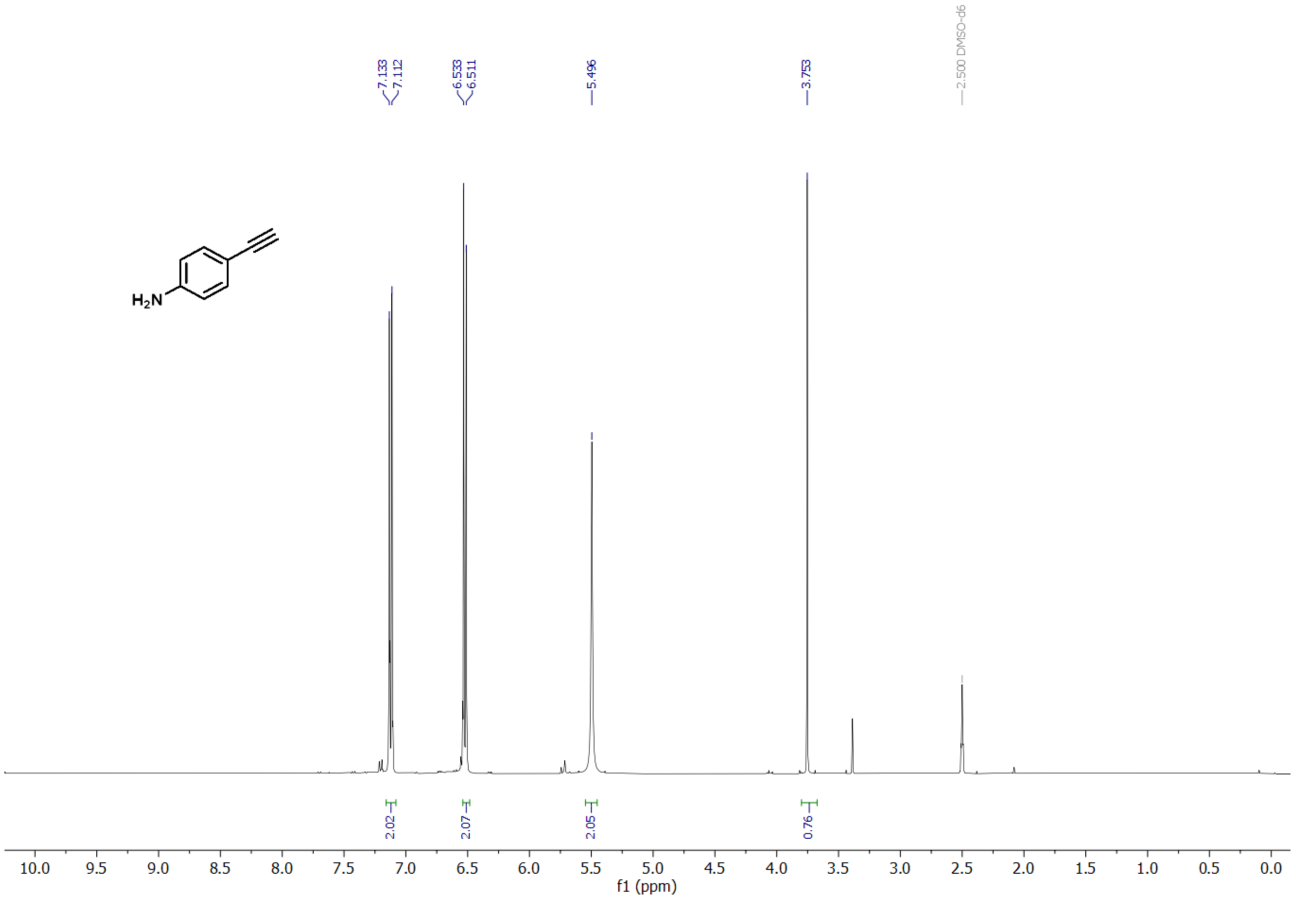

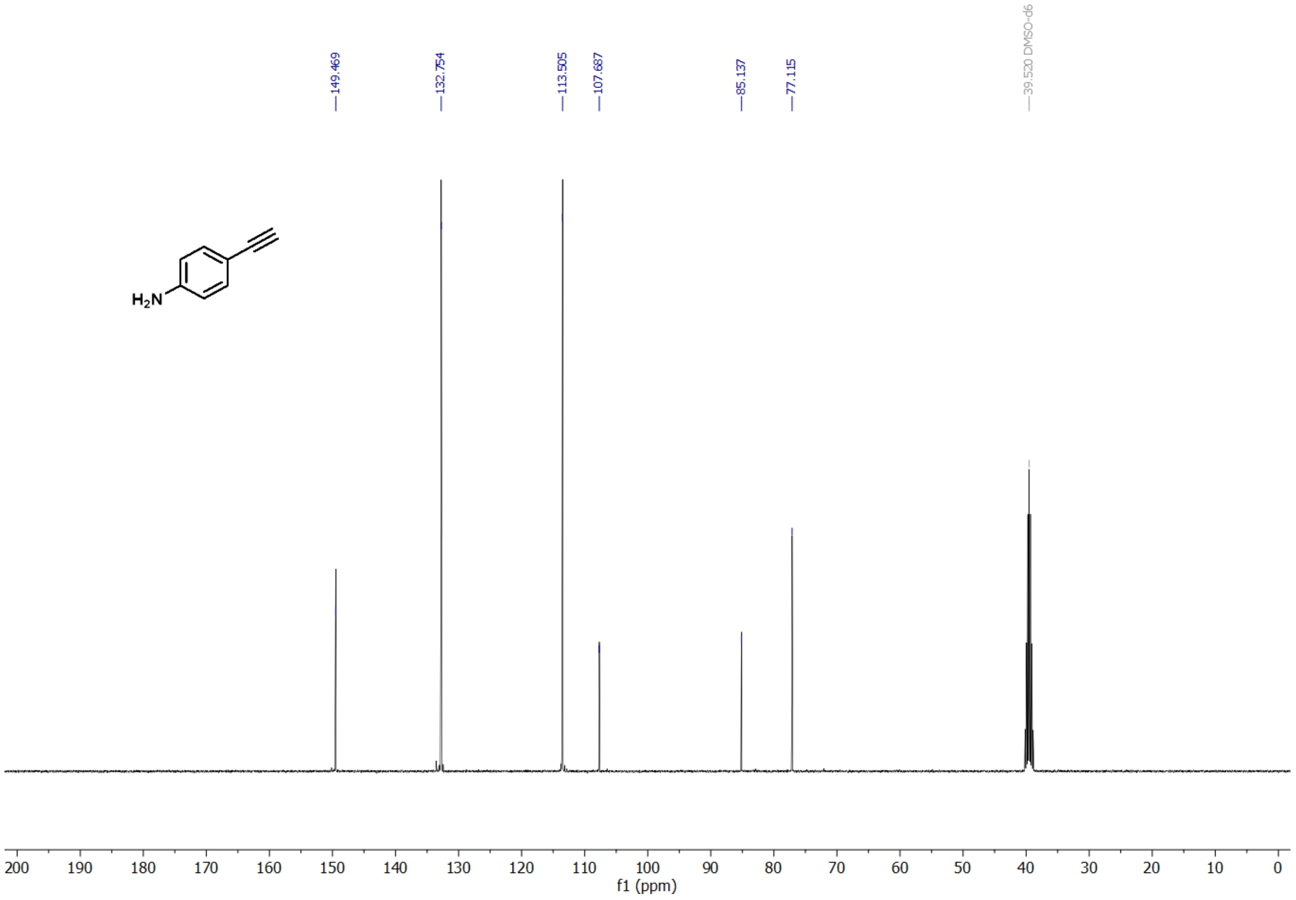

3-((4-Ethynylphenyl)diazenyl)-4-hydroxypent-3-en-2-one (2p)

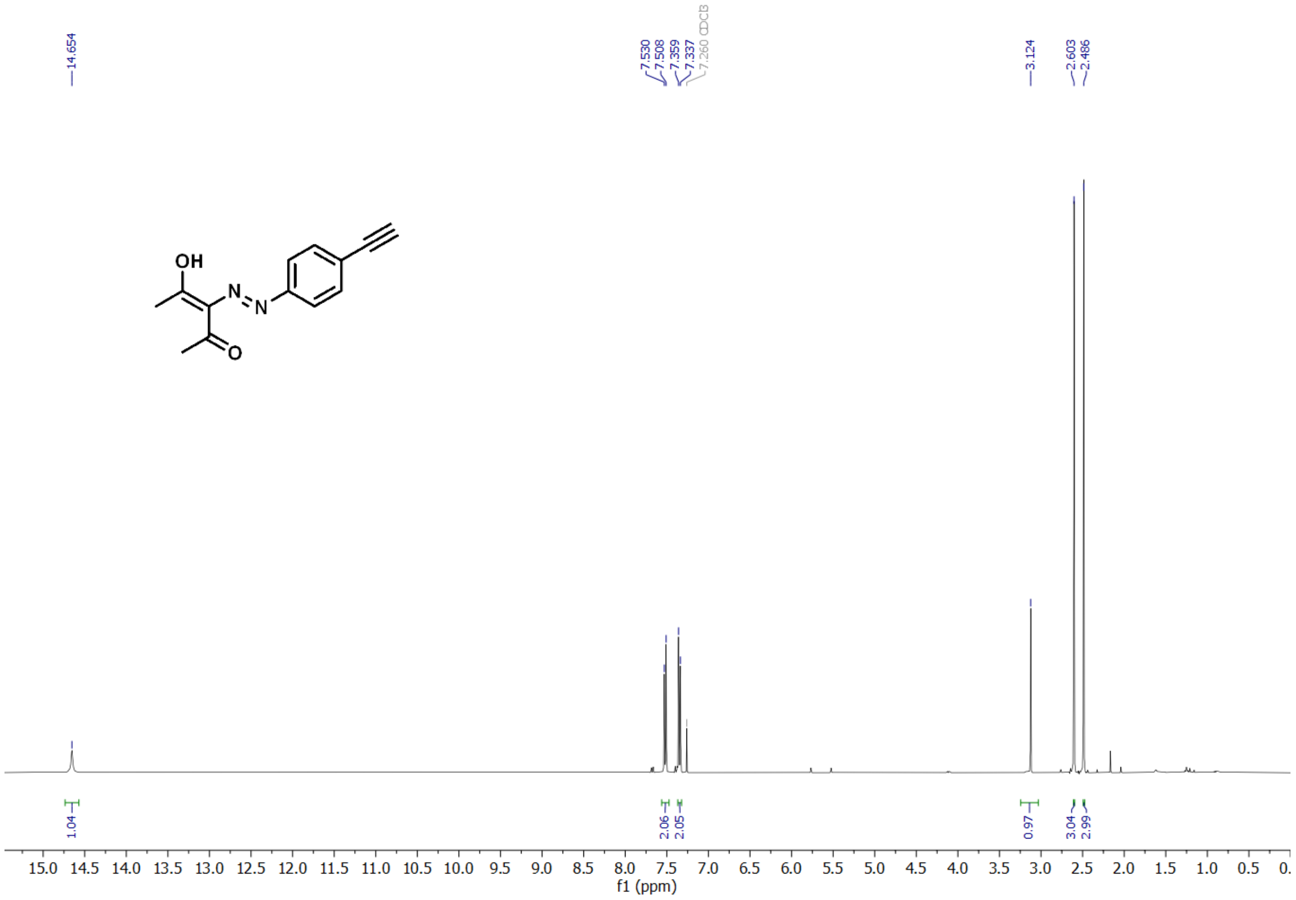

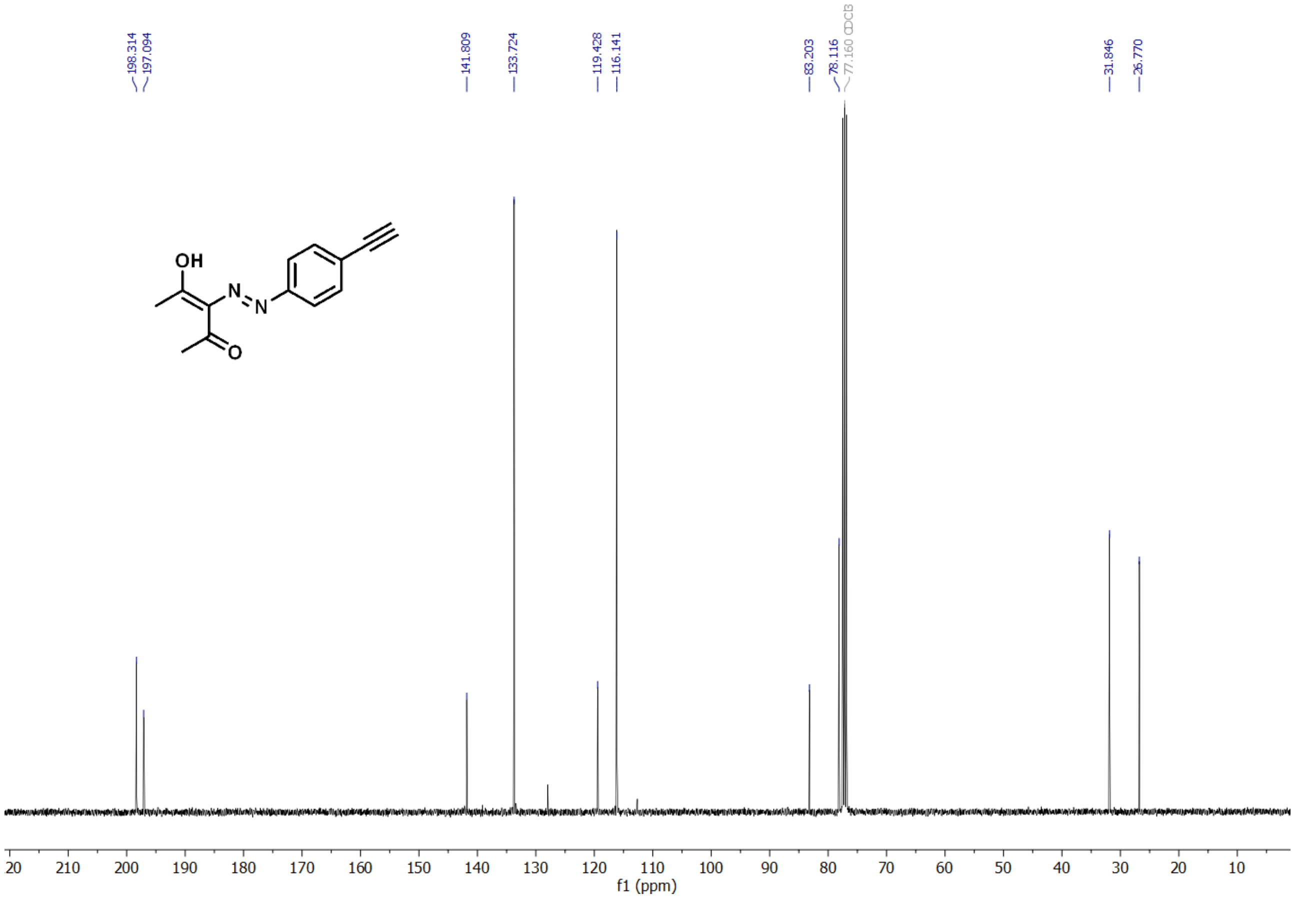

4-((4-Ethynylphenyl)diazenyl)-1,3,5-trimethyl-1H-pyrazole (3p)

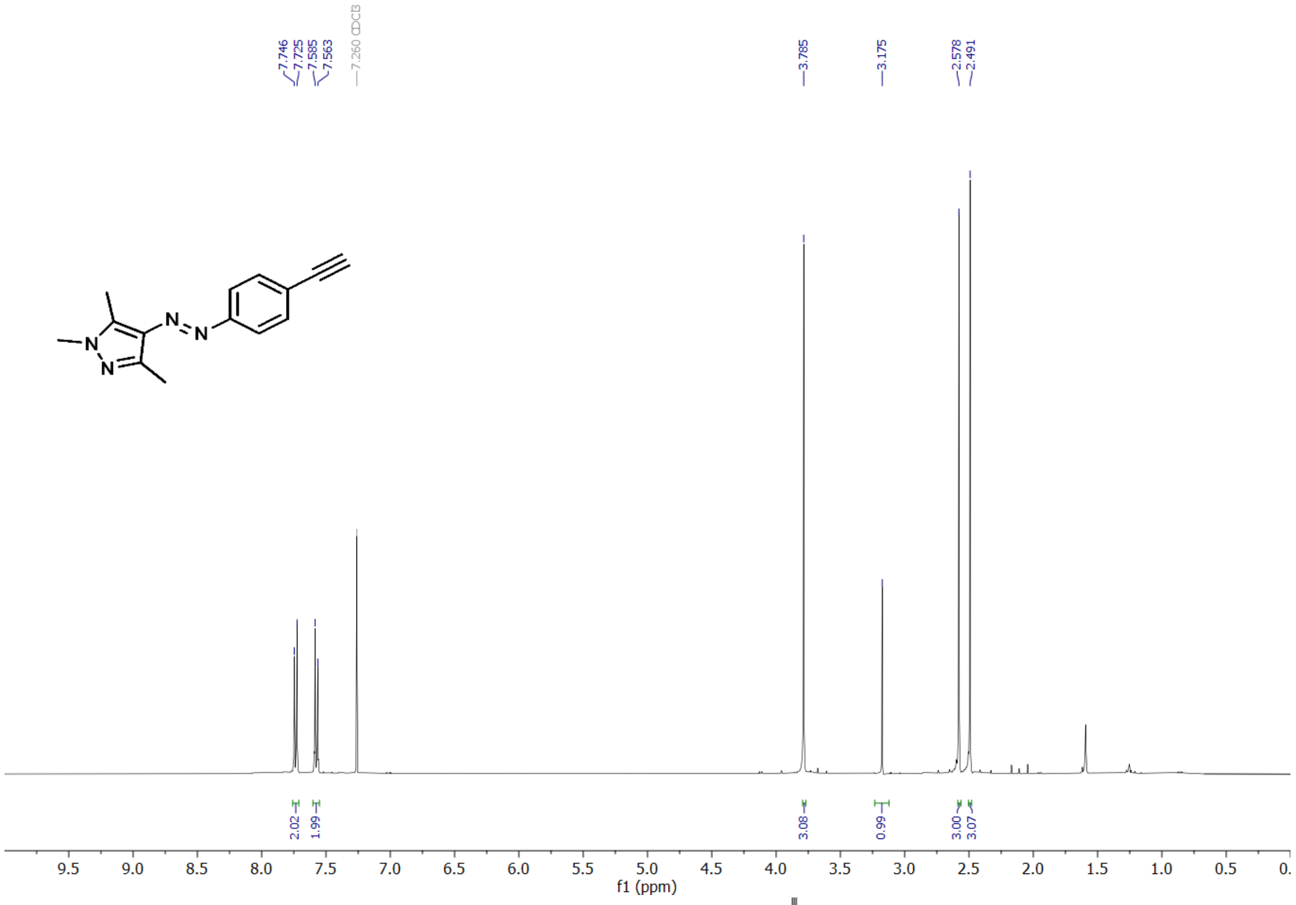

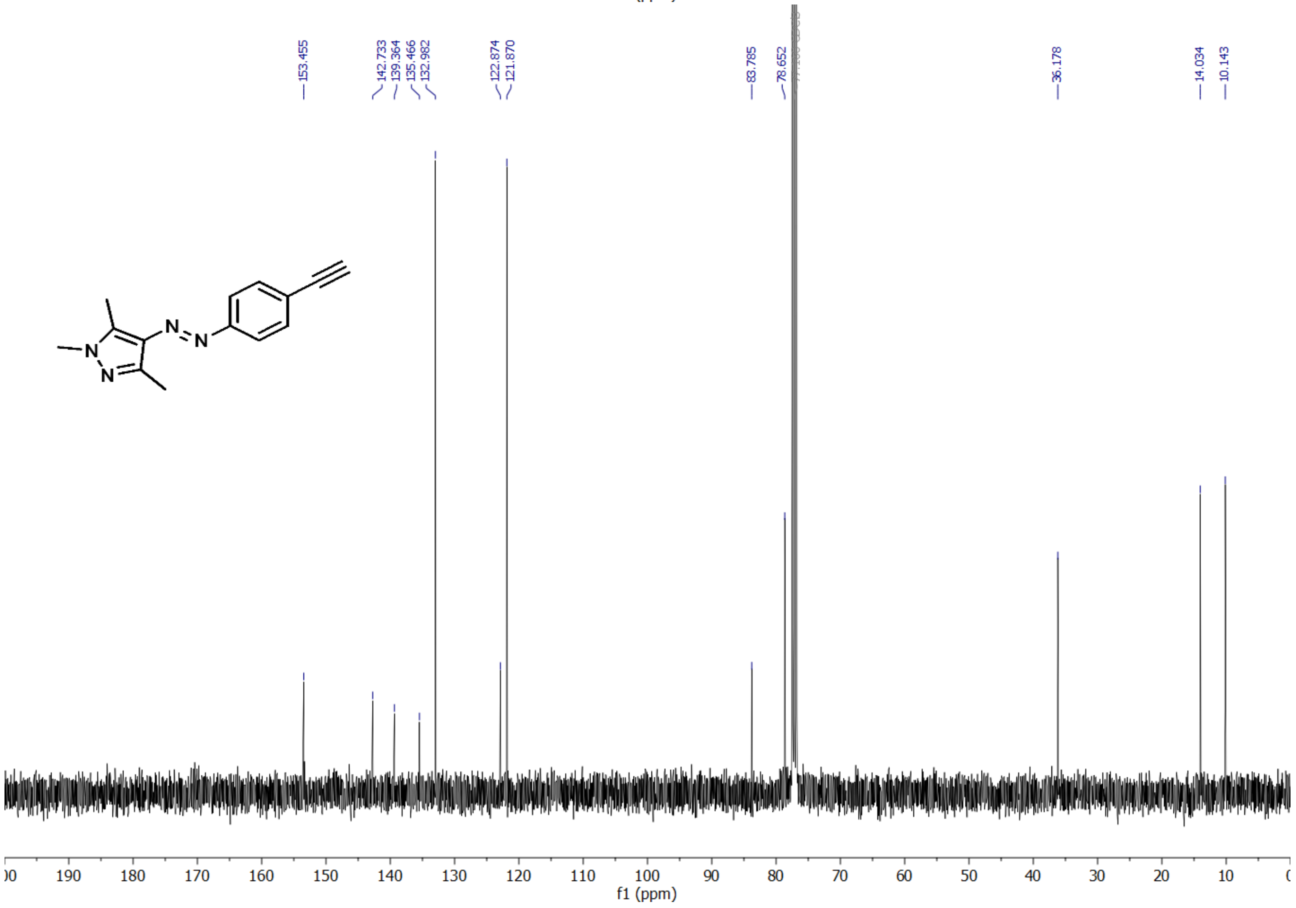

3-((4-Acetylphenyl)diazenyl)-4-hydroxypent-3-en-2-one (2q)

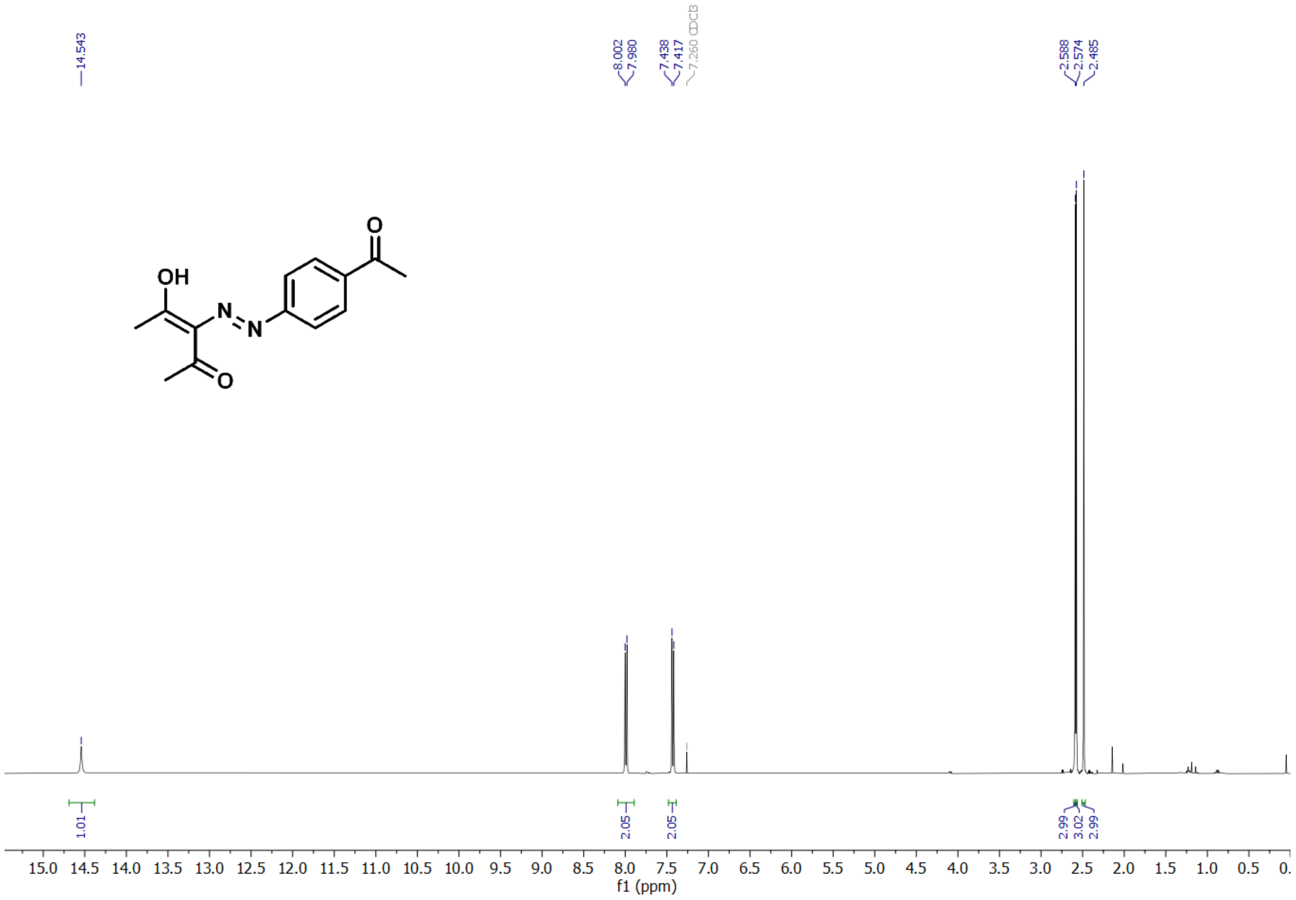

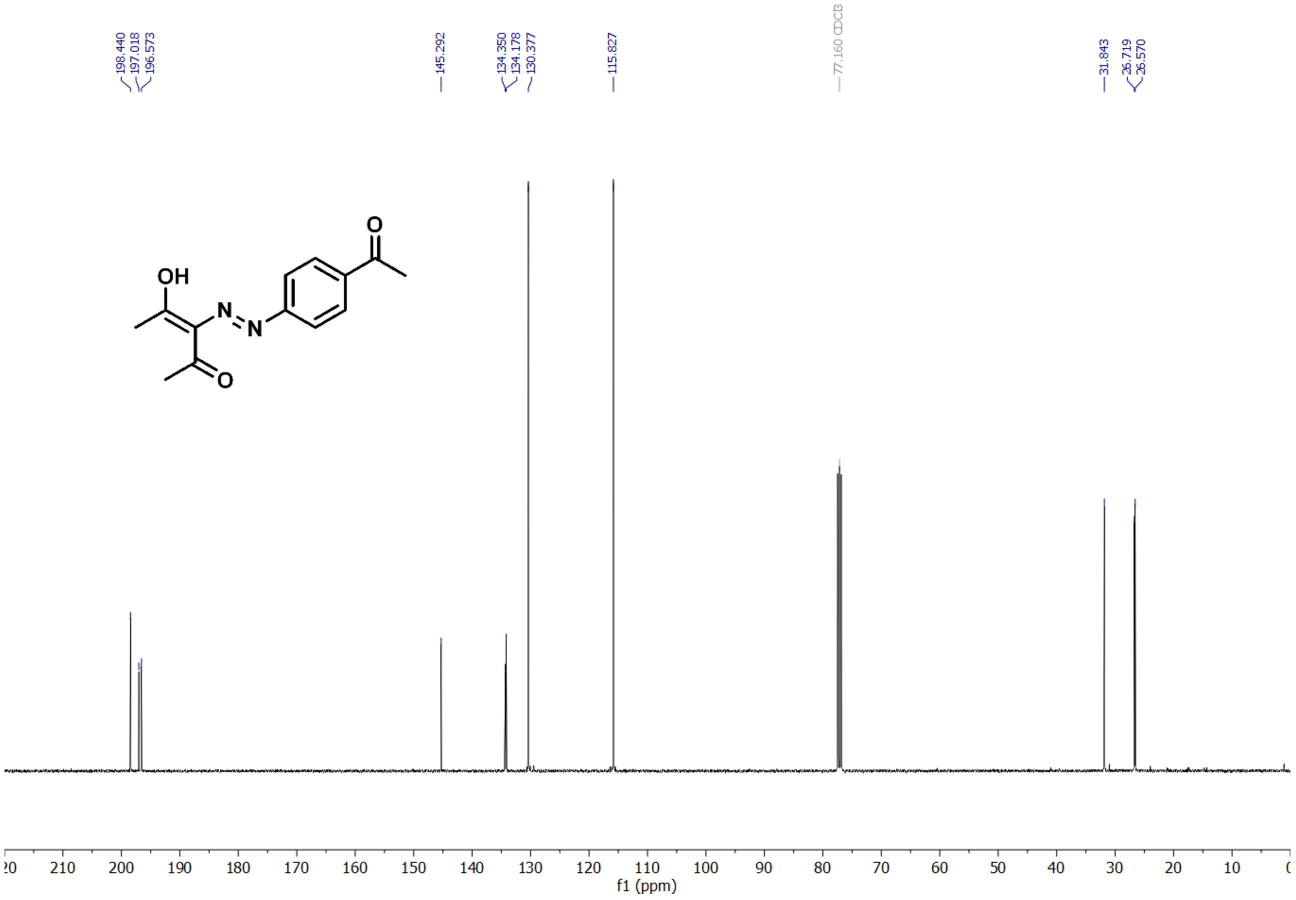

1-(4-((1,3,5-Trimethyl-1H-pyrazol-4-yl)diazenyl)phenyl)ethan-1-one (3q)

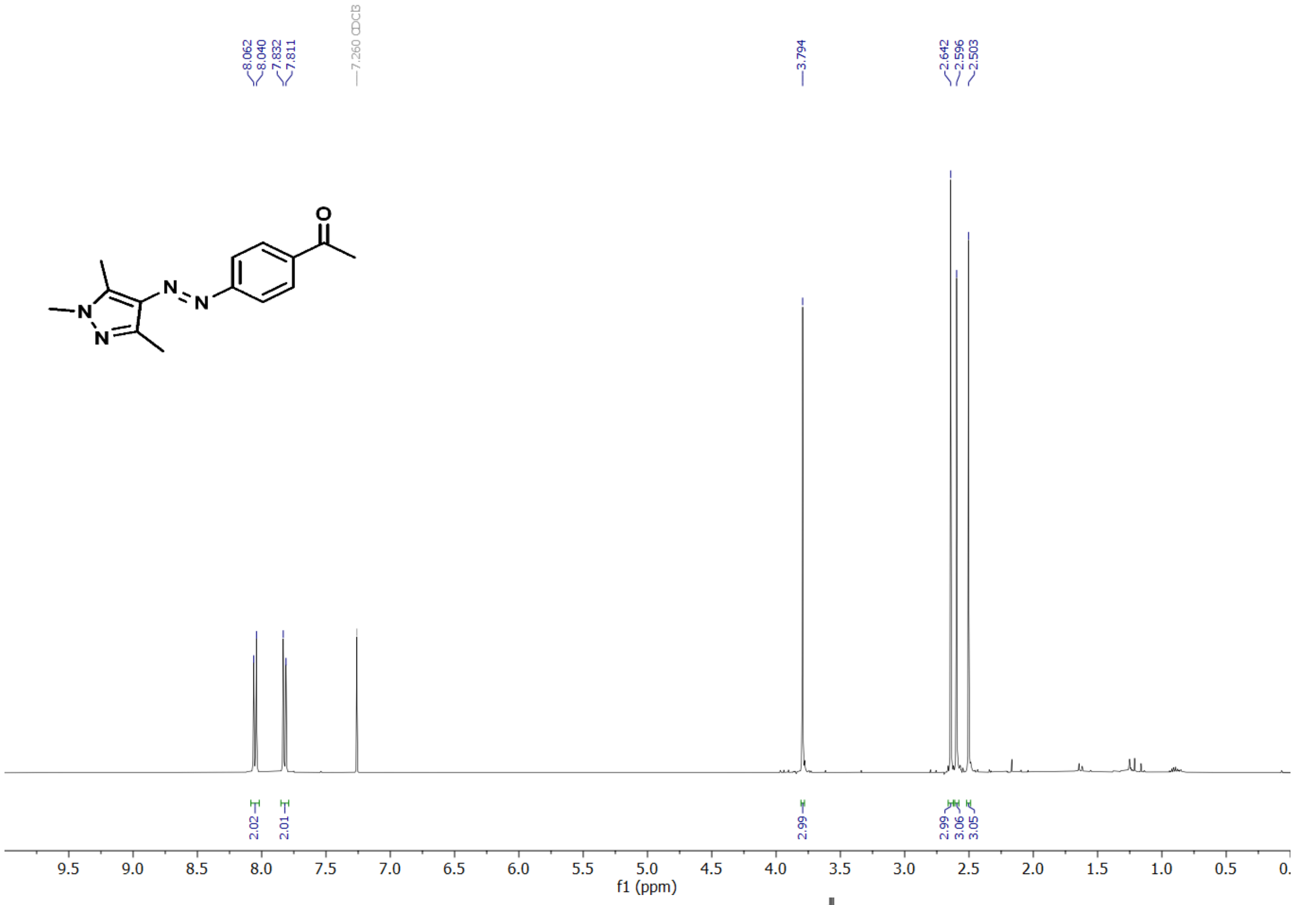

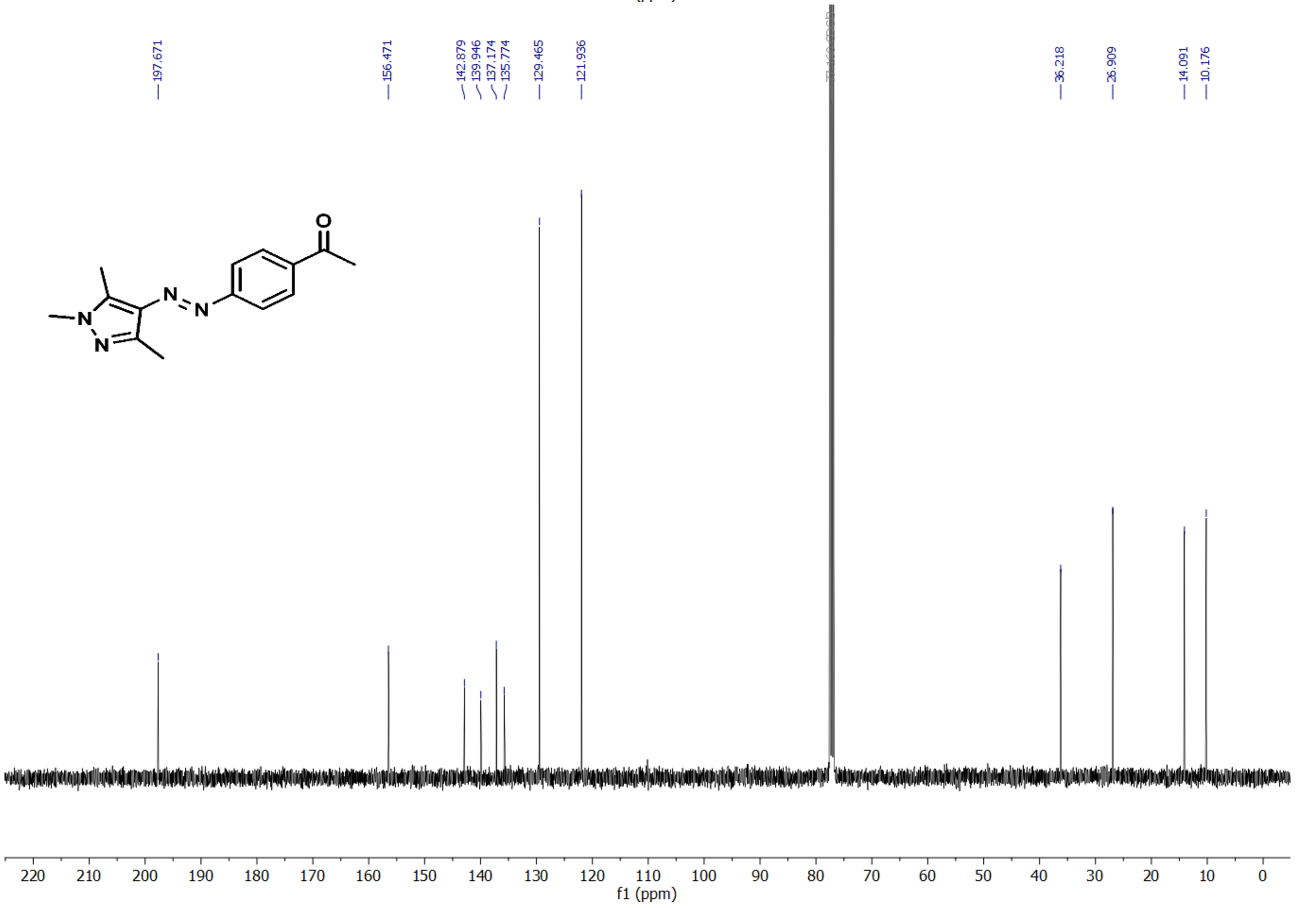

4-((1,3,5-Trimethyl-1H-pyrazol-4-yl)diazenyl)aniline (3r)

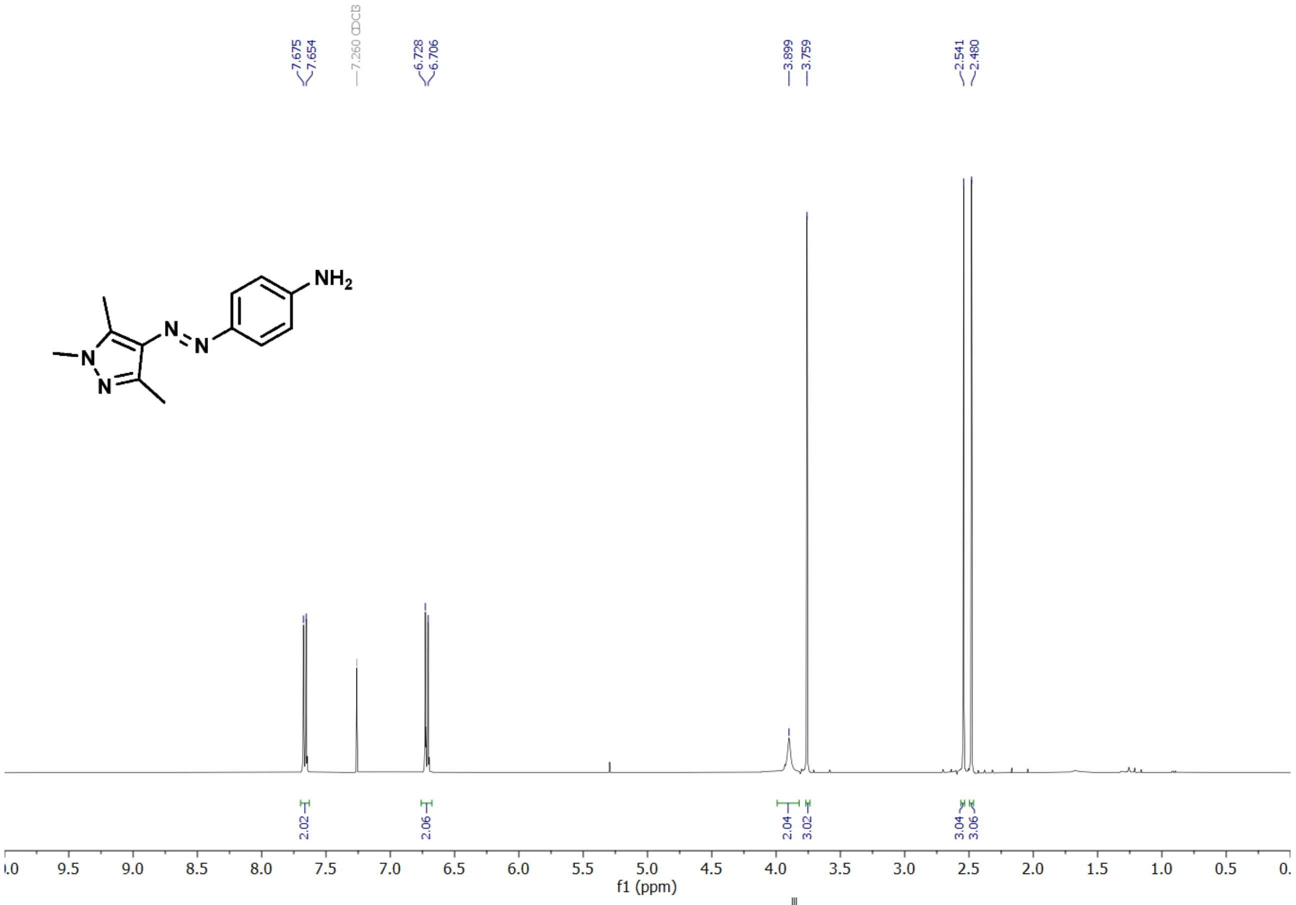

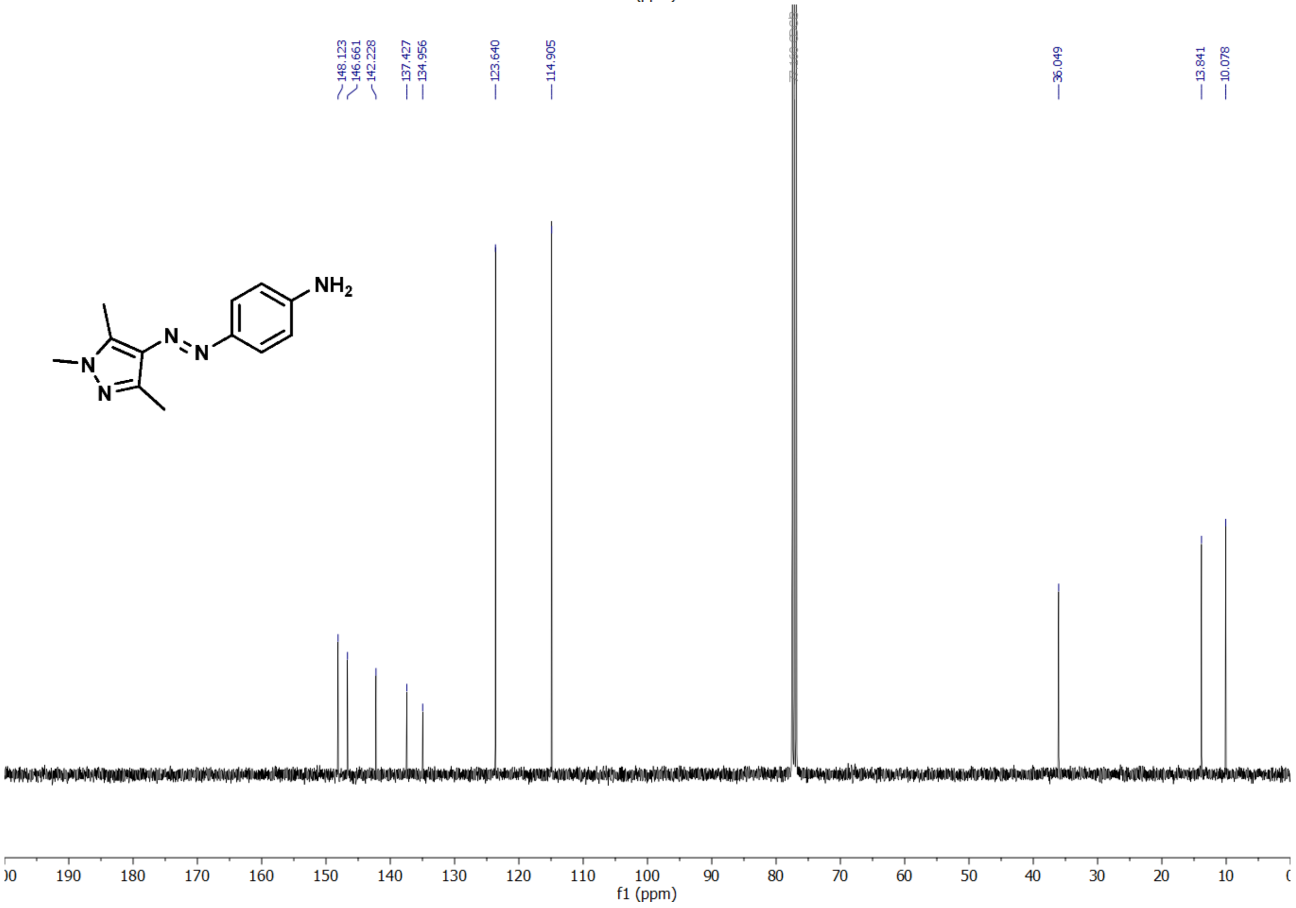

4-((4-Methoxyphenyl)diazenyl)-1,3,5-trimethyl-1H-pyrazole (3s)

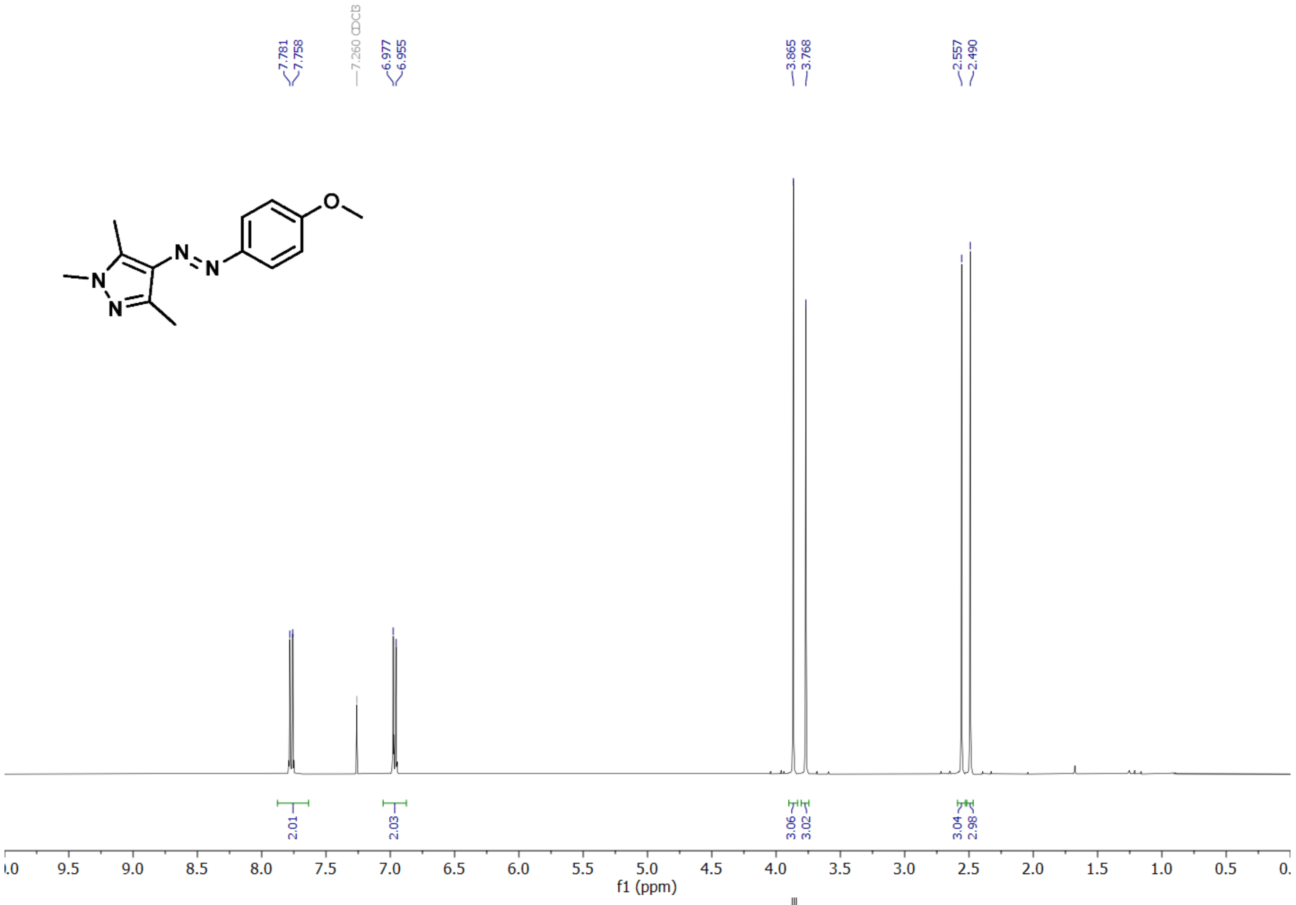

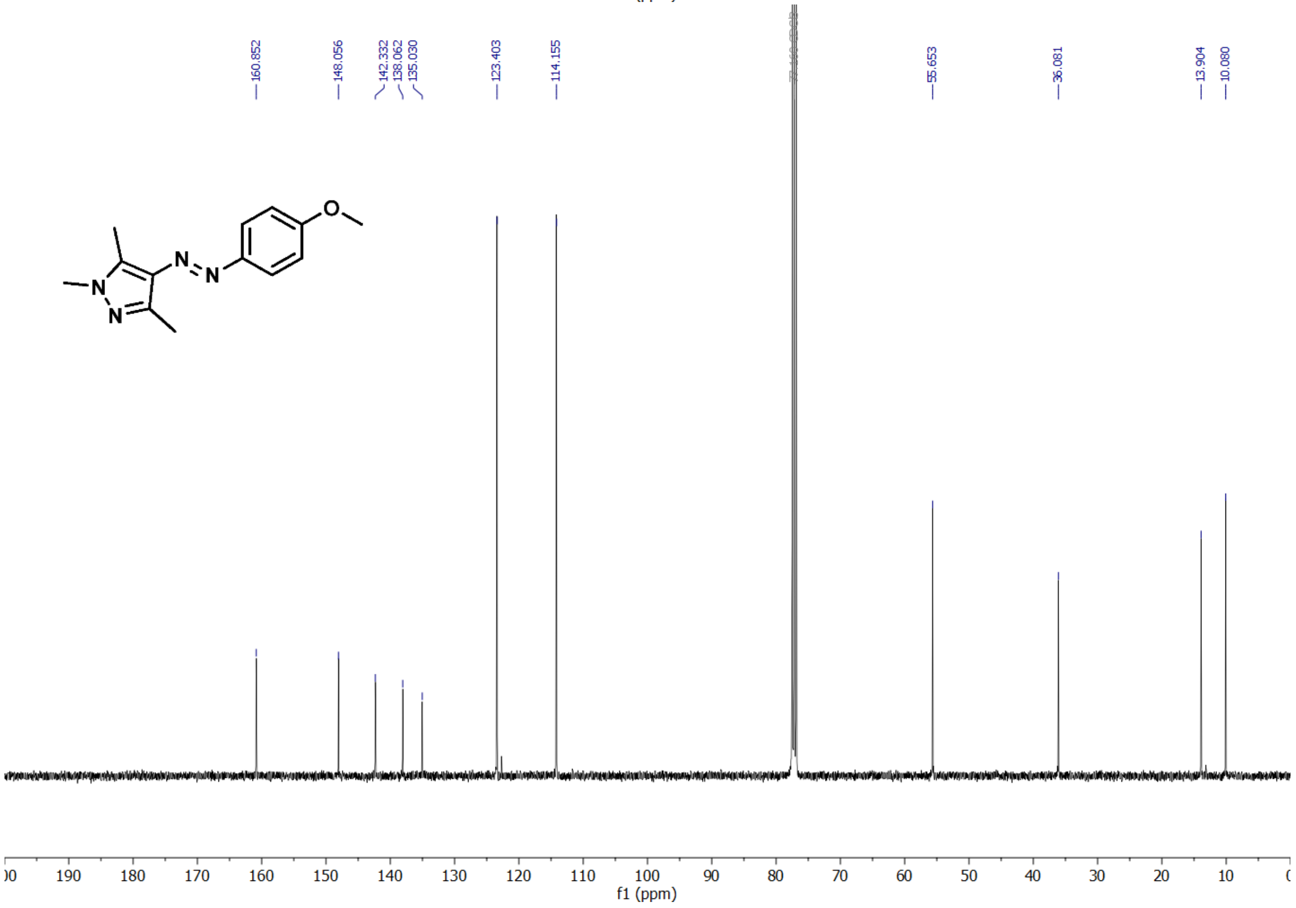

4-((1,3,5-Trimethyl-1H-pyrazol-4-yl)diazenyl)benzamide (3t)

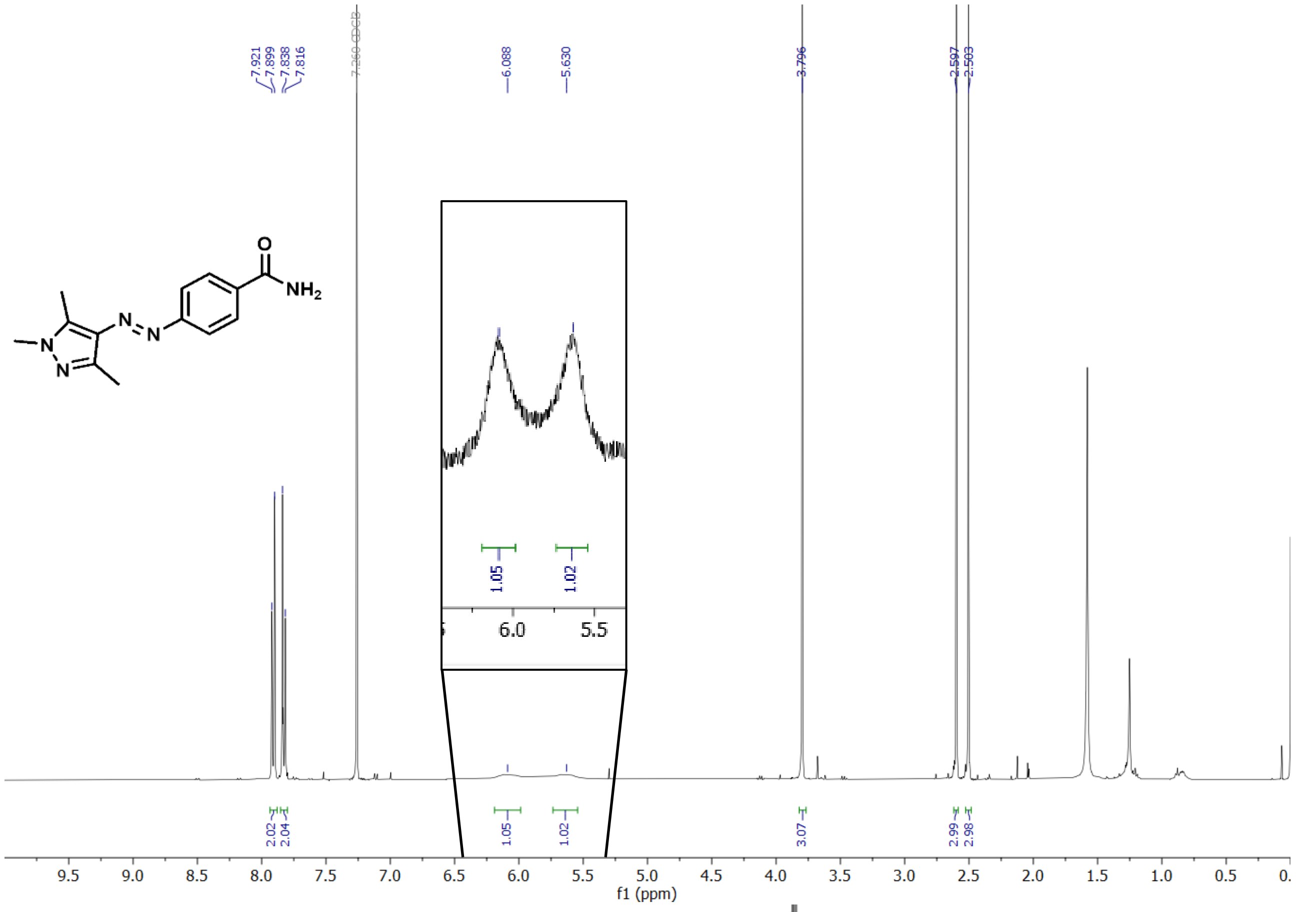

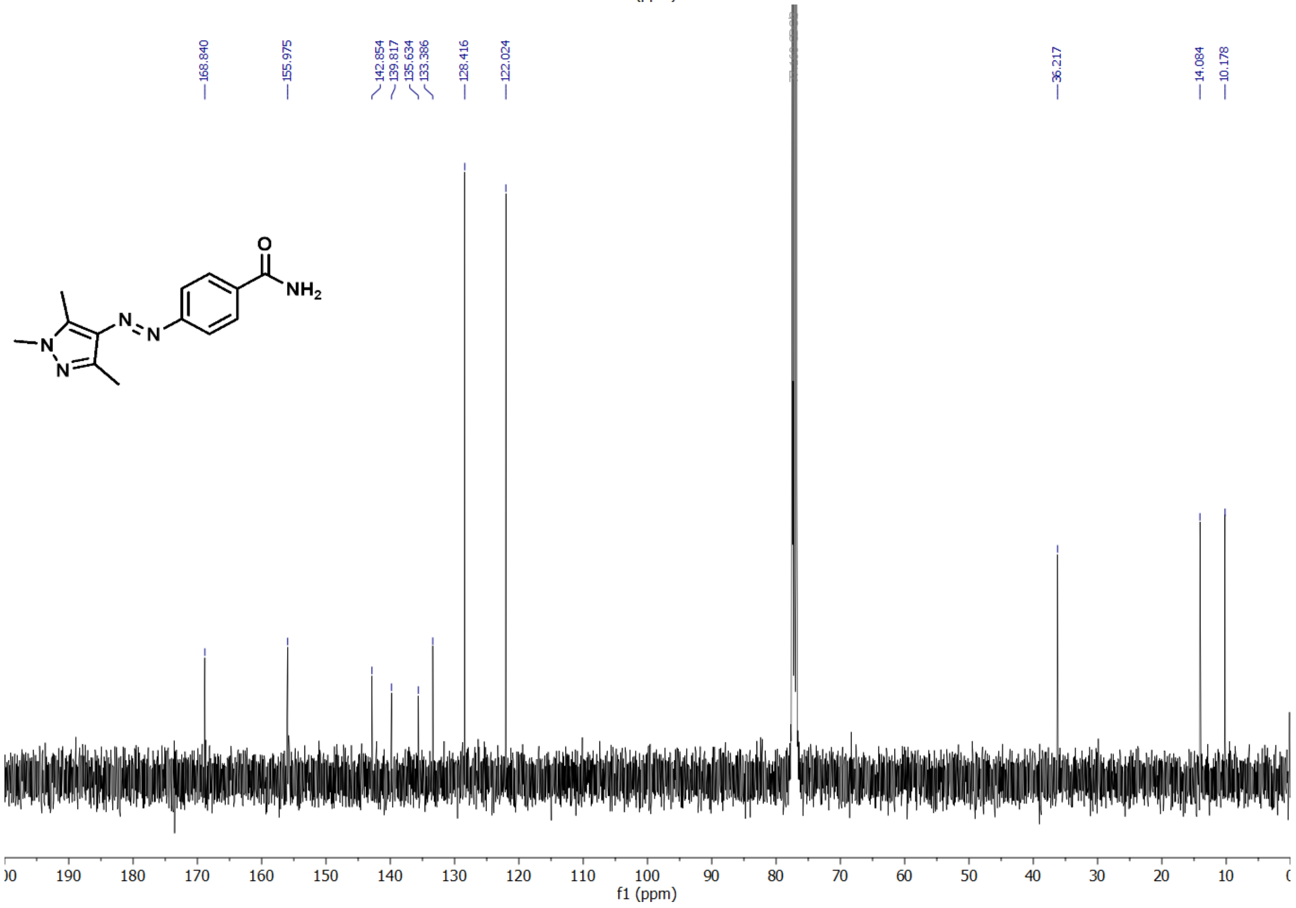

Ethyl 4-((1,3,5-trimethyl-1H-pyrazol-4-yl)diazenyl)benzoate (3u)

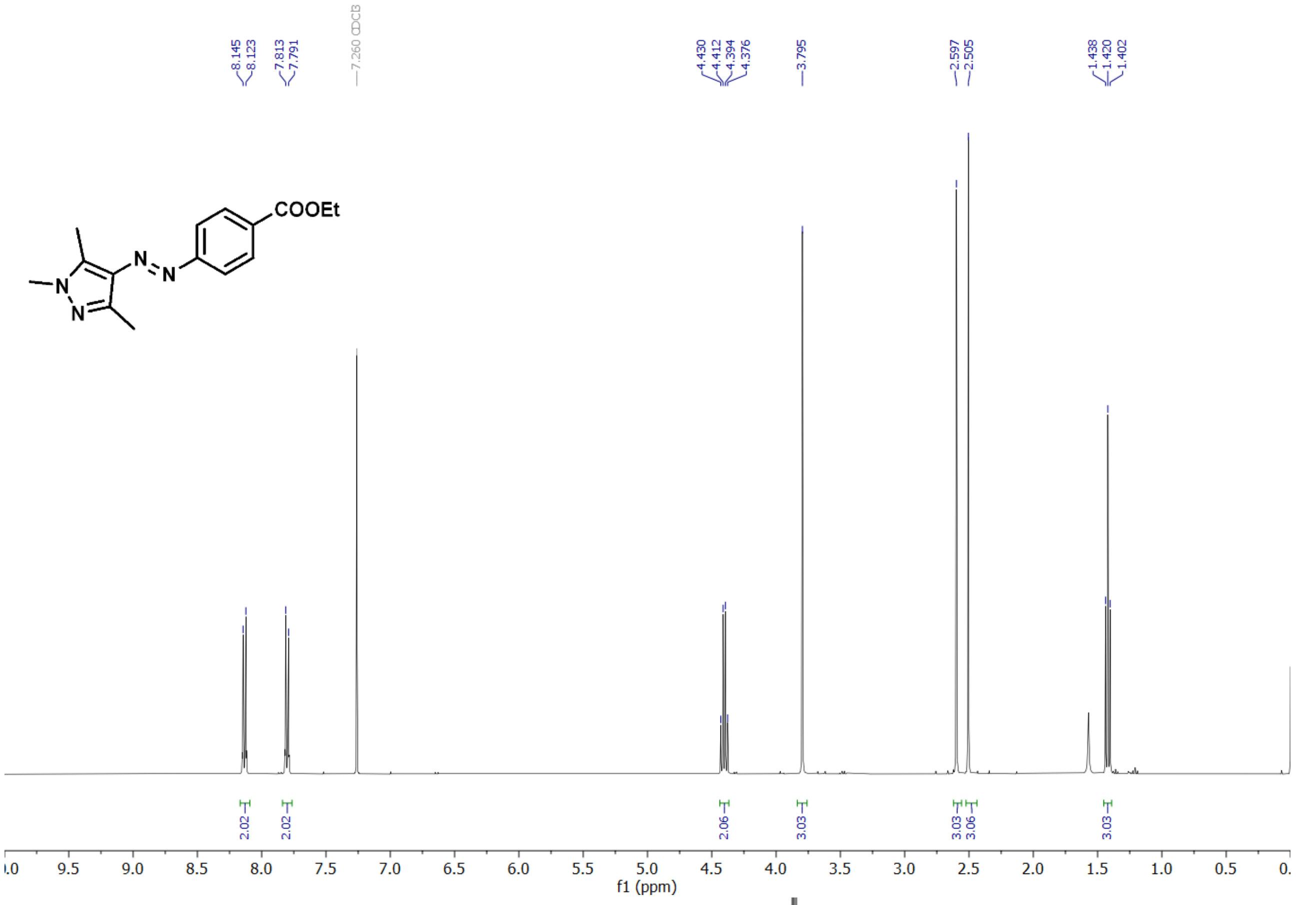

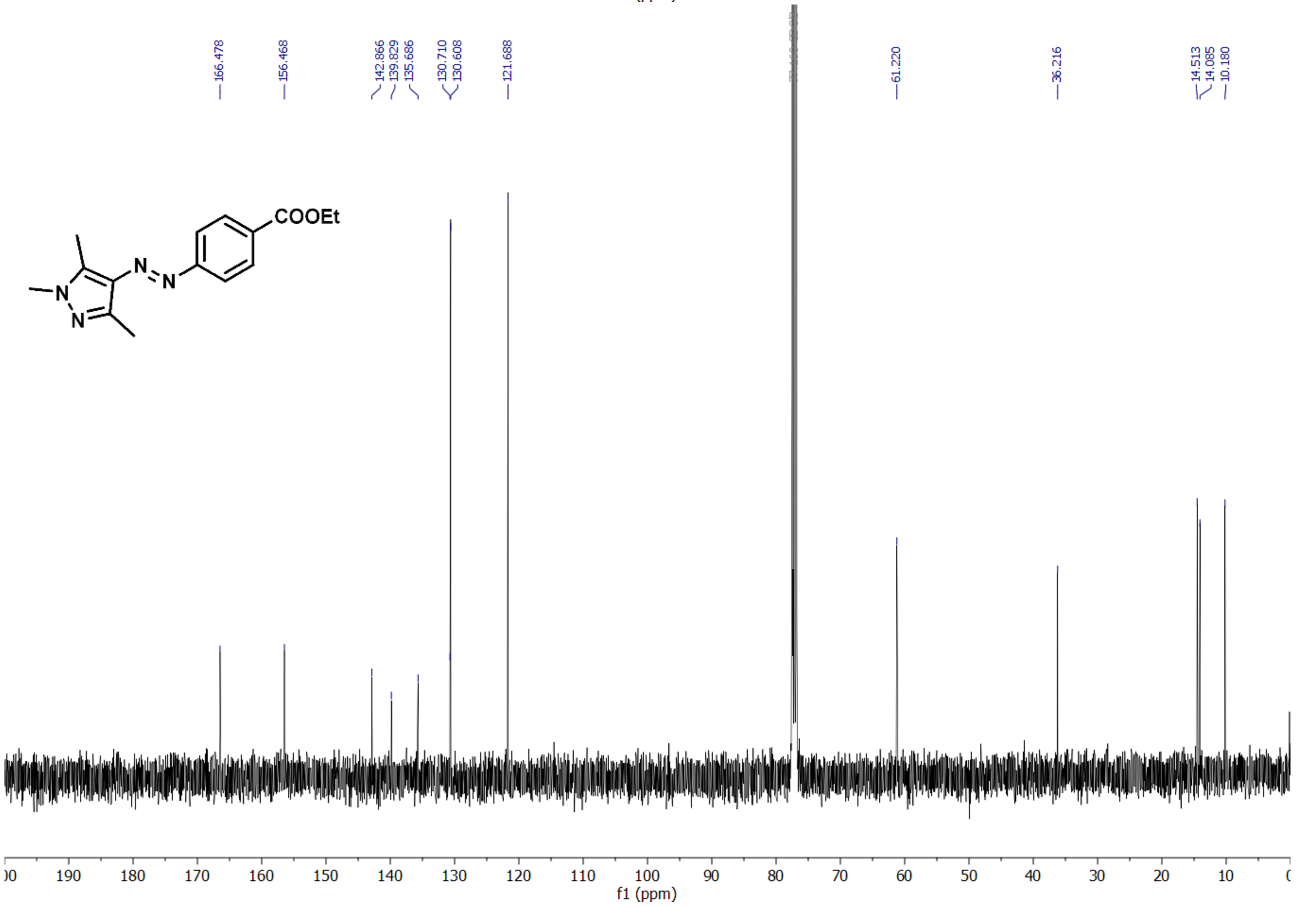

N-Phenyl-4-((1,3,5-trimethyl-1H-pyrazol-4-yl)diazenyl)benzamide (3v)

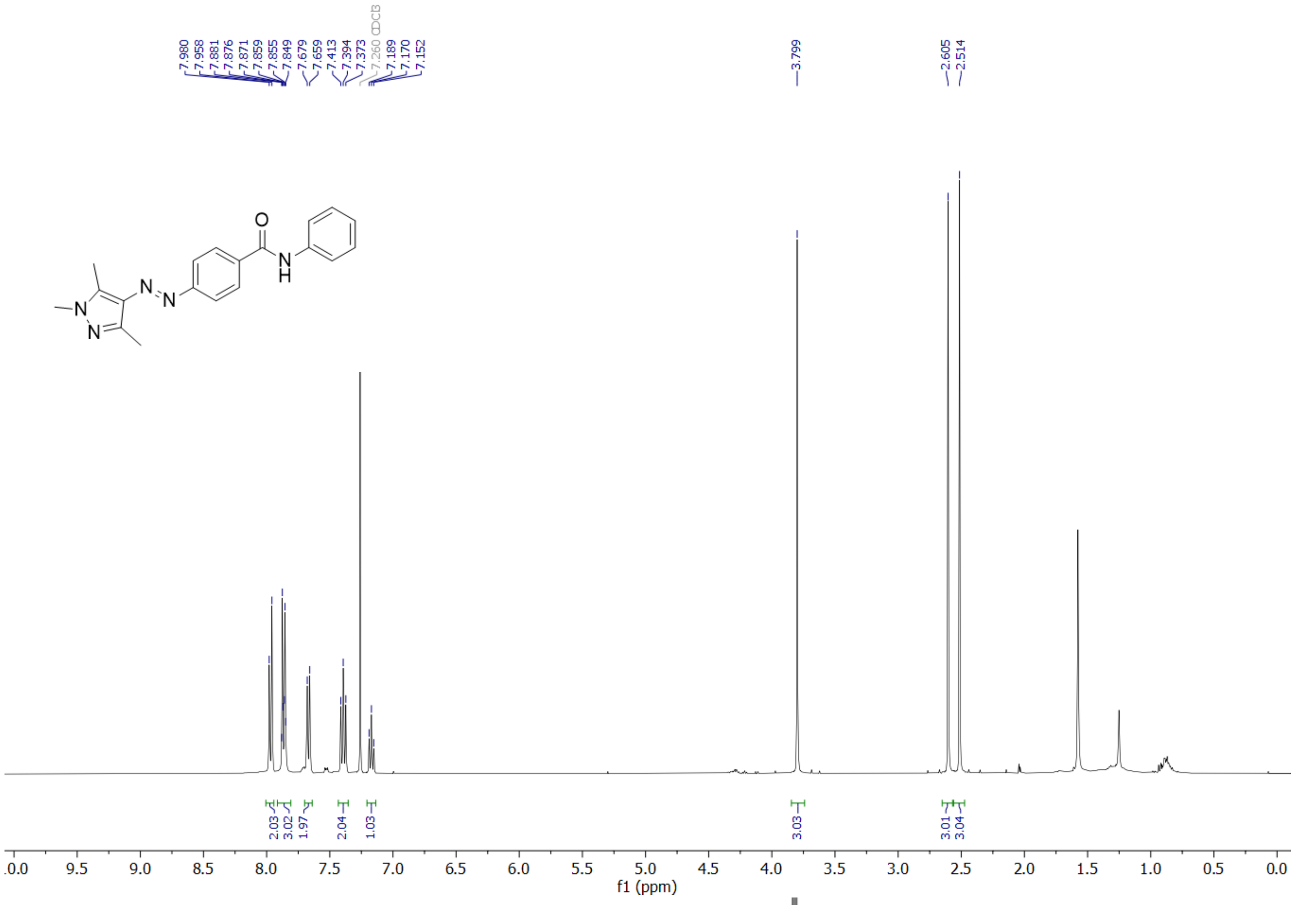

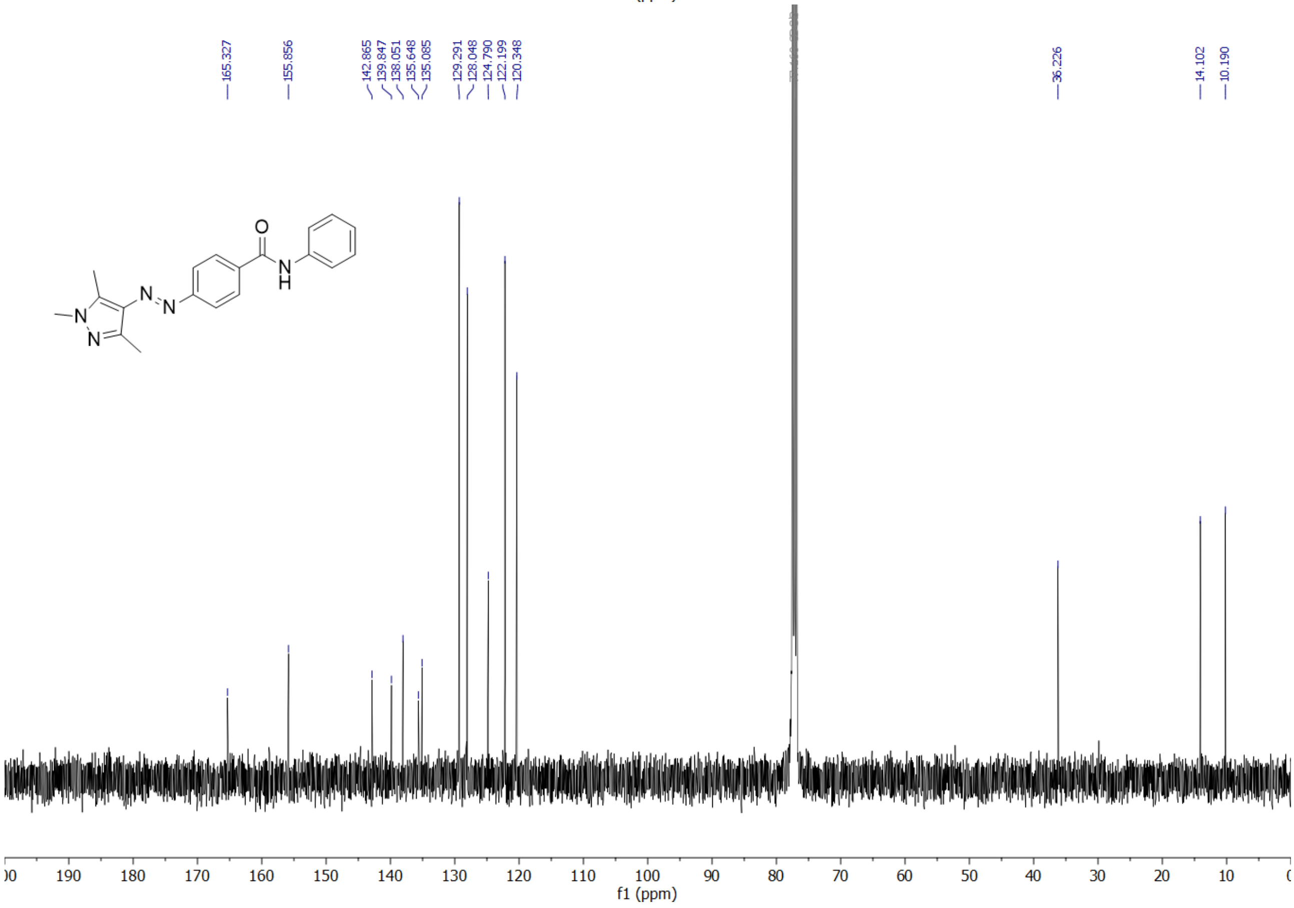

N-Benzyl-4-((1,3,5-trimethyl-1H-pyrazol-4-yl)diazenyl)benzamide (3w)

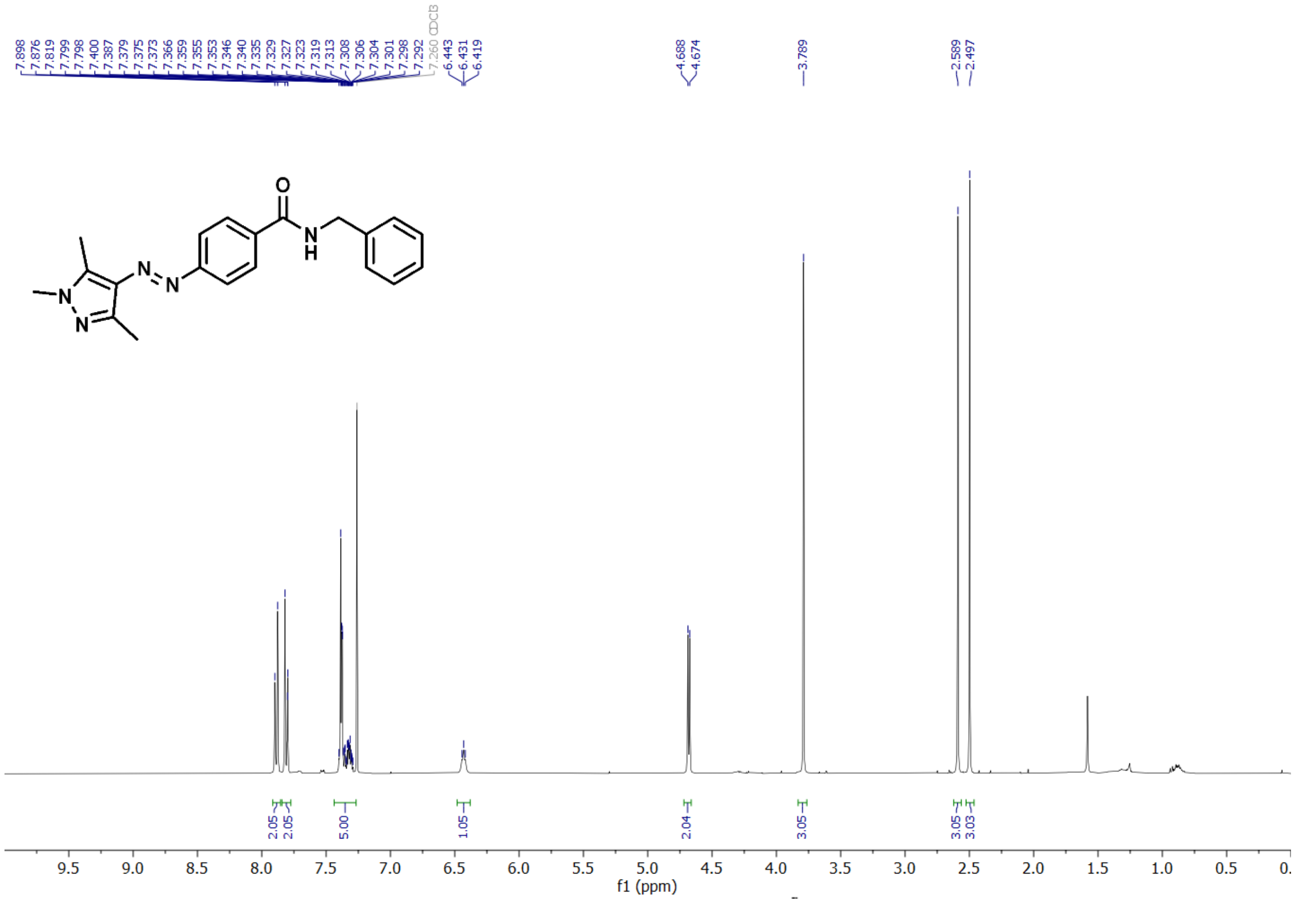

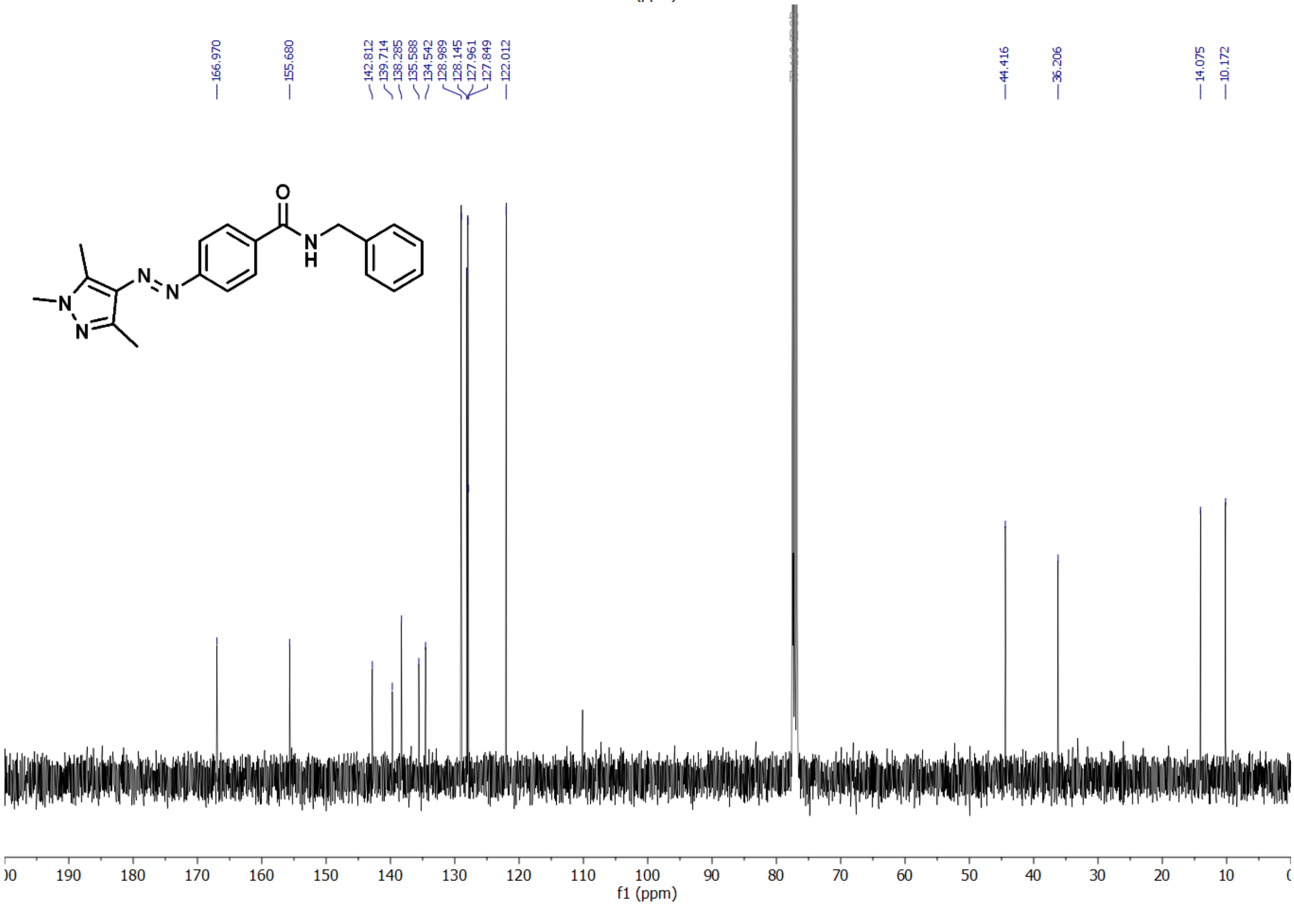

SECTION S3: EXPERIMENTAL UV/VIS SPECTRA

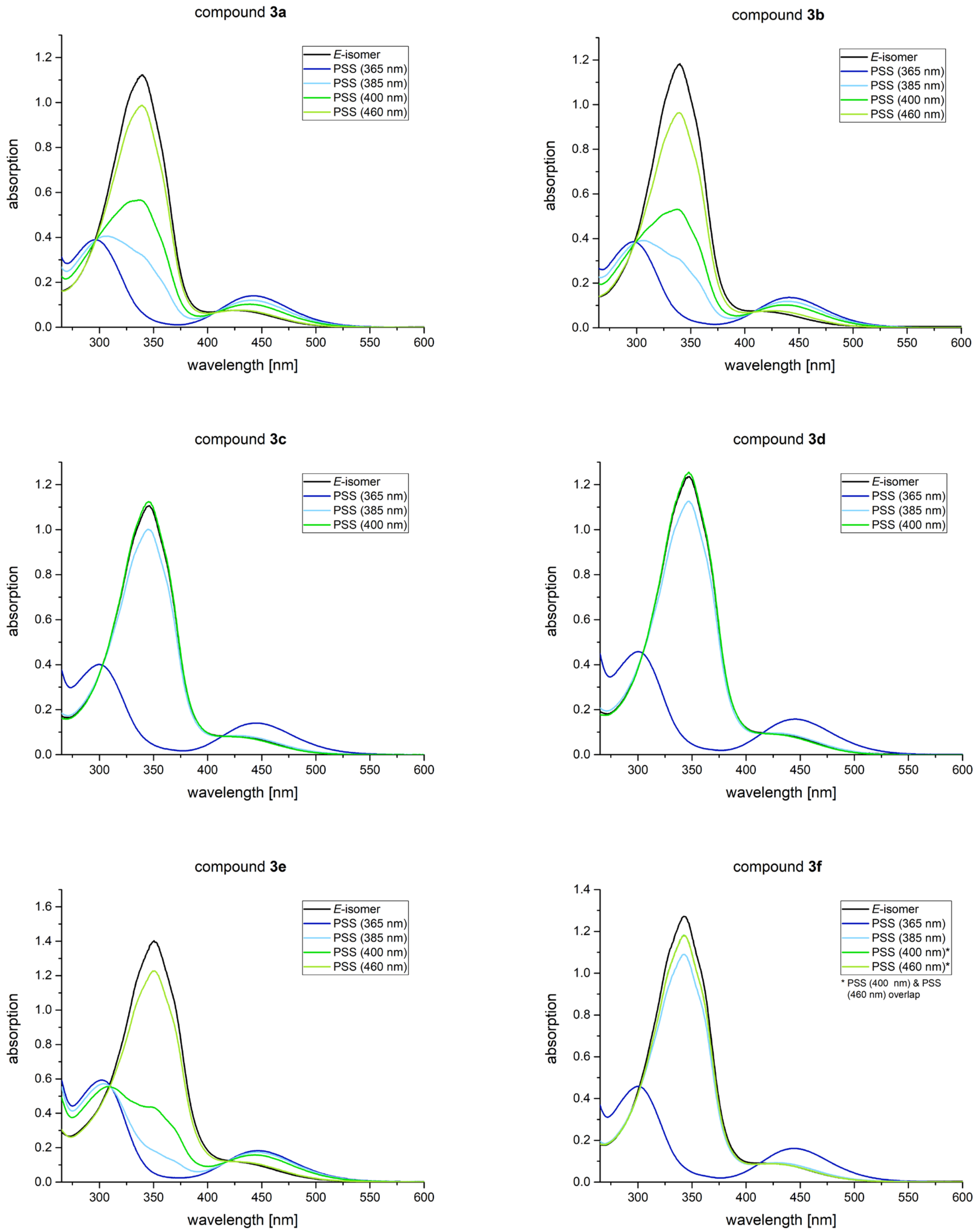

Figure S1. A) UV/Vis spectra of compounds **3a** – **3f** in the dark (= *E*-isomer, black line) and after irradiation with several wavelengths showing respective PSSs (green and blue lines).

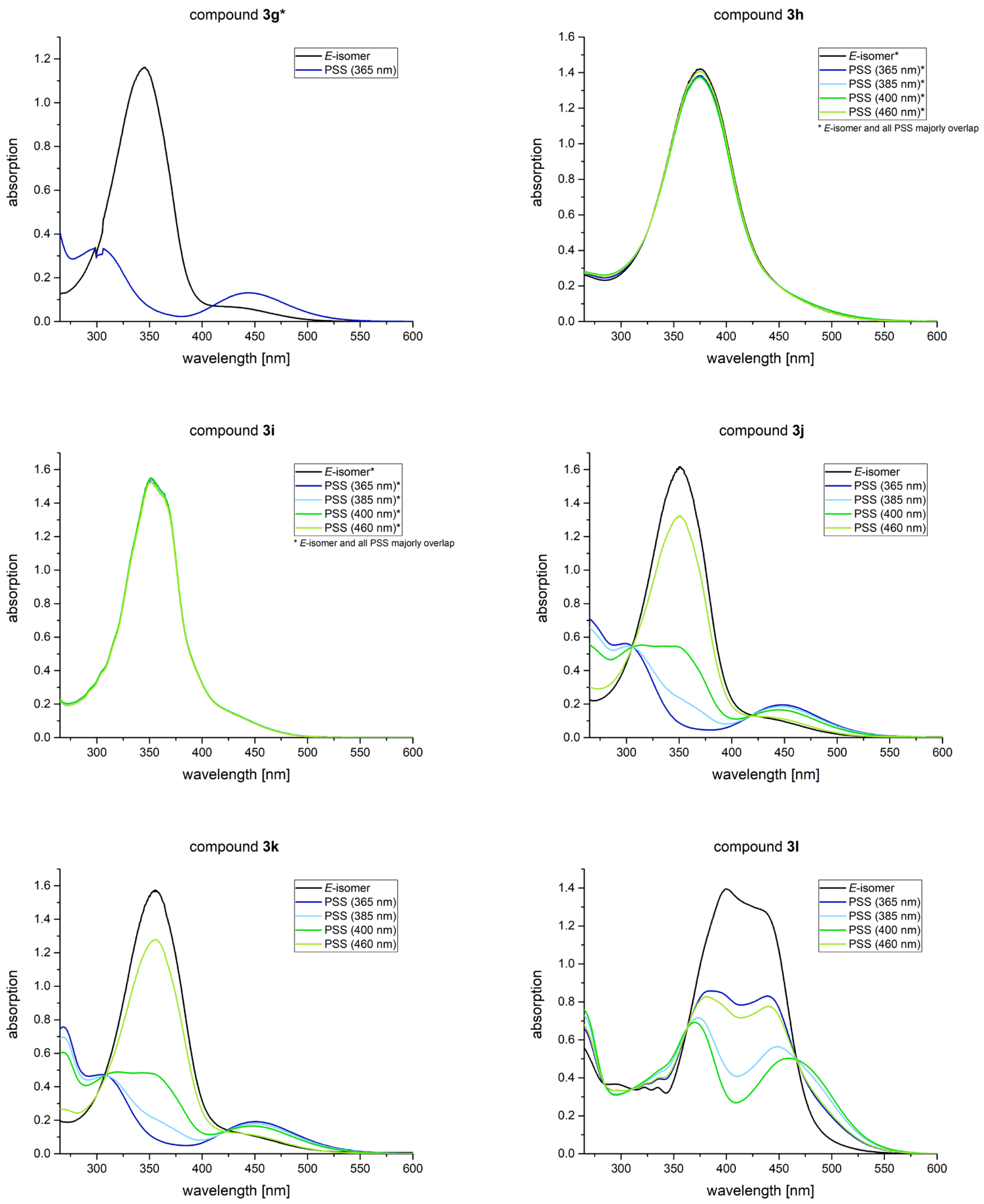

Figure S1. B) UV/Vis spectra of compounds **3g** – **3l** in the dark (= *E*-isomer, black line) and after irradiation with several wavelengths showing respective PSSs (green and blue lines), *UV/Vis spectra of compound **3g** exhibit a kink around 300 nm as the UV/Vis spectrophotometer changed lamps during measurement at these wavelengths.

Note: As the instrumentation available to us was not suitable for measuring PSSs for compounds with ultra-short half-life times, "PSSs" and *E*-isomer spectra for compounds **3h** and **3i** majorly overlap.

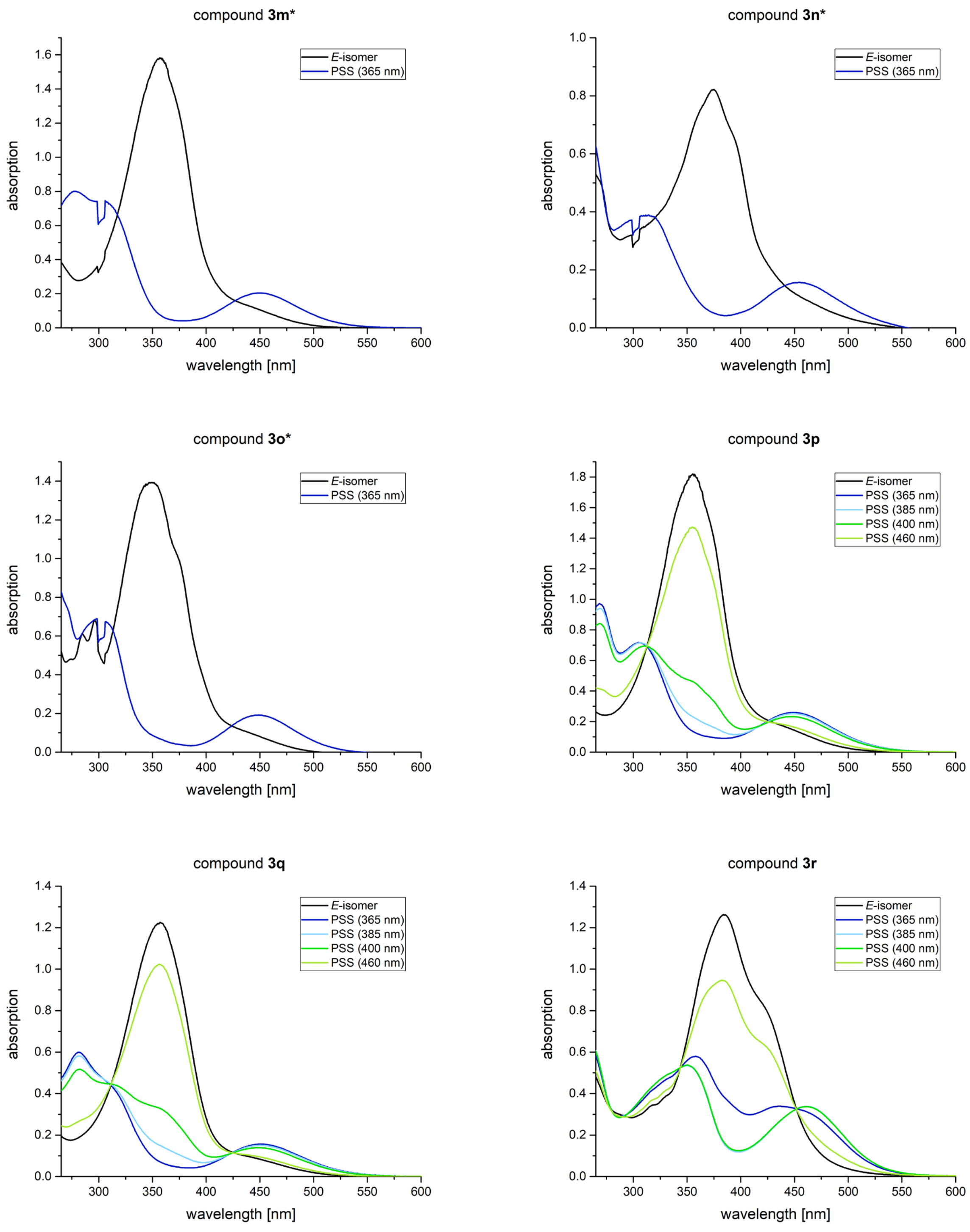

Figure S1. C) UV/Vis spectra of compounds **3m** – **3r** in the dark (= *E*-isomer, black line) and after irradiation with several wavelengths showing respective PSSs (green and blue lines), *UV/Vis spectra of compounds **3m**, **3n**, and **3o** exhibit a kink around 300 nm as the UV/Vis spectrophotometer changed lamps during measurement at these wavelengths.

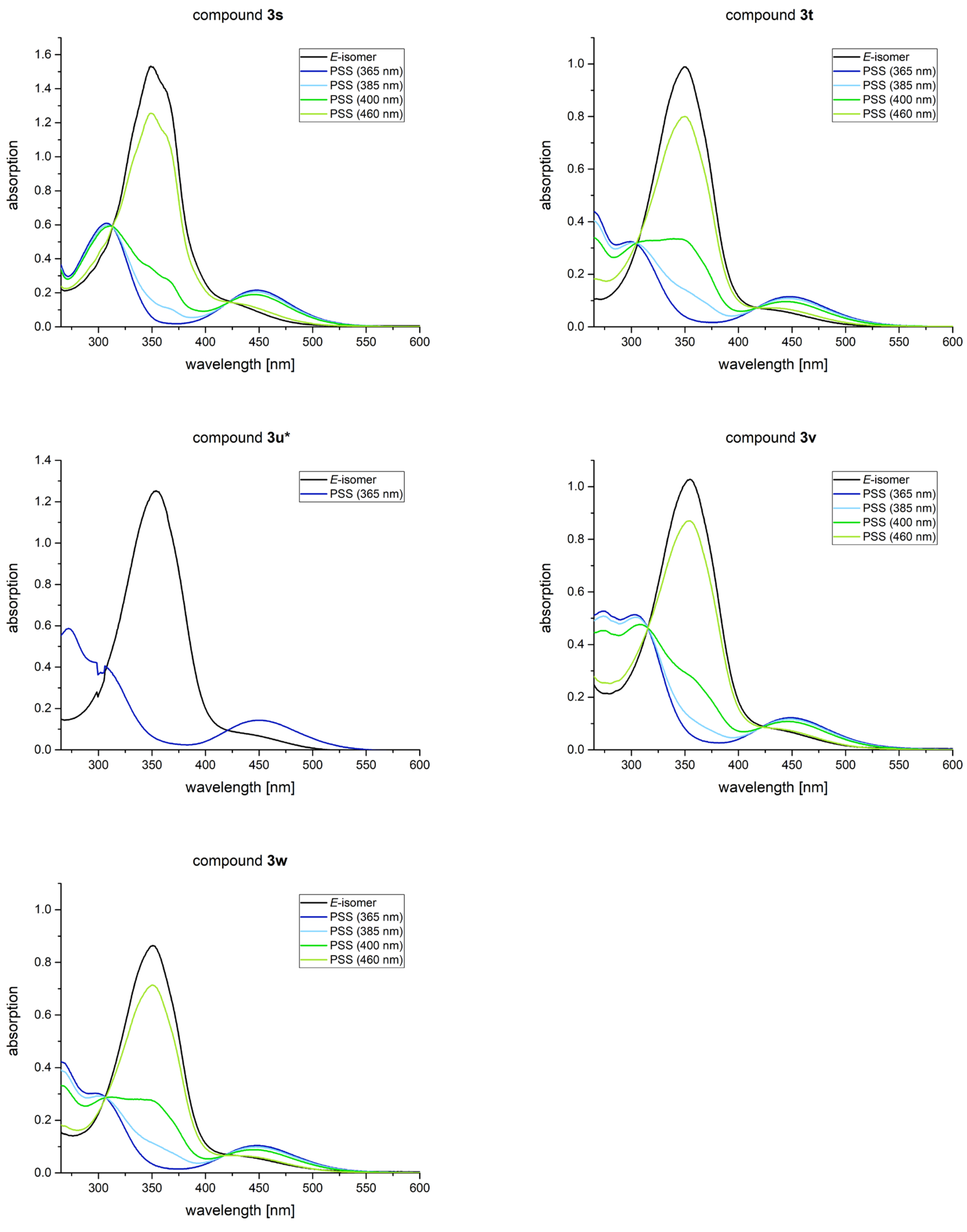

Figure S1. D) UV/Vis spectra of compounds **3s** – **3w** in the dark (= *E*-isomer, black line) and after irradiation with several wavelengths showing respective PSSs (green and blue lines), *UV/Vis spectra of compound **3u** exhibit a kink around 300 nm as the UV/Vis spectrophotometer changed lamps during measurement at these wavelengths.

SECTION S4: CALCULATION OF PSS

Method 1

For most compounds **3a-3w**, the *Z*/*E* ratio for the PSS with the highest observed *Z*-isomer content (most of the time at 365 nm) was calculated according to a method by Fischer.[4] This method allows the calculation of PSSs of systems A ⇌ B, here *E*- and *Z*-isomer, when only A (*E*-isomer) is known. The method assumes that the ratio of Φ_E/Φ_Z , Φ_E being the quantum yield of $E \overset{h\nu}{\rightarrow} Z$ and Φ_Z being the quantum yield of $Z \overset{h\nu}{\rightarrow} E$, does not differ for two chosen wavelengths. As this is generally the case, the *Z*-isomer content can be calculated for all experimentally measured PSS spectra. Our main interest was the maximum *Z*-isomer content reached. For the sake of completion all *Z*-isomer contents for available PSS were calculated. The results can be seen in Table S2.

Table S2. Overview over necessary data and results of the calculation of the *Z*-content in the PSS at several wavelengths according to Fischer[4] of compounds **3a-3f**, **3j-3l**, **3p-3t** and **3v-3w**. [a]The UV/Vis spectra of the *E*-isomer of **3c** and **3d** and their respective PSS at 400 nm overlap nearly fully at the relevant wavelengths of 365 nm and 400 nm. Therefore, the calculation suggests a slight decrease of -1% *Z*-content which can be attributed to measurement variations.

		all *E*	PSS (365 nm)	PSS (385 nm)	PSS (400 nm)	PSS (460 nm)		
**3a**	A at 365 nm	0.556	0.014	0.144	0.267	0.484	$Z\%_{PSS\ (365\ nm)}$	**99%**
	A at 385 nm	0.107	-	0.039	-	-	$Z\%_{PSS\ (385\ nm)}$	75%
	A at 400 nm	0.068	-	-	0.054	-	$Z\%_{PSS\ (400\ nm)}$	52%
	A at 460 nm	0.047	-	-	-	0.052	$Z\%_{PSS\ (460\ nm)}$	13%
**3b**	A at 365 nm	0.580	0.018	0.138	0.249	0.467	$Z\%_{PSS\ (365\ nm)}$	**98%**
	A at 385 nm	0.123	-	0.043	-	-	$Z\%_{PSS\ (385\ nm)}$	77%
	A at 400 nm	0.077	-	-	0.058	-	$Z\%_{PSS\ (400\ nm)}$	58%
	A at 460 nm	0.035	-	-	-	0.047	$Z\%_{PSS\ (460\ nm)}$	20%
**3c**	A at 365 nm	0.782	0.023	0.707	0.793	-	$Z\%_{PSS\ (365\ nm)}$	**97%**
	A at 385 nm	0.205	-	0.185	-	-	$Z\%_{PSS\ (385\ nm)}$	10%
	A at 400 nm	0.097	-	-	0.097	-	$Z\%_{PSS\ (400\ nm)}$	-1%[a]
	A at 460 nm	-	-	-	-	-	$Z\%_{PSS\ (460\ nm)}$	-
**3d**	A at 365 nm	0.919	0.024	0.834	0.932	-	$Z\%_{PSS\ (365\ nm)}$	**97%**
	A at 385 nm	0.264	-	0.239	-	-	$Z\%_{PSS\ (385\ nm)}$	9%
	A at 400 nm	0.121	-	-	0.119	-	$Z\%_{PSS\ (400\ nm)}$	-1%[a]
	A at 460 nm	-	-	-	-	-	$Z\%_{PSS\ (460\ nm)}$	-
**3e**	A at 365 nm	1.147	0.027	0.134	0.342	1.004	$Z\%_{PSS\ (365\ nm)}$	**99%**
	A at 385 nm	0.425	-	0.066	-	-	$Z\%_{PSS\ (385\ nm)}$	89%
	A at 400 nm	0.185	-	-	0.092	-	$Z\%_{PSS\ (400\ nm)}$	71%
	A at 460 nm	0.075	-	-	-	0.084	$Z\%_{PSS\ (460\ nm)}$	13%
**3f**	A at 365 nm	0.802	0.026	0.681	0.742	0.740	$Z\%_{PSS\ (365\ nm)}$	**97%**
	A at 385 nm	0.182	-	0.155	-	-	$Z\%_{PSS\ (385\ nm)}$	15%
	A at 400 nm	0.097	-	-	0.090	-	$Z\%_{PSS\ (400\ nm)}$	8%
	A at 460 nm	0.054	-	-		0.055	$Z\%_{PSS\ (460\ nm)}$	8%
**3j**	A at 365 nm	1.369	0.053	0.181	0.439	1.109	$Z\%_{PSS\ (365\ nm)}$	**98%**
	A at 385 nm	0.578	-	0.096	-	-	$Z\%_{PSS\ (385\ nm)}$	88%
	A at 400 nm	0.230	-	-	0.114	-	$Z\%_{PSS\ (400\ nm)}$	69%
	A at 460 nm	0.079	-	-	-	0.095	$Z\%_{PSS\ (460\ nm)}$	19%
**3k**	A at 365 nm	1.456	0.066	0.177	0.424	1.176	$Z\%_{PSS\ (365\ nm)}$	**97%**
	A at 385 nm	0.785	-	0.104	-	-	$Z\%_{PSS\ (385\ nm)}$	88%
	A at 400 nm	0.316	-	-	0.125	-	$Z\%_{PSS\ (400\ nm)}$	72%
	A at 460 nm	0.089	-	-	-	0.097	$Z\%_{PSS\ (460\ nm)}$	20%

**3l**	A at 365 nm	0.719	0.694	-	-	-	Z%$_{PSS (365 nm)}$	45%
	A at 385 nm	1.204	-	0.632	-	-	Z%$_{PSS (385 nm)}$	80%
	A at 400 nm	1.394	0.832	0.450	0.311	0.765	Z%$_{PSS (400 nm)}$	**87%**
	A at 460 nm	0.718	-		-	0.588	Z%$_{PSS (460 nm)}$	50%
**3p**	A at 365 nm	1.658	0.108	0.194	0.404	1.337	Z%$_{PSS (365 nm)}$	**96%**
	A at 385 nm	0.925	-	0.129	-	-	Z%$_{PSS (385 nm)}$	90%
	A at 400 nm	0.410	-	-	0.152	-	Z%$_{PSS (400 nm)}$	78%
	A at 460 nm	0.114	-	-	-	0.140	Z%$_{PSS (460 nm)}$	20%
**3q**	A at 365 nm	1.156	0.055	0.130	0.300	0.962	Z%$_{PSS (365 nm)}$	**97%**
	A at 385 nm	0.665	-	0.082	-	-	Z%$_{PSS (385 nm)}$	89%
	A at 400 nm	0.282	-	-	0.101	-	Z%$_{PSS (400 nm)}$	75%
	A at 460 nm	0.070	-	-	-	0.083	Z%$_{PSS (460 nm)}$	17%
**3r**	A at 365 nm	1.033	0.555	0.436	0.439	-	Z%$_{PSS (365 nm)}$	76%
	A at 385 nm	1.261	0.381	0.153	0.161	0.944	Z%$_{PSS (385 nm)}$	**95%**
	A at 400 nm	1.057	-	-	0.125	-	Z%$_{PSS (400 nm)}$	**95%**
	A at 460 nm	0.218	-	-	-	0.251	Z%$_{PSS (460 nm)}$	26%
**3s**	A at 365 nm	1.353	0.020	0.111	0.276	1.098	Z%$_{PSS (365 nm)}$	**99%**
	A at 385 nm	0.495	-	0.056	-	-	Z%$_{PSS (385 nm)}$	92%
	A at 400 nm	0.256	-	-	0.093	-	Z%$_{PSS (400 nm)}$	80%
	A at 460 nm	0.062	-	-	-	0.085	Z%$_{PSS (460 nm)}$	19%
**3t**	A at 365 nm	0.796	0.019	0.103	0.257	0.642	Z%$_{PSS (365 nm)}$	**99%**
	A at 385 nm	0.298	-	0.048	-	-	Z%$_{PSS (385 nm)}$	88%
	A at 400 nm	0.115	-	-	0.060	-	Z%$_{PSS (400 nm)}$	68%
	A at 460 nm	0.042	-	-	-	0.055	Z%$_{PSS (460 nm)}$	20%
**3v**	A at 365 nm	0.926	0.037	0.095	0.240	0.781	Z%$_{PSS (365 nm)}$	**97%**
	A at 385 nm	0.466	-	0.054	-	-	Z%$_{PSS (385 nm)}$	90%
	A at 400 nm	0.185	-	-	0.071	-	Z%$_{PSS (400 nm)}$	75%
	A at 460 nm	0.055	-	-	-	0.062	Z%$_{PSS (460 nm)}$	16%
**3w**	A at 365 nm	0.713	0.017	0.084	0.219	0.713	Z%$_{PSS (365 nm)}$	**99%**
	A at 385 nm	0.285	-	0.043	-	-	Z%$_{PSS (385 nm)}$	89%
	A at 400 nm	0.113	-	-	0.054	-	Z%$_{PSS (400 nm)}$	70%
	A at 460 nm	0.046	-	-	-	0.046	Z%$_{PSS (460 nm)}$	18%

Method 2

For the compounds where only one PSS (λ) was available for *Z*-content calculation, method 1 could not be used. Therefore, *Z*-content in PSS (365 nm) of compounds **3g**, **3m**, **3n**, **3o** and **3u** was estimated roughly from the available UV/Vis spectra under the consideration of the Lambert-Beer law (Eq. S1).

$$A = \varepsilon * l * c$$

Equation S1. Lamber-Beer law.

We determined the wavelength between the two isosbestic points of the UV/Vis data of the respective compounds, at which the absorption value for PSS (365 nm) was minimal $\lambda_{A_{min}}(PSS\ (365\ nm))$. Next, we determined the absorption value of the pure *E*-isomer at this same wavelength $A_E(\lambda_{A_{min}}(PSS\ (365\ nm)))$.

As absorption is an additive value, by assuming that the absorption $A_E(\lambda_{A_{min}}(PSS\ (365\ nm)))$ equals 100% *E*-content, we estimated the *Z*-content of PSS (365 nm) assuming that the pure *Z*-isomer absorption value at $\lambda_{A_{min}}(PSS\ (365\ nm))$ would be 0.

This is of course an extremely pessimistic assumption and the thereby obtained values for the *Z*-content in the PSS (365 nm) are very likely too low. However, as we still obtain estimated values ≥94% (Table S3) and already showed near full switchability for most of the other arylazopyrazoles in this paper (*vide supra*), we believe these values are sufficient to further show the supreme switchability of compounds **3g**, **3m**, **3n**, **3o**, and **3u**.

Table S3. Overview over values used for the calculation of the *Z*-content in the PSS (365 nm) for compounds **3g**, **3m**, **3n**, **3o**, and **3u** using only UV/Vis data.

	$\lambda_{A_{min}}(PSS\ (365\ nm))$	$A_{min}(PSS\ (365\ nm))$	$A_E(\lambda_{A_{min}}(PSS\ (365\ nm)))$	*Z*%$_{min}$ $f_{PSS\ (365\ nm)}$
**3g**	379 nm	0.023	0.351	94%
**3m**	377 nm	0.042	1.196	97%
**3n**	387 nm	0.042	0.714	94%
**3o**	385 nm	0.033	0.675	95%
**3u**	382 nm	0.024	0.636	96%

SECTION S5: EXPERIMENTAL KINETIC DATA

The experimental half-lives of the investigated compounds were determined on a UV-1800 UV/Vis spectrophotometer from Shimadzu at 23°C. A 50 µM sample in dry DMSO was preirradiated with 365 nm for 5 seconds (**3l** and **3r** were irradiated with 400 nm for 5 seconds) to reach the highest possible *Z*-isomer content. Then, the absorption at the respective λ_{max}, the *E*-isomer's absorption maximum, was regularly measured over the course of a representative time frame (mainly ranging 7 to 15 hours) at 23°C. This data was used for the determination of the respective half-life times of the thermal *Z*/*E* isomerization. Two different mathematical fitting functions, depending on the length of half-life time, were used.

For half-life times around or less than two hours, the absorption at the chosen wavelength was plotted over time and an exponential fit function (Eq. S2) was applied. Rate constant k was obtained from the fit function.

$$A = A_{max} + (A_0 - A_{max}) * e^{-kt}$$

Equation S2. Exponential fit function for absorption A.

For half-life times longer than two hours, the data was linearized according to Equation S3.

$$A_{linearized} = \ln(A_{max} - A_t)$$

Equation S3. Linear fit for absorption A.

The linearized absorption value gained thereby was again plotted over time and a linear fit function was applied, yielding rate constant k as its slope value.

The half-life time was calculated according to Equation S4 in both cases. The resulting graphs are shown in Figure S2 and the respective half-lives listed in the manuscript in Table 1.

$$\tau_{1/2} = \frac{\ln(2)}{k}$$

Equation S4. Calculation of half-life $\tau_{1/2}$ time from rate constant k.

As exemplary shown for compound **3a** in an earlier publication of the authors[5] this method of thermal half-life time determination is sufficiently precise for the investigated compounds. Even though the compounds are not allowed to fully relax back to 100% *E*-isomer in most cases, the thermal half-life determined by the presented method is very similar to the half-life determined by compiling an Eyring plot. By such means, we were able to decrease the required measurement time for half-life determination drastically, which was important considering that all 23 synthesized arylazopyrazoles were photophysically characterized.

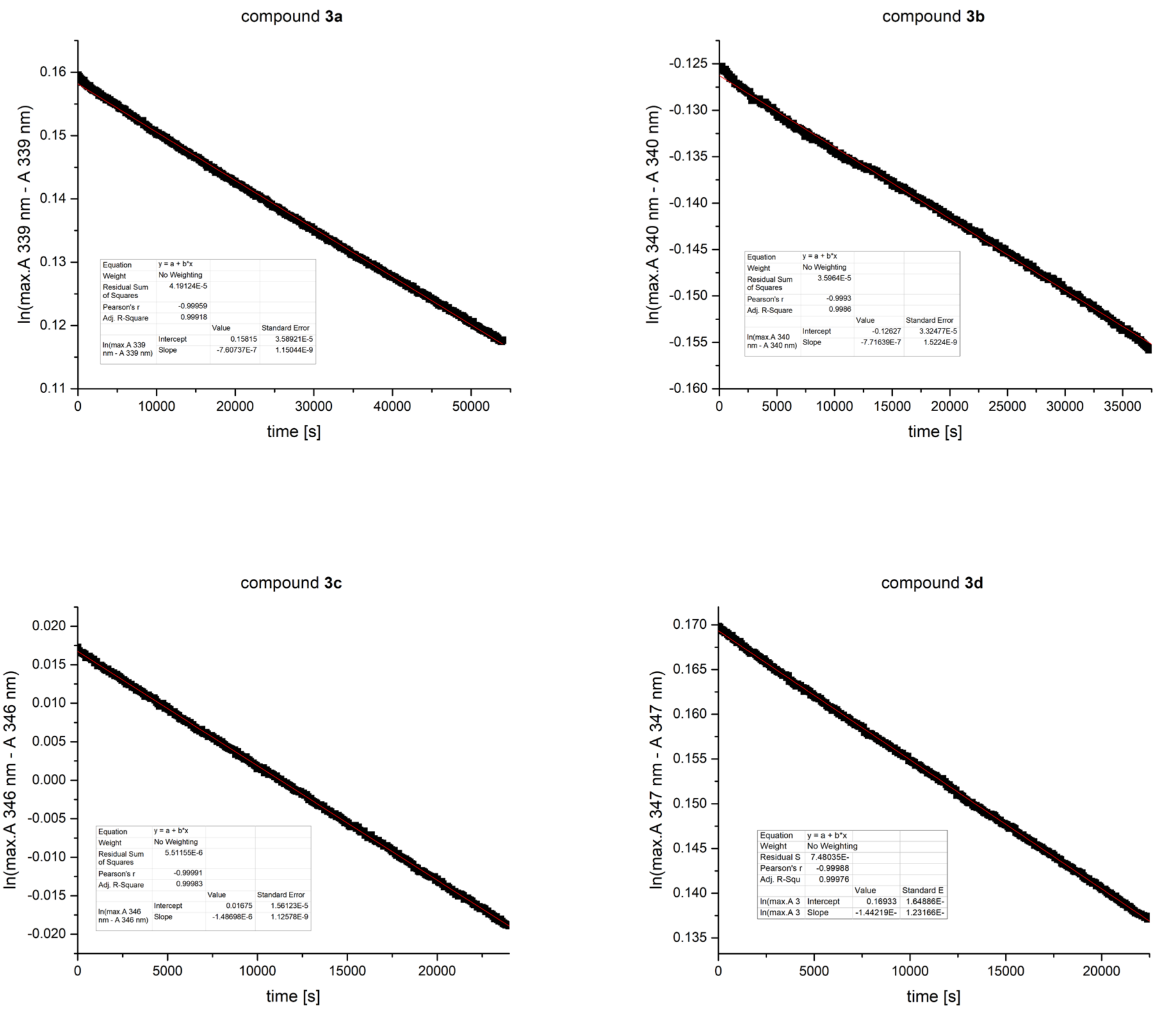

Figure S2. A) Experimental kinetic data for the thermal *Z*/*E* isomerization of compounds **3a-3d**.

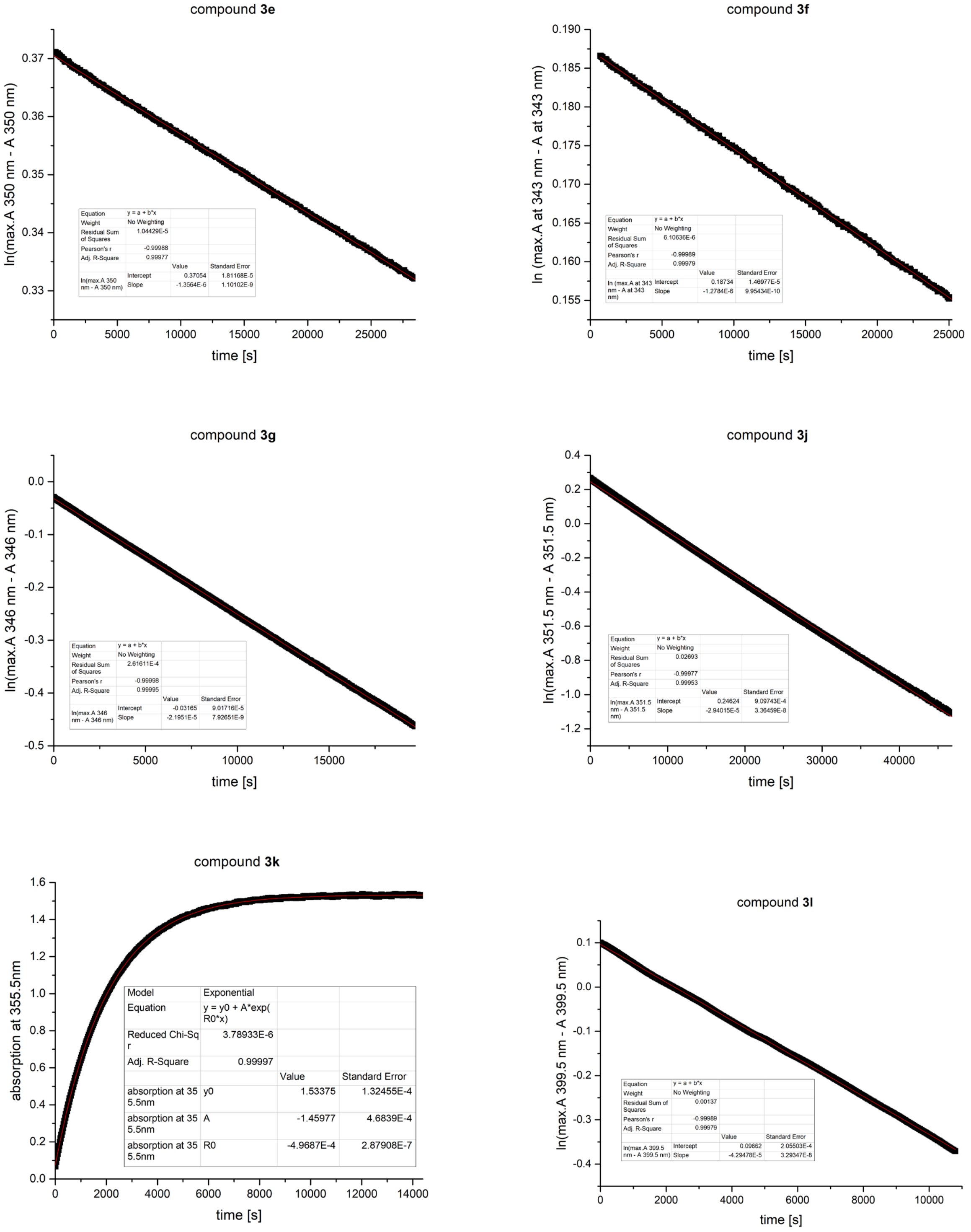

Figure S2. B) Experimental kinetic data for the thermal *Z*/*E* isomerization of compounds **3e-3g** and **3j-3l**.

Note: As the instrumentation available to us was not suitable for measuring ultra-short half-life times, no half-life times for compounds **3h** and **3i** could be obtained.

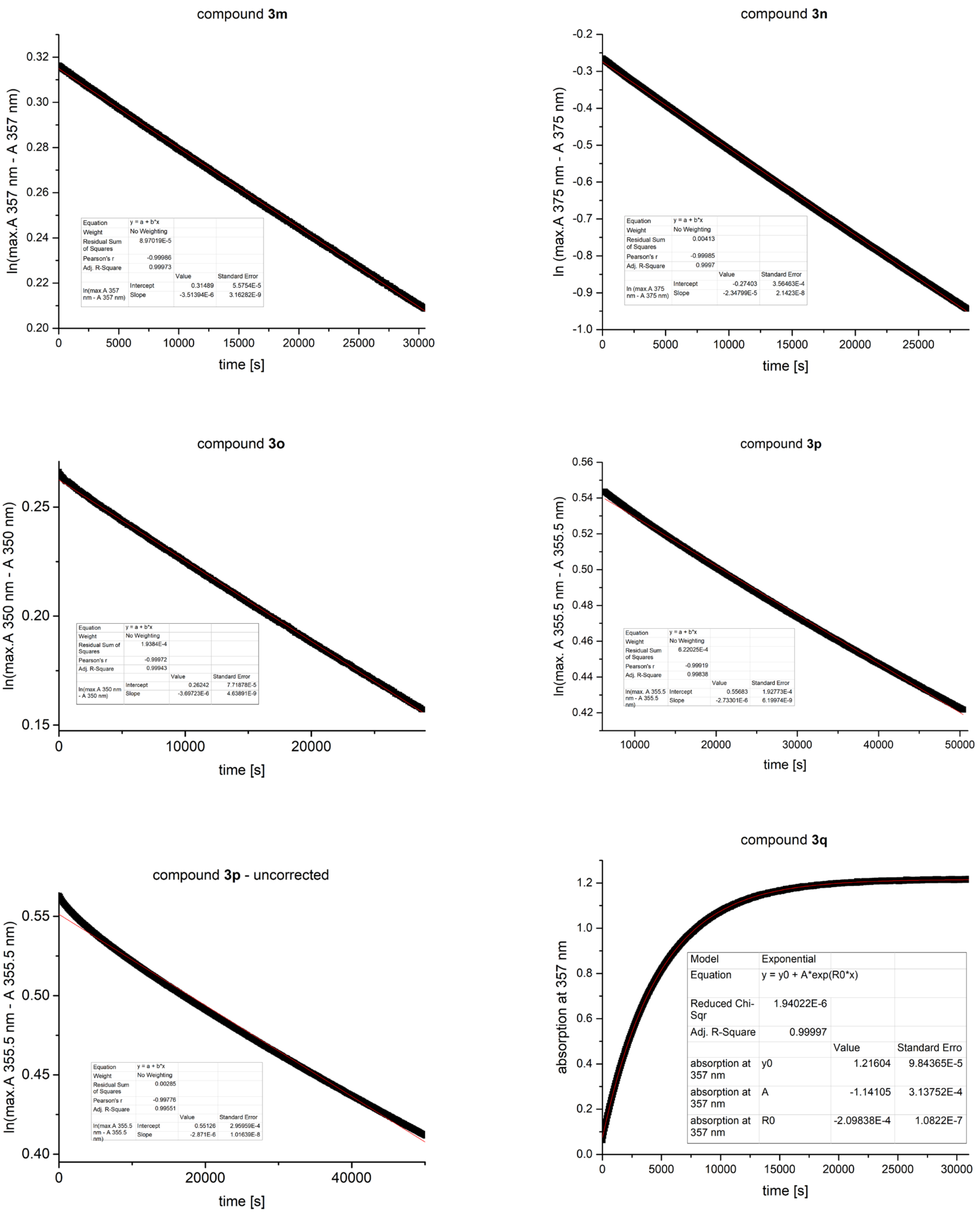

Figure S2. C) Experimental kinetic data for the thermal *Z*/*E* isomerization of compounds **3m-3q**.

Note: For compound **3p** corrected and uncorrected kinetic data is provided. The corrected data set was narrowed in order to provide a better linear fit. As can be seen from the uncorrected data, the half-life time is hardly influenced by this, especially as it is already in the timeframe of a couple of days. The half-life time reported in the paper is the corrected one.

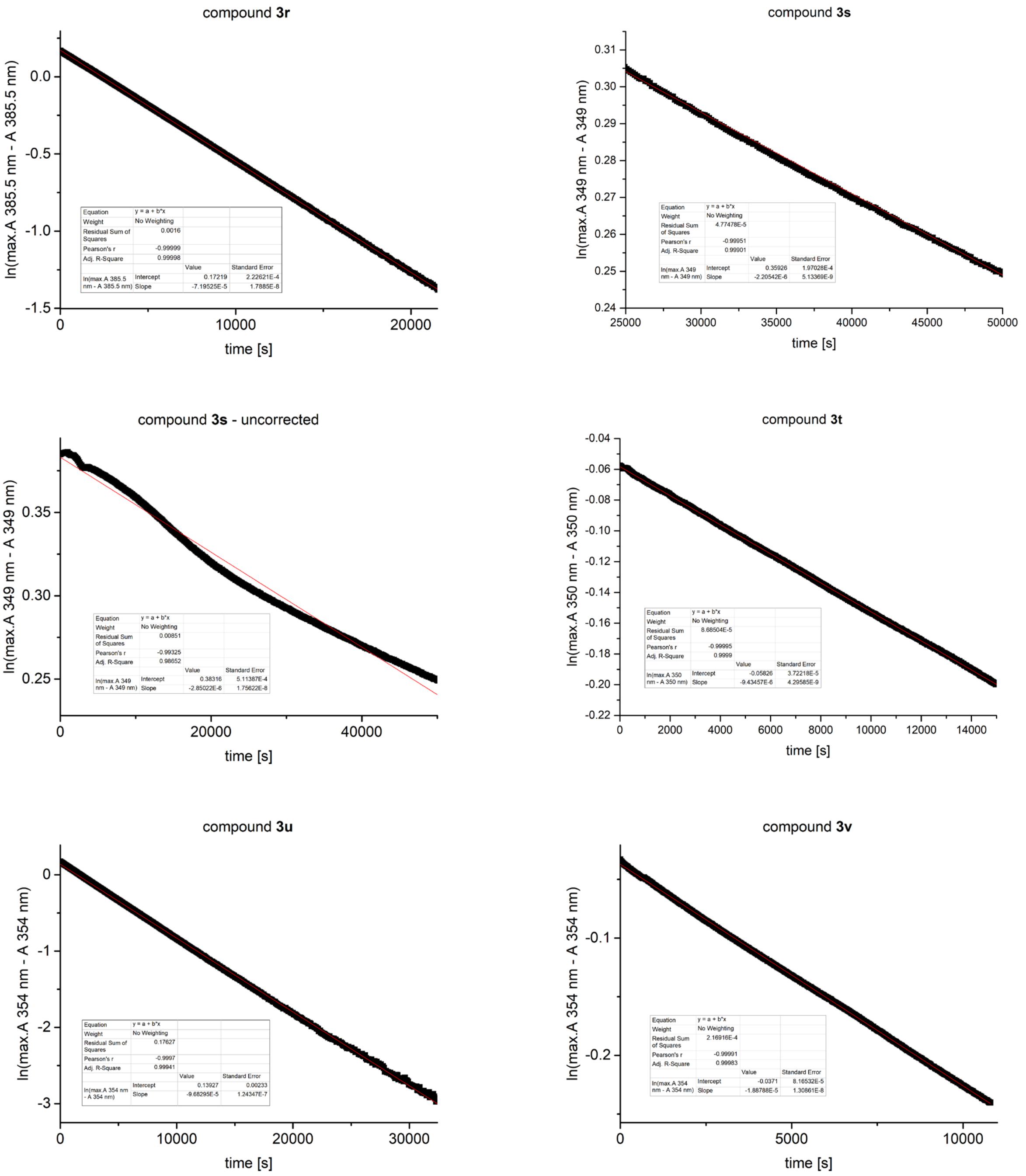

Figure S2. D) Experimental kinetic data for the thermal *Z*/*E* isomerization of compounds **3r-3v**.

Note: For compound **3s** corrected and uncorrected kinetic data is provided. The corrected data set was narrowed in order to provide a better linear fit. As can be seen from the uncorrected data, the half-life time is hardly influenced by this, especially as it is already in the timeframe of a couple of days. The half-life time reported in the paper is the corrected one.

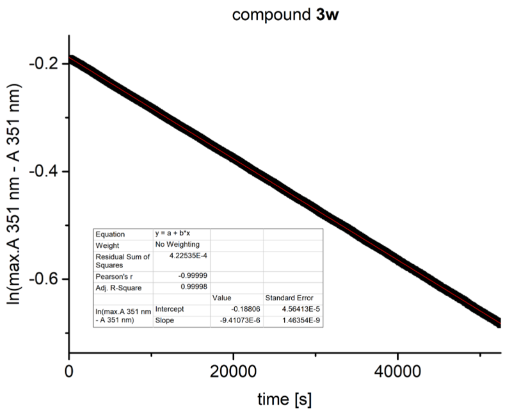

Figure S2. E) Experimental kinetic data for the thermal *Z*/*E* isomerization of compounds **3w**.

SECTION S6: COMPUTATIONAL DETAILS

We followed the same density functional theory (DFT) protocol as in our preceding investigation of the thermal isomerization mechanism of **3a**.[5] The geometry optimizations of *E*, *Z*, TS_{iAr}, and TS_{iPy} geometries were carried out using the ωB97X-D functional[6,7] and the def2-TZVP basis set.[8,9] The solvation model based on density (SMD) variation of the integral-equation-formalism polarizable continuum model (IEF PCM)[10] was used to include the dimethylsulfoxide (DMSO, ε=46.826) as implicit solvent at 23°C and mimic the experimental setup. [10] The calculations were carried out using the *Gaussian 16 v. C.01* suite.[11] According to Axelrod et al.,[12] it is important to account for multi-configurational effects to optimize the rotational TS (TS_r). Therefore, the spin-flip time-dependent DFT (SF-TDDFT) approach was used at the ωB97X-D/def2-TZVP@CPCM(DMSO) level as implemented in *ORCA 5.0*.[6,8,9,13–15] The minimum energy crossing points (MECPs: M1, M2) were optimized at the ωB97X-D/def2-TZVP@SMD(DMSO)[6–10] level of theory using the *ORCA 4.2*[13] optimizer and *Gaussian 16*[11] energies by the *SHARC 2.1* code.[16] All optimized XYZ-geometries are provided.[17] Furthermore, relevant bond lengths, angles, and dihedrals of the computationally optimized structures are shown in Figure S3.

All optimized geometries (except for MECPs) were verified as correct stationary points by frequency analysis (0 imaginary frequencies for minima, 1 imaginary frequency for TSs).

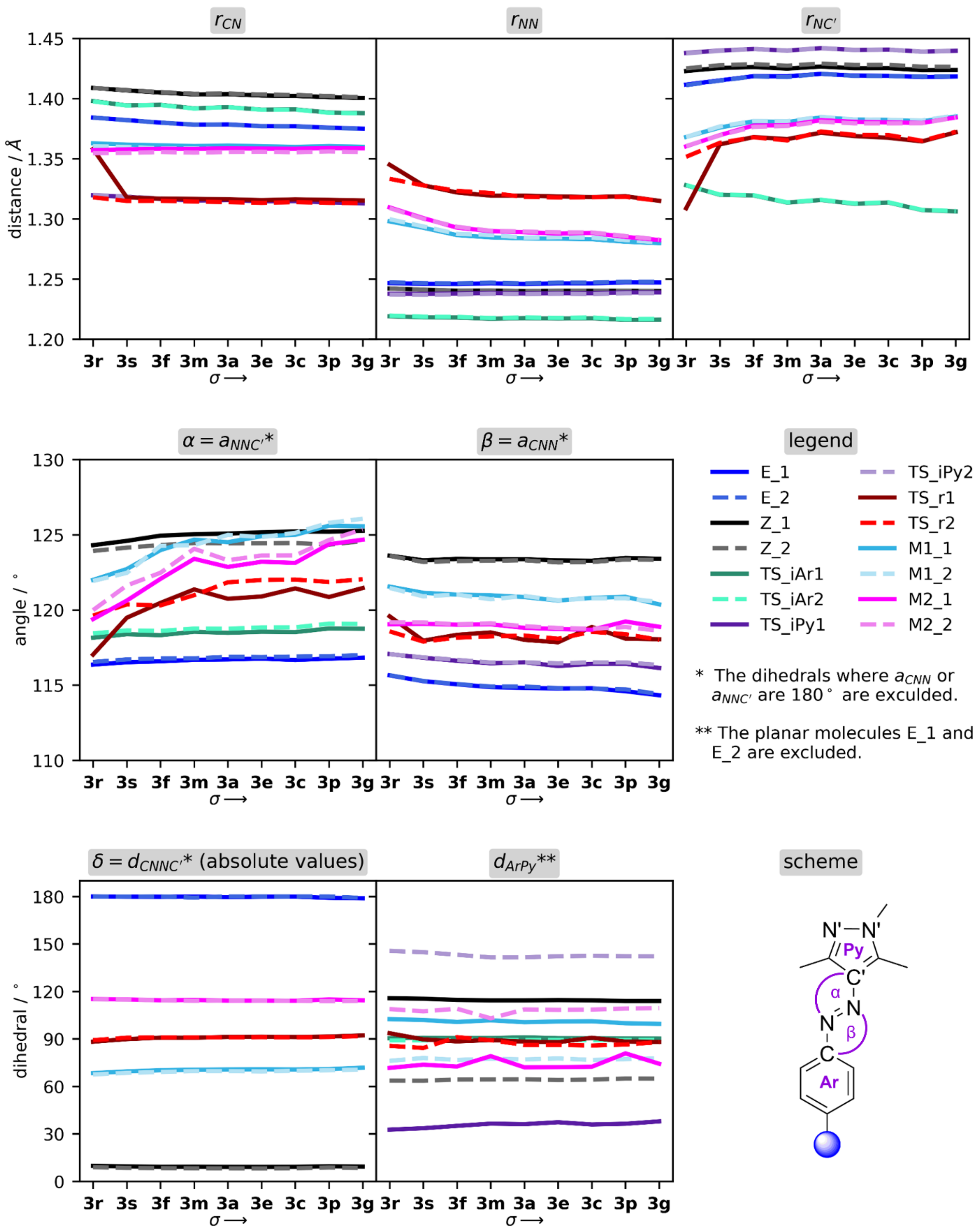

Figure S3: Plotted structural parameters, like bond lengths (r_{CN}, r_{NN}, $r_{NC'}$), angles ($a_{NNC'}$, a_{CNN}), and dihedral angles ($d_{CNNC'}$, d_{ArPy}), relevant to the Z- to E-isomerization mechanisms around the N=N azo bond.

All obtained geometries were followed by ωB97X-D/def2-TZVP@SMD(DMSO)[6–10] Gibbs free energy calculations at 23°C using the *Gaussian 16*[11] suite for consistent results. The results are visualized in Figure S4 and given in Table S4.

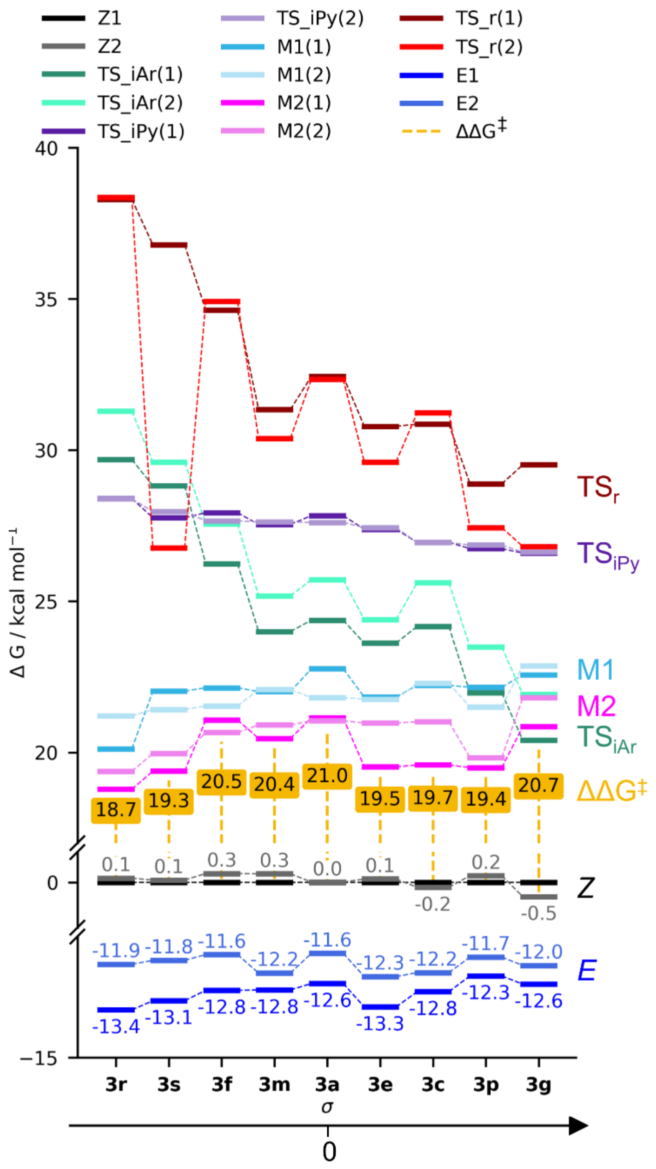

Figure S4. Gibbs free energy (ΔG) plot of all *Z* (black/gray), *E* (dark blue), TS conformers (in green, violet, and red shades), and MECPs (light blue, pink shades) at the ωB97X-D/def2-TZVP@SMD(DMSO) level of theory. All energies are plotted relative to the respective *Z1* conformer. The Gibbs free energy difference (ΔΔG‡, yellow) is calculated as the difference between the Boltzmann weighted average of the *Z* conformers and the lowest TS/MECP energy. The molecules are sorted by Hammett parameter σ values. Dotted lines between energy levels have no physical meaning but are shown as visual guide.

Table S4. Calculated relative Gibbs free energies (ΔG in kcal mol^{-1}) of all *Z*, *E*, TS, and MECPs structures of both conformers (1 and 2) at the ωB97X-D/def2-TZVP@SMD(DMSO) level of theory.

compound	Z1	Z2	TS_{iAr} (1)	TS_{iAr} (2)	TS_{iPy} (1)	TS_{iPy} (2)	M_1 (1)	M_1 (2)	M_2 (1)	M_2 (2)	TS_r (1)	TS_r (2)	E1	E2
**3r**	0.0	0.1	29.7	31.3	28.4	28.4	20.1	21.2	18.8	19.4	38.3	38.4	-13.4	-11.9
**3s**	0.0	0.1	28.8	29.6	27.8	28.0	22.0	21.4	19.4	20.0	36.8	26.8	-13.1	-11.8
**3f**	0.0	0.3	26.2	27.6	27.9	27.7	22.1	21.5	21.1	20.7	34.6	34.9	-12.8	-11.6
**3m**	0.0	0.3	24.0	25.2	27.5	27.6	22.0	22.1	20.5	20.9	31.3	30.4	-12.8	-12.2
**3a**	0.0	0.0	24.4	25.7	27.8	27.6	22.8	21.8	21.1	21.0	32.4	32.3	-12.6	-11.6
**3e**	0.0	0.1	23.6	24.4	27.4	27.4	21.8	21.8	19.5	21.0	30.8	29.6	-13.3	-12.3
**3c**	0.0	-0.2	24.2	25.6	26.9	26.9	22.2	22.3	19.6	21.0	30.9	31.2	-12.8	-12.2
**3p**	0.0	0.2	22.0	23.5	26.7	26.9	22.2	21.5	19.5	19.8	28.9	27.4	-12.3	-11.7
**3g**	0.0	-0.5	20.4	21.9	26.6	26.6	22.6	22.9	20.9	21.8	29.5	26.8	-12.6	-12.0

Atomic charges of relevant atoms were evaluated by Mulliken population analysis and are shown in Figure S5.

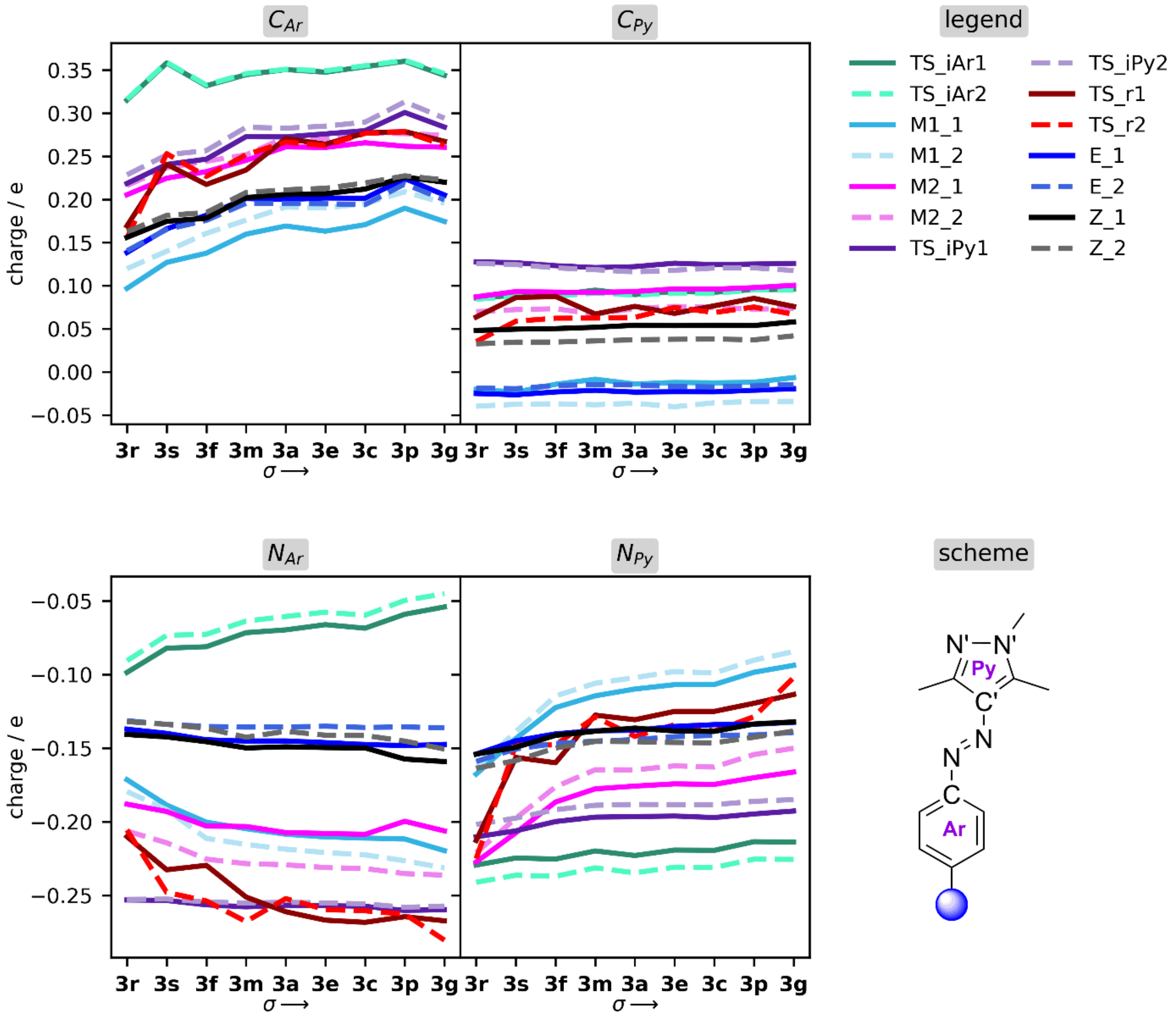

Figure S5. Plotted Mulliken charges of the N=N azo bond nitrogen atoms and their neighboring carbon atoms.

The Wiberg bond index (WI) of the N=N azo bond, as a measure of electron delocalization, was calculated with NBO Version 3.1[18] implemented in the *Gaussian 16*[11] suite and is shown in Figure S6.

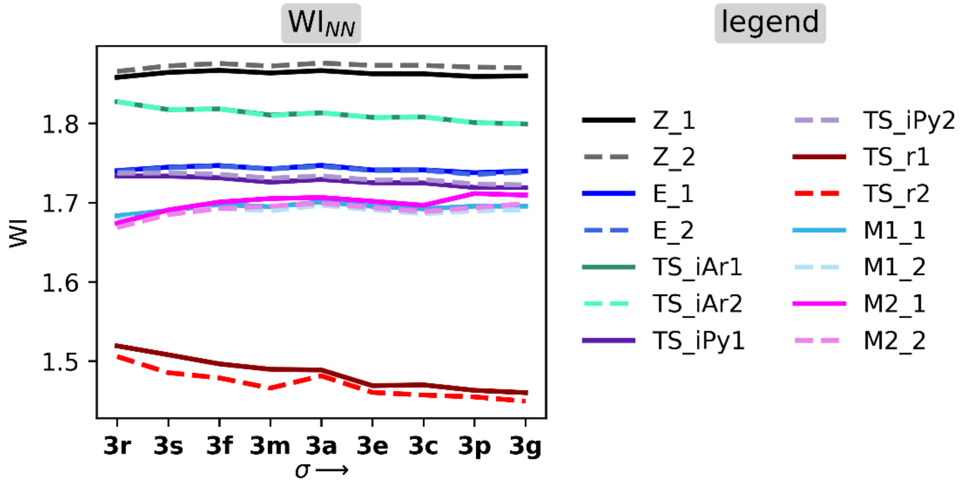

Figure S6. Wiberg bond index (WI) of the N=N azo bond.

The absorption spectra were calculated by vertical excitations from the ground state geometry of **3a**-Z and **3a**-E to the lowest lying 30 singlet states using the time-dependent version of DFT implemented in the *Gaussian 16*[11] suite. The calculated S_1 and S_2 absorption maxima λ_{max} compared to the experimental results are given in Table S5.

Table S5. Calculated S_1 and S_2 absorption maxima λ_{max} for compound **3a** compared to the experimental results.

**compound**	**S_1 Calc. λ_{max} / eV (nm)**	***f_{osc}***	**Exp. λ_{max} / nm**	**S_2 Calc. λ_{max} / eV (nm)**	***f_{osc}***	**Exp. λ_{max} / nm**
**3a**-E1	2.94 (422)	0.00	425	3.88 (320)	0.93	340
**3a**-E2	2.91 (426)	0.00		3.79 (327)	0.88	
**3a**-Z1	2.83 (439)	0.05	442	4.41 (281)	0.26	296
**3a**-Z2	2.82 (440)	0.05		4.34 (286)	0.17	

The rate constants for all four mechanisms were calculated according to the conventional (for $Path_{iAr}$, $Path_{iPy}$, and $Path_r$) and non-adiabatic transition state theory (for $Path_{rT1}$), as described by Singer et al.[5] The overall rate is calculated as the sum over the rate of all four possible mechanisms ($Path_{iAr}$, $Path_{iPy}$, $Path_r$, $Pathr_{T1}$) for each conformer (1 and 2), which are then Boltzmann weighted (w_1, w_2) according to their Z-isomer energies (Equation S5). The calculated shares of all eight paths to the overall rate constant and the half-lives $\tau_{1/2}$ are given in Table S6.

$$k_{total}(T) = w_1 \sum_{i}^{Paths} k_{1,i}(T) + w_2 \sum_{i}^{Paths} k_{2,i}(T)$$

Equation S5. Equation to calculate the overall rate constants $k_{total}(T)$ from the Boltzmann weighted summed rate constant $k(T)$ for each conformer (indicated by indices 1 and 2).

Table S6. Calculated shares (in %) of all eight paths to the overall rate constant, the overall rate constant k (in s^{-1}), and the half-lives $\tau_{1/2}$ (in h).

compound	$Path_{iAr}$ (1) [%]	$Path_{iAr}$ (2) [%]	$Path_{iPy}$ (1) [%]	$Path_{iPy}$ (2) [%]	$Path_{rT1}$ (1) [%]	$Path_{rT1}$ (2) [%]	$Path_{r}$ (1) [%]	$Path_{r}$ (2) [%]	k [s^{-1}]	$\tau_{1/2}$ [h]
**3r**	0.0	0.0	0.0	0.0	78.2	21.8	0.0	0.0	1.08E-04	0.83
**3s**	0.0	0.0	0.1	0.1	22.1	77.2	0.0	0.5	1.34E-05	7.39
**3f**	0.5	0.1	0.0	0.1	12.0	87.2	0.0	0.0	1.93E-05	5.74
**3m**	29.3	10.3	0.1	0.2	22.9	37.2	0.0	0.0	1.57E-05	5.72
**3a**	35.5	3.8	0.1	0.1	11.6	48.9	0.0	0.0	8.90E-06	10.82
**3e**	39.6	15.3	0.1	0.1	18.0	27.0	0.0	0.0	2.66E-05	3.54
**3c**	45.9	2.3	0.4	0.2	35.4	15.9	0.0	0.0	1.12E-05	9.24
**3p**	76.8	12.2	0.0	0.0	1.5	9.5	0.0	0.0	2.01E-04	0.42
**3g**	98.4	1.5	0.0	0.0	0.1	0.0	0.0	0.0	3.82E-03	0.03

In Figure 6 in the main manuscript, we plot the overall rate constants (including all four mechanisms) against the Hammett parameter. To highlight the importance of including the nonadiabatic $Path_{rT1}$ when comparing experimental and calculated rate constants, here we also plot the overall rate constants excluding $Path_{rT1}$ (Figure S7).

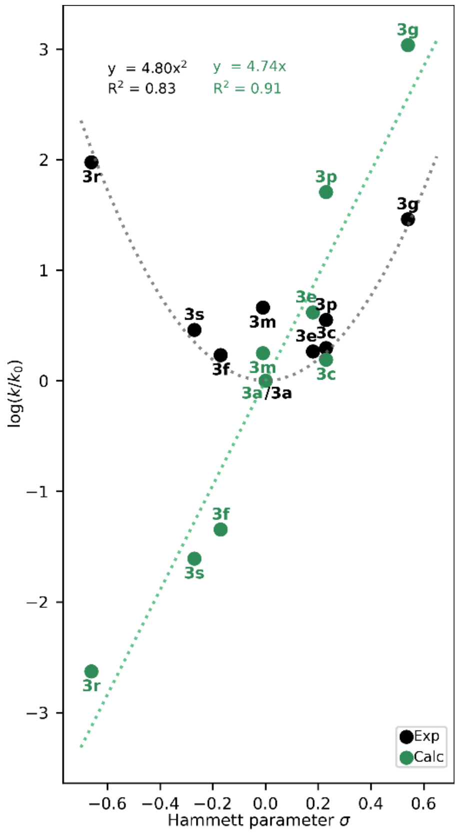

Figure S7. Hammett plot of the investigated nine arylazopyrazole compounds with experimental relative rate constants (black) and calculated ones but excluding the nonadiabatic $Path_{rT1}$ (green). The convex shape of the experimental plot is indicated with a parabolic fit (4.80*x^2) with compound **3a** set to be the minimum of the curve. The calculated rates are fitted by a linear function (4,74*x). The coefficient of determination (R^2) is given.

SECTION S7: OTHER POTENTIAL INFLUENCES

For the sake of completeness, we also discuss hydrogen bonding, concentration dependence, and solvent pH, as factors discussed in the literature that potentially can influence half-lives. For example, Venkataramani and co-workers[19] found a deviation of experimental and computational log(k/k_0) values for arylazo-*1H*-3,5-dimethylpyrazoles. In their work, NMR spectroscopy data suggested decreasing log(k/k_0) values with increasing Hammett parameter, while their calculations (not including all 4 relevant mechanisms) suggested the opposite. They explained the deviations mainly by the involvement of the pyrazole NH in hydrogen bonding. On one hand, the *Z*-conformer is stabilized through intermolecular hydrogen bonding; on the other hand, the solvent-assisted tautomerization and hydrazone formation has been shown before to increase the half-lives drastically.[20,21] The hypothesis of the effect of pyrazole hydrogen bonding seems to hold true for the *meta*-substituted molecules, as they were able to obtain the same trends for experiment and computation after methylating the pyrazole NH, making *meta*-substituted arylazo-1,3,5-trimethylpyrazoles. By contrast, our arylazopyrazoles (arylazo-1,3,5-trimethylpyrazoles) are *para*-substituted and trimethylated and therefore have no opportunity for the same pyrazole NH hydrogen bonds. Hence, we do not expect hydrogen bonding effecting our half-lives.

Concentration dependence has been also discussed to influence the thermal isomerization in azoheteroaryls.[22] However, while Calbo *et al.*[22] showed concentration dependence in azopyrroles, it could not be shown for arylazo-*1H*-3,5-dimethylpyrazole (**3a** without one methyl group); therefore, this argument has also been disregarded here.

Moreover, we note that Gibson *et al.*[23] published the acid-accelerated isomerization of **3a**, where using acids, the reverse isomerization of **3a** was speeded up from 19 days (no acid) to 80 min (chloroacetic acid) and to a point where the switch was too fast to detect with the method used (trifluoroacetic acid, hydrochloric acid). This suggests that the half-lives of arylazopyrazoles can be also manipulated with the pH of the environment.

For completeness, we mention here that the choice of the solvent can influence the photophysical properties of compounds. Simeth et al.[20] used a DMSO:H_2O mixture and found that slight changes of the solvent composition can drastically tune the properties of phenylazoindoles. However, while Calbo *et al.*[22] found the same effect for azopyrroles, they could not show it for other heteroaryl systems, including arylazopyrazoles.

REFERENCES:

[1] B. Haag, Z. Peng, P. Knochel, *Org Lett* **2009**, *11*, 4270–4273.

[2] D. V. Kosynkin, J. M. Tour, *Org Lett* **2001**, *3*, 993–995.

[3] M. Schönberger, M. Althaus, M. Fronius, W. Clauss, D. Trauner, *Nat Chem* **2014**, *6*, 712–719.

[4] E. Fischer, *J Phys Chem* **1967**, *71*, 3704–3706.

[5] N. K. Singer, K. Schlögl, J. P. Zobel, M. D. Mihovilovic, L. González, *J Phys Chem Lett* **2023**, *14*, 8956–8961.

[6] S. Grimme, J. Antony, S. Ehrlich, H. Krieg, *J Chem Phys* **2010**, *132*, 154104.

[7] J.-D. Chai, M. Head-Gordon, *Phys Chem Chem Phys* **2008**, *10*, 6615.

[8] F. Weigend, R. Ahlrichs, *Phys Chem Chem Phys* **2005**, *7*, 3297.

[9] F. Weigend, *Phys Chem Chem Phys* **2006**, *8*, 1057.

[10] A. V. Marenich, C. J. Cramer, D. G. Truhlar, *J Phys Chem B* **2009**, *113*, 6378–6396.

[11] M. J. Frisch, G. W. Trucks, H. B. Schlegel, G. E. Scuseria, M. A. Robb, J. R. Cheeseman, G. Scalmani, V. Barone, G. A. Petersson, H. Nakatsuji, X. Li, M. Caricato, A. V Marenich, J. Bloino, B. G. Janesko, R. Gomperts, B. Mennucci, H. P. Hratchian, J. V Ortiz, A. F. Izmaylov, J. L. Sonnenberg, D. Williams-Young, F. Ding, F. Lipparini, F. Egidi, J. Goings, B. Peng, A. Petrone, T. Henderson, D. Ranasinghe, V. G. Zakrzewski, J. Gao, N. Rega, G. Zheng, W. Liang, M. Hada, M. Ehara, K. Toyota, R. Fukuda, J. Hasegawa, M. Ishida, T. Nakajima, Y. Honda, O. Kitao, H. Nakai, T. Vreven, K. Throssell, J. A. Montgomery Jr., J. E. Peralta, F. Ogliaro, M. J. Bearpark, J. J. Heyd, E. N. Brothers, K. N. Kudin, V. N. Staroverov, T. A. Keith, R. Kobayashi, J. Normand, K. Raghavachari, A. P. Rendell, J. C. Burant, S. S. Iyengar, J. Tomasi, M. Cossi, J. M. Millam, M. Klene, C. Adamo, R. Cammi, J. W. Ochterski, R. L. Martin, K. Morokuma, O. Farkas, J. B. Foresman, D. J. Fox, **2019**.

[12] S. Axelrod, E. Shakhnovich, R. Gómez-Bombarelli, *ACS Cent Sci* **2023**, *9*, 166–176.

[13] F. Neese, F. Wennmohs, U. Becker, C. Riplinger, *J Chem Phys* **2020**, *152*, 224108.

[14] F. Neese, *WIREs Comput Mol Sci* **2022**, DOI 10.1002/wcms.1606.

[15] U. Ekström, L. Visscher, R. Bast, A. J. Thorvaldsen, K. Ruud, *J Chem Theory Comput* **2010**, *6*, 1971–1980.

[16] S. Mai, M. Richter, M. Heindl, M. F. S. J. Menger, A. Atkins, M. Ruckenbauer, F. Plasser, L. M. Ibele, S. Kropf, M. Oppel, P. Marquetand, L. Gonzalez, *SHARC2.1: Surface Hopping Including Arbitrary Couplings — Program Package for Non-Adiabatic Dynamics* **2019**.

[17] The PHAIDRA repository number o:2139404 contains the supplementary XYZ-structures for this paper. These data are provided free of charge by the permanent secure storage of digital assets at the University of Vienna.They can be found by DOI 10.25365/phaidra.691 or under https://phaidra.univie.ac.at/o:2139404.

[18] E. D. Glendening, A. E. Reed, J. E. Carpenter, F. Weinhold, *NBO Version 3.1.*

[19] S. Devi, M. Saraswat, S. Grewal, S. Venkataramani, *J Org Chem* **2018**, *83*, 4307–4322.

[20] N. A. Simeth, S. Crespi, M. Fagnoni, B. König, *J Am Chem Soc* **2018**, *140*, 2940–2946.

[21] J. Otsuki, K. Suwa, K. K. Sarker, C. Sinha, *J Phys Chem A* **2007**, *111*, 1403–1409.

[22] J. Calbo, C. E. Weston, A. J. P. White, H. S. Rzepa, J. Contreras-García, M. J. Fuchter, *J Am Chem Soc* **2017**, *139*, 1261–1274.

[23] R. S. L. Gibson, J. Calbo, M. J. Fuchter, *ChemPhotoChem* **2019**, *3*, 372–377.
